# Mechanism of IFITM1 regulating epidural scar hyperplasia after laminectomy through SMAD3/CBR4 pathway

**DOI:** 10.3389/fimmu.2025.1628970

**Published:** 2025-09-03

**Authors:** Haoran Wang, Zekai Zhu, Jun Liu

**Affiliations:** ^1^ Department of Orthopaedics, Tongji Hospital of Tongji University, School of Medicine, Tongji University, Shanghai, China; ^2^ Department of Orthopaedics, The Second Affiliated Hospital of Nanjing Medical University, Nanjing, China

**Keywords:** adipocyte, CBr4, epidural scar hyperplasia, fibroblast, IFITM1, Smad3

## Abstract

**Background:**

Epidural scar hyperplasia is a prevalent complication post-laminectomy, contributing significantly to persistent low back pain and other symptoms, ultimately undermining surgical outcomes. Previous studies have identified fibroblast proliferation and differentiation, as well as adipocyte fibrosis, as central to this process, though the precise mechanisms remain elusive.

**Methods:**

A model of laminectomy was established using wild-type mice and IFITM1-KO mice. Methods such as HE staining and Masson staining were employed to assess the degree of fibrosis in the postoperative wound area of the mice. Immunofluorescence and Western blot were performed to verify the localization of IFITM1 and fibronectin. NIH-3T3 fibroblast cells and primary fibroblast cell models were established, and immunoblotting was used to detect changes in the expression levels of fibronectin, P-smad3, smad3, and IFITM1. Subsequently, co-immunoprecipitation was conducted to preliminarily demonstrate that CBR4 is a related protein of IFITM1. The amounts of adipose tissue and CBR4 in the postoperative wound area were compared between wild-type and IFITM1-KO mice in the laminectomy model. CBR4 localization was examined using immunofluorescence, followed by the establishment of an in vitro adipocyte model, where Oil Red O staining and other methods were utilized to confirm the process of adipocyte fibrosis and the roles of IFITM1/CBR4 therein.

**Results:**

In a murine laminectomy model, fibroblast proliferation, activation, and adipocyte fibrosis were found to exacerbate epidural scar formation. IFITM1, a critical protein regulating cell proliferation, is expressed in fibroblasts. The proliferation and activation of fibroblasts, characterized by high IFITM1 expression, were inhibited by suppression of the SMAD3 signaling pathway. *In vivo* studies revealed a reduction in epidural fibrosis following laminectomy in the absence of IFITM1. Additionally, CBR4, a protein associated with IFITM1 and involved in fatty acid synthesis, showed reduced expression in adipocytes under inflammatory conditions, triggering their transformation into fibroblasts, a process regulated by IFITM1. Our animal experiments also confirmed the presence of adipose tissue within epidural scars, with IFITM1 deficiency correlating with reduced adipose tissue and increased CBR4 expression.

**Conclusion:**

These findings demonstrate that IFITM1 inhibits fibroblast proliferation and differentiation *via* SMAD3 signaling suppression and modulates adipocyte fibrosis by regulating CBR4 expression, thereby influencing epidural scar hyperplasia post-laminectomy.

## 1 Introduction

Laminectomy is a widely performed spinal surgical procedure, commonly employed as the primary treatment for conditions such as disc herniation, spinal stenosis, intraspinal tumors, lumbar spondylolisthesis, and lumbar fractures (1–6). However, epidural fibrosis following laminectomy often impairs surgical outcomes, contributing to lumbar surgery failure syndrome (FBSS). Approximately 8% to 40% of patients undergoing lumbar laminectomy develop FBSS, with some experiencing severe symptoms that require secondary surgical intervention (7, 8). The exact mechanisms behind epidural fibrosis remain poorly understood, highlighting the critical need to explore strategies for preventing its occurrence in spinal surgery.

Interferon-induced transmembrane protein 1 (IFITM1), a member of the interferon-induced transmembrane protein family, has been implicated in various biological processes, including anti-proliferation, cell adhesion regulation, and immune monitoring in digestive tract cancers such as esophageal, gastric, liver, and colorectal cancers (9). Previous research has established IFITM1’s involvement in the IFN-γ signaling pathway, which inhibits cell growth (10). Upregulation of IFITM1 is essential for IFN-γ’s ability to suppress cellular proliferation (11, 12). As a pivotal signaling pathway in fibrotic diseases, the extracellular signal-regulated kinase (ERK) pathway promotes disease progression upon activation (13–16), and IFITM1 has been shown to inhibit ERK kinase activity (12). Moreover, IFITM1’s interaction with Caveolin-1 enhances ERK inhibition, and reduced Caveolin-1 expression can activate the ERK pathway in NIH-3T3 fibroblast cells (12, 17). The inhibitory effect of betulinic acid (BA) on fibroblast proliferation has been linked to the upregulation of interferon (IFN)-induced genes, including IFIT1, IFITM1, IFI6, MX1, and OAS2 (18). Despite these insights, the role of IFITM1 in epidural scar hyperplasia has not been previously investigated. In this study, the absence of IFITM1 exacerbated epidural fibrosis in a murine model. Cytological experiments revealed a reduction in IFITM1 expression during the proliferation and differentiation of fibroblasts into myofibroblasts. Additionally, inflammatory factors were found to activate the SMAD3 signaling pathway, a key regulator of fibrotic processes, to promote this transformation. Notably, high levels of IFITM1 were shown to inhibit SMAD3 pathway activation, suggesting its critical role in modulating fibrotic responses.

**Figure 1:**
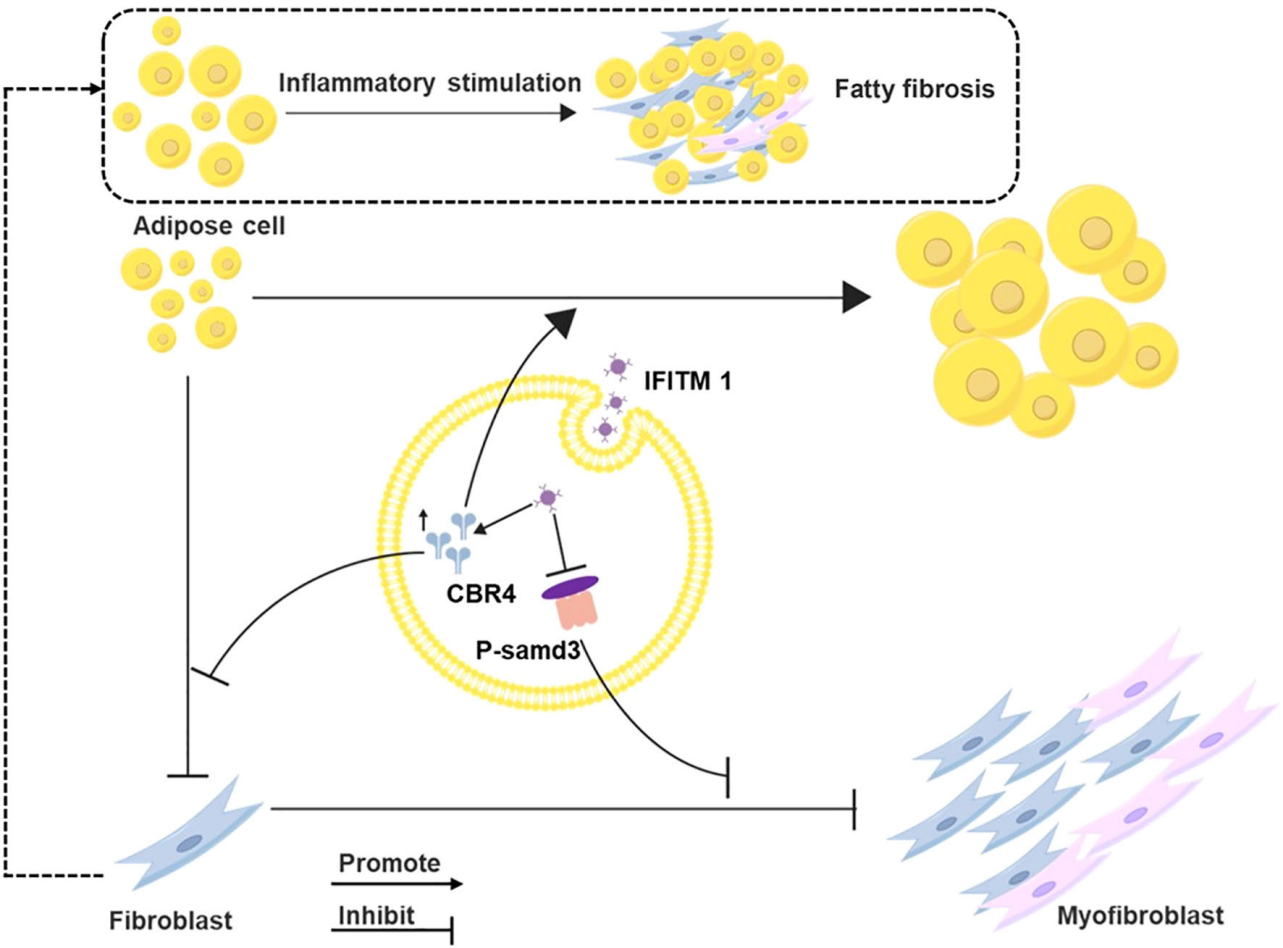
Mechanism diagram of IFITM regulating epidural scar hyperplasia through SMAD3/CBR4 IFITM1 can inhibit the activation of smad3 signal pathway, and then inhibit the proliferation and mature differentiation of fibroblasts; IFITM1 can also increase the expression of CBR4 protein, thus inducing the increase of fatty acid synthesis, leading to the increase of adipose tissue, and inhibiting the transformation of adipocytes into fibroblasts.

CBR4 plays a pivotal role in fatty acid synthesis (19, 20). Adipogenesis is the process by which adipocytes uptake free fatty acids and glycerol from the bloodstream to synthesize triacylglycerol for storage, whereas lipolysis refers to the breakdown of triacylglycerol into free fatty acids and glycerol, which are released into the bloodstream and utilized by other organs for energy (21–23). Thus, CBR4 promotes adipocyte proliferation and hypertrophy, contributing to increased adipose tissue. While adipocytes have been shown to regulate metabolism and influence inflammation (24), their role in tissue repair and epidural fibrosis remains poorly understood. Adipocytes can serve as a source of myofibroblasts, thereby participating in fibrotic processes, particularly in skin-related diseases (25–27). In the present study, a reduction in adipose tissue was observed within epidural scars following IFITM1 gene knockout (KO). Furthermore, IFITM1 regulated CBR4 expression, modulating the transformation of adipocytes into fibroblasts and myofibroblasts. High IFITM1 expression was associated with increased CBR4 levels, which mitigated adipocyte fibrosis, while IFITM1 inhibition led to reduced CBR4 expression, thereby exacerbating adipocyte fibrosis. In conclusion, our results confirm that IFITM1 suppresses fibroblast proliferation and differentiation *via* the SMAD3 signaling pathway. Moreover, IFITM1, in concert with CBR4, promotes adipose tissue synthesis and reduces adipocyte fibrosis, ultimately alleviating epidural fibrosis (**Figure 1**). The regulation of fibroblast and adipocyte proliferation and differentiation through the SMAD3/CBR4 pathway offers a novel approach to addressing epidural scar hyperplasia.

## 2 Materials and methods

### 2.1 Experimental animals

Wild-type male C57BL/6J mice, aged 6 to 8 weeks, were obtained from the Experimental Animal Center of Yangzhou University, China. IFITM1-KO mice, also on a C57BL/6J background, were provided by Dr. Mingshun Zhang from the Department of Immunology at Nanjing Medical University. All experimental procedures involving animals were reviewed and approved by the Animal Protection and Use Committee at Nanjing Medical University (IACUC-2412014).

For the study, 3 to 5 male wild-type C57BL/6J mice, aged 6 to 8 weeks and weighing 23 to 25 grams, were selected. Anesthesia was induced with an intraperitoneal injection of xylazine (Medistar, Ascheberg, Germany), diluted in 100 μL of physiological saline to a dose of 10 mg/kg. During the laminectomy, the mice were positioned prone on the operating table, and a midline skin incision was made to expose the T12-L2 lamina. After removing the spinous process, the dura mater was accessed, and the T12-L2 lamina was excised with a rongeur. The incision was then closed by suturing the spinal fascia, muscle, and skin (28, 29).

To investigate the role of IFITM1 in epidural fibrosis pathogenesis, IFITM1-KO mice were generated using the protocol for wild-type mice, with 3 to 5 mice per group as described earlier. All mice were SPF grade and housed in the Animal Center of Nanjing Medical University before and after modeling in a positive-pressure barrier environment.

The experimental design was optimized to minimize animal use. Following the modeling phase, all mice received an intraperitoneal injection of xylazine (Medistar, Ascheberg, Germany), diluted in 100 μL of physiological saline at a dose of 10 mg/kg to induce anesthesia. Once anesthetized, the head and neck were stabilized using a rigid rod or with the thumb and forefinger, while the tail or hind limb was held with the other hand. Cervical vertebrae dislocation was then induced by swiftly pulling the head back and forth, resulting in euthanasia.

### 2.2 Cell extraction and culture

The NIH-3T3 mouse fibroblast cell line (ATCC, CRL-1658) was cultured in complete medium containing 5% fetal bovine serum (Lonsera) and 1% penicillin/streptomycin (HyClone, GE). Cultures were maintained at 37°C in a 5% CO_2_ incubator. Following initial culture, NIH-3T3 cells were seeded in 24-well plates at a density of 1 × 10^5^ cells per well. The following day, the cells were treated with TGF-β or phosphate-buffered saline (PBS) (HyClone). If necessary, IFITM1 plasmid transfection reagent (MG50876-ACG, Beijing Yiqiao) was added to the culture medium at the appropriate working concentration. Cells were harvested 24 hours later for further analysis (29).

For primary mouse fibroblast extraction and culture, adult wild-type and IFITM1-KO mice were euthanized *via* cervical dislocation. The carcasses were then immersed in 75% ethanol for 5 minutes, followed by three washes with PBS. The mice were placed prone on the operating table, and the skin was shaved with a surgical razor. A section of the tail tip or ear skin was excised and submerged gently in PBS in a flat dish to remove adhering tissues, such as adipose and vascular tissues. The specimens were washed three times with PBS and subjected to enzymatic digestion at 37°C. After digestion, the epidermis was discarded, and the remaining tissue was washed with PBS three additional times. The tissue was then minced, washed three times with PBS, and all PBS was removed. The pretreated tissue blocks were placed evenly on the bottom of a culture dish, and complete culture medium (10% fetal bovine serum from Lonsera and 1% penicillin/streptomycin from HyClone, GE) was added. The culture was incubated at 37°C in a 5% CO_2_ atmosphere. On day 2, additional culture medium was added to ensure the tissue block remained submerged, with medium changes every 2-3 days. By day 7, numerous cells were observed at the periphery of the tissue block. After 2-3 passages, relatively pure primary fibroblasts were obtained. Based on prior experimental protocols, primary fibroblasts were cultured in 24-well plates at a density of 1 × 10^5^ cells per well. The following day, the cells were treated with TGF-β for the specified duration (29).

Mouse adipocytes were differentiated from mouse embryonic fibroblasts (3T3-L1), which were obtained from Wuhan Servicebio Company (STCC20001). The complete culture medium consisted of 10% fetal bovine serum (Lonsera) and 1% penicillin/streptomycin (HyClone, GE) at a final concentration of 5%. Cells were cultured in an incubator at 37°C with 5% CO_2_. 3T3-L1 fibroblasts were seeded in 24-well plates at a density of 1 × 10^5^ cells per well. Upon reaching 70% confluence, dexamethasone (50-02-2, MedChemExpress) was added at a concentration of 1 µM (100 µL of dexamethasone stock solution per 100 mL of culture medium) to induce differentiation. Adipocyte-like differentiation was monitored by oil red O staining to assess lipid accumulation within the cells (30, 31). Following differentiation, 3T3-L1 cells were treated with TGF-β cytokines or an equivalent volume of PBS (HyClone). IFITM1 plasmid transfection reagent (MG50876-ACG, Beijing Yiqiao) or IFITM1 siRNA reagent (2407111M46, General Biotechnology) was added to the culture medium at the appropriate working concentration. Cells were harvested after 24 hours for subsequent analysis.

### 2.3 HE staining

Thirty days post-laminectomy, the mice were euthanized, and tissues from the surgical site were harvested. These tissues were fixed in 4% paraformaldehyde for 48 hours. After fixation, the tissues were decalcified in 10% neutral EDTA for approximately four weeks. The tissues were then subjected to dehydration using an ethanol gradient, followed by immersion in xylene for transparency and embedding in paraffin. Tissue sections (5 µm thick) were cut using a microtome and placed onto glass slides. Dewaxed sections were stained with hematoxylin for 5 to 10 minutes, rinsed in distilled water for 1 to 2 minutes, and rapidly differentiated in 1% hydrochloric acid-ethanol for 5 minutes. After washing with distilled water, the sections were immersed in 0.6% ammonia water to achieve a blue coloration, then stained with eosin for 5 minutes. The slides were dehydrated in anhydrous ethanol three times, followed by two rounds of xylene for transparency, each lasting 10 minutes. Finally, the slides were sealed with neutral mounting medium and observed under an optical microscope for subsequent analysis.

### 2.4 Masson staining

Paraffin sections were sequentially immersed in xylene I and xylene II for 10 minutes each. The sections were then placed in anhydrous ethanol I and anhydrous ethanol II for 5 minutes each, followed by immersion in 75% ethanol for an additional 5 minutes. Subsequently, the sections were immersed in potassium dichromate solution overnight. Afterward, they were stained with hematoxylin dye for 3 to 5 minutes, washed with distilled water for 1 to 2 minutes, and rapidly differentiated using 1% hydrochloric acid alcohol for a few minutes. The sections were then washed with distilled water, and the solution reverted to blue. After differentiation, the sections were immersed in ponceau acid fuchsin dye solution for 5 to 10 minutes, followed by immersion in phosphomolybdic acid solution for 2 minutes. The sections were then placed in preheated aniline blue dye for 5 minutes, followed by differentiation in 1% glacial acetic acid three times. The sections were then dehydrated with anhydrous ethanol and placed in xylene for 5 minutes before being sealed with neutral gum. Images were captured and analyzed using an upright optical microscope.

### 2.5 Immunohistochemical staining

The dewaxing of paraffin sections to water followed the same protocol as hematoxylin and eosin (HE) staining. Dewaxed sections were immersed in citrate buffer (pH 6.0) for antigen retrieval, followed by three washes with PBS. The sections were then incubated in a 3% hydrogen peroxide (H_2_O_2_) solution at room temperature for 30 minutes, followed by washing with distilled water. Next, 5% goat serum was applied to the selected areas, and the sections were incubated and sealed at room temperature for one hour. Fibronectin (1:100, GB114491, Servicebio) and α-smooth muscle actin (α-SMA) (1:100, GB111364, Servicebio) antibodies were then added to the staining area. The sections were incubated with goat anti-rabbit IgG (1:200, GB23303, Servicebio) for one hour at room temperature. The freshly prepared DAB chromogenic reagent was applied to stain the tissue, followed by a gentle rinse with distilled water. The slides were then immersed in hematoxylin dye for 2 to 5 minutes, washed with distilled water, and differentiated in 1% hydrochloric acid alcohol. The tissue was then turned blue with 0.2% ammonia water, followed by another gentle rinse with distilled water. Finally, the slides were sealed with neutral resin, and microscopic examination was performed after the resin had completely solidified.

### 2.6 Immunofluorescence

To investigate the relationship between IFITM1 and fibrosis, immunofluorescence analysis was performed on tissue samples from the surgical area of mice. The embedded tissue was sectioned into 4-micron slices and incubated with primary antibodies: fibronectin (1:100, GB114491, Servicebio), IFITM1 (1:100, 60074-1-Ig, Proteintech), and CBR4 (13725-1-AP, Proteintech) at 4°C. After incubation, the slides were treated with secondary antibodies, goat anti-rabbit IgG (1:200, GB23303, Servicebio) and goat anti-mouse IgG (1:200, GB28303, Servicebio), at room temperature for one hour. DAPI was added for nuclear staining, and the samples were observed and photographed using a fluorescence inverted microscope.

To confirm that CBR4 can influence adipocyte fibrosis, cells were washed three times with PBS and fixed with 4% paraformaldehyde. The fixed adipocytes were incubated with fibronectin (1:100, GB114491, Servicebio) and CBR4 (13725-1-AP, Proteintech) at room temperature for one hour. The cells were then treated with a fluorescent secondary antibody (1:100, Alexa Fluor 488, G1231-25UL, Servicebio) for an additional hour. Images were captured using a fluorescence microscope.

### 2.7 Oil red O staining

For lipid droplet formation in adipocytes, Oil Red O staining was performed. Cells were washed three times with PBS, fixed in 4% paraformaldehyde for 15 minutes, and then incubated with Oil Red O solution (G1035-100ML, Servicebio) for 15 minutes. Afterward, cells were washed with PBS to remove any residual Oil Red O solution and observed under a microscope.

### 2.8 Western blot

For protein extraction from epidural scar tissue, a mixture of PMSF (1 mmol/L, ST 506, Beyotime) and RIPA buffer (P0013B, Beyotime) was prepared at a 1:100 ratio. Epidural scar tissue (20 µg) was weighed, and the protein lysis solution was added in a 1:10 ratio. The tissue was homogenized using a tissue homogenizer, and after centrifugation, the supernatant was collected into a new EP tube. Protein loading buffer was added at five times the volume of the sample and thoroughly mixed. Equal volumes of the sample were loaded onto a 10% sodium dodecyl sulfate-polyacrylamide gel for electrophoresis. After separation, the proteins were transferred to a polyvinylidene fluoride membrane, which was blocked with 5% bovine serum albumin at 27°C for 1 hour to prevent nonspecific binding. The membrane was washed three times with PBS containing Tween and incubated with primary antibodies: fibronectin (1:1000, GB114491, Servicebio), IFITM1 (1:100, 60074-1-Ig, Proteintech), α-SMA (1:1000, GB111364, Servicebio), and TGF-β (1:1000, GB115739, Servicebio). The membrane was then incubated with goat anti-rabbit IgG (1:5000, HRP, ab6721, Abcam), and protein detection was performed using enhanced chemiluminescence (ECL) reagent (36208ES60, Yeasen). β-actin was used as a loading control for normalization of protein expression.

Similarly, proteins from fibroblasts and adipocytes were extracted using a radioimmunoassay analysis buffer and quantified by Western blot analysis. The membrane was incubated with primary antibodies: fibronectin (1:1000, GB114491, Servicebio), IFITM1 (1:100, 60074-1-Ig, Proteintech), CBR4 (13725-1-AP, Proteintech), anti-β-actin (Abcam), P-Smad3 (1:1000, 80427-2-RR, Proteintech), and Smad3 (1:1000, 66516-1-lg, Proteintech). Expression levels of all proteins were normalized to β-actin levels.

### 2.9 Enzyme-linked immunosorbent assay

To assess collagen levels in the epidural region of mice, tissue samples from the surgical site were dissected into small fragments, centrifuged at 4°C and 10,000 × g for 10 minutes with lysis buffer. The supernatant was collected, and type I collagen concentration was quantified using a type I collagen ELISA kit (Beijing Tianlong Technology, Beijing, China).

### 2.10 Immunocoprecipitation

NIH-3T3 mouse fibroblasts were harvested, and a cell suspension was prepared at a concentration of 1 × 10^5^ cells in 20 to 30 µL of Binding Buffer, followed by the addition of a protease inhibitor. After incubation, the mixture was centrifuged to obtain the supernatant, which was diluted to 5 to 50 µg/mL. Magnetic beads (C0104, TargetMol^®^) were washed and combined with IFITM1 (1:200, 60074-1-Ig, Proteintech) and IgG (1:200, SA00001-2, Proteintech). The complex was washed, and 1× SDS-PAGE Loading Buffer (W B3002, NCM Biotechnology) was added. The supernatant was collected by centrifugation and analyzed by SDS-PAGE, followed by mass spectrometry and Immunoprecipitation (Co-IP) verification.

### 2.11 Statistical analysis

Statistical analysis was performed using SPSS version 22.0. Measurement data are presented as mean ± standard error of mean (± SEM). Group comparisons were made using t-tests or one-way ANOVA, with pairwise comparisons conducted *via* the Tukey method. A P-value of < 0.05 was considered statistically significant. This study adheres to the ARRIVE guidelines 2.0.

## 3 Results

### 3.1 Epidural fibrosis is related to IFITM1

**Figure 2:**
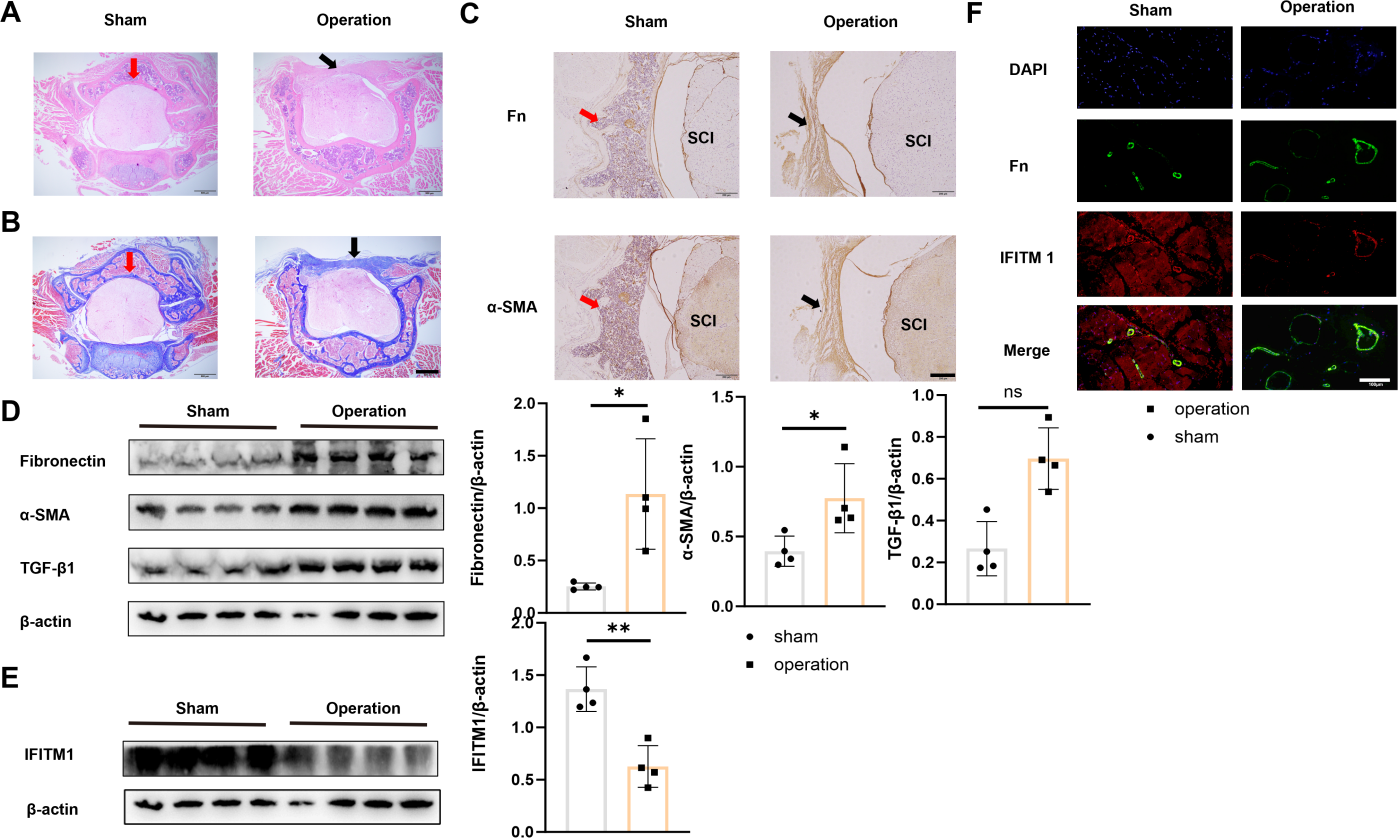
Epidural fibrosis is related to IFITM1 **(A)** Representative images of HE staining in wound area; The scale is 500 μm. **(B)** Representative images of Masson staining in wound area; The scale is 500 μm. **(C)** Immunohistochemical images of fibronectin and α-SMA in representative wound tissues; The scale is 100 μm. **(D)** The expression of fibronectin, α-SMA and TGF-β1 in wound area was analyzed by western blot. **(E)** The expression of IFITM1 in wound area was analyzed by western blot. **(F)** The imaging of fibronectin and α-SMA in fibroblasts in wound tissue was observed by immunofluorescence. The scale is 100 μm. Black arrow indicates epidural scar, red arrow indicates dura mater, and "SC" indicates spinal cord. *, P<0.05; **, P<0.01; ns, no statistical significance. Full-length blots/gels are presented in **Supplementary Figures 2D, E**.

To examine the changes in IFITM1 expression during epidural scar tissue hyperplasia, a mouse laminectomy model was established, consisting of a sham operation group and an experimental operation group. Histological analysis with HE and Masson staining revealed significant fibrotic lesions in the epidural space of mice in the operation group (**Figures 2A, B**). These findings were further confirmed by fibronectin and α-SMA histochemical staining (**Figure 2C**). Tissues from the surgical sites in both groups were collected and analyzed by Western blot, which revealed a downregulation of IFITM1 expression as epidural fibrosis developed (**Figures 2D, E**), a result also validated by immunofluorescence staining of postoperative tissues (**Figure 2F**).

**Figure 3:**
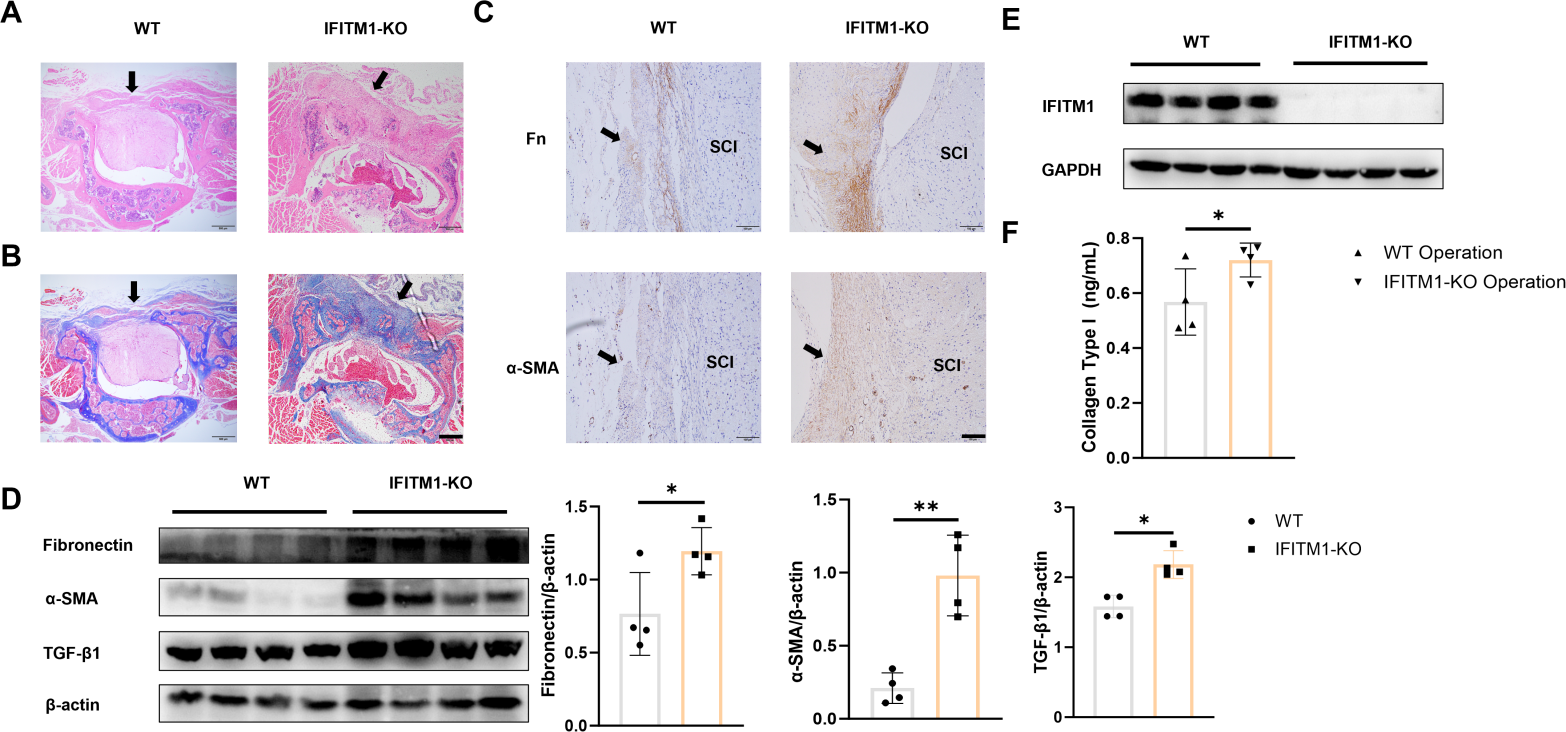
IFITM1 directly regulates the process of epidural scar hyperplasia **(A)** Representative images of HE staining in wound area; The scale is 500 μm. **(B)** Representative images of Masson staining in wound area; The scale is 500 μm. **(C)** Immunohistochemical images of fibronectin and α-SMA in representative wound tissues; The scale is 100 μm. **(D)** The expression of fibronectin ,α-SMA and TGF-β1 in wound area was analyzed by western blot. **(E)** The expression of IFITM1 in wound area was analyzed by western blot. **(F)** The content of type I collagen in wound tissue was detected by ELISA. Black arrow indicates epidural scar, and "SC" indicates spinal cord. *, P<0.05; **, P<0.001. Full-length blots/gels are presented in **Supplementary Figures 3D, E**.

To further explore the role of IFITM1 in epidural fibrosis following laminectomy, both wild-type and IFITM1-KO mice were used to establish the model. Thirty days post-laminectomy, IFITM1-KO mice exhibited significantly more extensive epidural fibrosis (**Figures 3A, B**). The levels of fibronectin and α-SMA in the epidural scar tissue of IFITM1-KO mice were elevated compared to wild-type mice (**Figure 3C**). Western blot analysis also revealed markedly higher expression of fibronectin, α-SMA, TGF-β1, and IFITM1 in the epidural tissue of IFITM1-KO mice compared to wild-type controls (**Figures 3D, E**). Furthermore, Collagen-I expression in the surgical region was significantly higher in IFITM1-KO mice (**Figure 3F**). These results suggest that IFITM1 is involved in epidural fibrosis post-spinal surgery and may play a role in negatively regulating the progression of fibrosis.

### 3.2 IFITM1 directly regulates the process of fibroblast proliferation and differentiation

**Figure 4:**
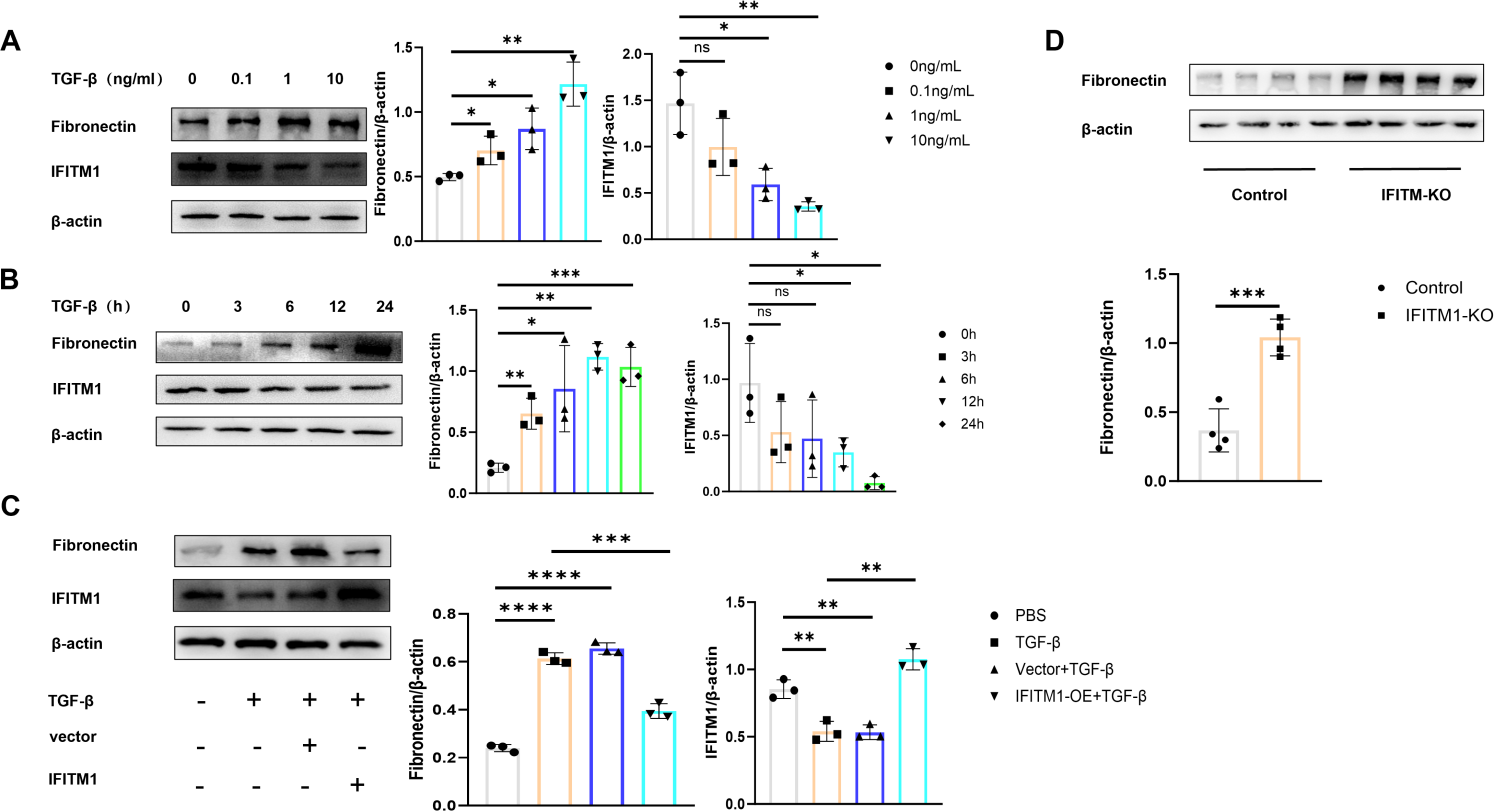
IFITM1 directly regulates the process of fibroblast proliferation and differentiation **(A, B)** The expression of fibronectin and IFITM1 in NIH-3T3 cells treated with TGF-β was analyzed by western blot. **(C)** The expression of fibronectin and IFITM1 in NIH-3T3 cells was analyzed by immunoblotting after 24 hours of treatment with TGF-β and IFITM1 plasmids. **(D)** The expression of fibronectin in primary fibroblasts treated with TGF-β and primary fibroblasts of IFITM1-KO were analyzed by western blot. *, P<0.05; **, P<0.01; ***, P<0.001; ns, no statistical significance. Full-length blots/gels are presented in **Supplementary Figures 4A1, B1, C1, D1**.

To investigate the direct impact of IFITM1 on fibroblast activity, NIH-3T3 fibroblasts were stimulated with TGF-β cytokines to simulate an inflammatory response *in vitro*. A concentration and time gradient of stimulation was applied. As the degree and duration of stimulation increased, a corresponding rise in fibroblast proliferation, differentiation, and maturation was observed. However, during this process, IFITM1 expression decreased (**Figures 4A, B**). To explore IFITM1’s regulatory role further, NIH-3T3 fibroblasts with elevated IFITM1 expression and primary fibroblasts from IFITM1-KO mice were analyzed. Proliferation and differentiation of NIH-3T3 fibroblasts overexpressing IFITM1 were significantly inhibited (**Figure 4C**), while primary fibroblasts from IFITM1-KO mice exhibited markedly enhanced proliferation and differentiation compared to wild-type fibroblasts (**Figure 4D**). These results suggest that IFITM1 negatively regulates fibroblast proliferation and differentiation, thereby influencing the progression of epidural fibrosis.

### 3.3 IFITM1 can regulate epidural fibrosis by inhibiting SMAD3 signaling pathway

**Figure 5:**
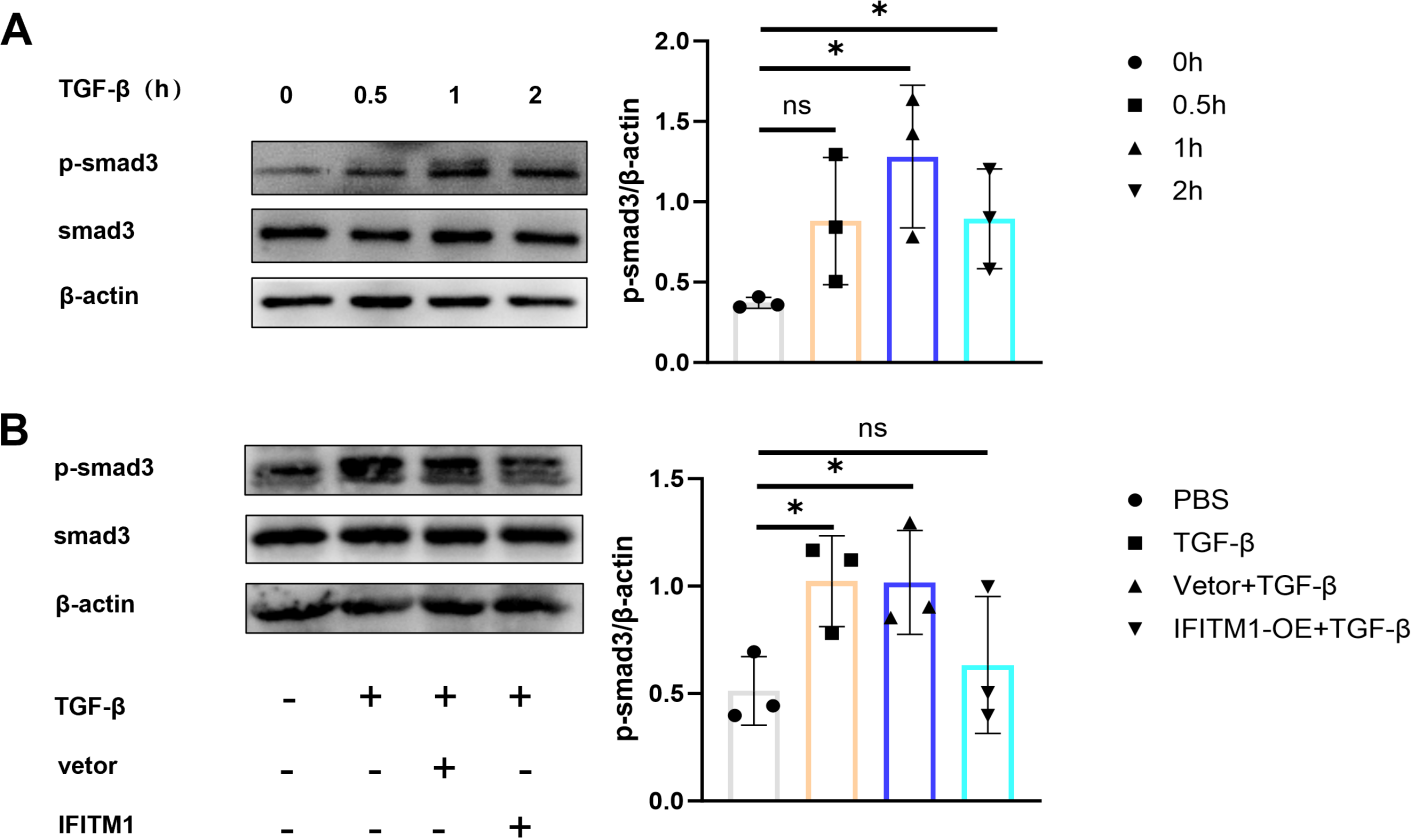
IFITM1 can regulate epidural fibrosis by inhibiting SMAD3 signaling pathway **(A)** The expression of p-smad3 in NIH-3T3 cells treated with 1 ng/ml TGF-β was analyzed by western blot. **(B)** The activation of p-smad3 in NIH-3T3 cells was analyzed by western blot after 24 hours of transfection withTGF-β and IFITM1 plasmids. *, P<0.05; ns, no statistical significance. Full-length blots/gels are presented in **Supplementary Figures 5A1, B1**.

The Smad3 signaling pathway plays a critical role in fibrosis development (32–34), and stimulation of NIH-3T3 fibroblasts with TGF-β cytokines activates the SMAD3 pathway, promoting fibroblast proliferation and differentiation (**Figure 5A**). In contrast, the SMAD3 signaling pathway in NIH-3T3 fibroblasts overexpressing IFITM1 was inhibited (**Figure 5B**). These results suggest that IFITM1 can inhibit the SMAD3 pathway in fibroblasts, thereby modulating fibroblast proliferation and differentiation processes.

### 3.4 IFITM1 can cooperate with CBR4 to regulate the process of epidural fibrosis

**Figure 6:**
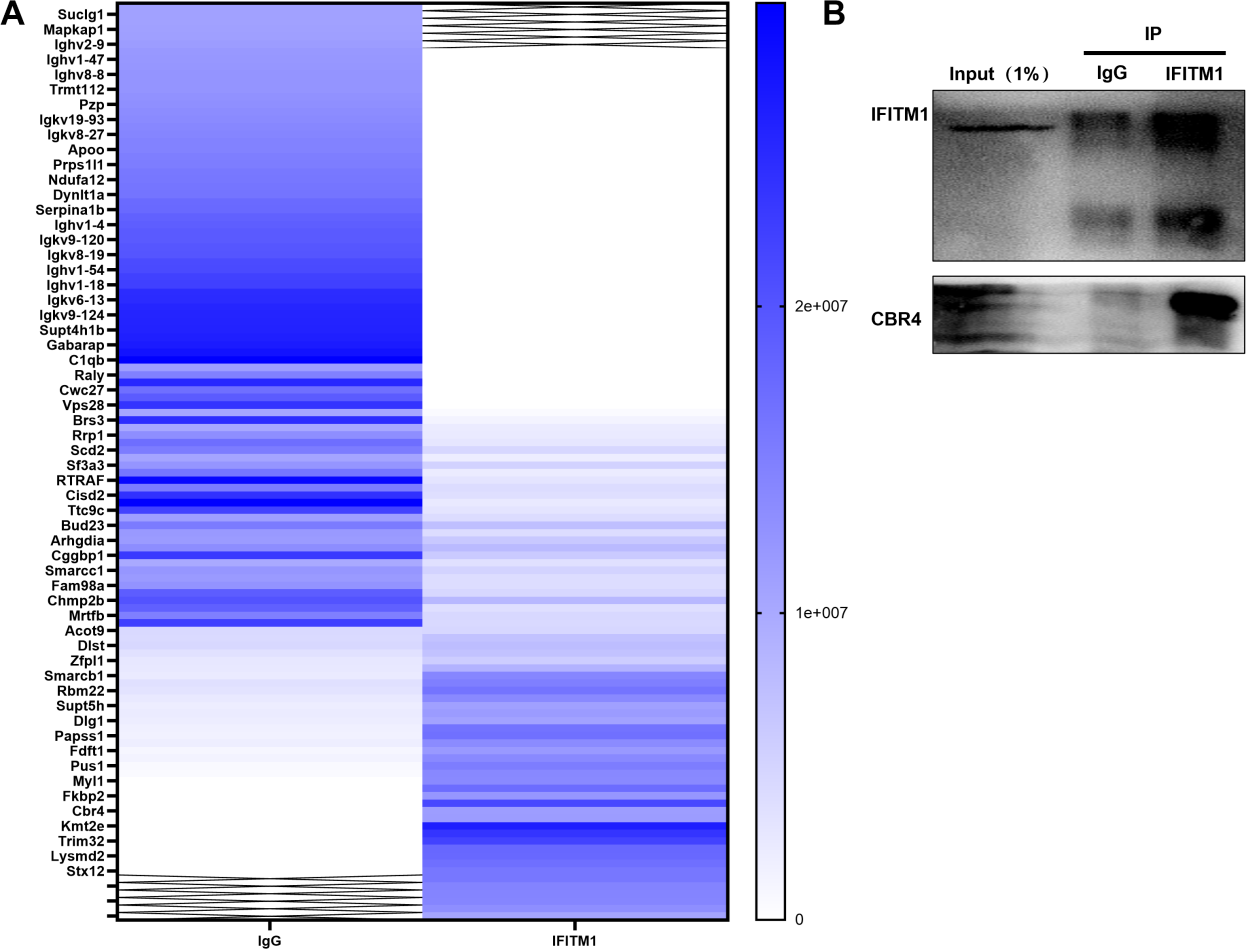
IFITM1 can cooperate with CBR4 to regulate the process of epidural fibrosis **(A)** Immunocoprecipitation experiment, lgG group and IFITM1 group were set up to explore IFITM1 related proteins.**(B)** Immunocoprecipitation (Co-ip)verified the results of mass spectrometry analysis, and CBR4 was the related protein of IFITM1. Full-length blots/gels are presented in **Supplementary Figure 6B**.

Mass spectrometry analysis of NIH-3T3 fibroblasts comparing the IgG group with the IFITM1 group revealed a significant positive correlation between IFITM1 and CBR4 (**Figure 6A**). Furthermore, co-immunoprecipitation (Co-IP) experiments confirmed that CBR4 interacts with IFITM1 (**Figure 6B**), providing preliminary evidence that IFITM1 may cooperate with CBR4 to regulate epidural fibrosis.

### 3.5 IFITM1 can interfere with the production of adipose tissue or cells by affecting the expression of CBR4

**Figure 7:**
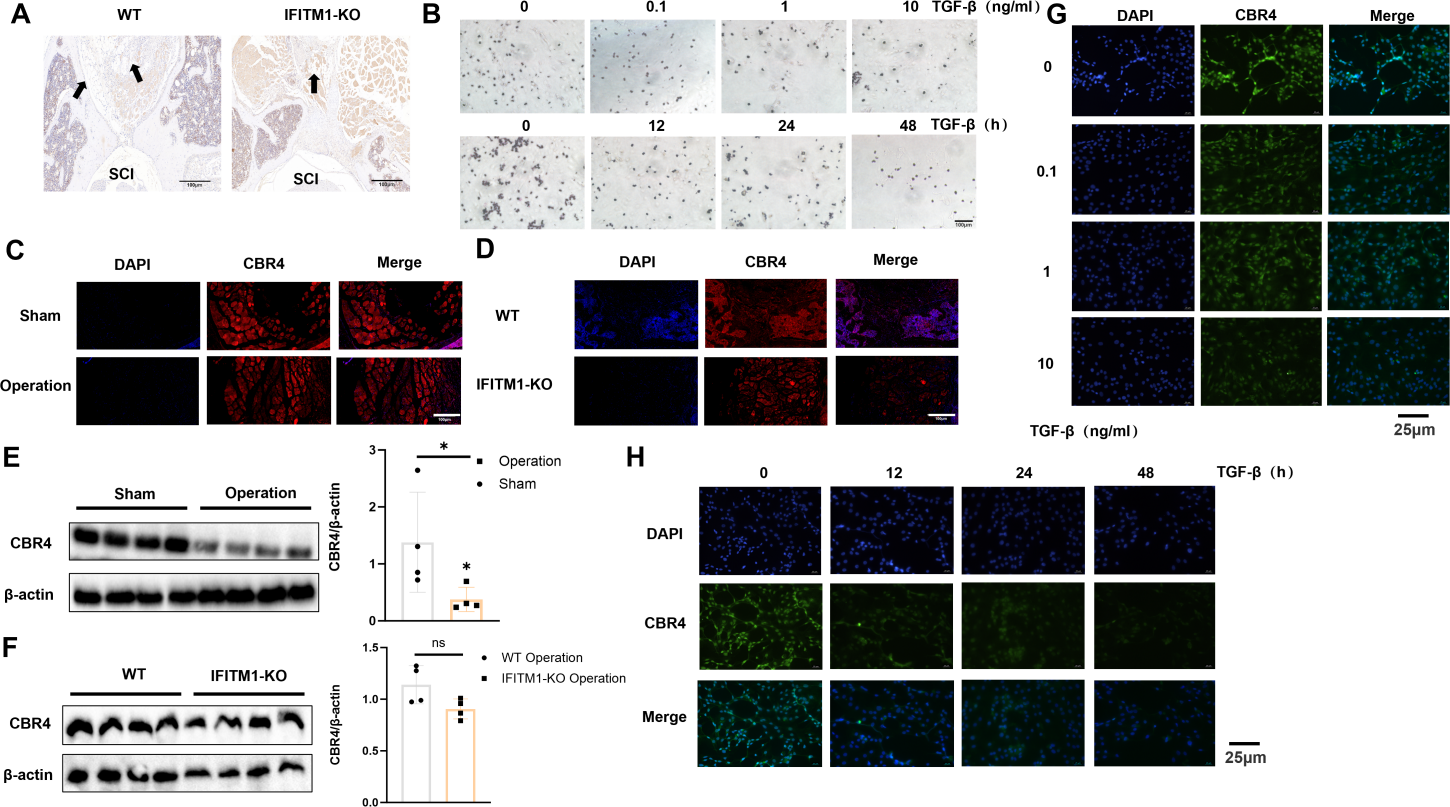
IFITM1 can interfere with the production of adipose tissue or cells by affecting the expression of CBR4 **(A)** Immunohistochemical images of fat tissue in representative wound tissues; The scale is 100 μm. **(B)** Analysis of lipid droplets in adipocytes treated with TGF-β by oil red O staining; The scale is 25 μm.**(C, D)** Immunofluorescence analysis of CBR4 expression in epidural tissue after laminectomy in mice; The scale is 100 μm.**(E, F)**Western blot analysis of CBR4 expression in epidural tissue after laminectomy in mice. (G-H) Immunofluorescence analysis of CBR4 expression in adipocytes treated with TGF-β. *, P<0.05; ns, no statistical significance. Full-length blots/gels are presented in **Supplementary Figures 7E, F**.

CBR4 is a key protein involved in fatty acid synthesis, promoting the production of fatty acids and the subsequent expansion of adipose tissue. To explore whether IFITM1 collaborates with CBR4 in regulating adipose tissue generation and its potential influence on epidural fibrosis, laminectomy models were established in both wild-type and IFITM1-KO mice. Histochemical staining of the surgical sites revealed the presence of adipose tissue, with IFITM1-KO mice showing significantly less adipose tissue compared to wild-type mice (**Figure 7A**). An *in vitro* model was also established by stimulating adipocytes directly with TGF-β cytokines, applying both concentration and time gradients. Oil Red O staining showed a marked reduction in lipid droplets following TGF-β stimulation, indicating a decrease in adipocyte numbers during this process (**Figure 7B**). Additionally, immunofluorescence staining of CBR4 in the postoperative areas of wild-type mice subjected to sham operations versus those that underwent surgery revealed significantly lower CBR4 expression in the surgical group (**Figure 7C**). To investigate the cooperation between IFITM1 and CBR4 in modulating adipose tissue generation, two experimental groups were established: a wild-type mouse operation group and an IFITM1-KO mouse operation group. CBR4 immunofluorescence staining of the postoperative areas in both groups revealed a marked reduction in the modeling area of IFITM1-KO mice compared to wild-type mice (**Figure 7D**). This finding further supports the relationship between IFITM1 and CBR4 at the experimental level. Additionally, Western blot analysis confirmed a consistent trend with the immunofluorescence results (**Figures 7E, F**). To further establish a positive correlation between CBR4 and adipocyte production, TGF-β stimulation experiments were conducted with varying concentrations and time gradients. The results demonstrated that CBR4 expression in adipocytes decreased as the intensity or duration of stimulation increased (**Figures 7G, H**). Inflammatory stimulation reduces the number of adipocytes, and fluorescence experiments revealed a decrease in CBR4 expression during this process. This evidence suggests that CBR4 contributes to adipocyte proliferation and expansion, processes modulated by IFITM1.

### 3.6 IFITM1 can cooperate with CBR4 to intervene in the process of adipocyte fibrosis and then regulate the process of epidural fibrosis

**Figure 8:**
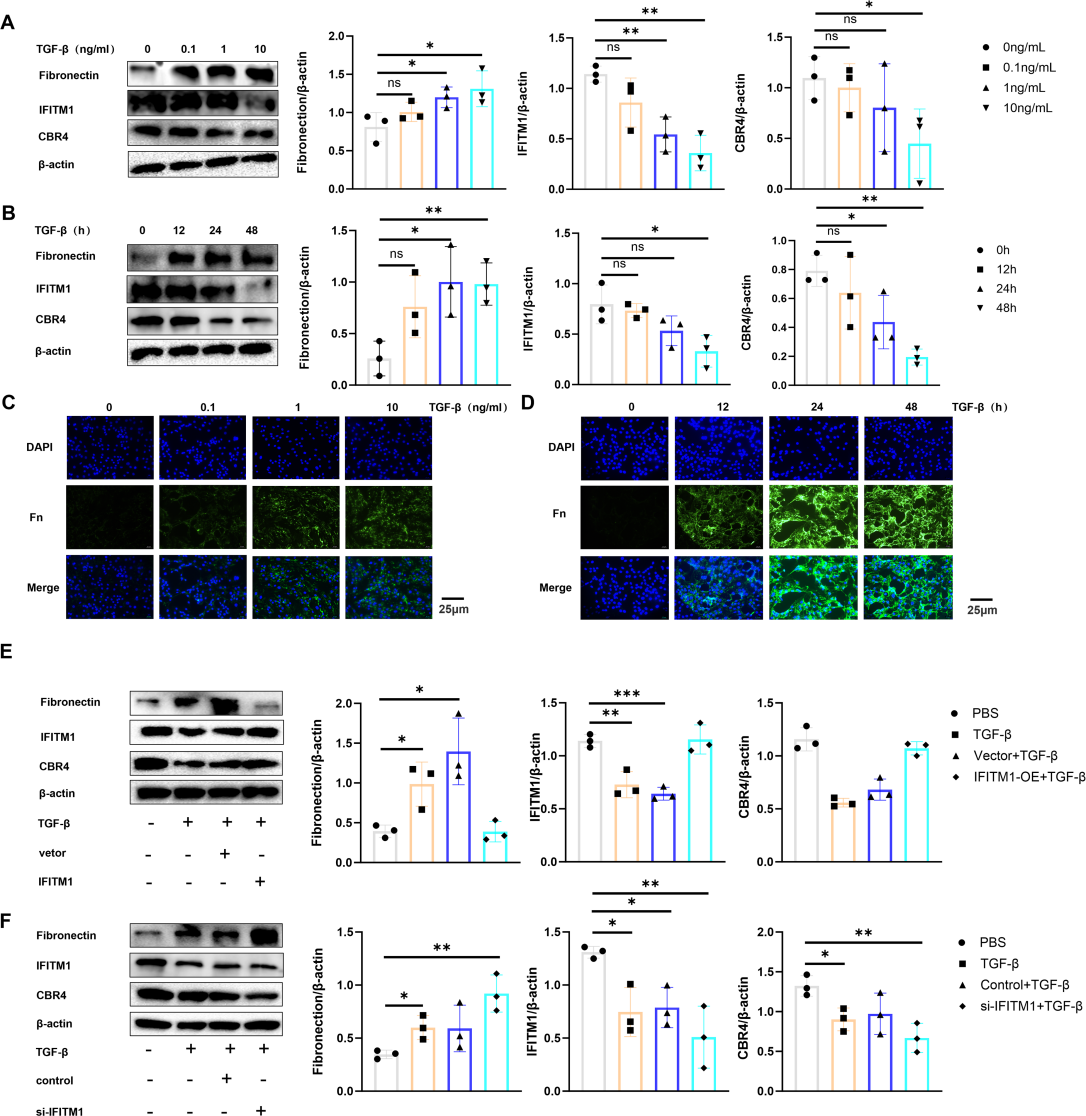
IFITM1 can cooperate with CBR4 to intervene the process of adipocyte fibrosis and then regulate the process of epidural fibrosis **(A, B)** The expression of fibronectin ,IFITM1 and CBR4 in adipocytes treated with 1ng/ml TGF-β was analyzed by western blot. **(C, D)** The expression of fibronectin in adipocytes treated with 1ng/mlTGF was analyzed by immunofluorescence. **(E)** western blot was used to analyze the expression of fibronectin ,IFITM1 and CBR4 in adipocytes after 24 hours of transfection with TGF-β and IFITM1 plasmids. **(F)** The expression of fibronectin ,IFITM1 and CBR4 in adipocytes treated with 1ng/ml TGF-β and si-IFITM1 was analyzed by western blot. *, P<0.05; **, P<0.01; ***, P<0.001; ns, no statistical significance. Full-length blots/gels are presented in Supplementary Figure **Supplementary Figures 8A1, B1, E1, F1**.

To investigate the regulation of adipocyte fibrosis by IFITM1 and CBR4, adipocytes were stimulated with TGF-β cytokines to simulate the inflammatory environment associated with fibrosis. The results showed that increased TGF-β stimulation or prolonged exposure led to upregulation of fibronectin expression in adipocytes, while IFITM1 and CBR4 expression decreased (**Figures 8A, B**). This preliminary evidence suggests that adipocyte fibrosis is regulated by IFITM1 and CBR4. Immunofluorescence staining further confirmed that fibronectin levels in adipocytes increased gradually with TGF-β stimulation (**Figures 8C, D**), supporting the notion that adipocytes undergo fibrosis in response to inflammatory signals, consistent with the Western blot findings. To further explore the role of IFITM1 in adipocyte fibrosis through CBR4 regulation, adipocytes with high and low expressions of IFITM1 were constructed. Adipocytes overexpressing IFITM1 also exhibited elevated CBR4 expression, and fibrosis was significantly reduced under inflammatory conditions (**Figure 8E**). In contrast, adipocytes with low IFITM1 expression showed reduced CBR4 levels, and fibrosis was exacerbated under similar inflammatory stimuli (**Figure 8F**). These results provide further evidence for the pivotal role of the IFITM1/CBR4 pathway in regulating adipocyte fibrosis, indicating that inhibition of this pathway intensifies the degree of fibrosis in adipocytes.

## 4 Discussion

### 4.1 IFITM1 regulates the proliferation and differentiation of fibroblasts and intervenes in epidural scar hyperplasia

Following laminectomy, up to 80% of patients exhibit substantial fibroblast proliferation and abnormal extracellular matrix metabolism in the surgical site, leading to the formation of epidural scar tissue (35, 36). IFITM1 plays a critical regulatory role in cell proliferation, and its co-expression with Caveolin-1 inhibits ERK activity (12). Reducing Caveolin-1 expression activates the ERK signaling pathway in NIH-3T3 fibroblasts. Since the ERK pathway is essential for fibroblast proliferation, IFITM1 likely suppresses both fibroblast proliferation and differentiation, thereby contributing to the treatment of fibrotic diseases (15, 17, 37–39). This study presents the novel finding that IFITM1 expression in epidural scar tissue decreases following laminectomy. Moreover, IFITM1-KO mice displayed more severe epidural fibrosis compared to wild-type mice after the procedure.

### 4.2 IFITM1 inhibits SMAD3 signaling pathway in fibroblasts

The Smad3 signaling pathway is a well-established mediator in fibrosis. Cytological experiments demonstrated that this pathway is activated by inflammatory factors. Additionally, as fibroblasts proliferate and differentiate, IFITM1 expression declines, suggesting that IFITM1 can inhibit the Smad3 pathway in fibroblasts. These results indicate that IFITM1 may offer therapeutic potential for epidural fibrosis by regulating fibroblast proliferation and differentiation.

### 4.3 IFITM1 interferes with adipocyte fibrosis by regulating the expression of CBR4

In fibrotic diseases, the proliferation and differentiation of fibroblasts into myofibroblasts are crucial, but adipocytes also play a pivotal role in this process (27, 40, 41). Adipocytes can transform into myofibroblasts, thereby promoting the progression of fibrosis. Studies on skin repair have identified adipocyte myofibroblast transformation (AMT) as a significant mechanism (27, 42). CBR4, a key protein in fatty acid synthesis (19, 20), facilitates the process of adipogenesis, where adipocytes absorb fatty acids and glycerol to synthesize triacylglycerol. When fat storage reaches a certain threshold, adipocytes can divide to form new adipocytes, enabling further fat storage (43, 44). In the present study, CBR4 was identified as a protein interacting with IFITM1 through immunoprecipitation. It is hypothesized that IFITM1 regulates adipocyte fibrosis by modulating CBR4 expression, thus contributing to the treatment of epidural fibrosis. Furthermore, adipose tissue was observed in epidural scar tissue following laminectomy, with IFITM1-KO mice showing significantly reduced adipose tissue in the epidural scars. Under inflammatory conditions, adipocytes can differentiate into fibroblasts and myofibroblasts, accompanied by reduced expression of both IFITM1 and CBR4. Moreover, inhibiting IFITM1 expression in adipocytes exacerbates adipocyte fibrosis, while IFITM1 overexpression in these cells inhibits fibrosis, with CBR4 expression correlating with IFITM1 levels. These results suggest that IFITM1 regulates adipocyte fibrosis through CBR4, alleviating epidural fibrosis. Future studies are essential to explore the mechanisms by which the IFITM1/CBR4 pathway regulates adipocyte fibrosis and to further validate IFITM1 as a potential therapeutic target in fibrotic diseases.

This study indicates that IFITM1 inhibits the Smad3 signaling pathway in fibroblasts and CBR4 expression in adipocytes, suppressing fibroblast proliferation and adipocyte fibrosis, which ultimately mitigates epidural fibrosis. However, the study is limited to animal and cell models, with a lack of clinical data. The infrequency of revision surgeries, which are typically only performed on patients with severe epidural fibrosis, restricts access to clinical epidural scar tissue. This limitation may affect the accuracy of our findings. Additionally, the process by which adipocyte fibrosis occurs, whether as a direct transformation or through differentiation into mesenchymal stem cells before being induced into myofibroblasts, remains unclear. It is also unknown whether adipocytes undergoing lipolysis exhibit differentiation abilities similar to mesenchymal stem cells. Therefore, further investigation is needed to fully elucidate the mechanisms through which IFITM1 regulates fibroblasts and adipocytes in the treatment of epidural fibrosis.

## 5 Conclusion

IFITM1 may regulate fibroblast proliferation and differentiation through the Smad3 signaling pathway and influence adipocyte fibrosis by modulating CBR4 protein expression, thereby achieving therapeutic effects on epidural scar hyperplasia following laminectomy.

## Data Availability

The original contributions presented in the study are included in the article/**Supplementary Material**. Further inquiries can be directed to the corresponding author.
