# Supplementary material for: Mechanism of IFITM1 regulating epidural scar hyperplasia after laminectomy through SMAD3/CBR4 pathway
Source: Front Immunol. 2025 Sep 3;16:1628970. doi: 10.3389/fimmu.2025.1628970 (PMC12440877; doi:10.3389/fimmu.2025.1628970)
Supplement: Supplementary file 2 [file DataSheet1.pdf]

figure1-D

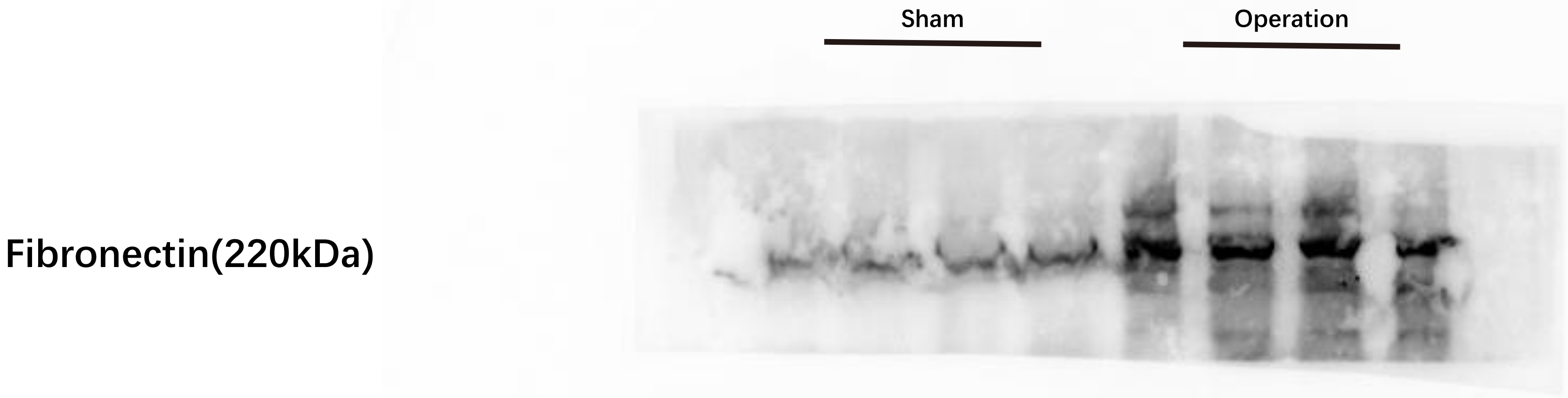

Fibronectin(220kDa)

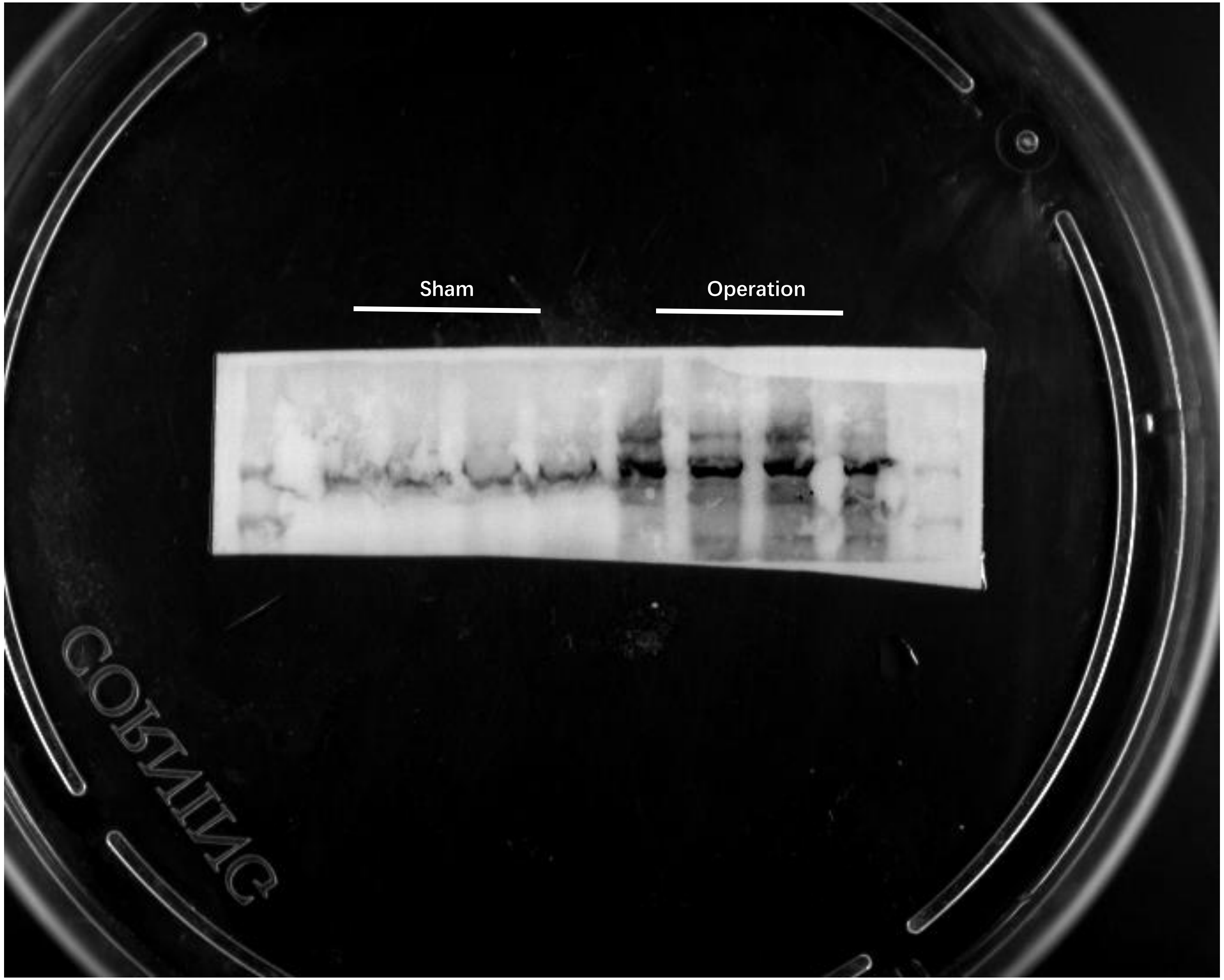

α-SMA(42kDa)

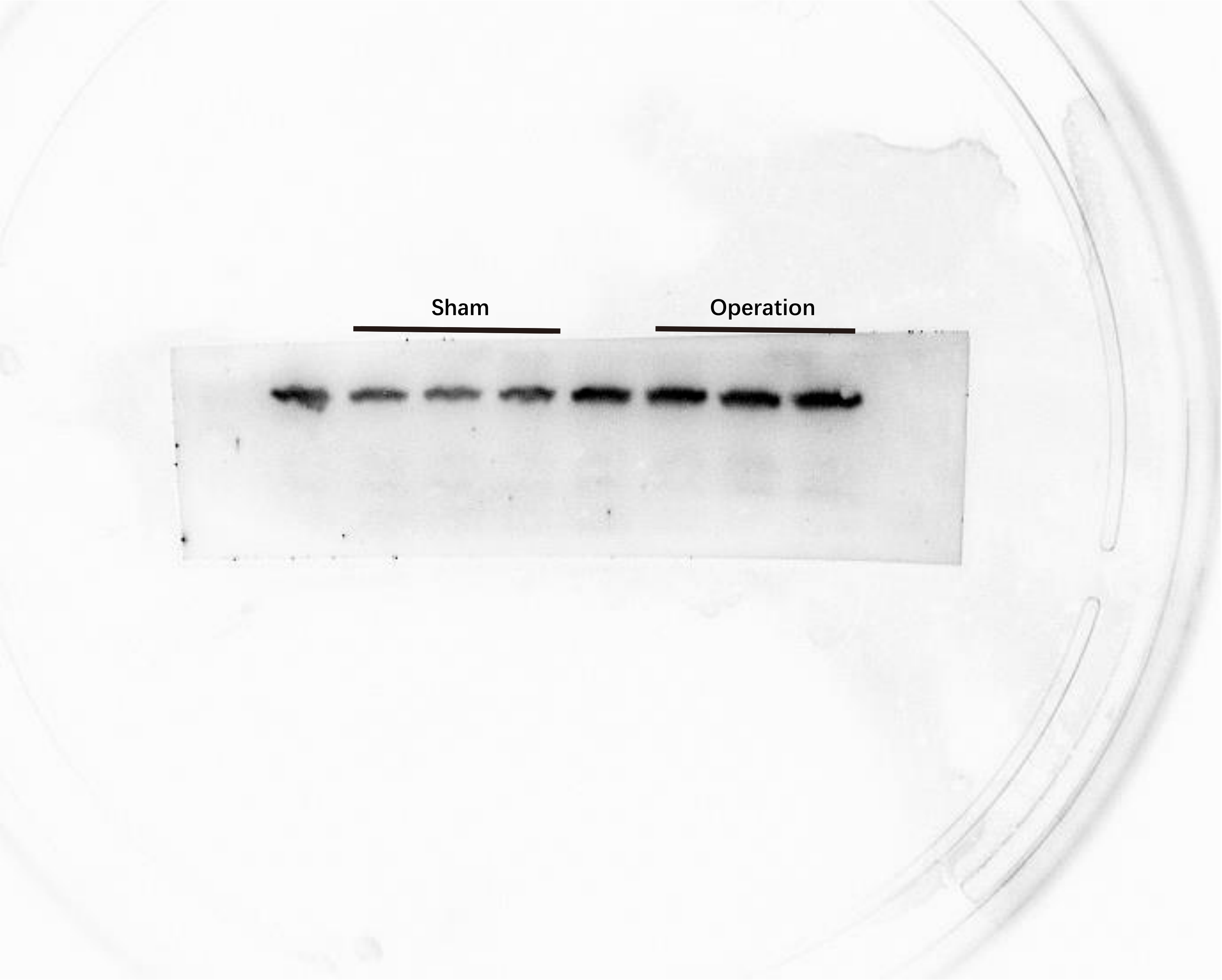

α-SMA(42kDa)

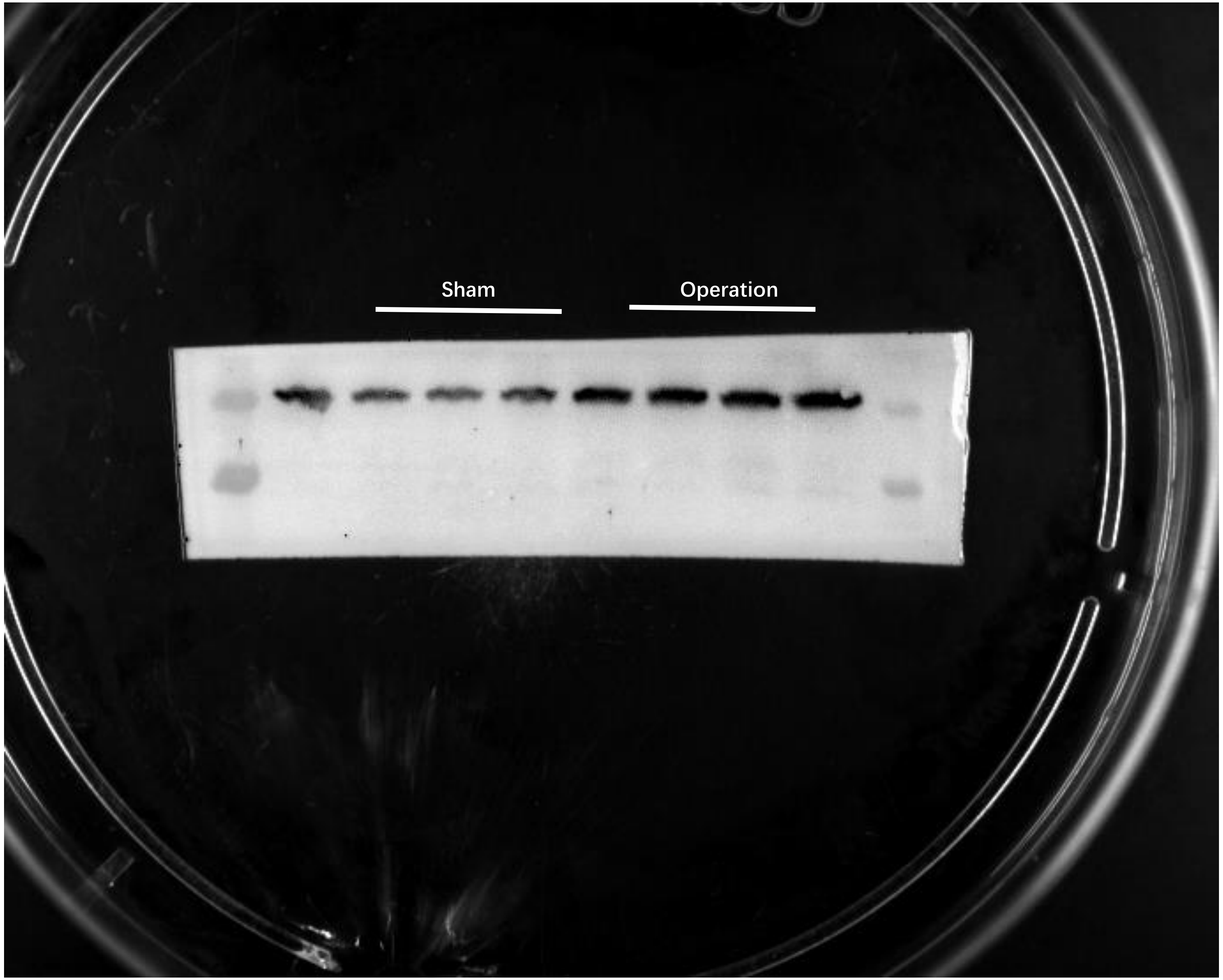

TGF-β1(55kDa)

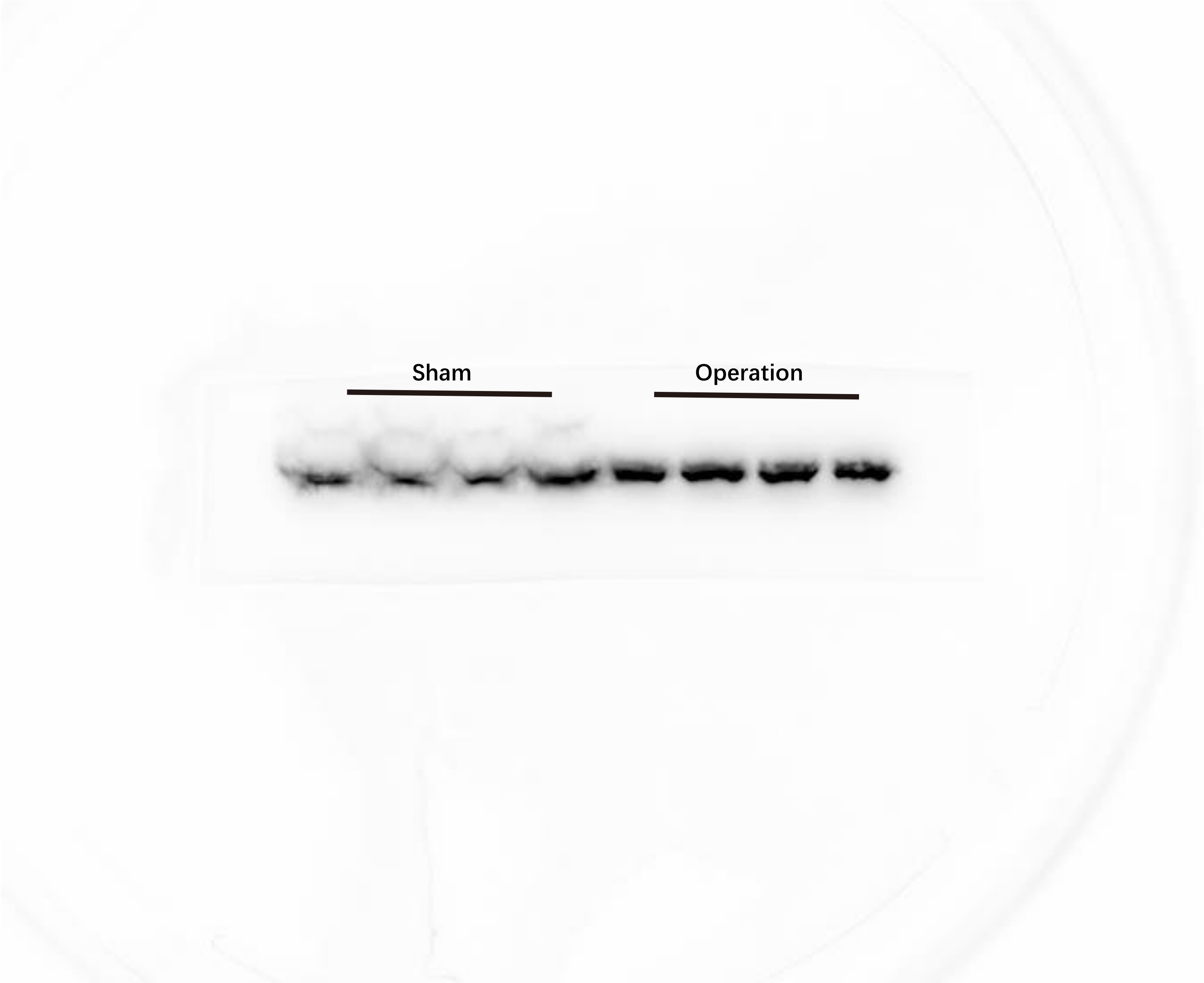

TGF-β1(55kDa)

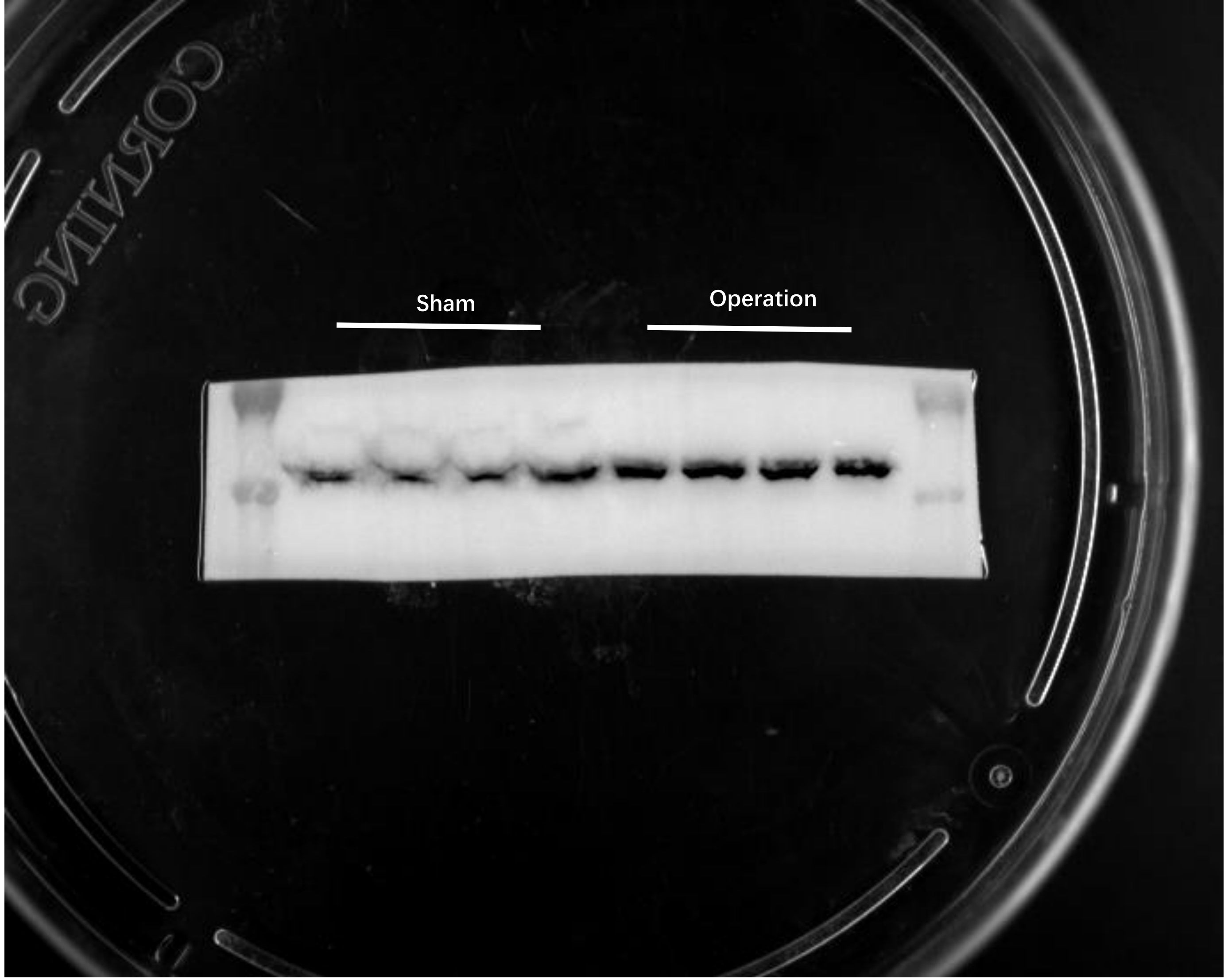

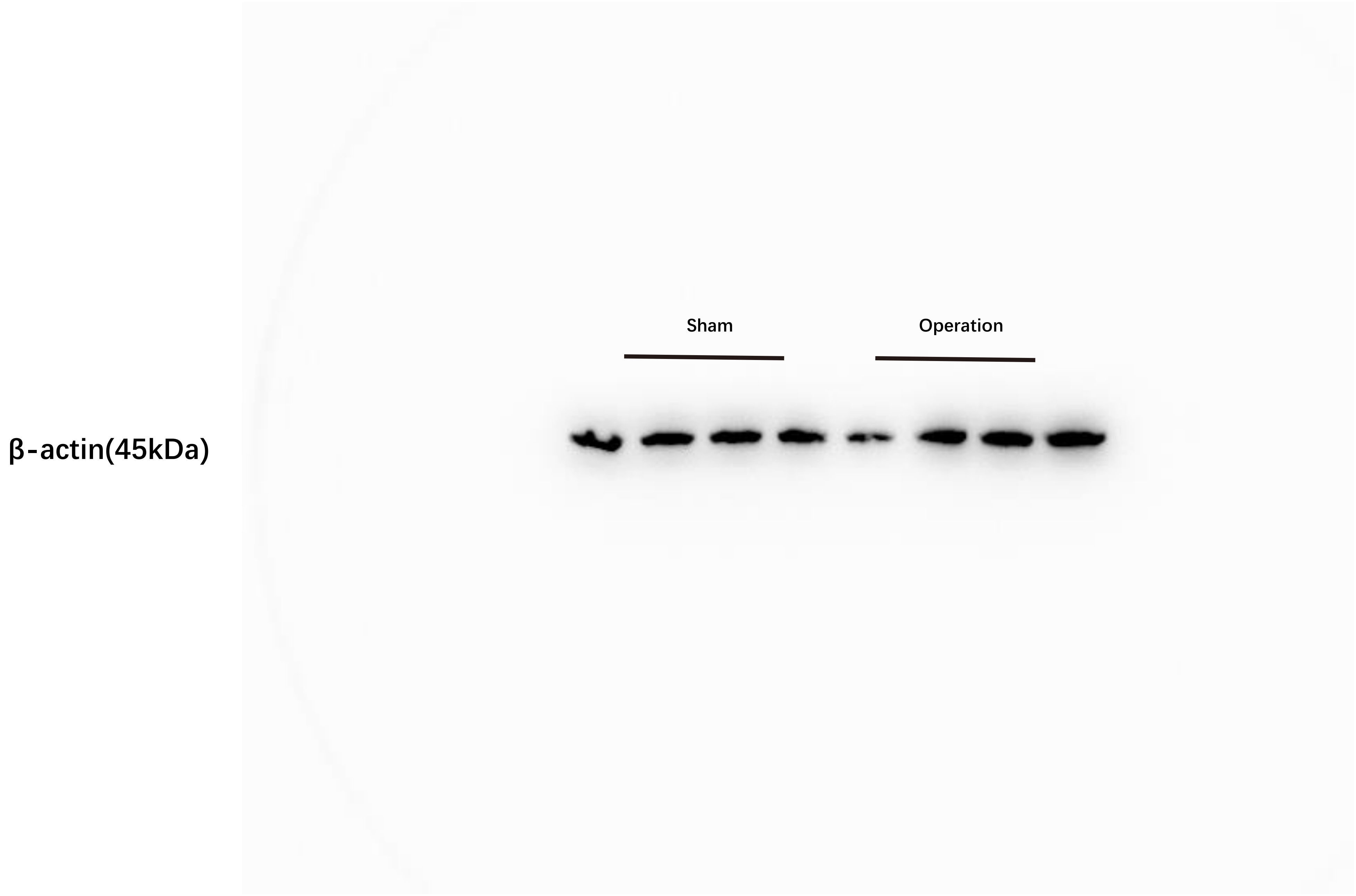
Sham
Operation
β-actin(45kDa)

β-actin(45kDa)

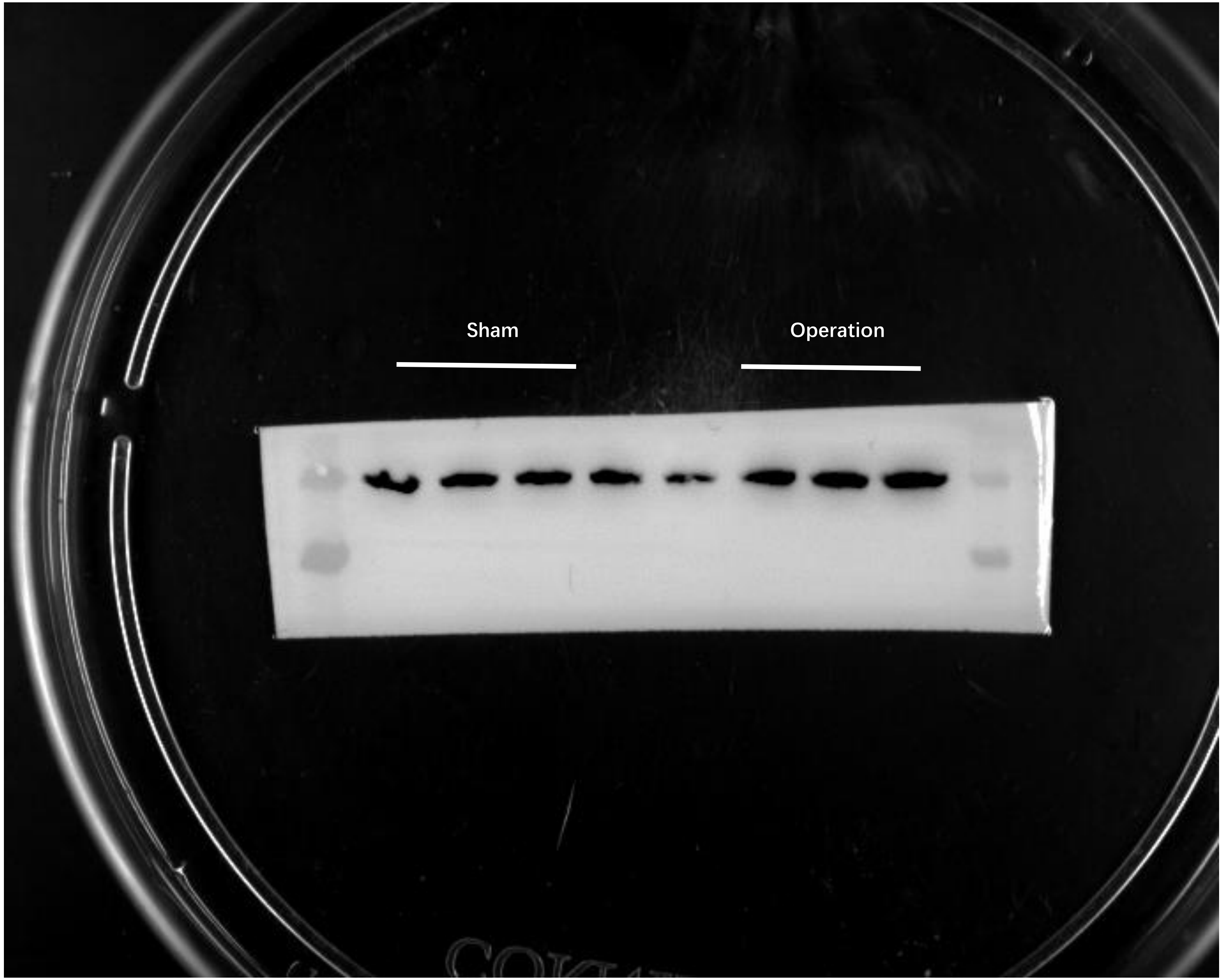

figure1-E

**IFITM1（25-35KDa）**

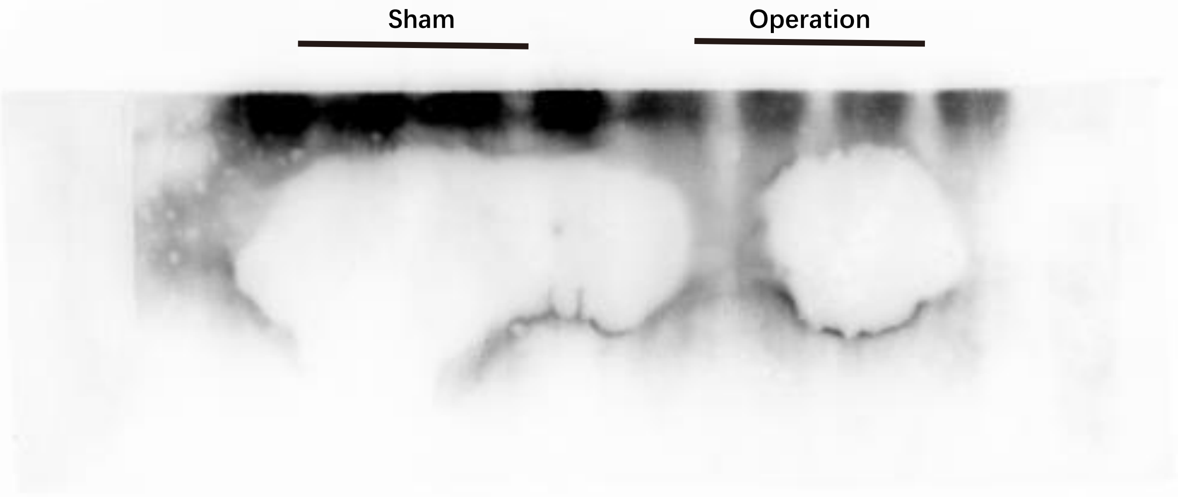

IFITM1（25-35KDa）

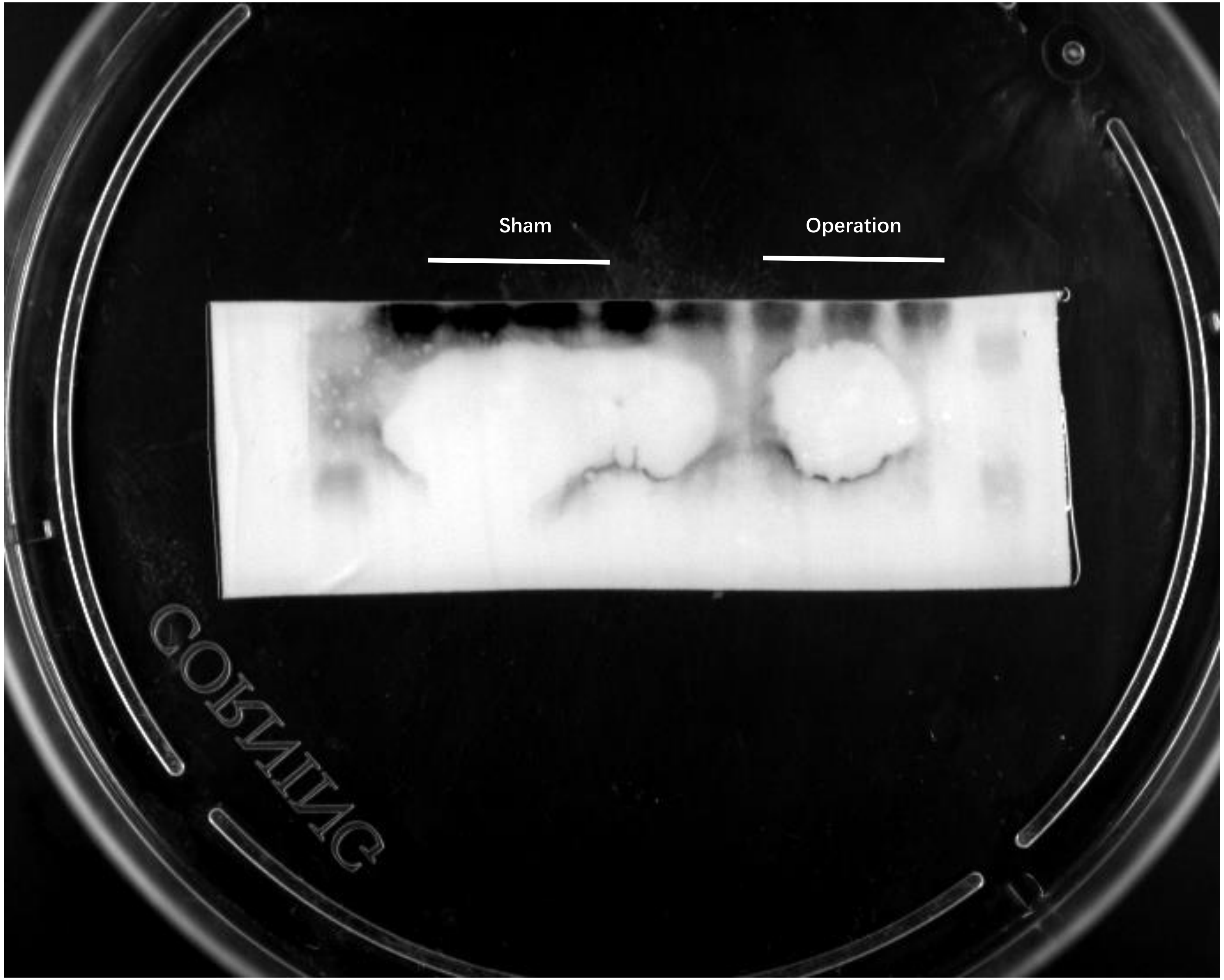

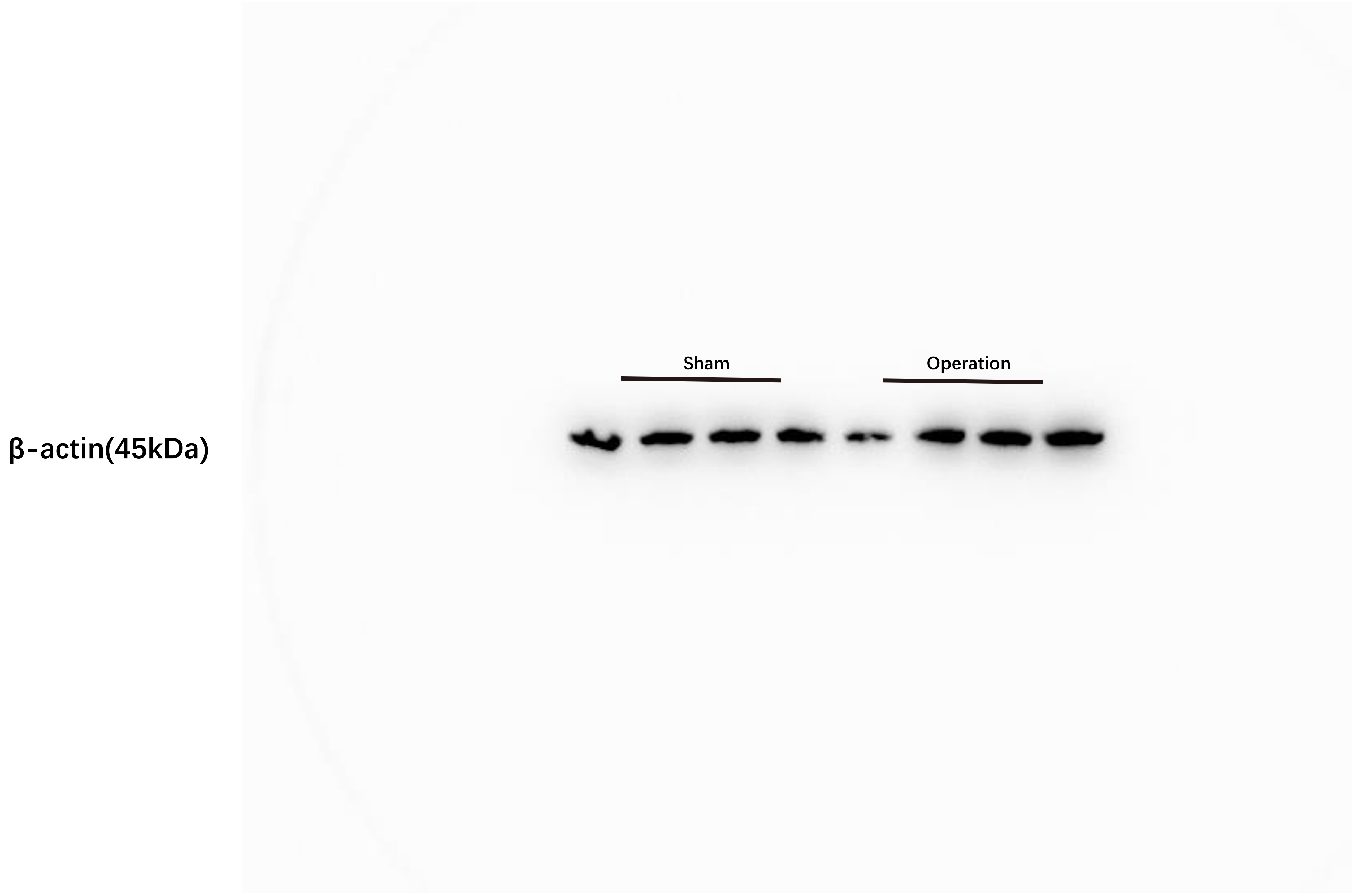
Sham
Operation
β-actin(45kDa)

β-actin(45kDa)

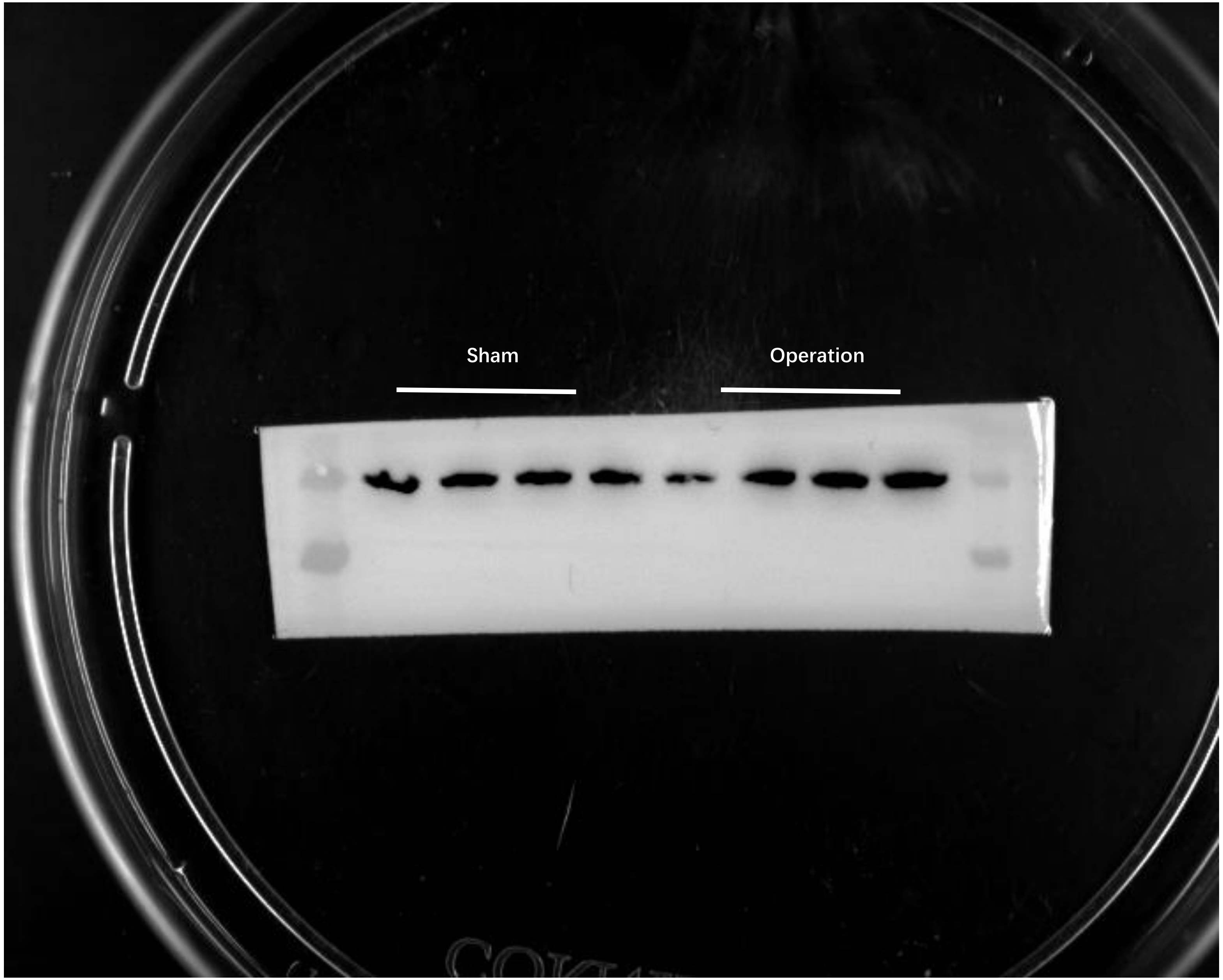

figure2-D

**Fibronectin(220kDa)**

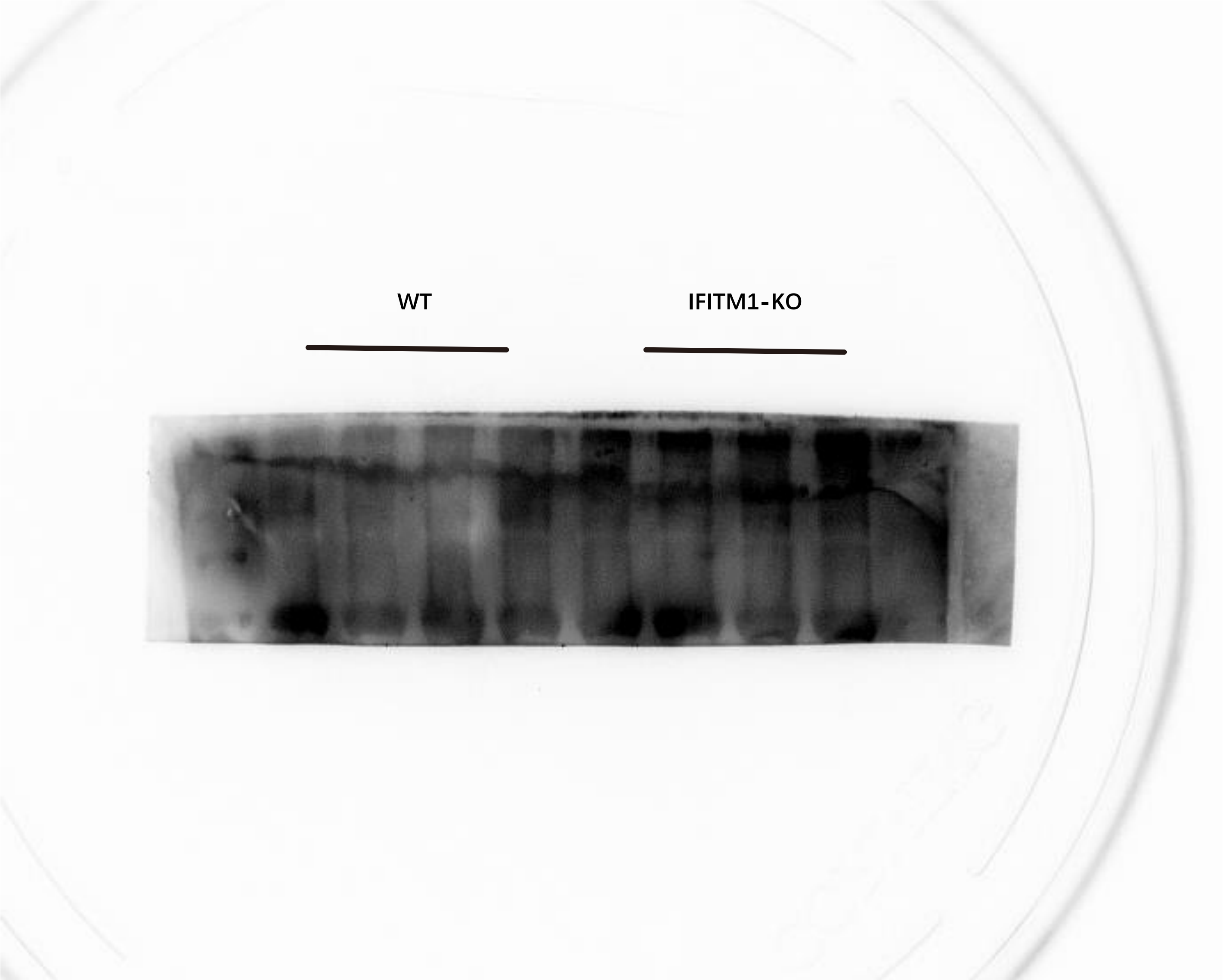

Fibronectin(220kDa)

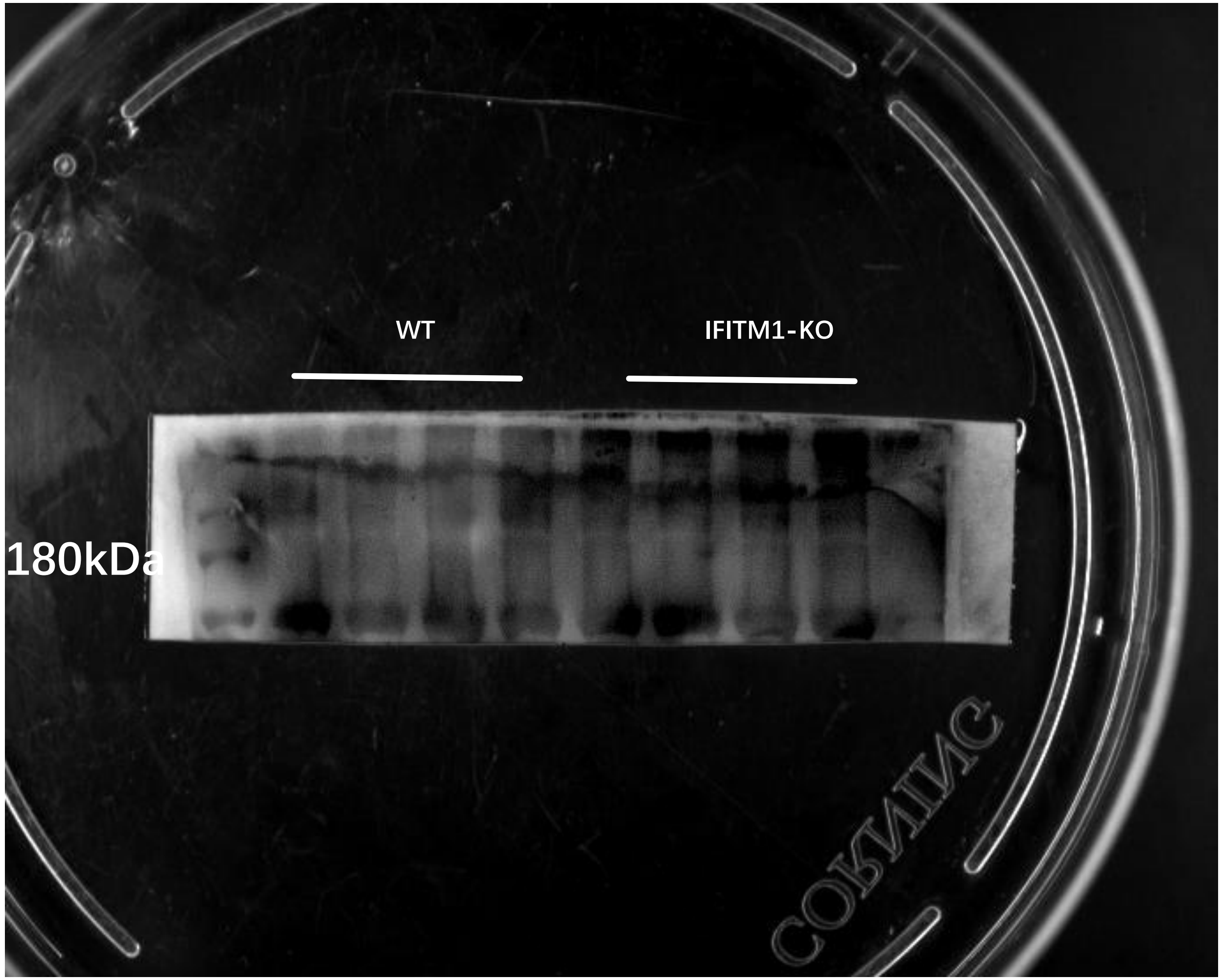

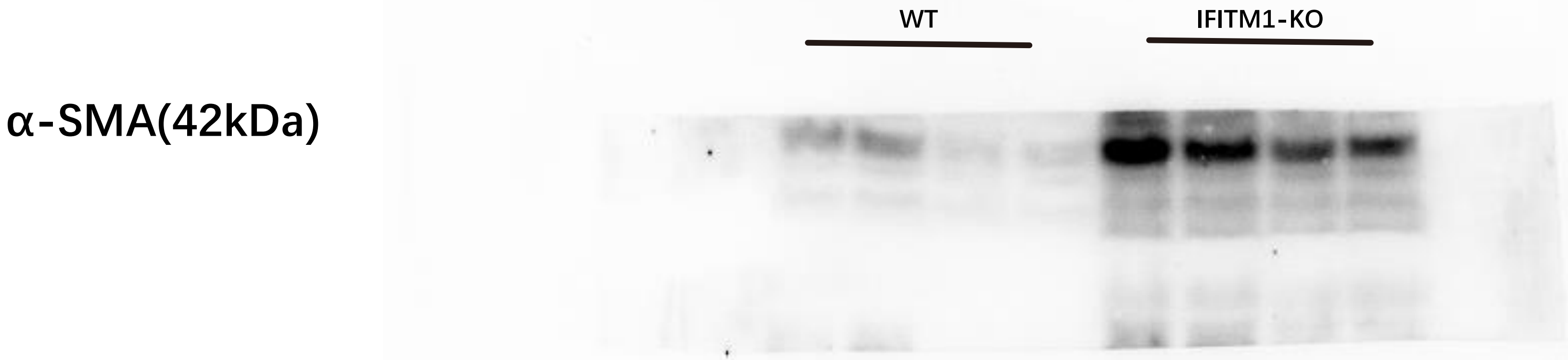
WT
IFITM1-KO
α-SMA(42kDa)

α-SMA(42kDa)

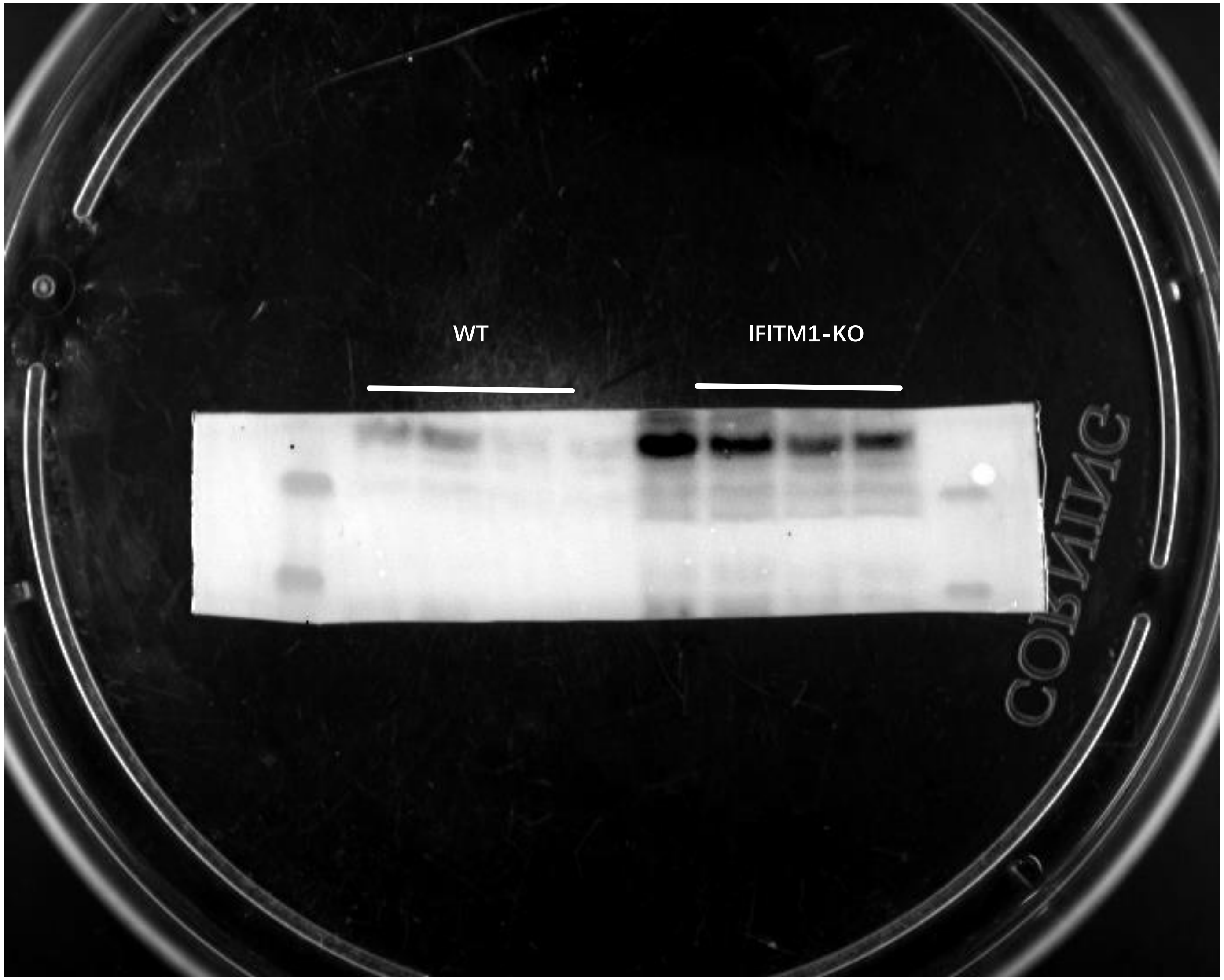

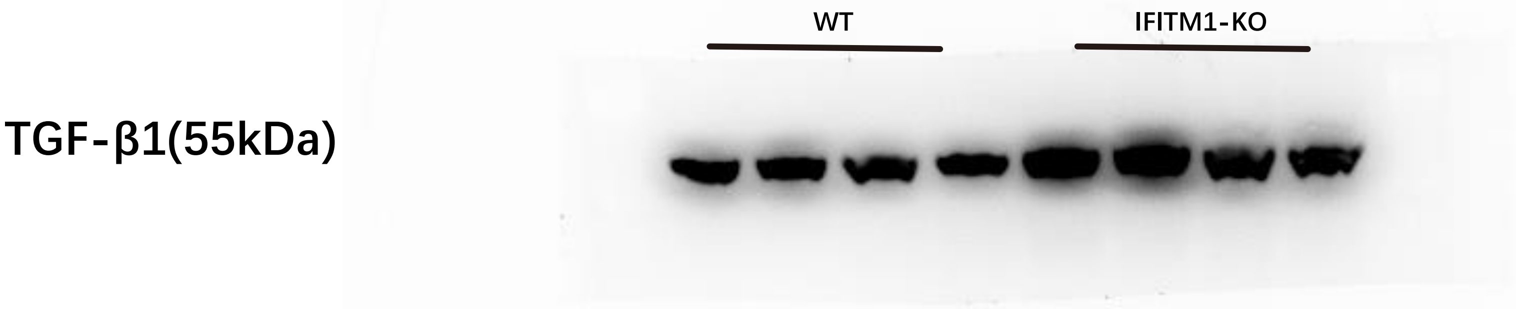
WT
IFITM1-KO
TGF-β1(55kDa)

TGF-β1(55kDa)

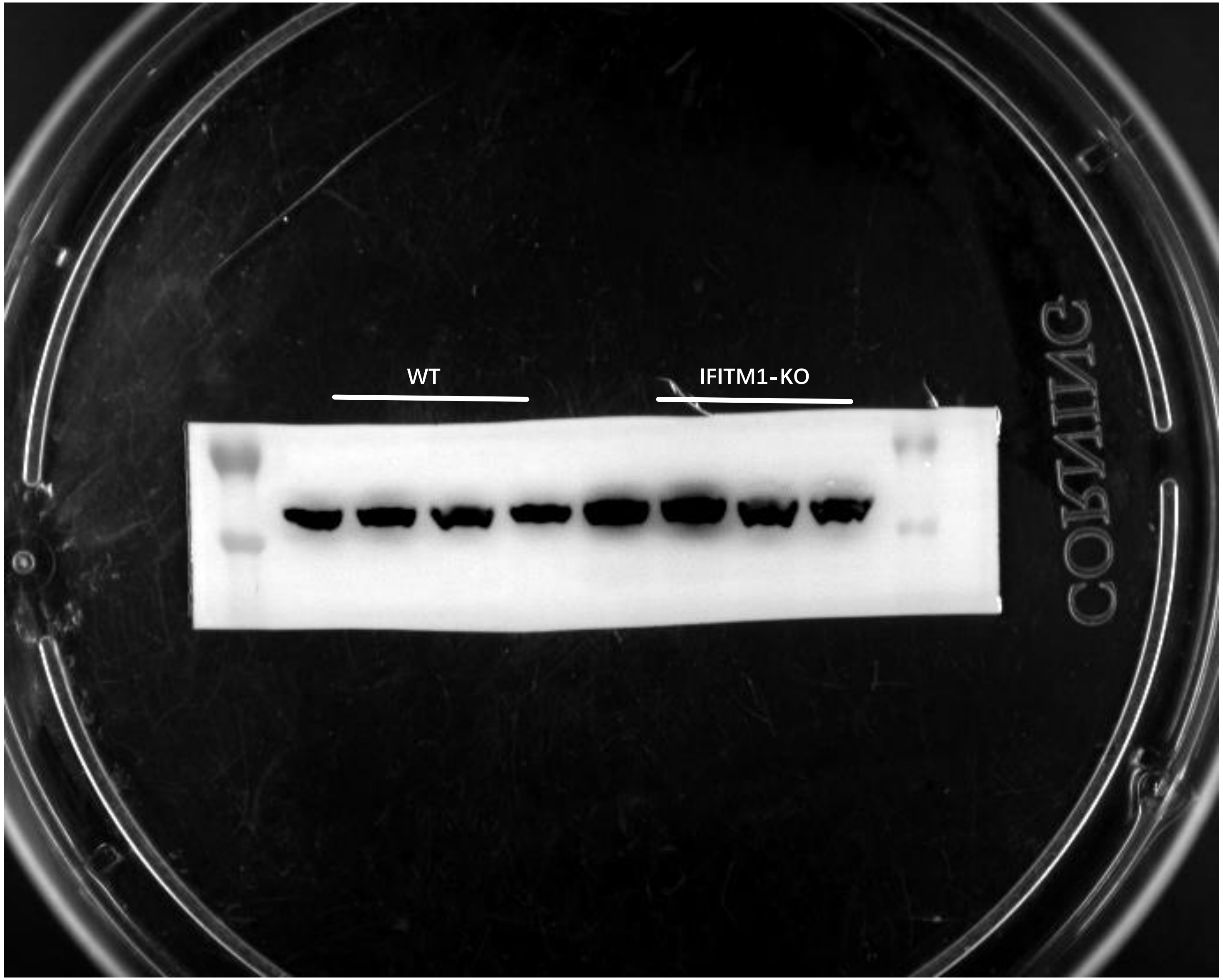

β-actin(45kDa)

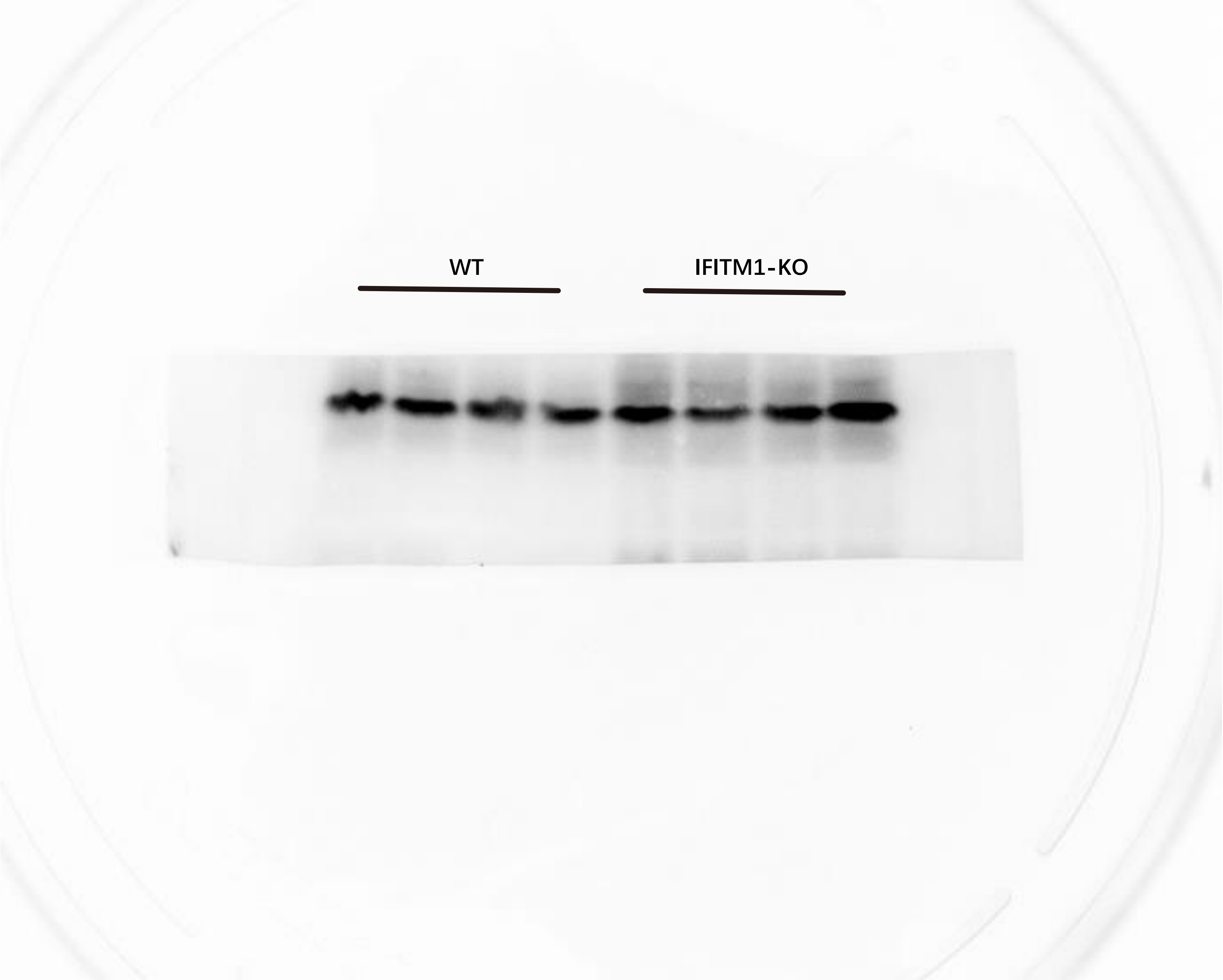

β-actin(45kDa)

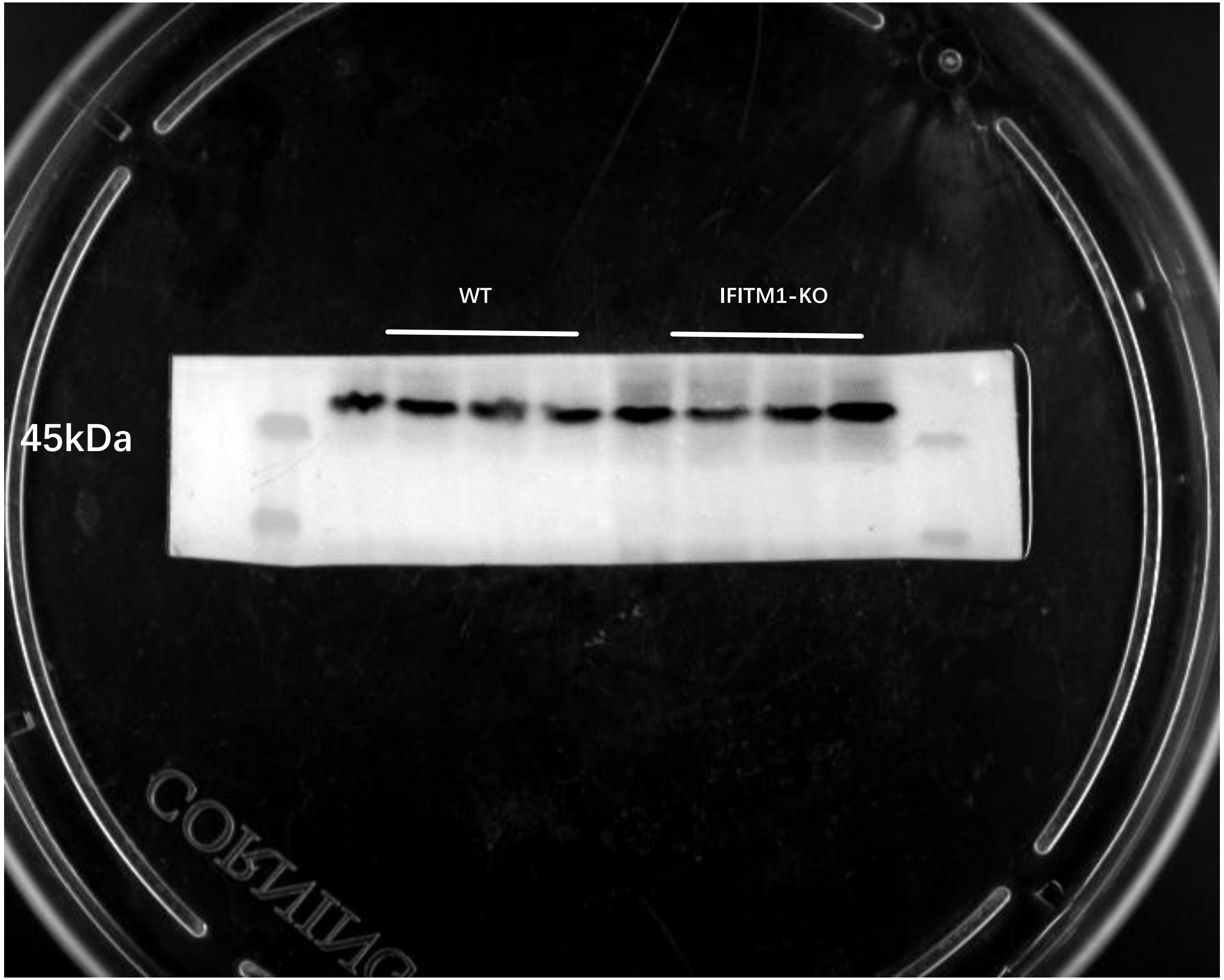

figure2-E

**IFITM1（25-35KDa）**

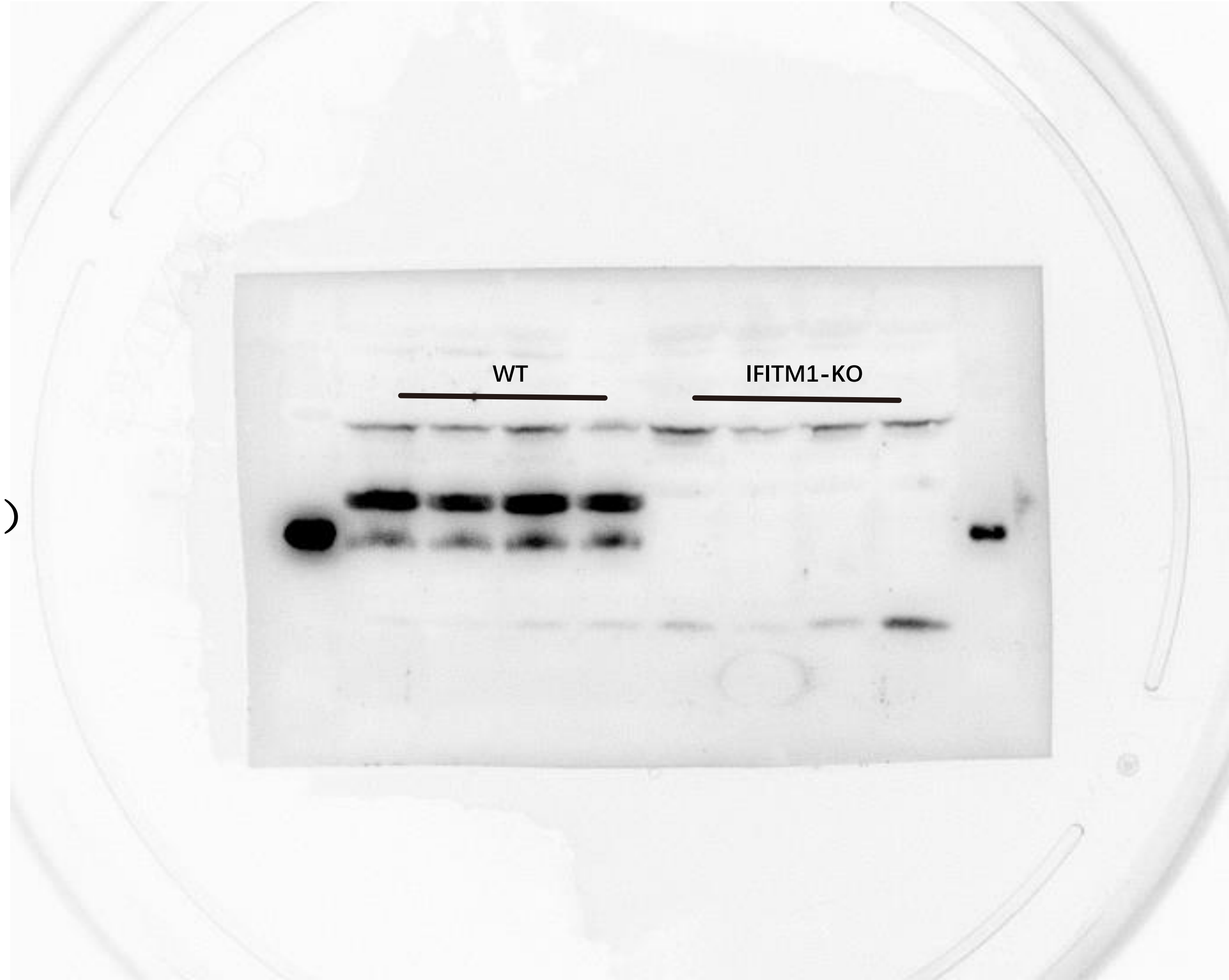

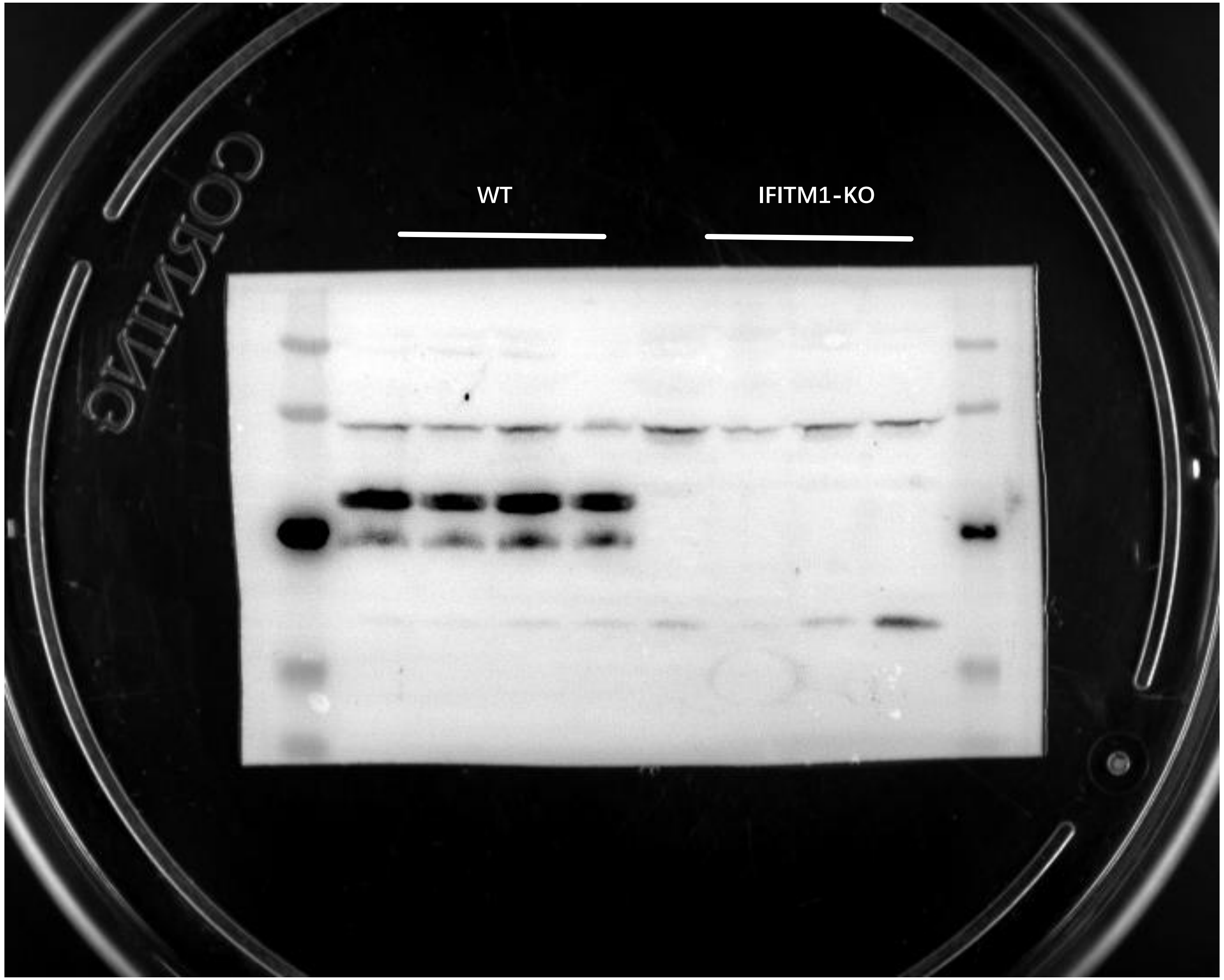


IFITM1（25-35KDa）

GAPDH(35kDa)

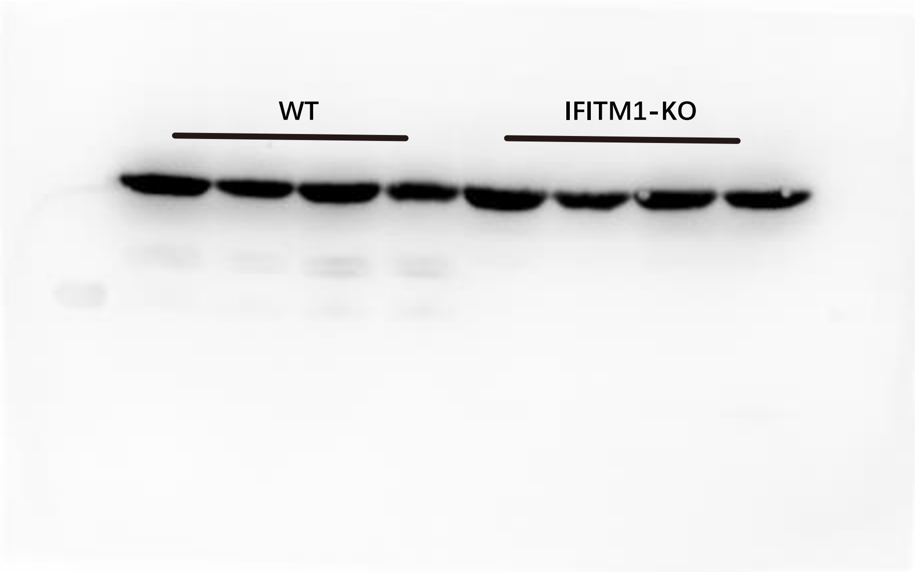

GAPDH(35kDa)

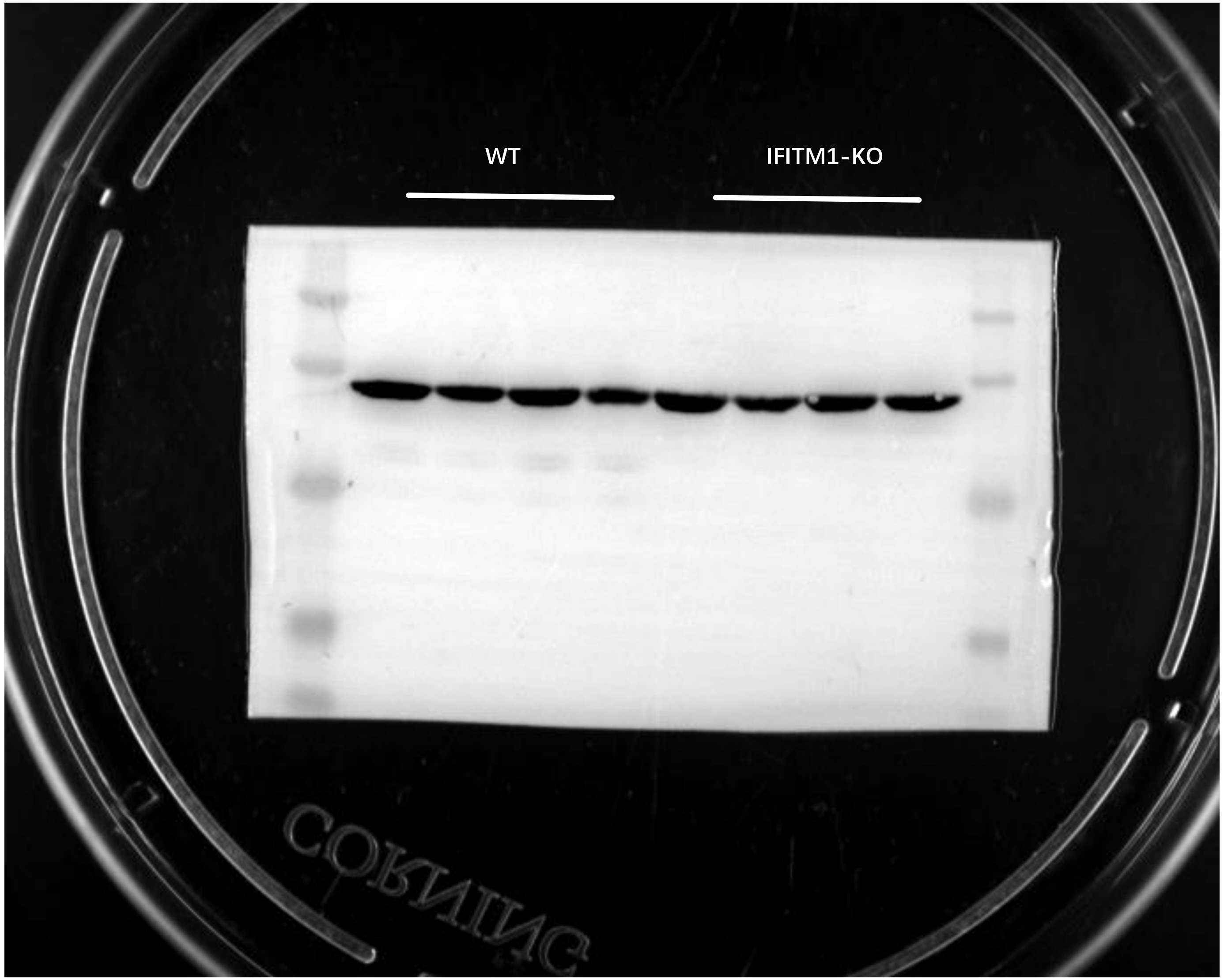

figure3-A-1

Fibronectin(220kDa)

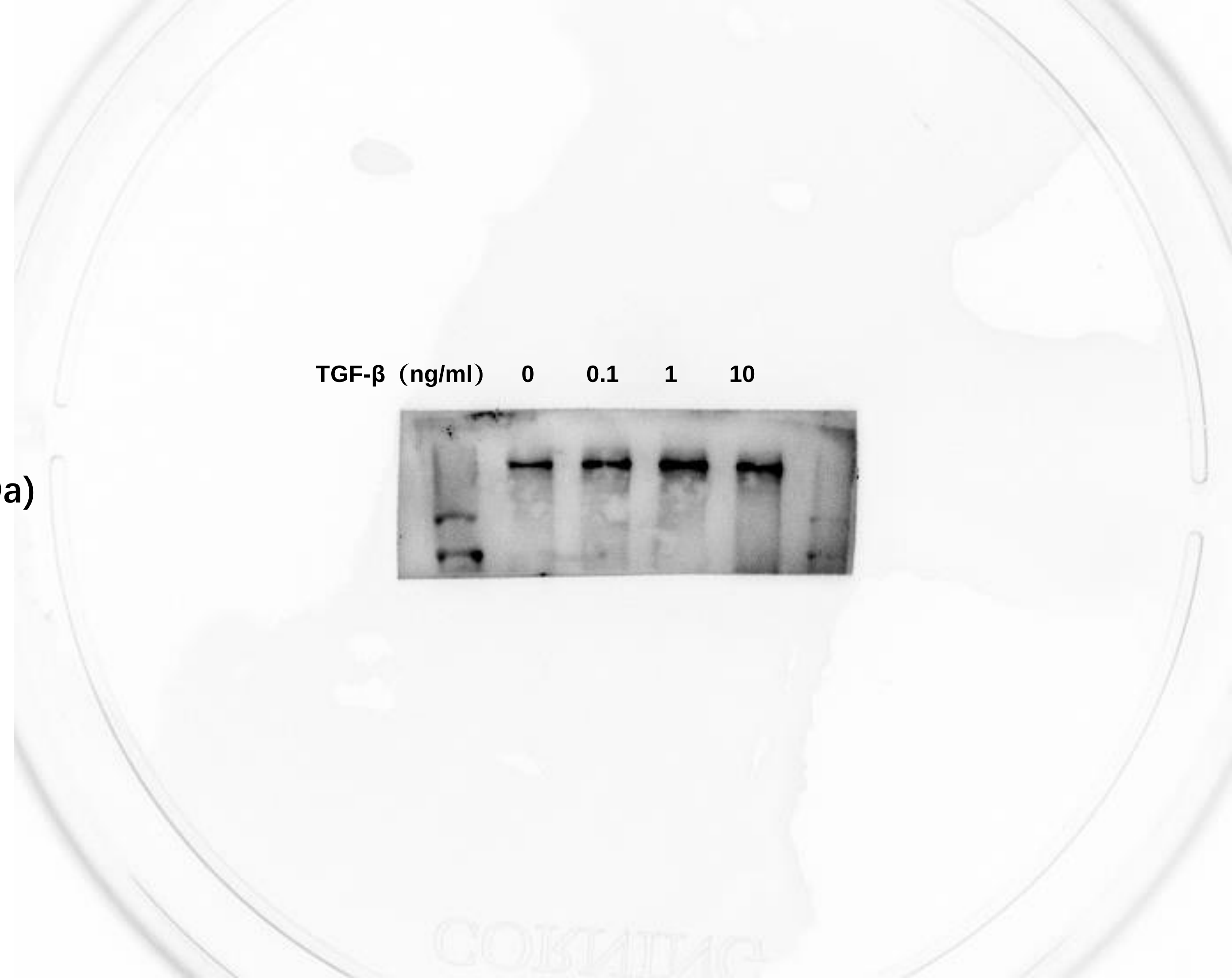

Fibronectin(220kDa)

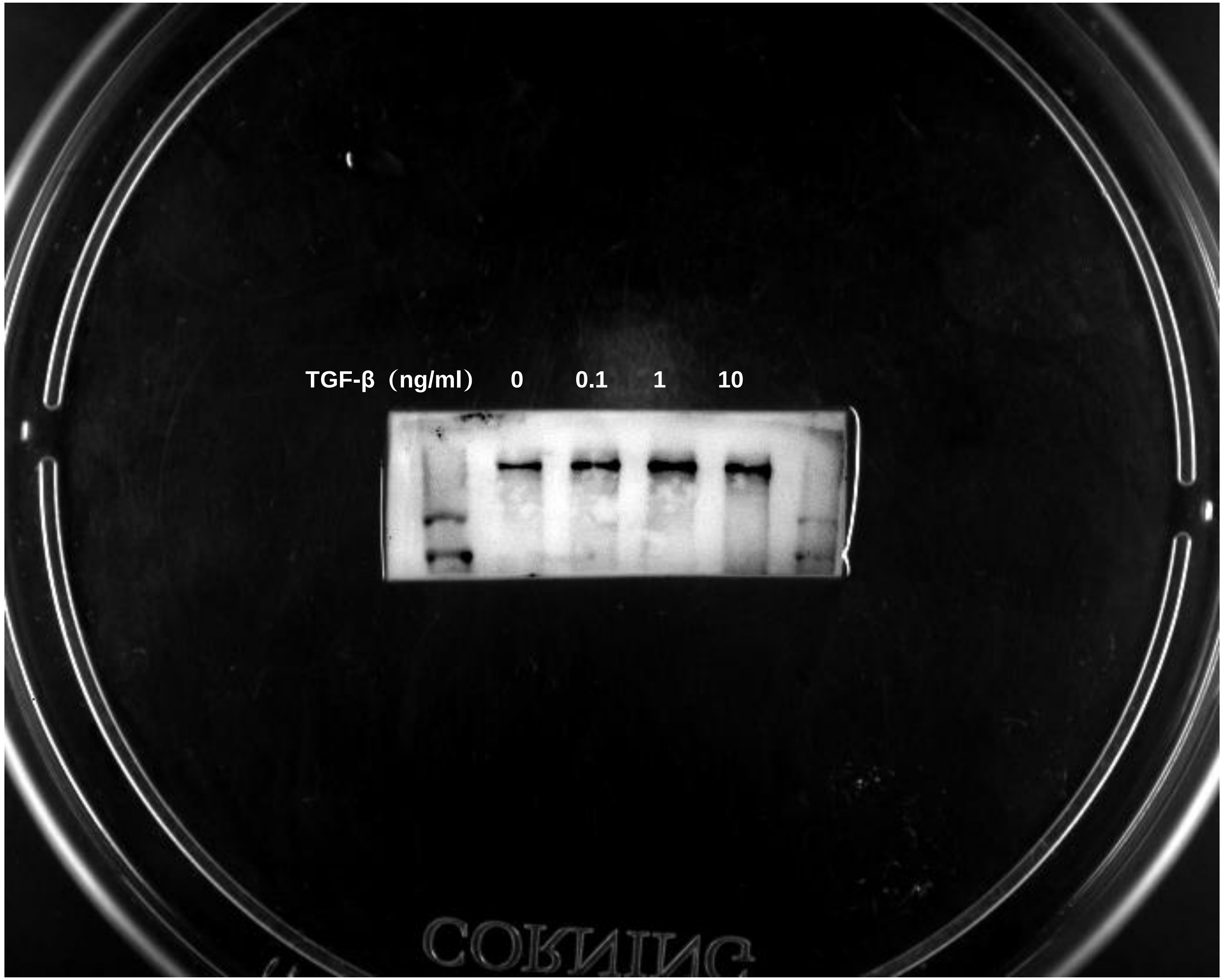

IFITM1（25-35KDa）

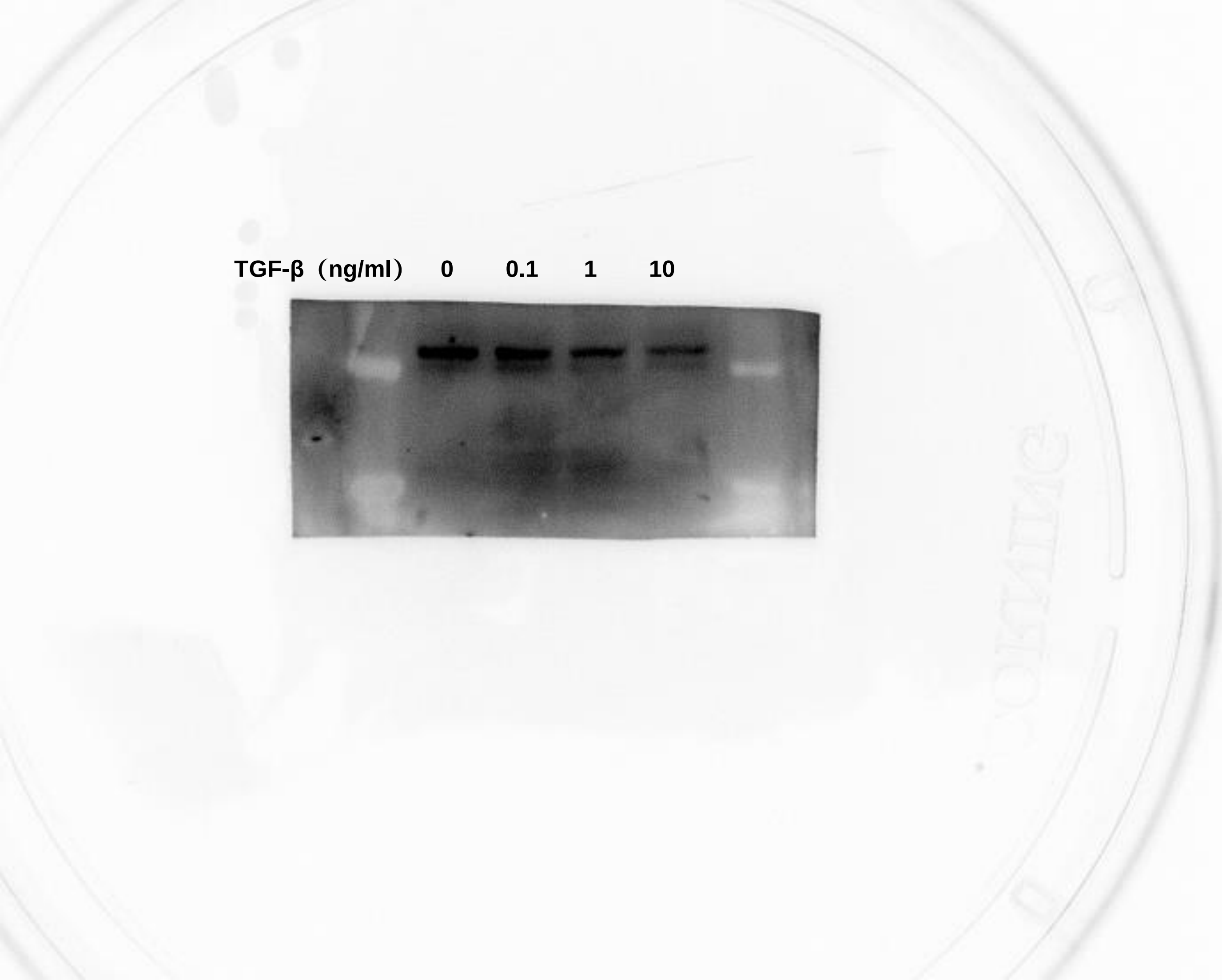

IFITM1（25-35KDa）

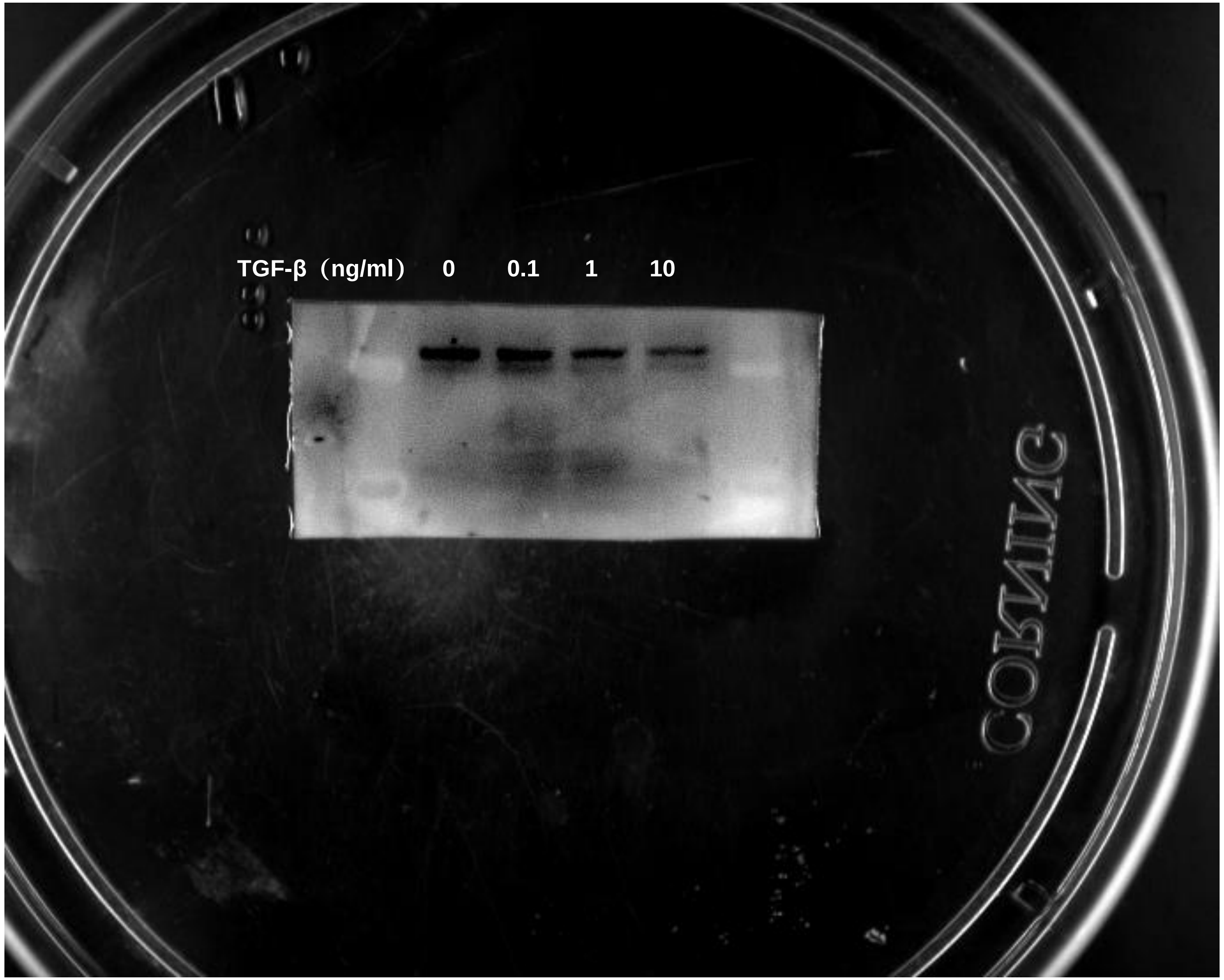

β-actin(45kDa)

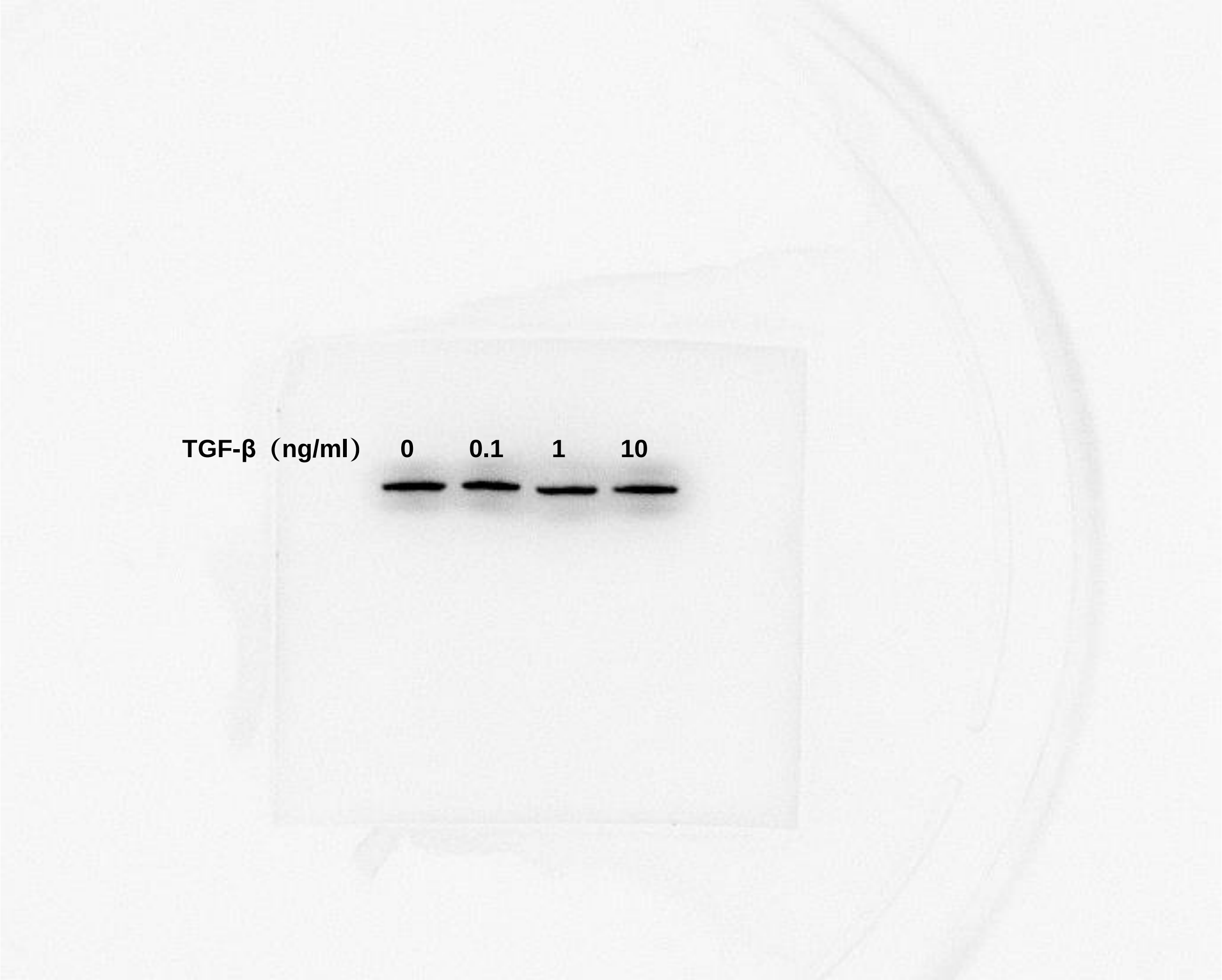

β-actin(45kDa)

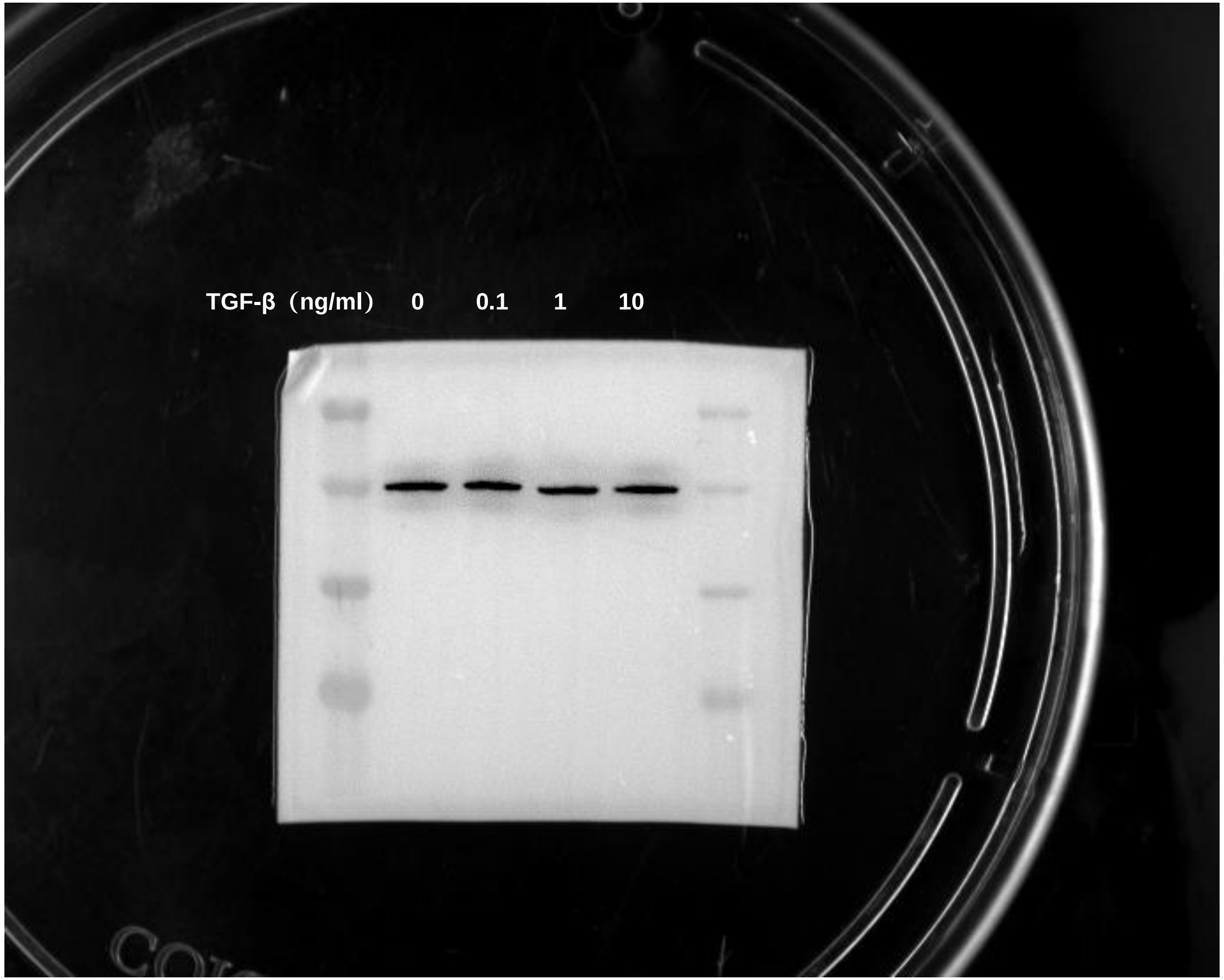

figure3-A-2

Fibronectin(220kDa)

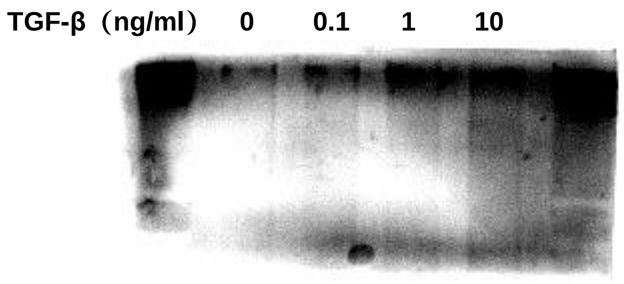

Fibronectin(220kDa)

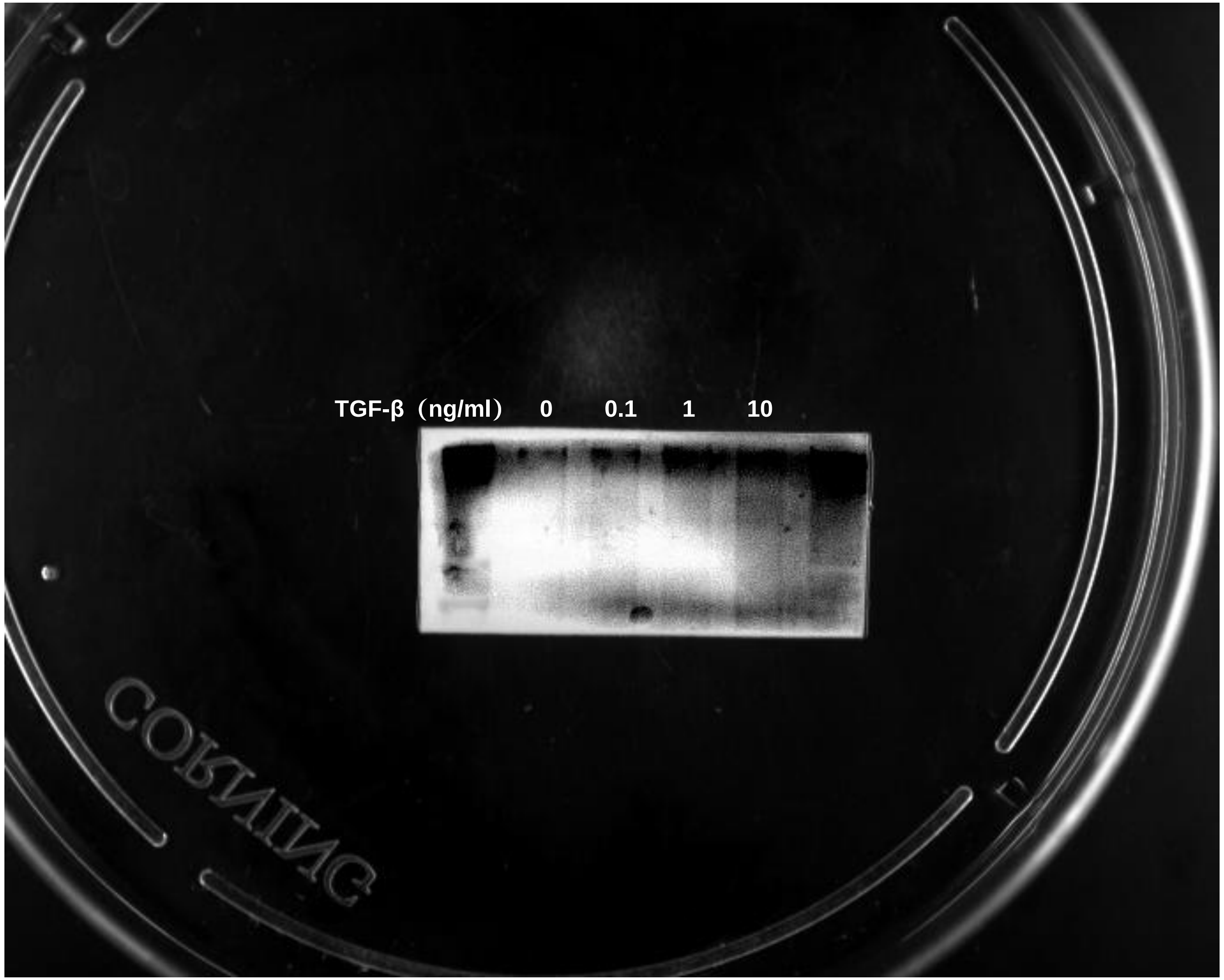

IFITM1（25-35KDa）

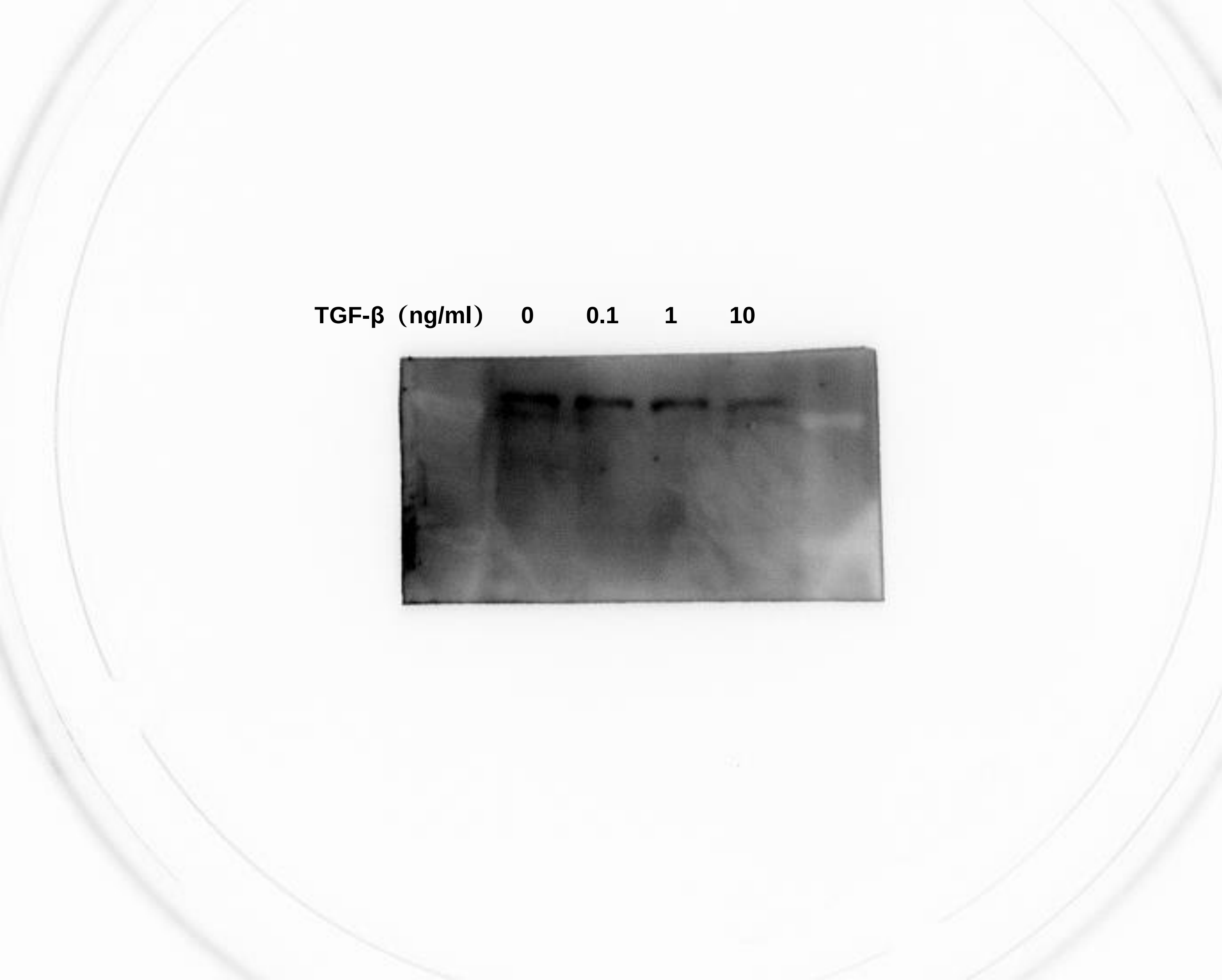

IFITM1（25-35KDa）

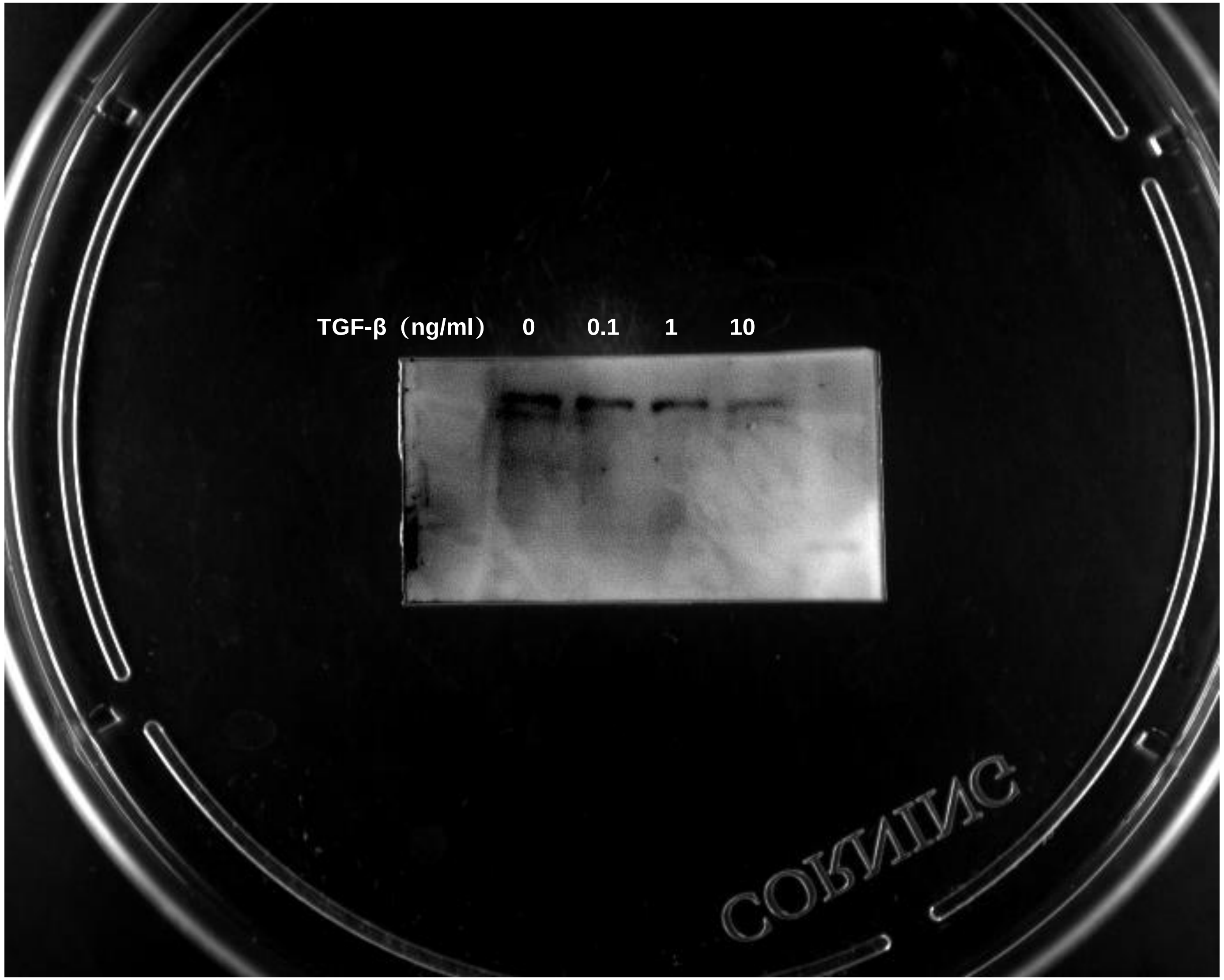

β-actin(45kDa)

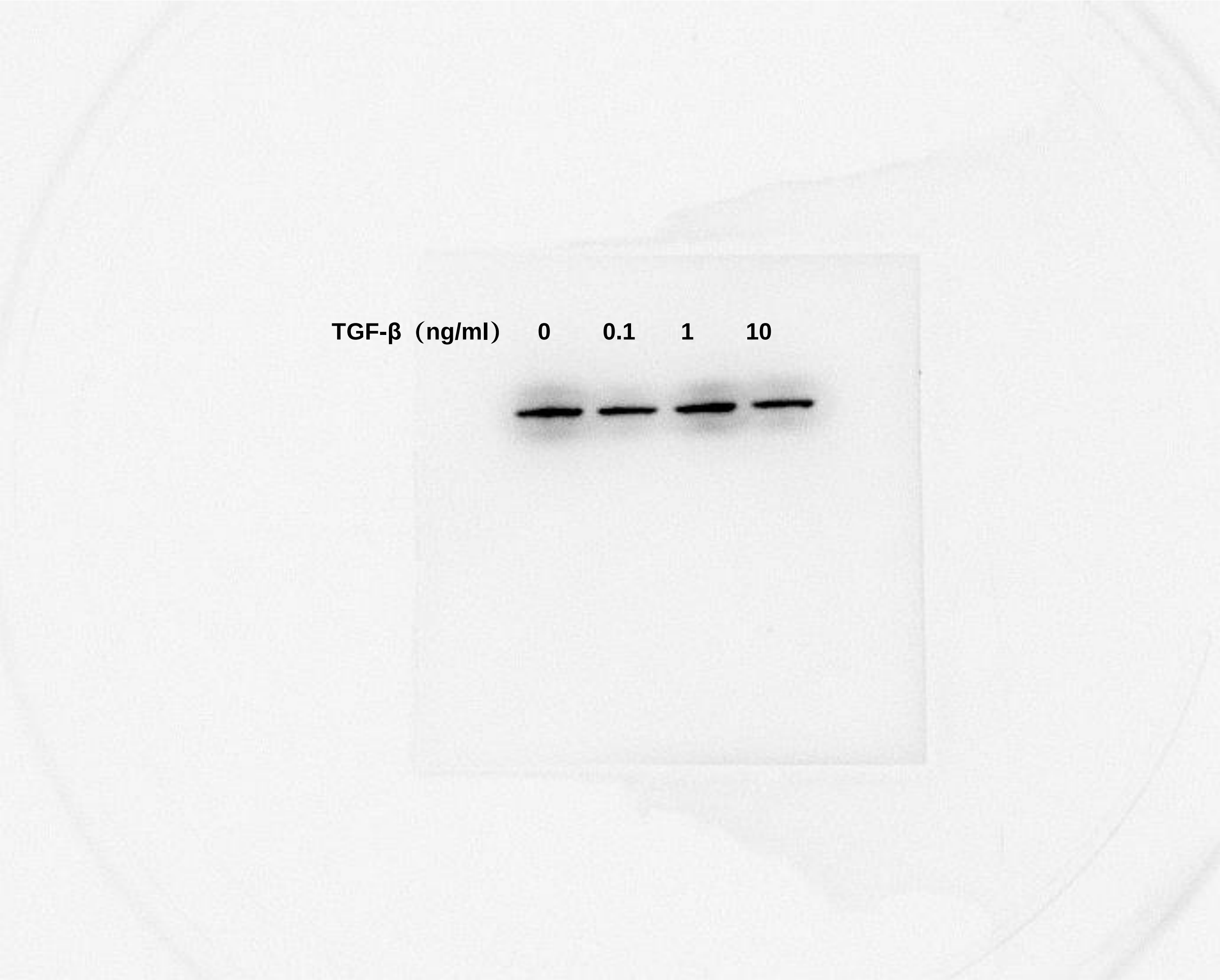

β-actin(45kDa)

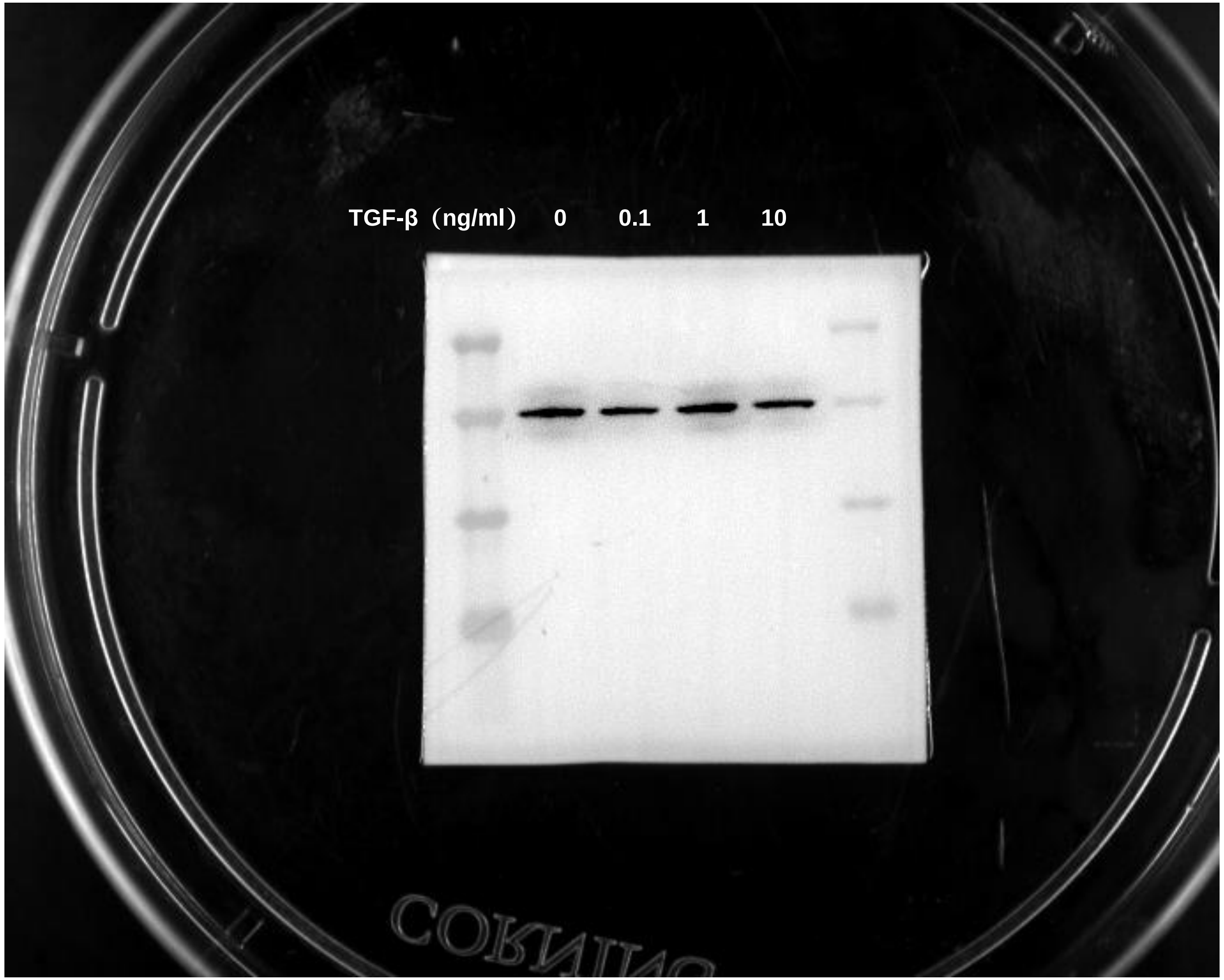

figure3-A-3

Fibronectin(220kDa)

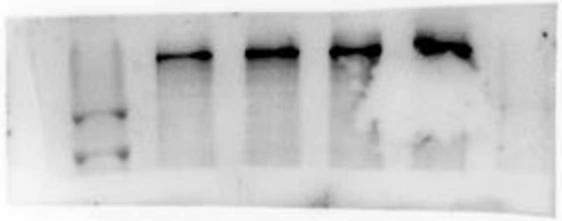

Fibronectin(220kDa)

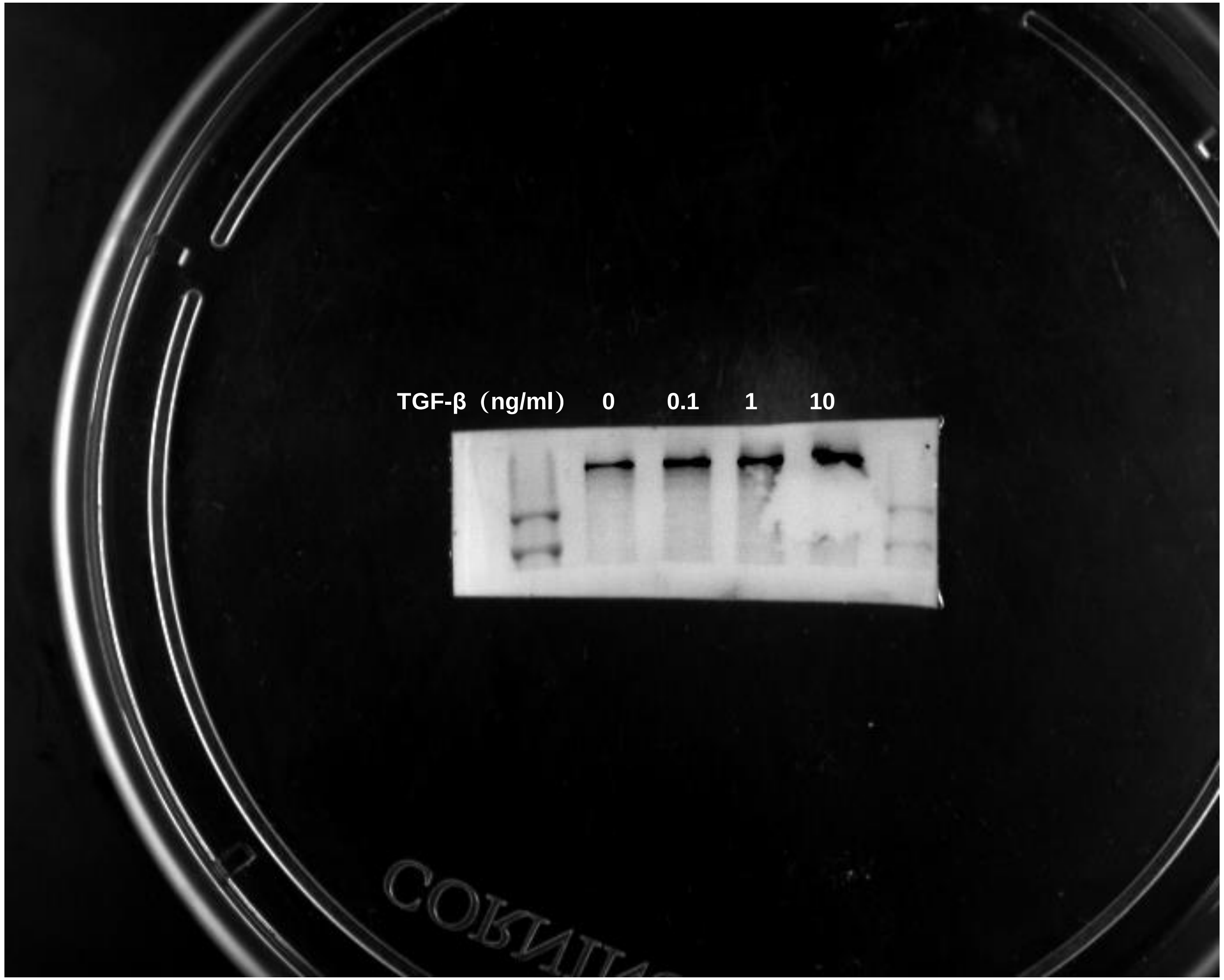

TGF-β（ng/ml） 0 0.1 1 10

IFITM1（25-35KDa）

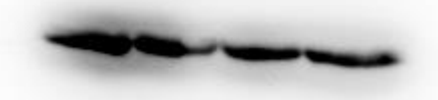

IFITM1（25-35KDa）

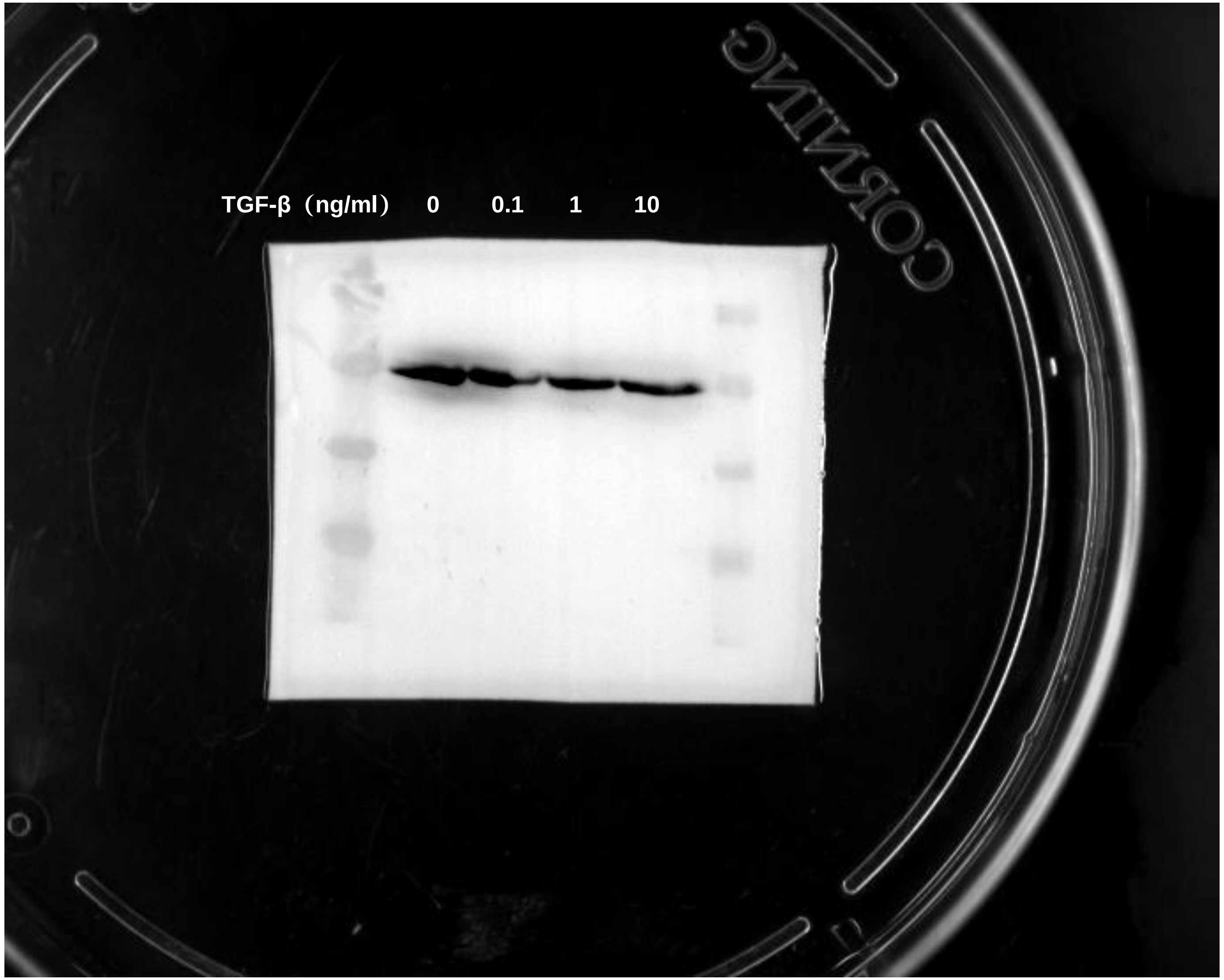

β-actin(45kDa)

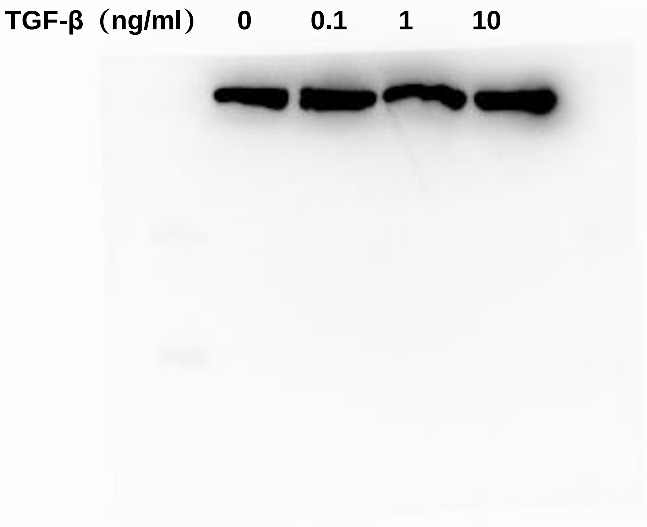

β-actin(45kDa)

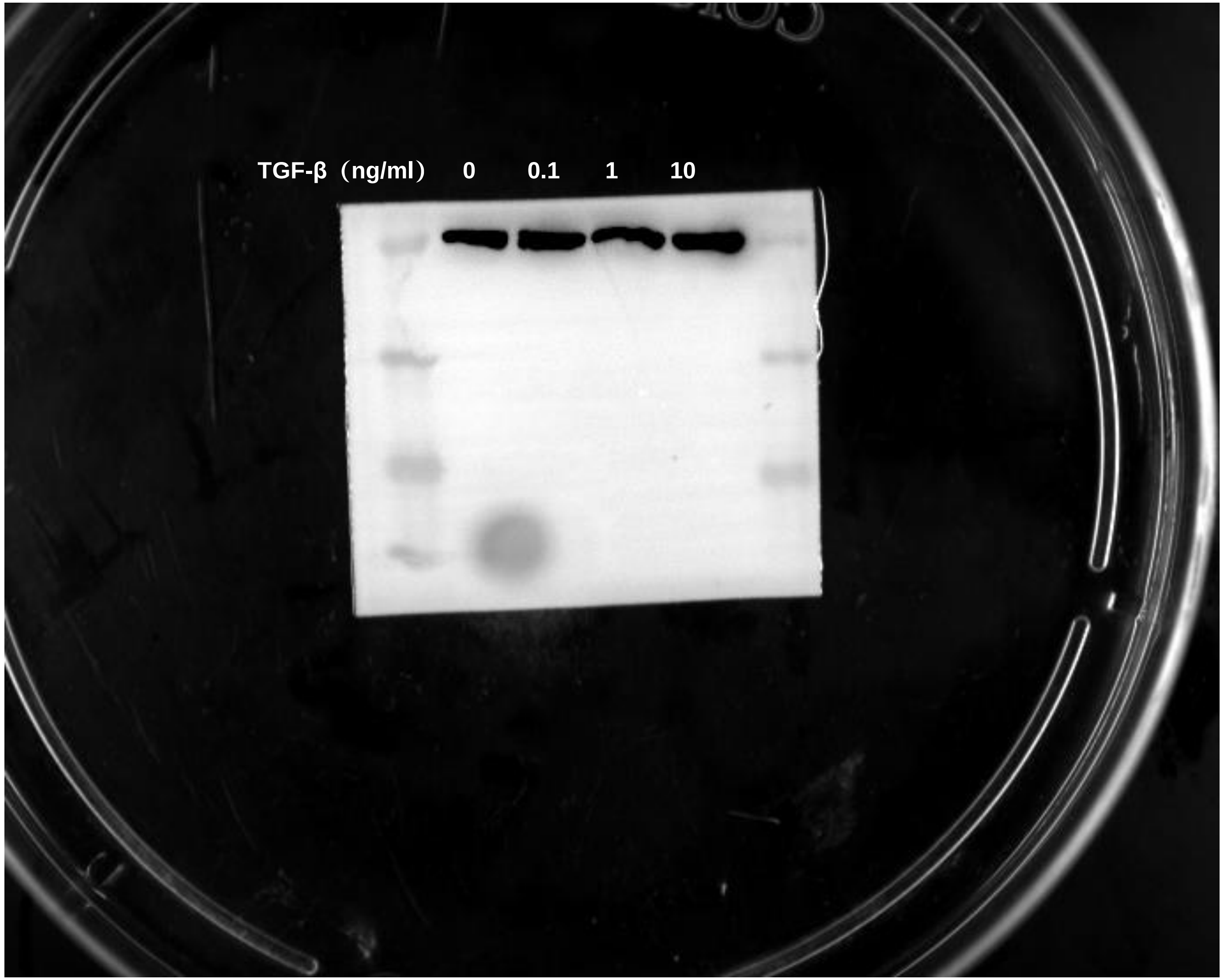

figure3-B-1

Fibronectin(220kDa)

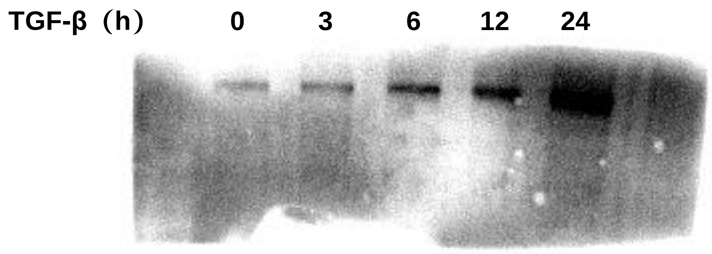

Fibronectin(220kDa)

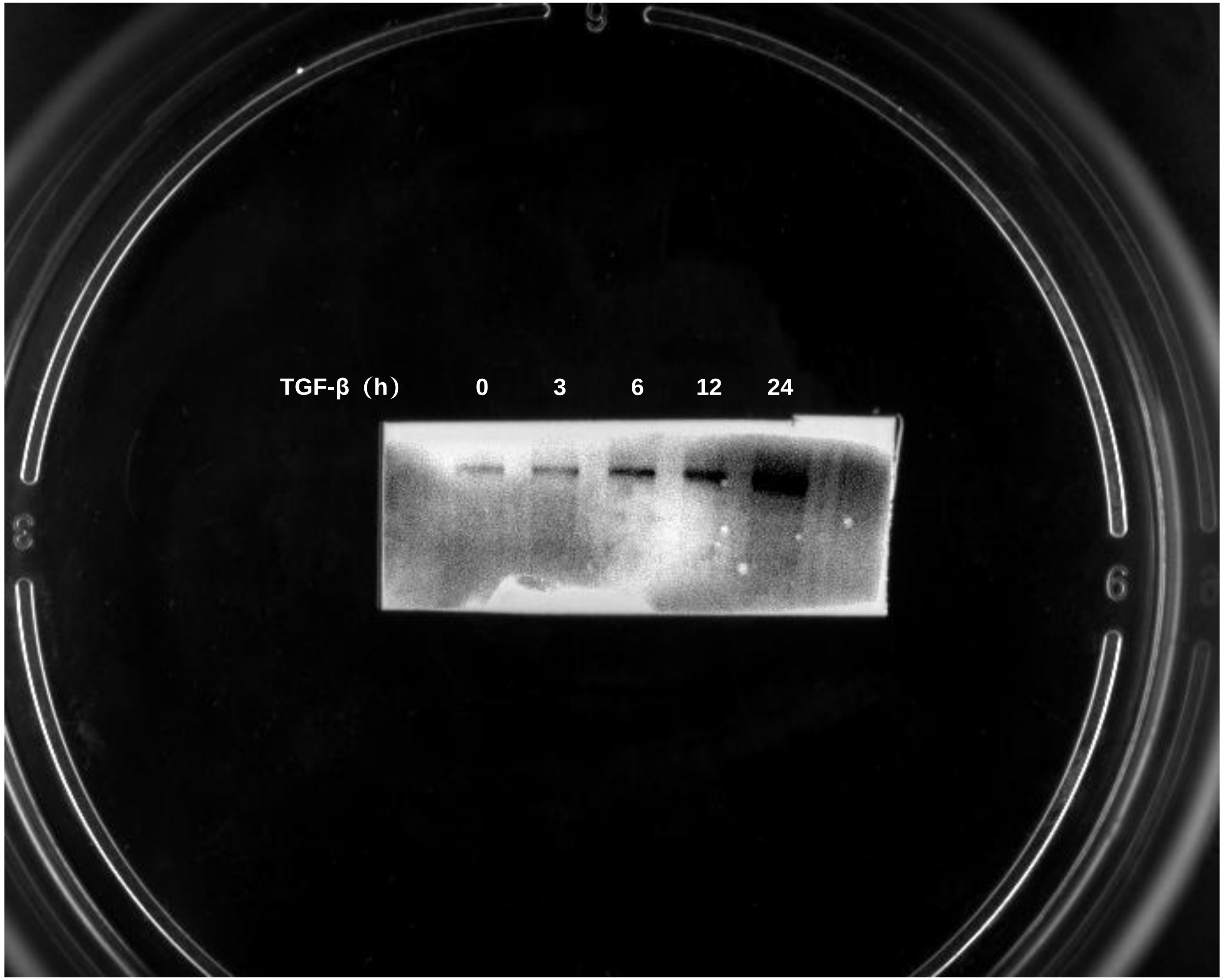

IFITM1（25-35KDa）

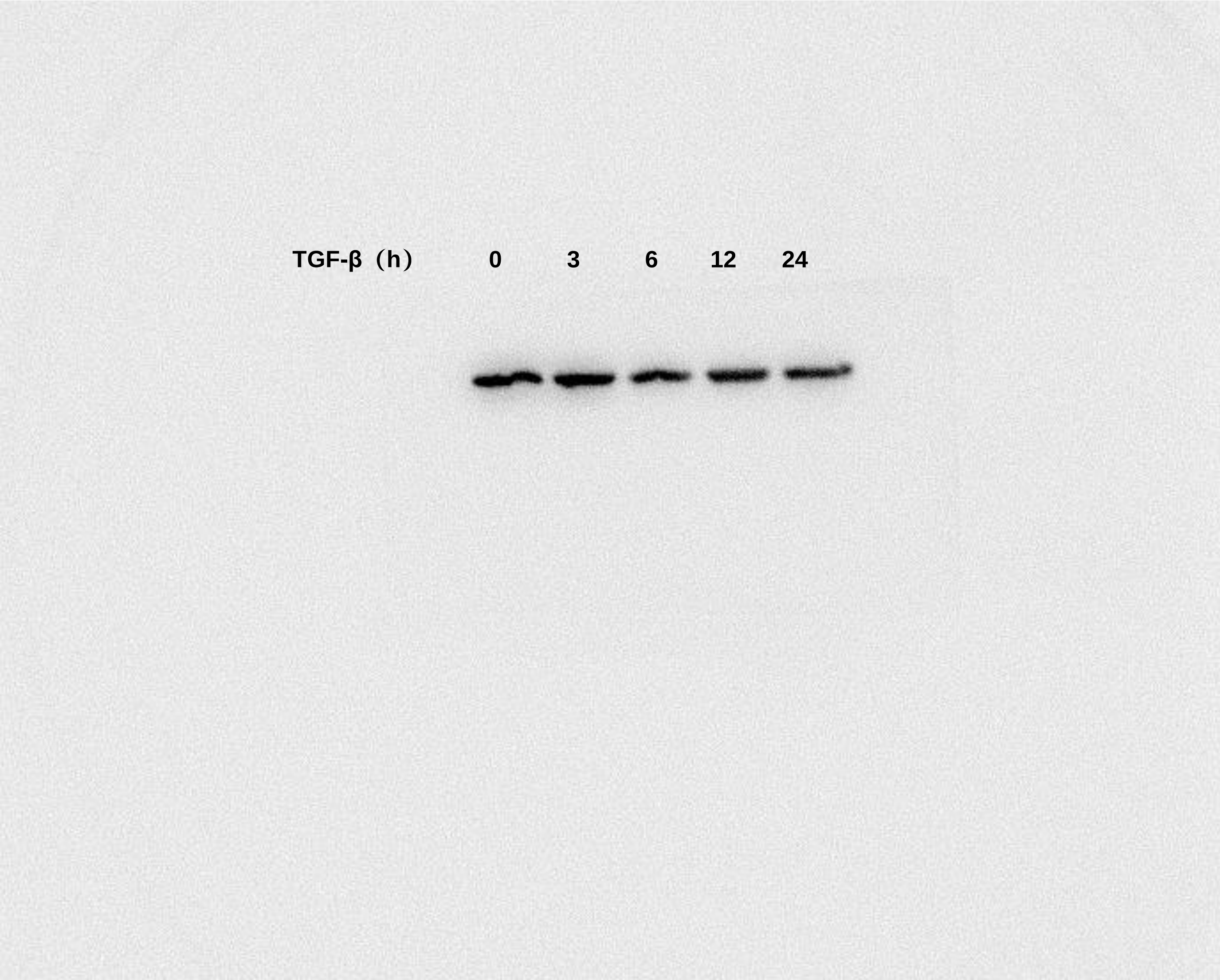

IFITM1（25-35KDa）

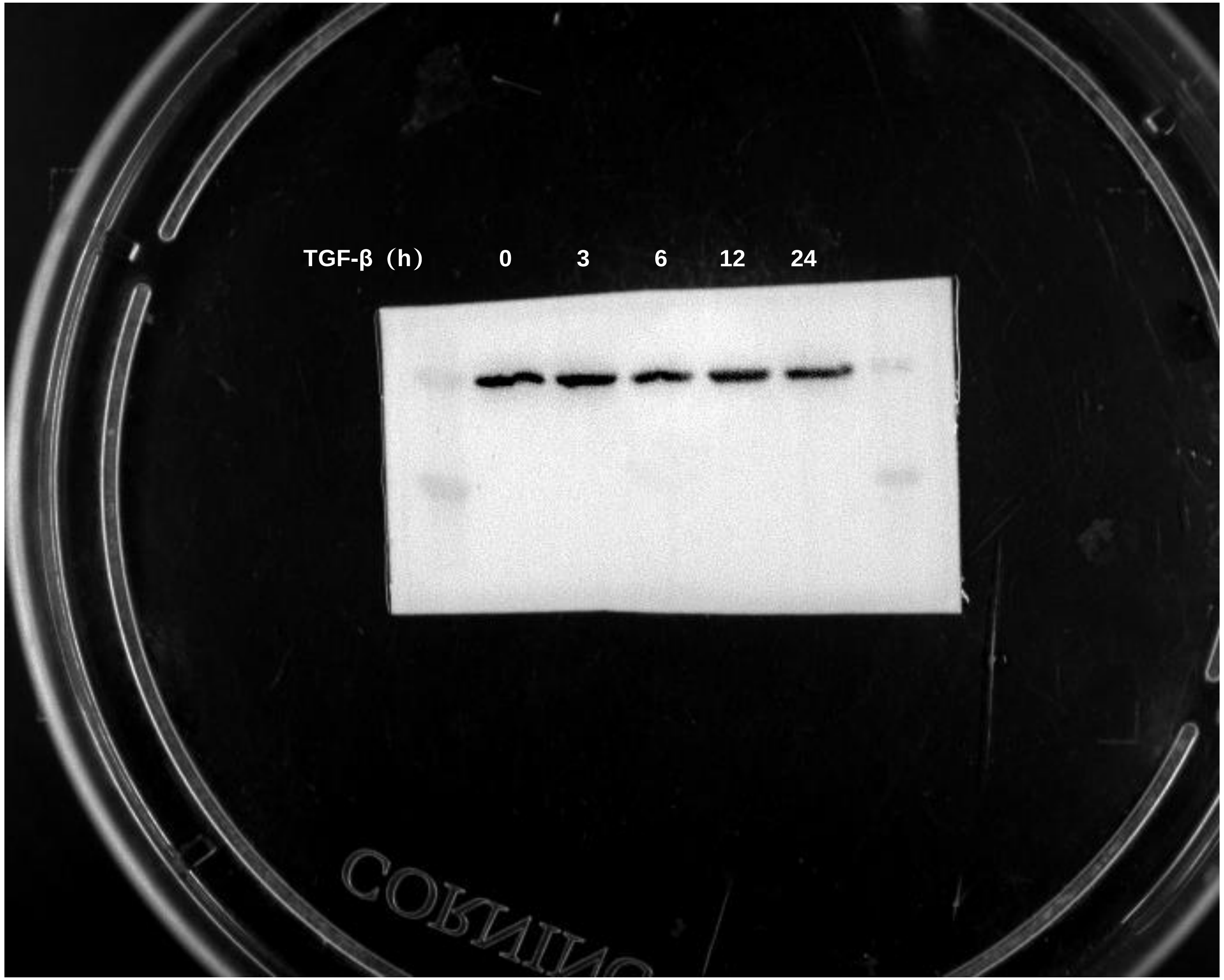

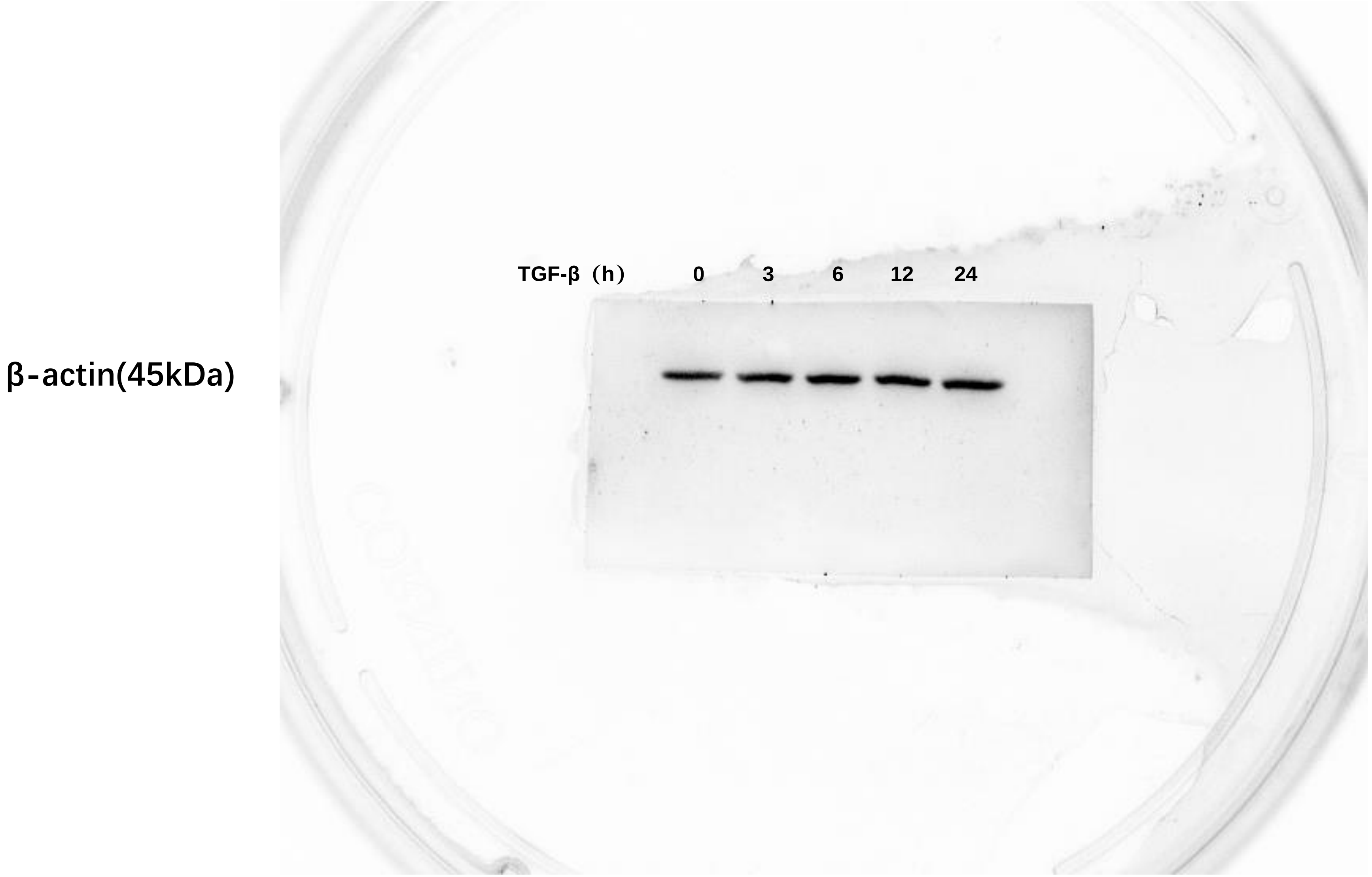
β-actin(45kDa)
TGF-β（h）
0
3
6
12
24

β-actin(45kDa)

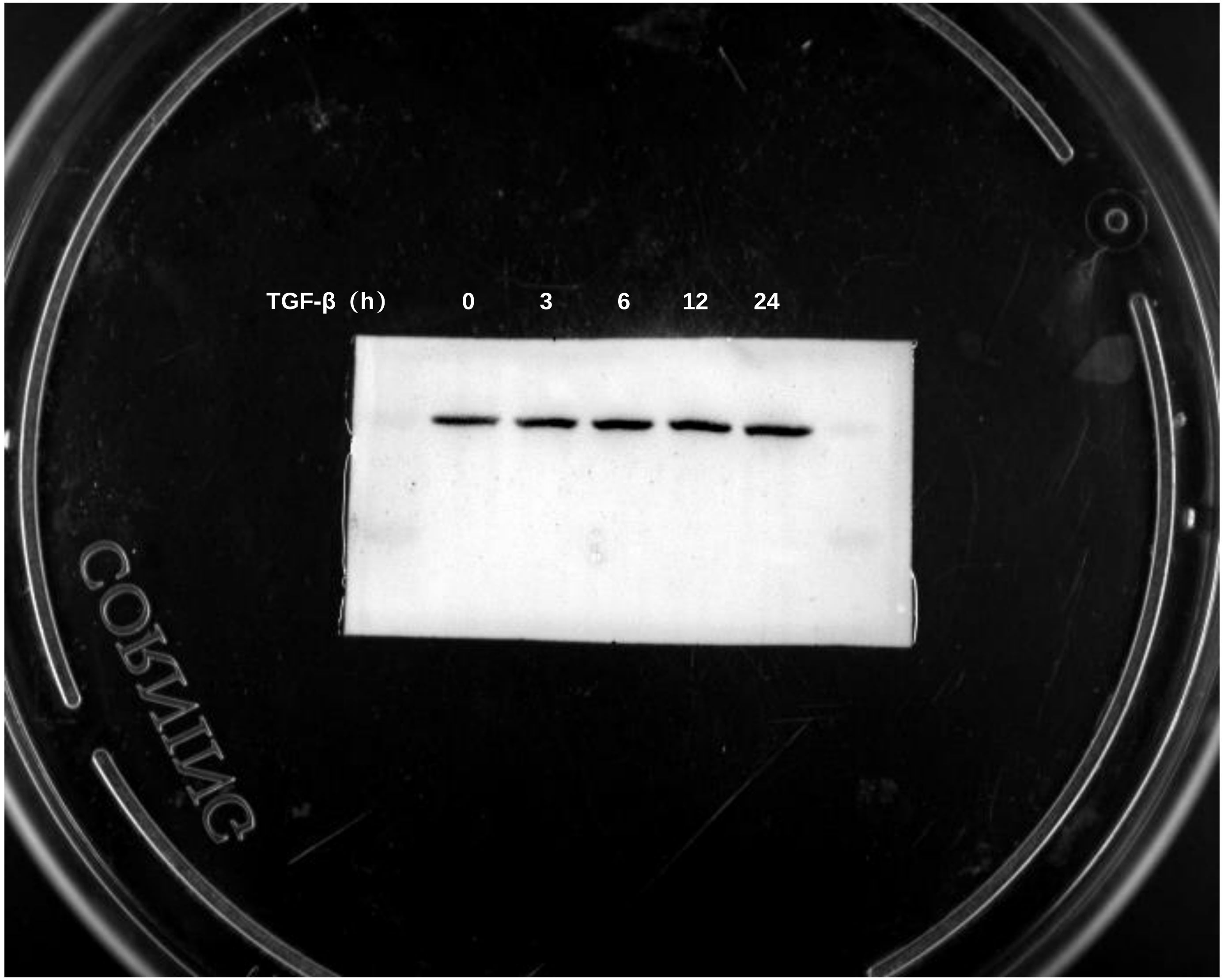

figure3-B-2

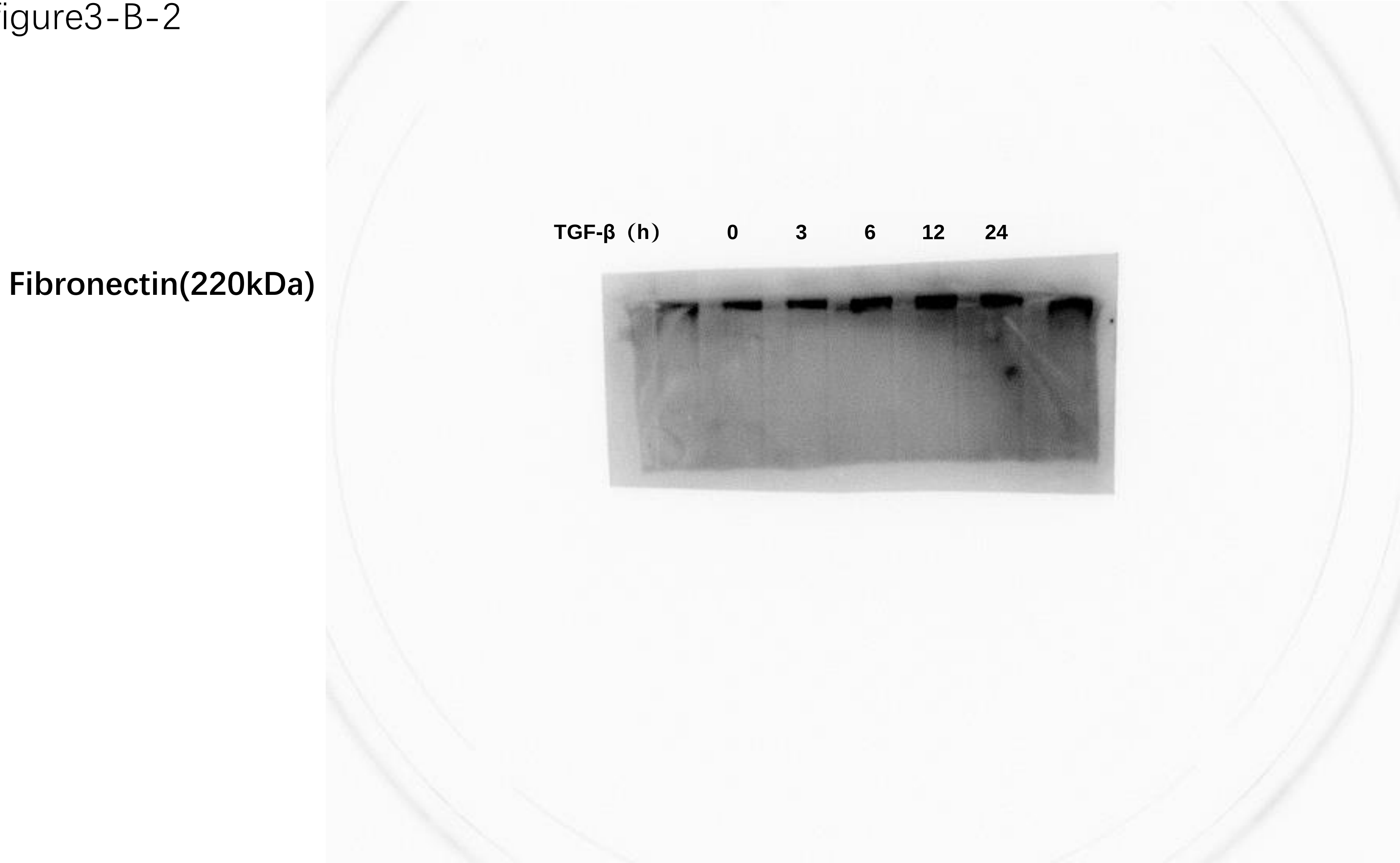

Fibronectin(220kDa)

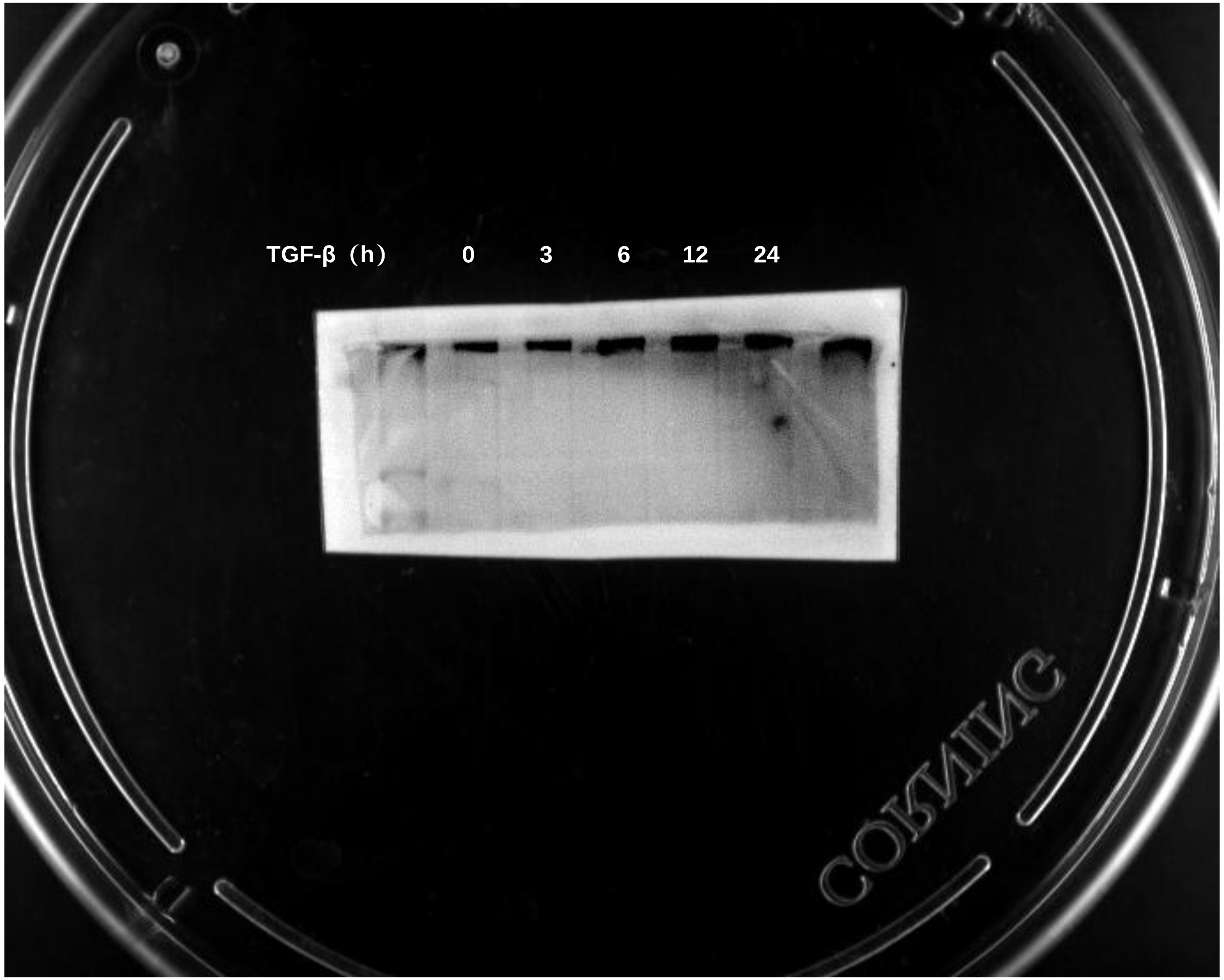

IFITM1（25-35KDa）

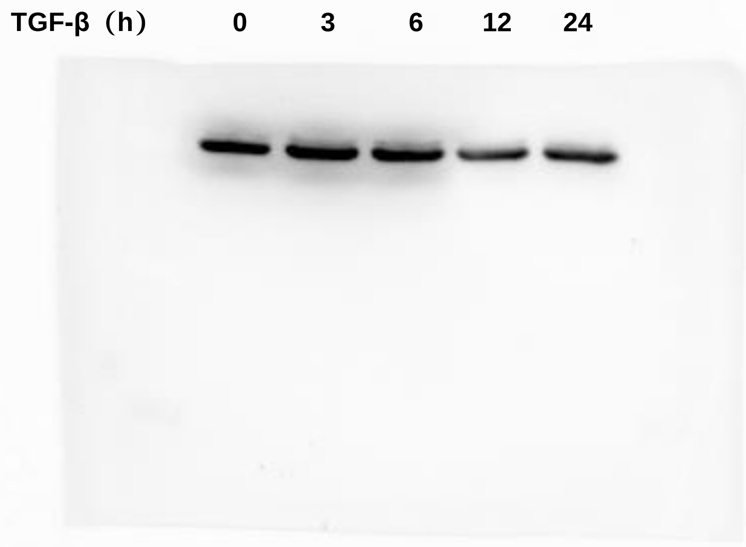

IFITM1（25-35KDa）

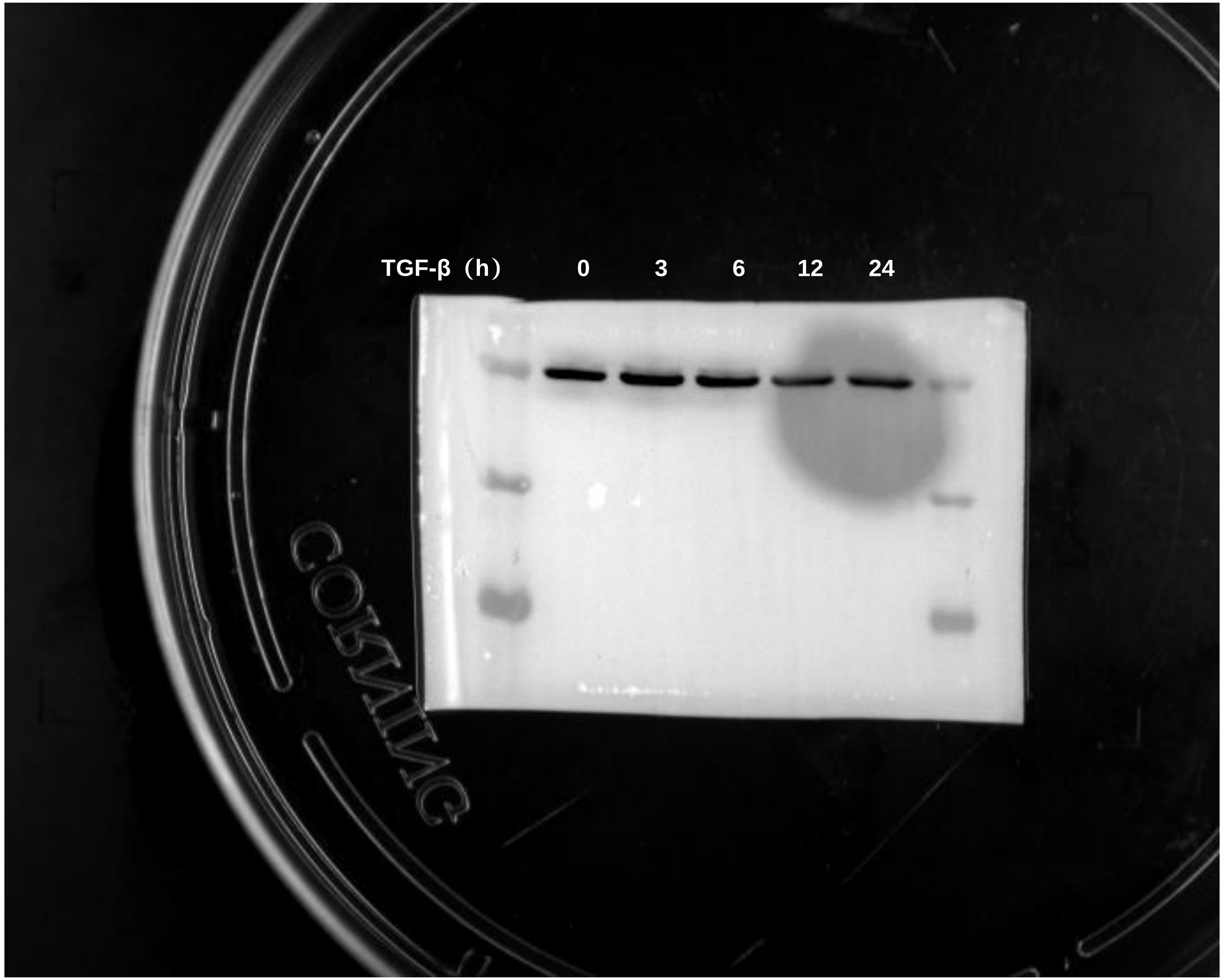

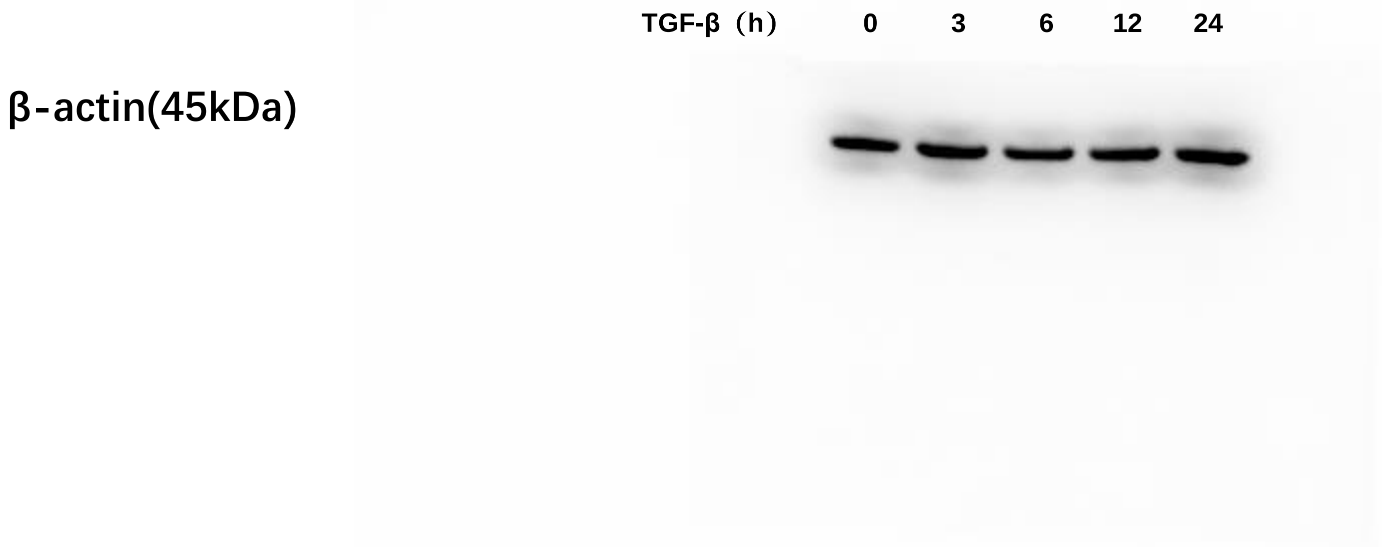
TGF-β（h）
0
3
6
12
24
β-actin(45kDa)

β-actin(45kDa)

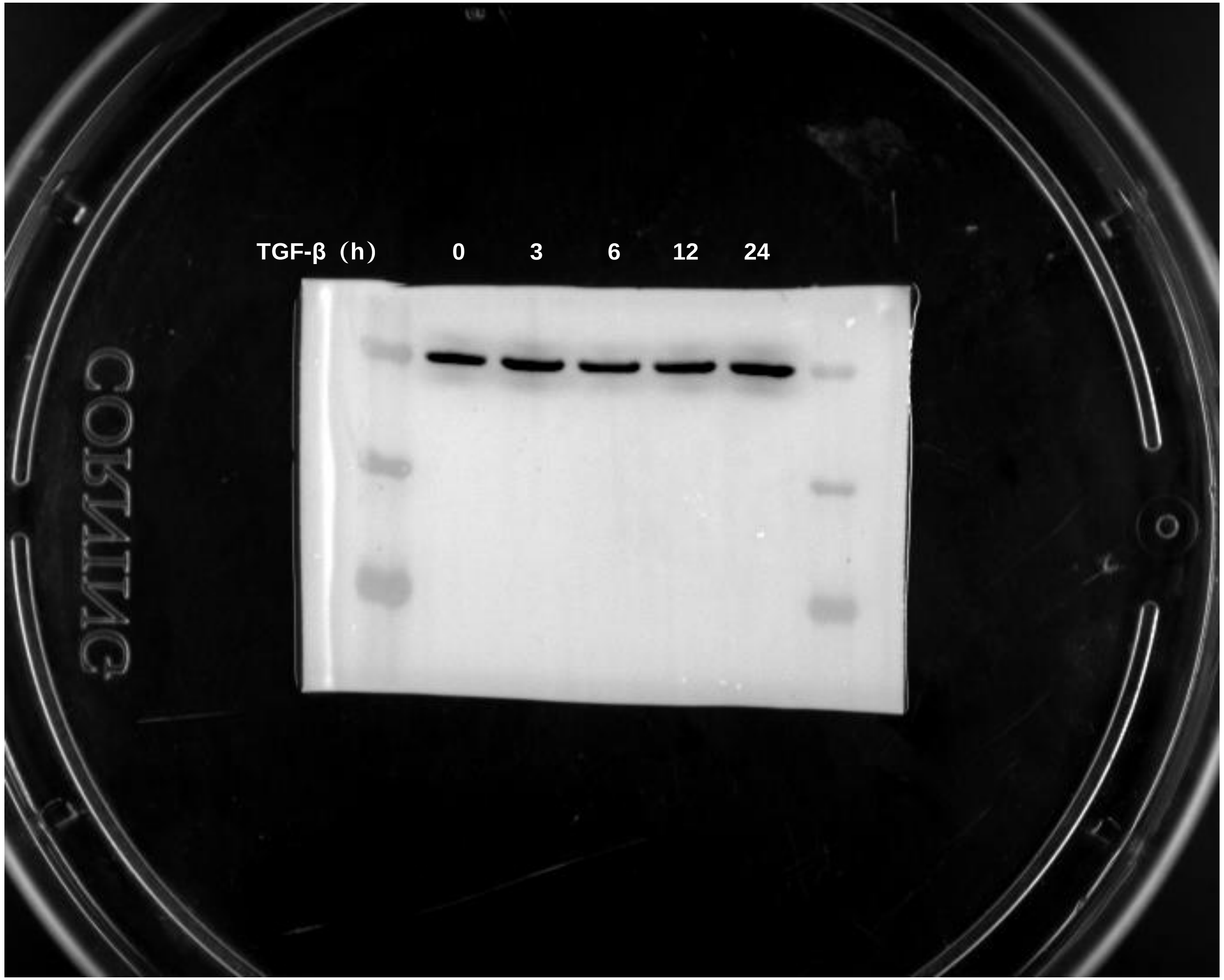

figure3-B-3

**Fibronectin(220kDa)**

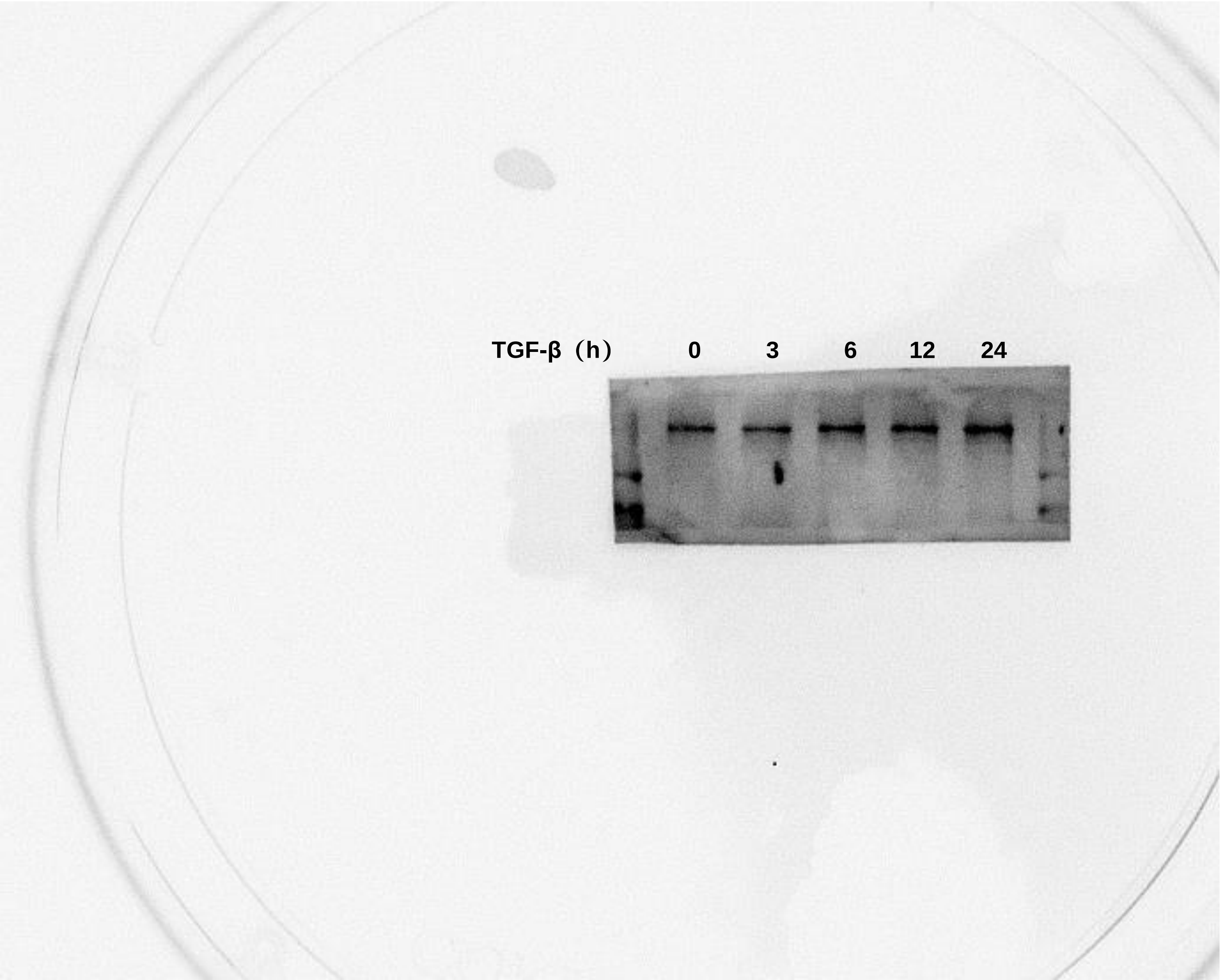

Fibronectin(220kDa)

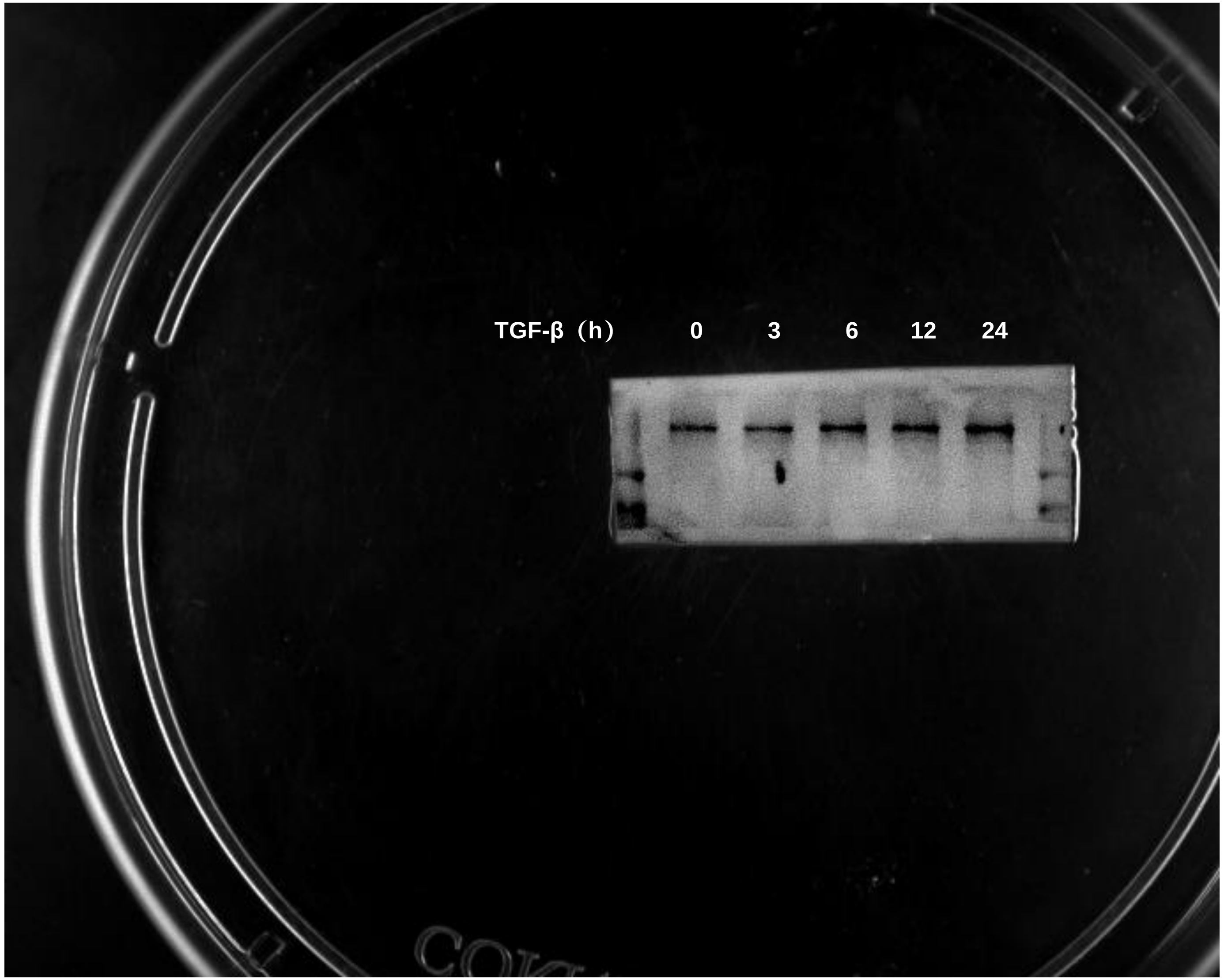

IFITM1（25-35KDa）

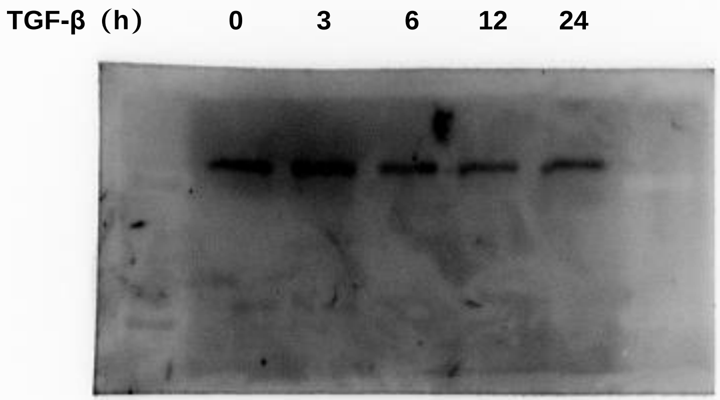

IFITM1（25-35KDa）

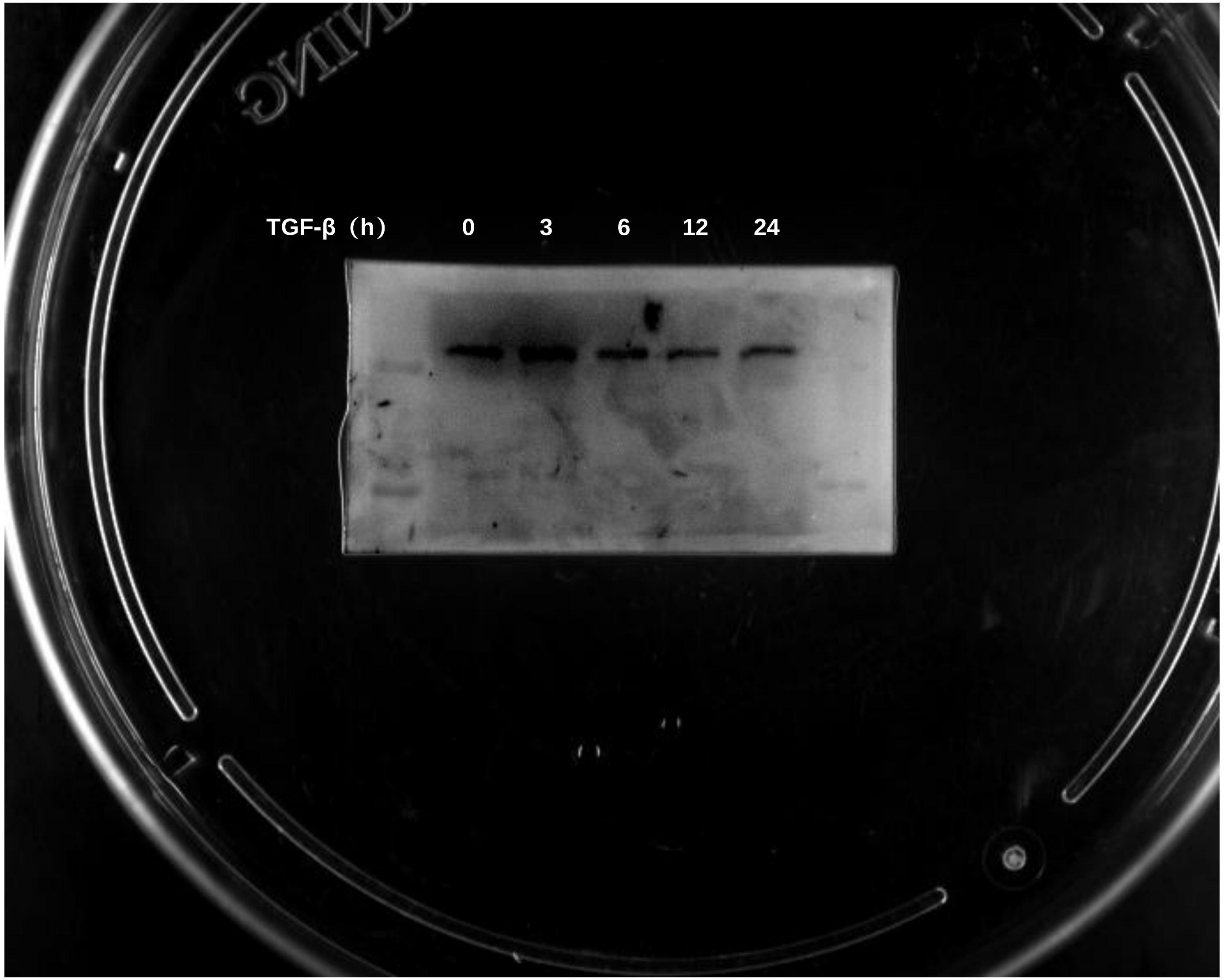

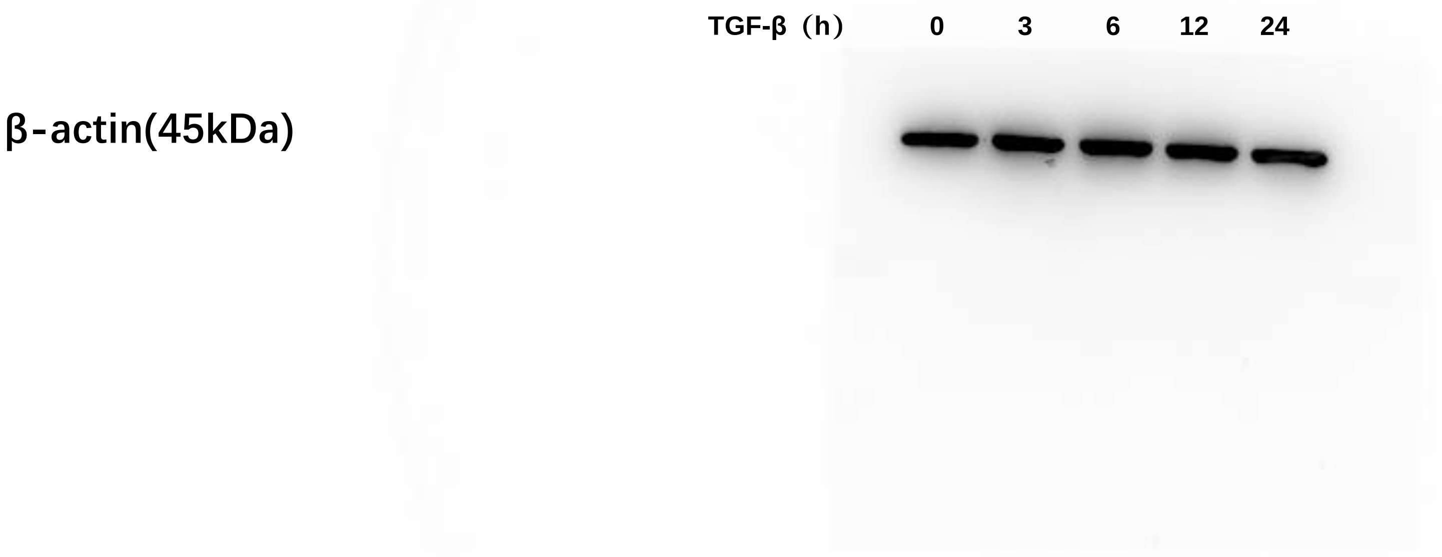
TGF-β（h） 0 3 6 12 24
β-actin(45kDa)

β-actin(45kDa)

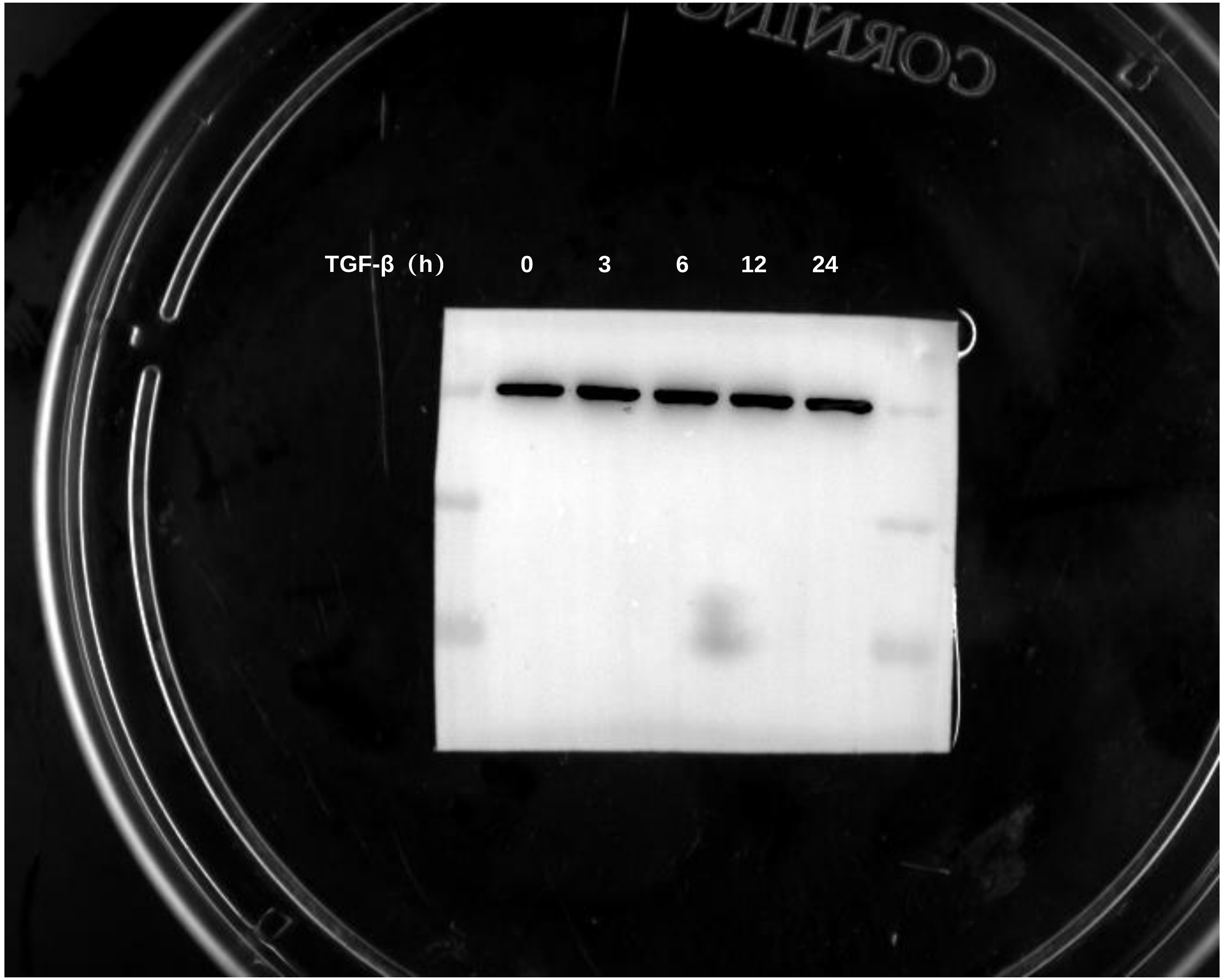

figure3-C-1

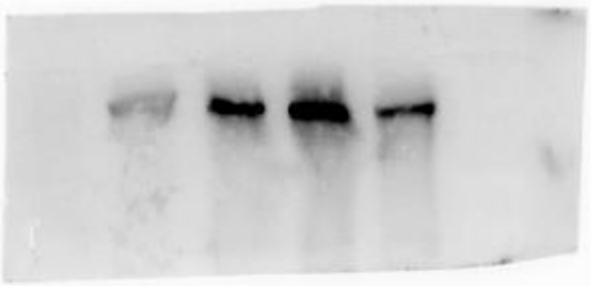

Fibronectin(220kDa)

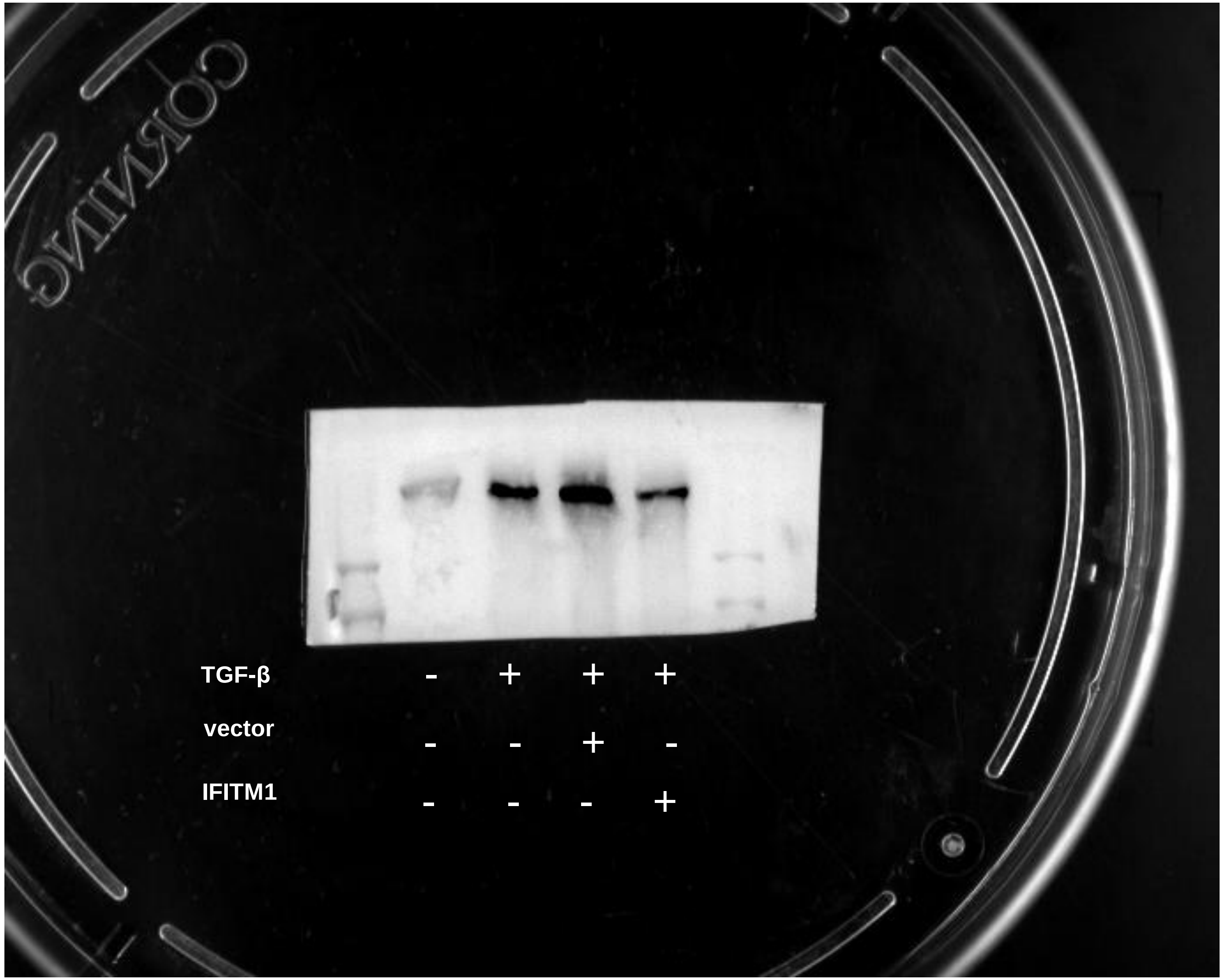

IFITM1（25-35KDa）

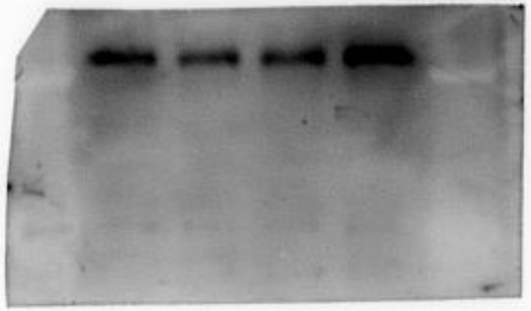

| | | | | |
|---|---|---|---|---|
| TGF-β | - | + | + | + |
| vector | - | - | + | - |
| IFITM1 | - | - | - | + |

IFITM1（25-35KDa）

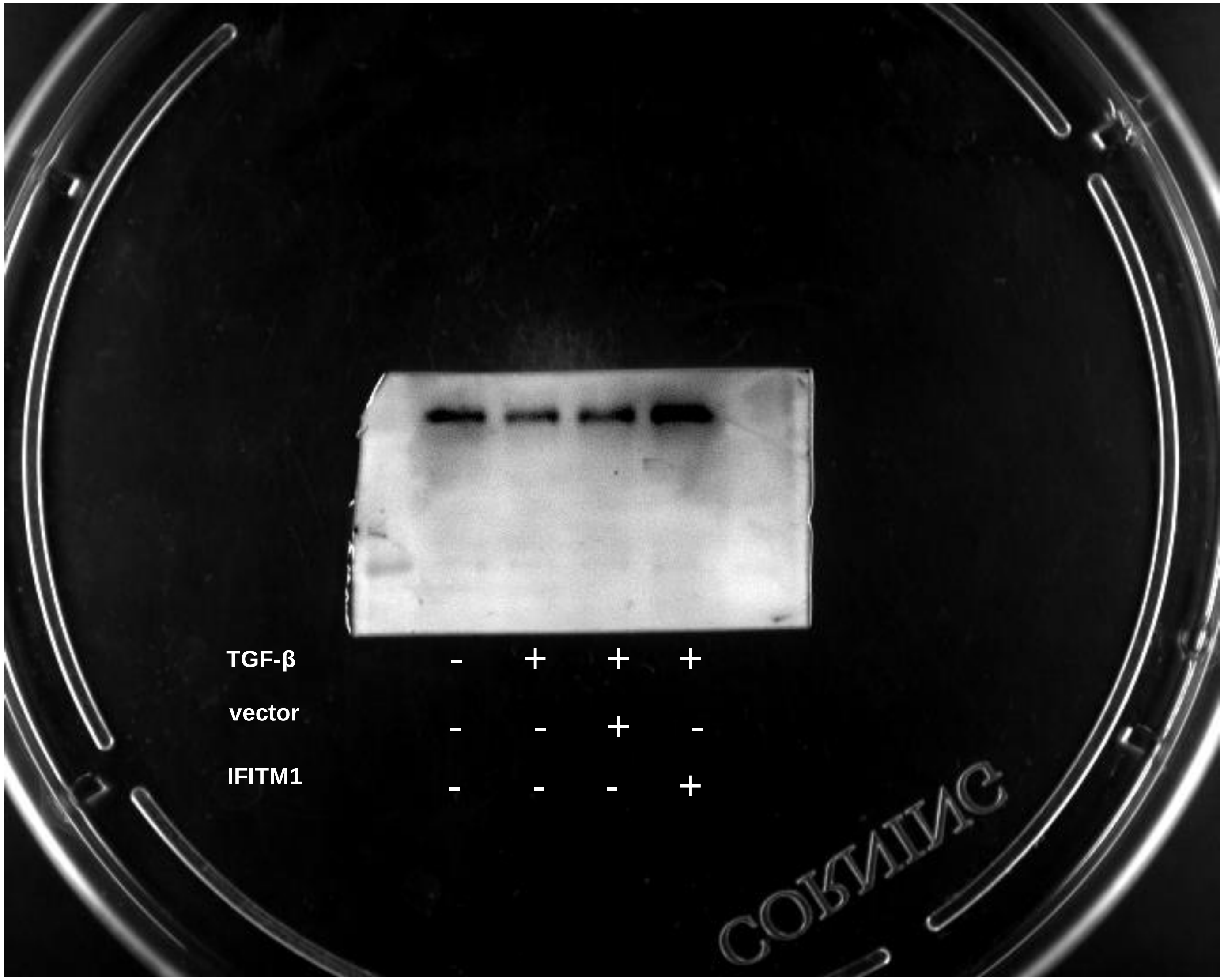

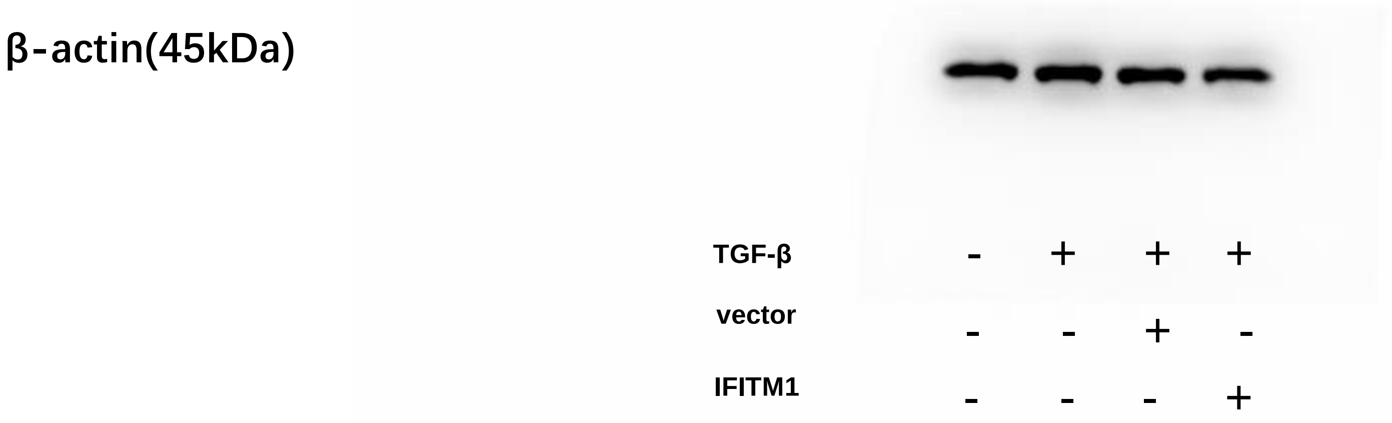
β-actin(45kDa)
TGF-β - + + +
vector - - + -
IFITM1 - - - +

β-actin(45kDa)

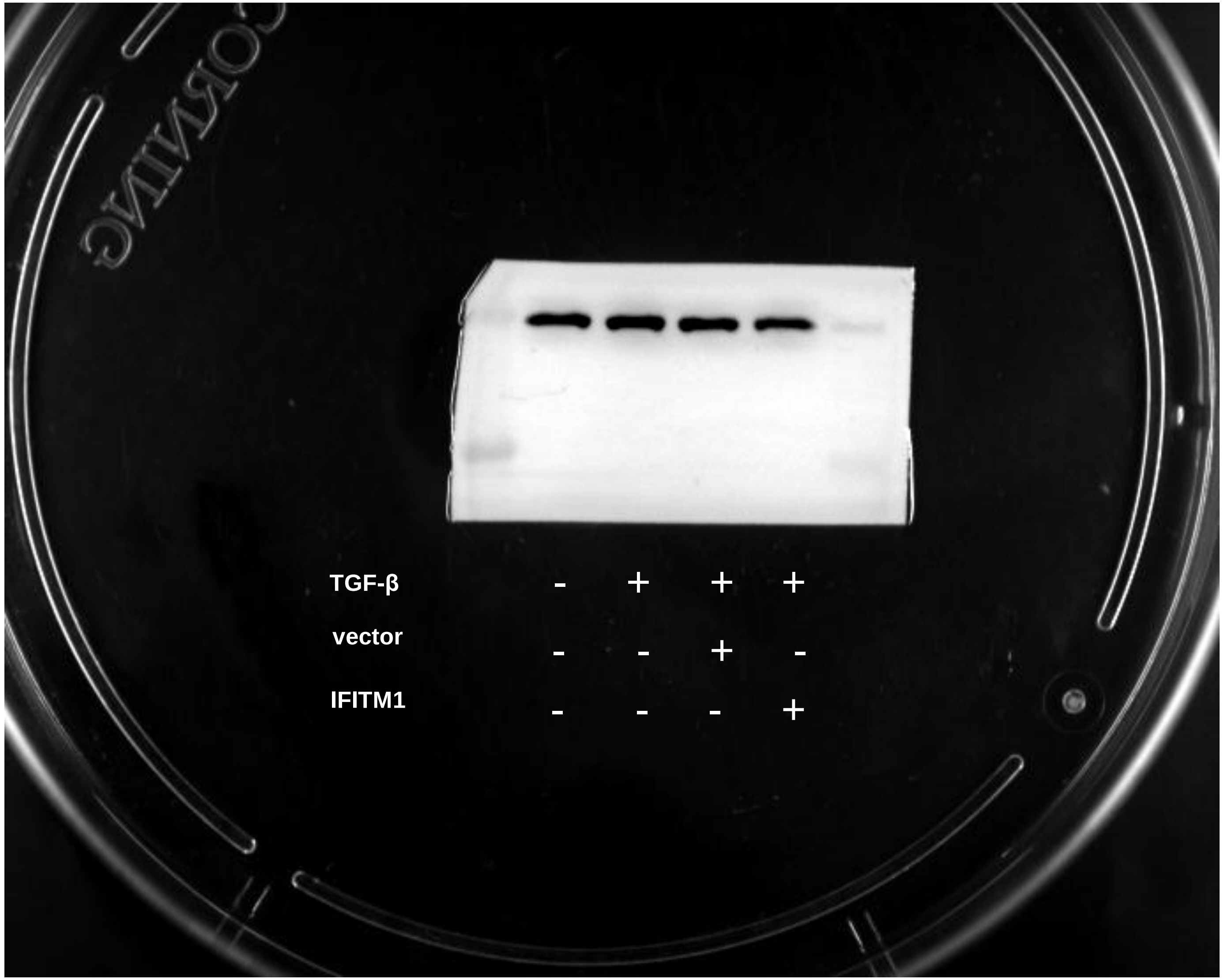

figure3-C-2

Fibronectin(220kDa)

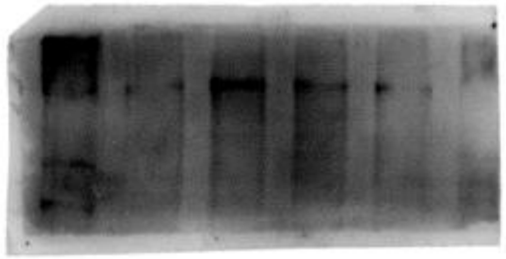

| | | | | |
|---|---|---|---|---|
| TGF-β | - | + | + | + |
| vector | - | - | + | - |
| IFITM1 | - | - | - | + |

Fibronectin(220kDa)

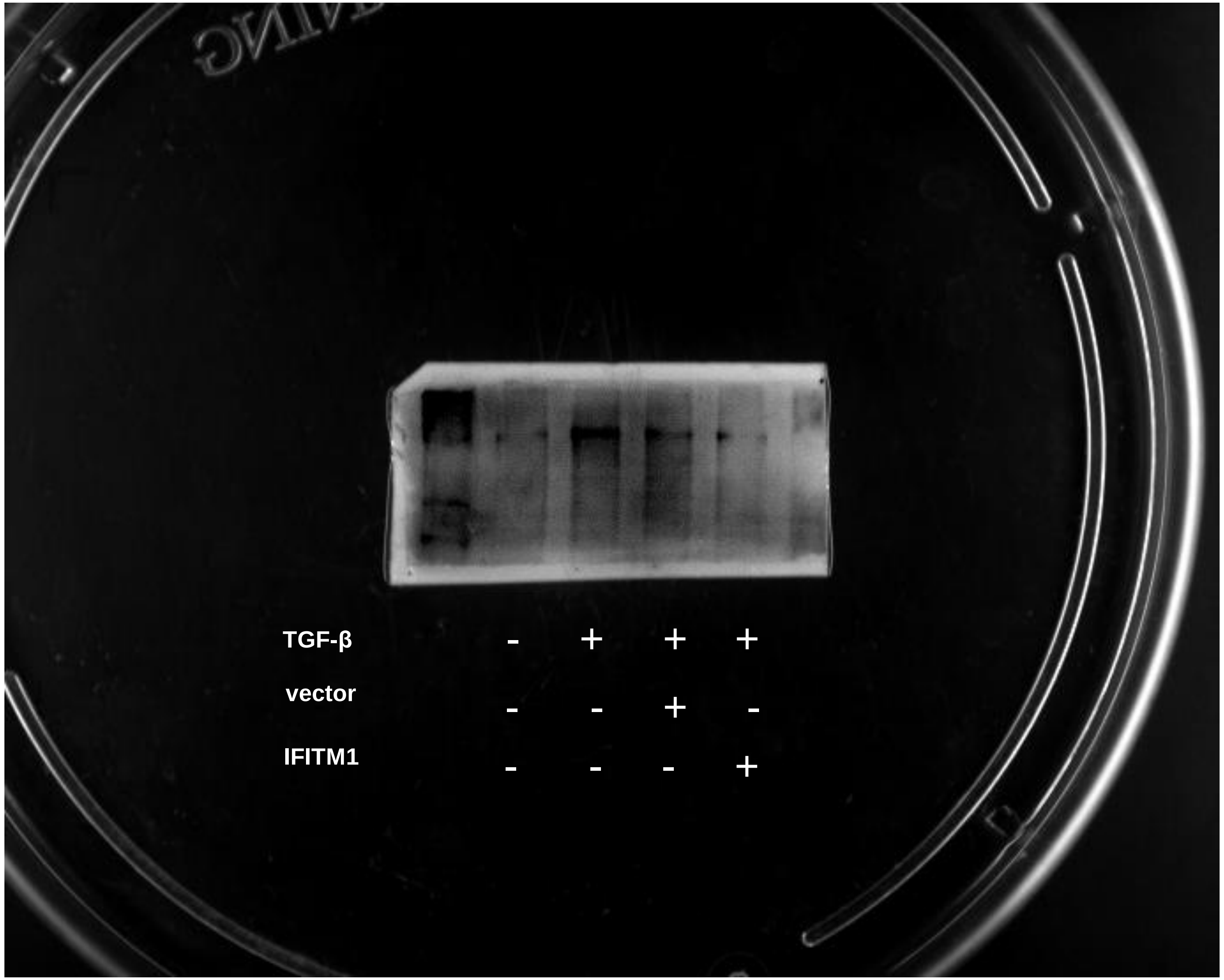

IFITM1（25-35KDa）

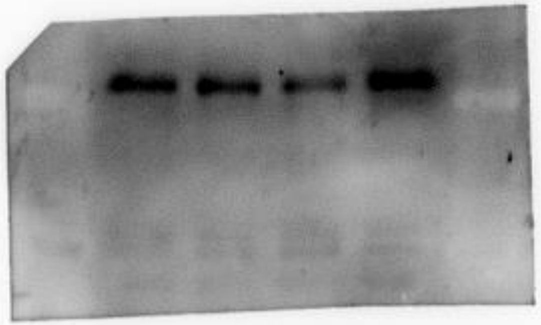

| | | | | |
|---|---|---|---|---|
| TGF-β | - | + | + | + |
| vector | - | - | + | - |
| IFITM1 | - | - | - | + |

IFITM1（25-35KDa）

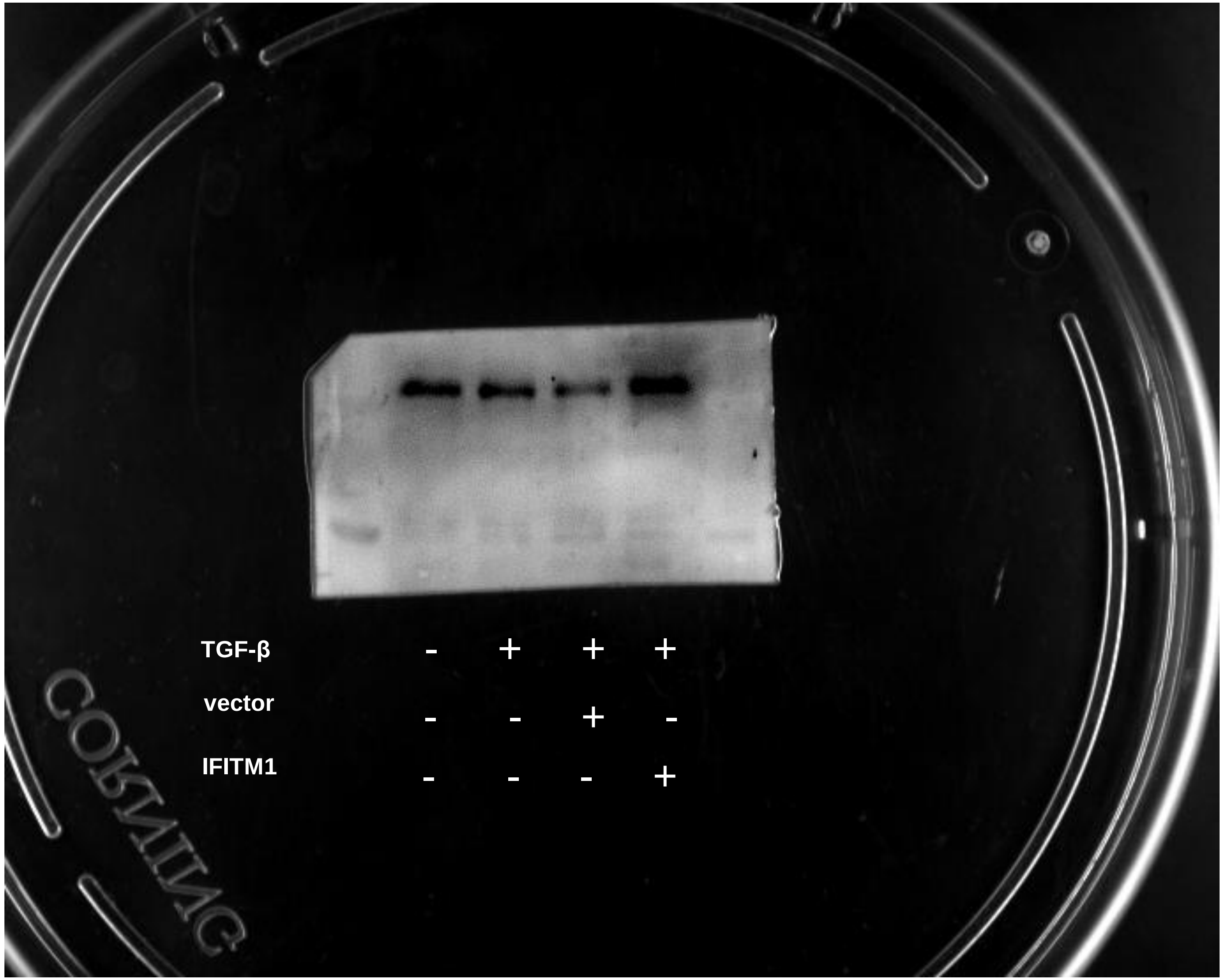

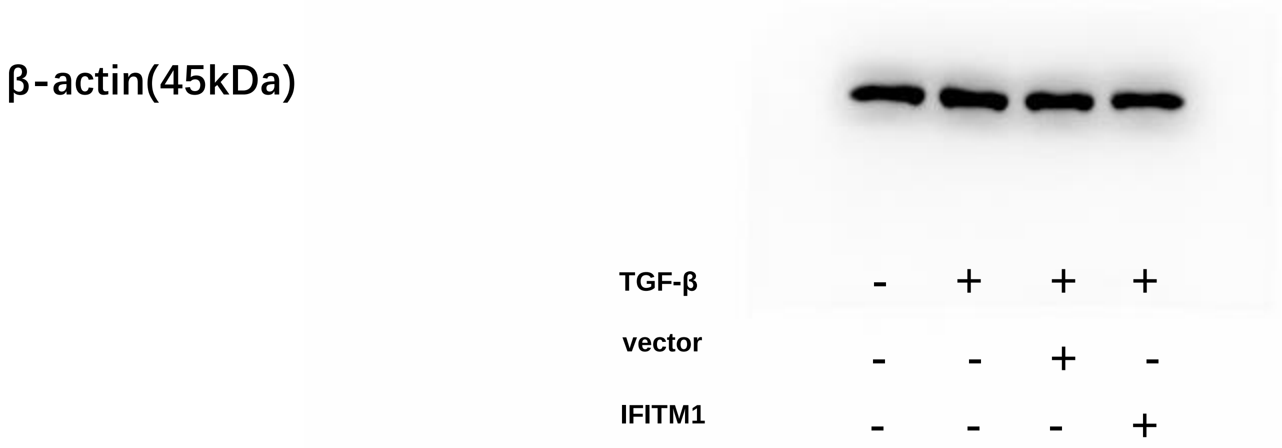
β-actin(45kDa)
TGF-β - + + +
vector - - + -
IFITM1 - - - +

β-actin(45kDa)

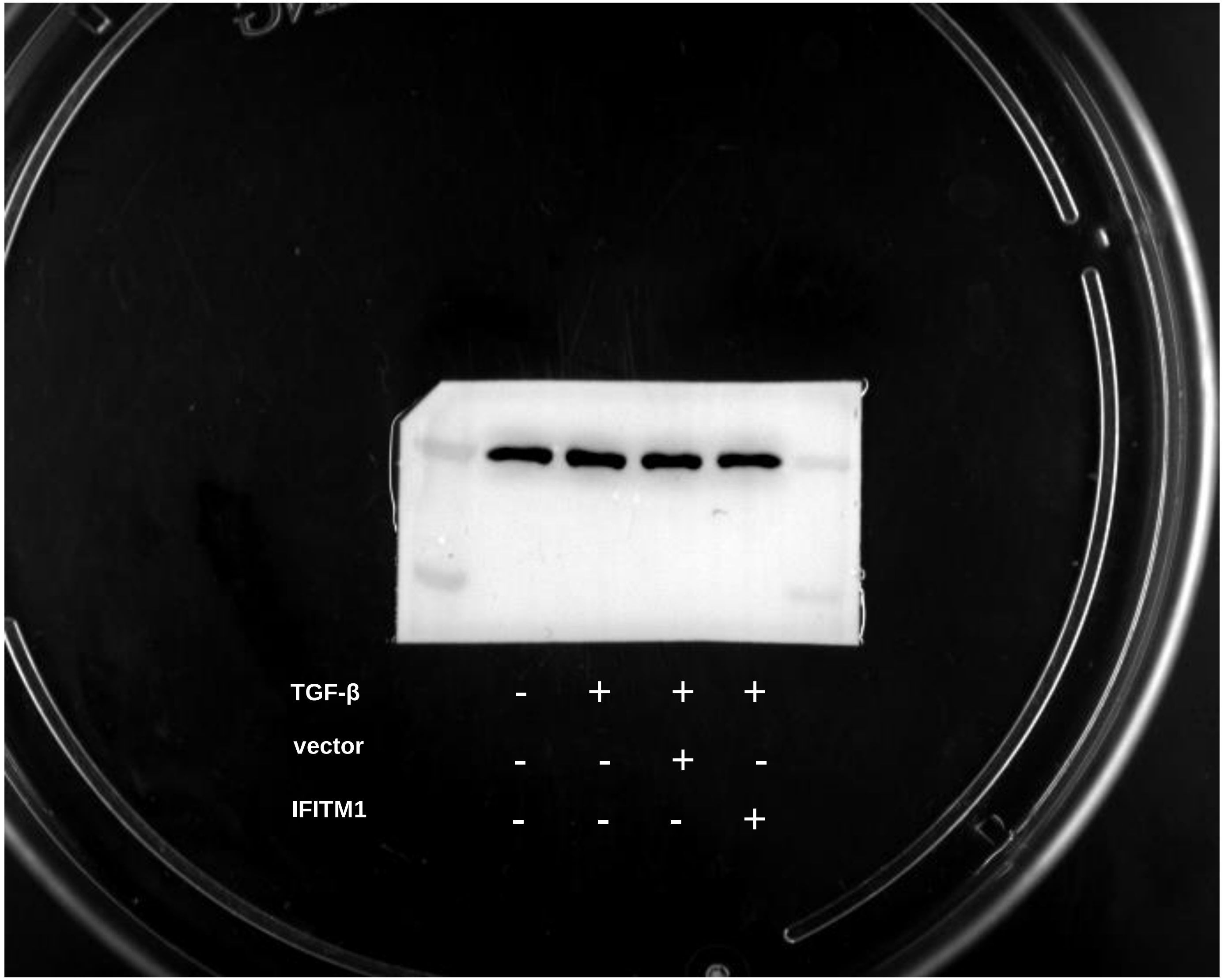

figure3-C-3

**Fibronectin(220kDa)**

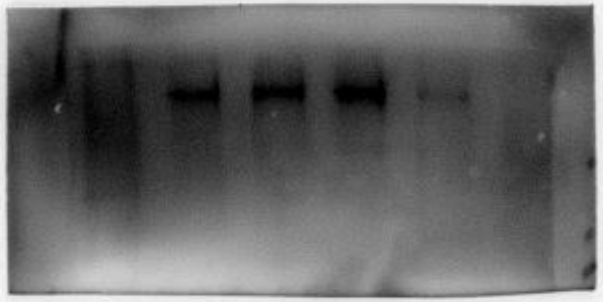

| | | | | |
|---|---|---|---|---|
| TGF-β | - | + | + | + |
| vector | - | - | + | - |
| IFITM1 | - | - | - | + |

Fibronectin(220kDa)

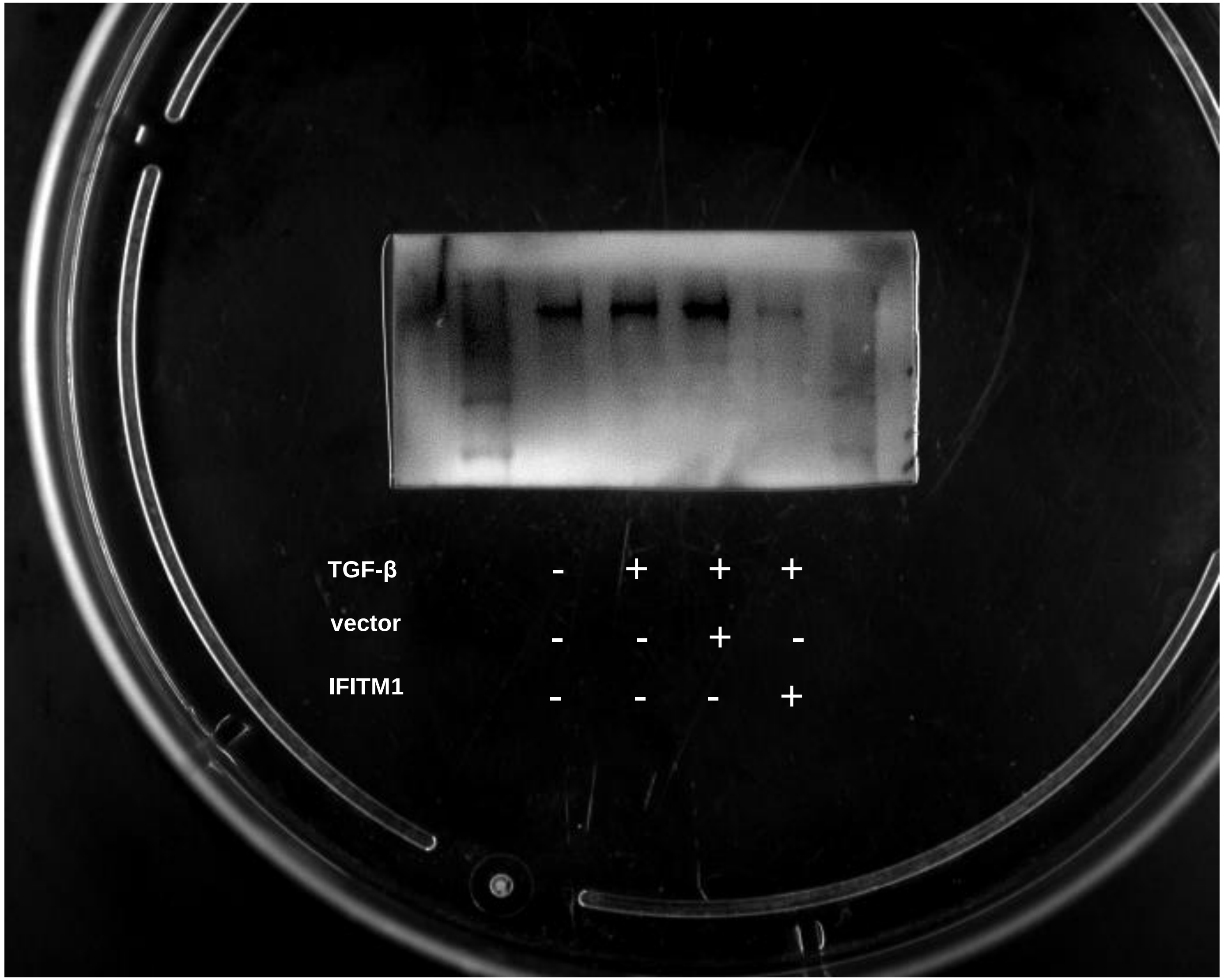

IFITM1（25-35KDa）

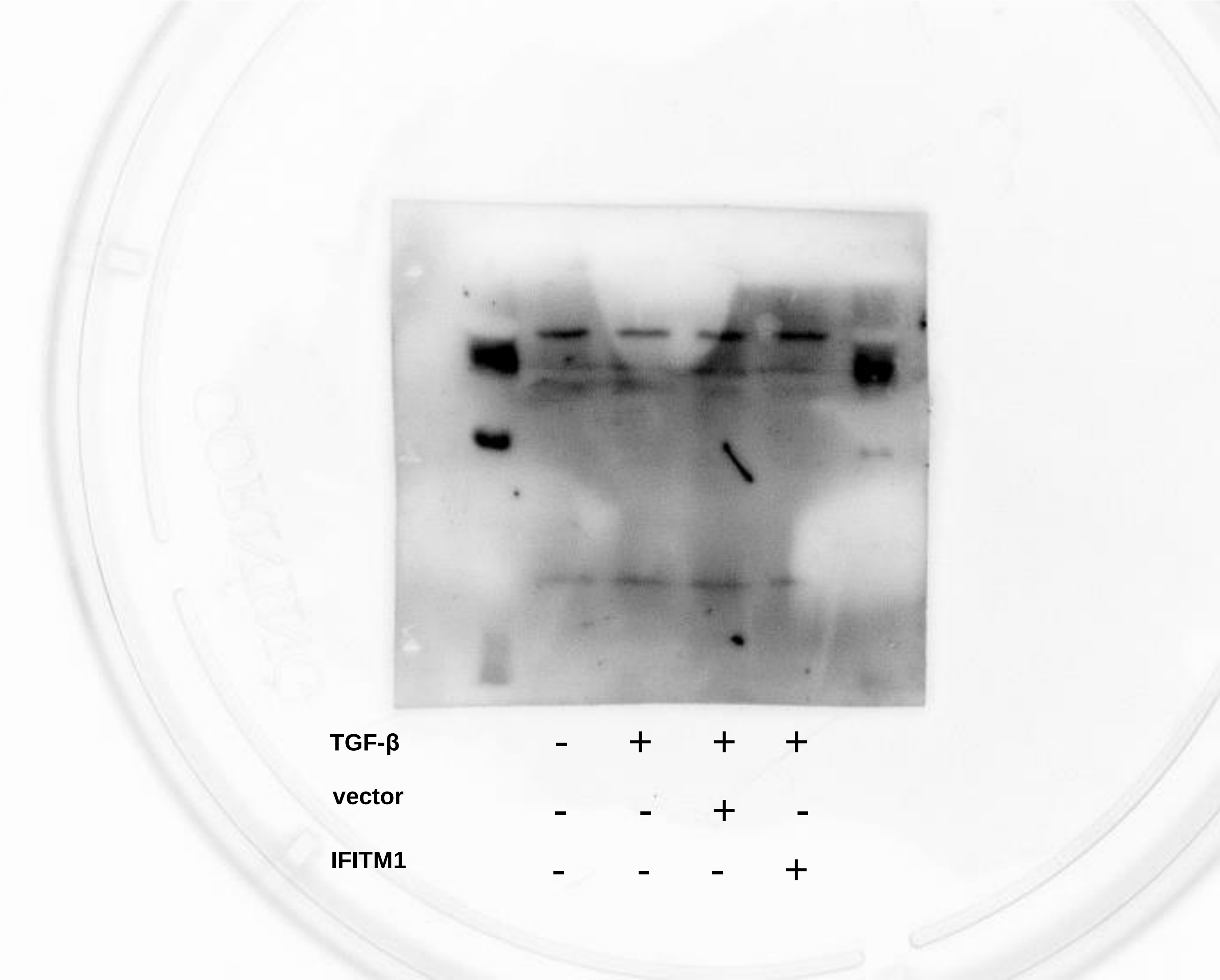

IFITM1（25-35KDa）

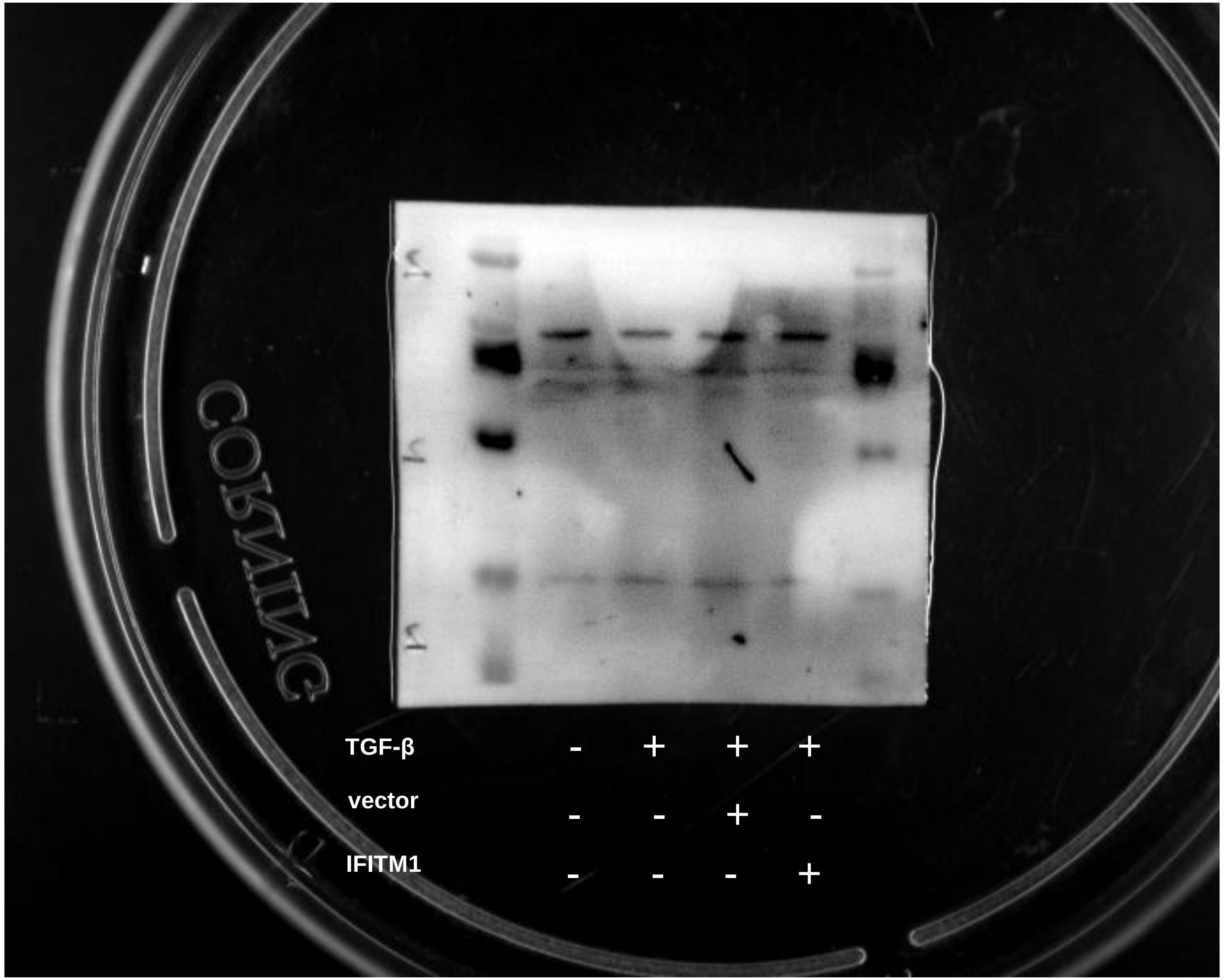

figure3-D

Fibronectin(220kDa)

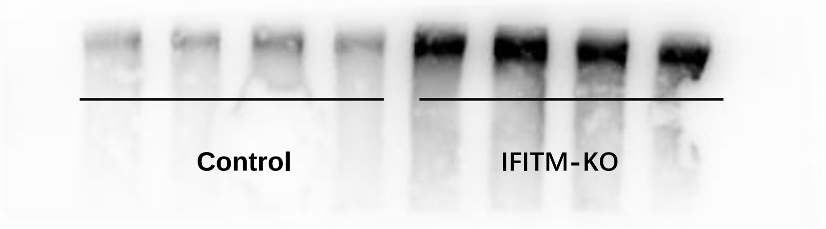

Fibronectin(220kDa)

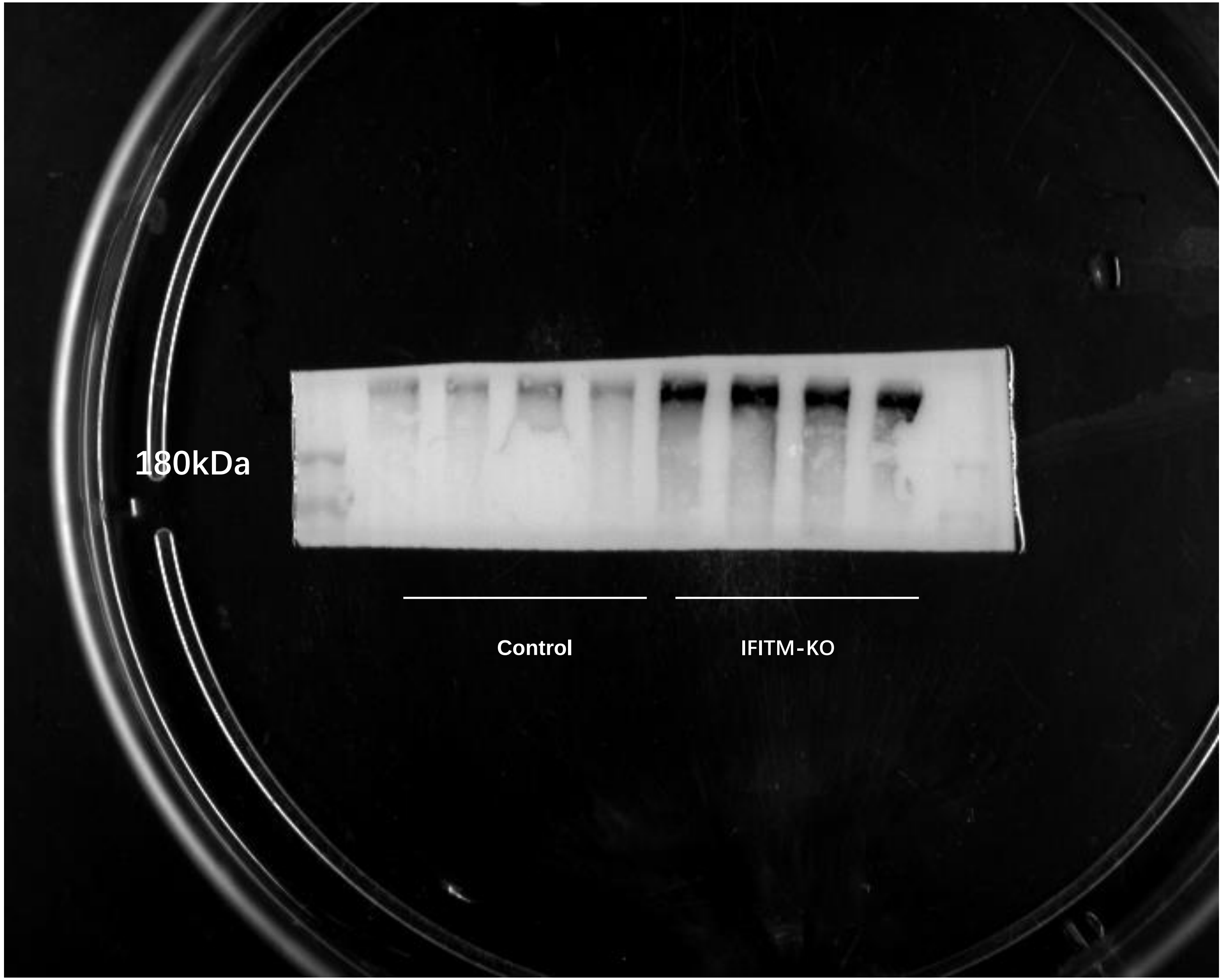

β-actin(45kDa)

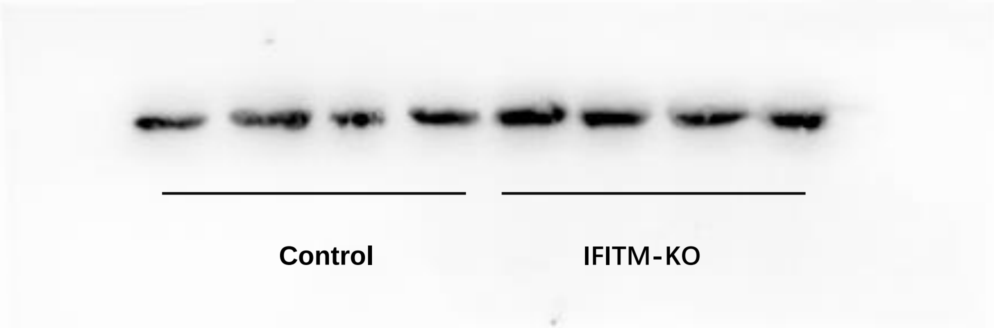

β-actin(45kDa)

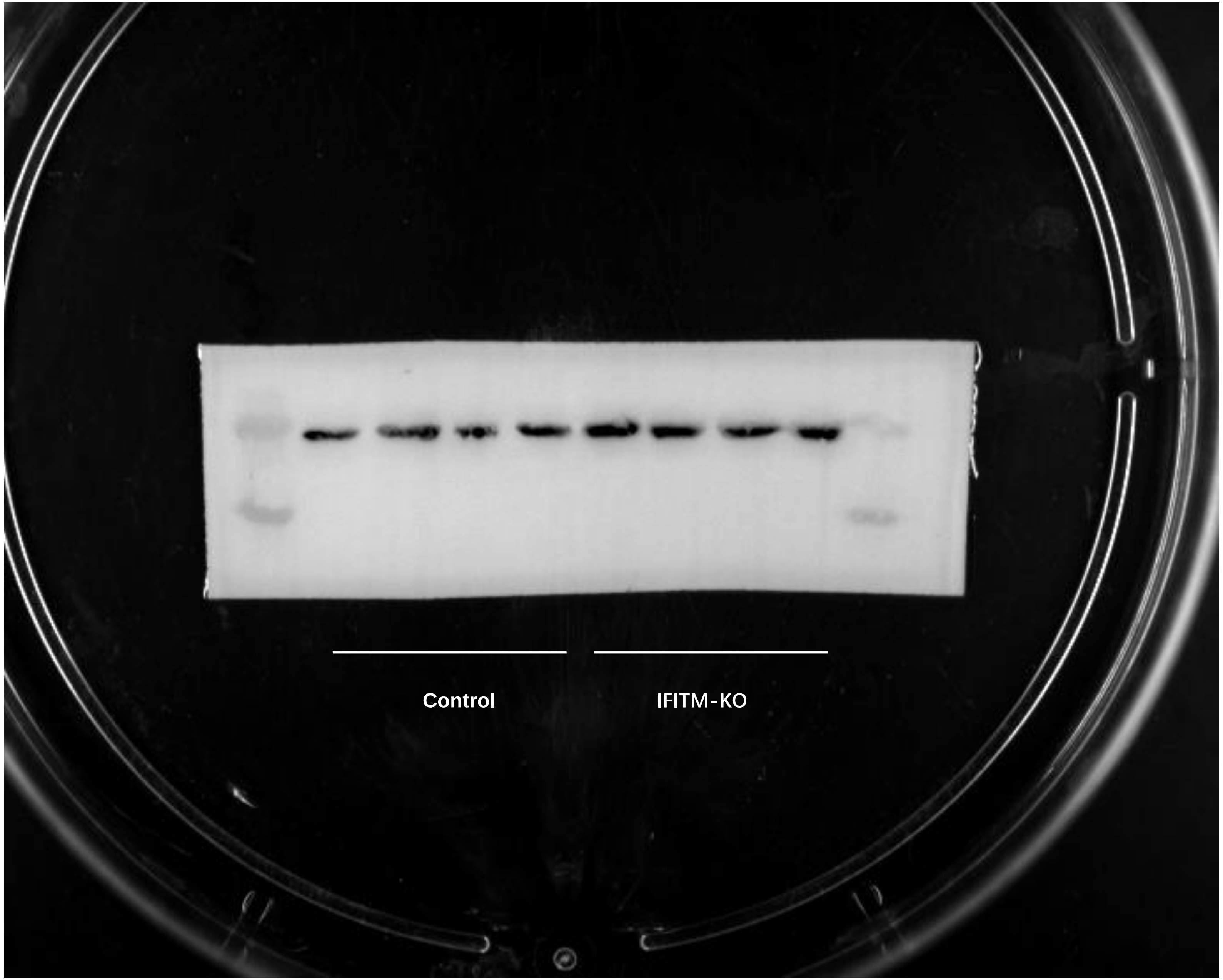

figure4-A-1

P-smad3(50-60kDa)

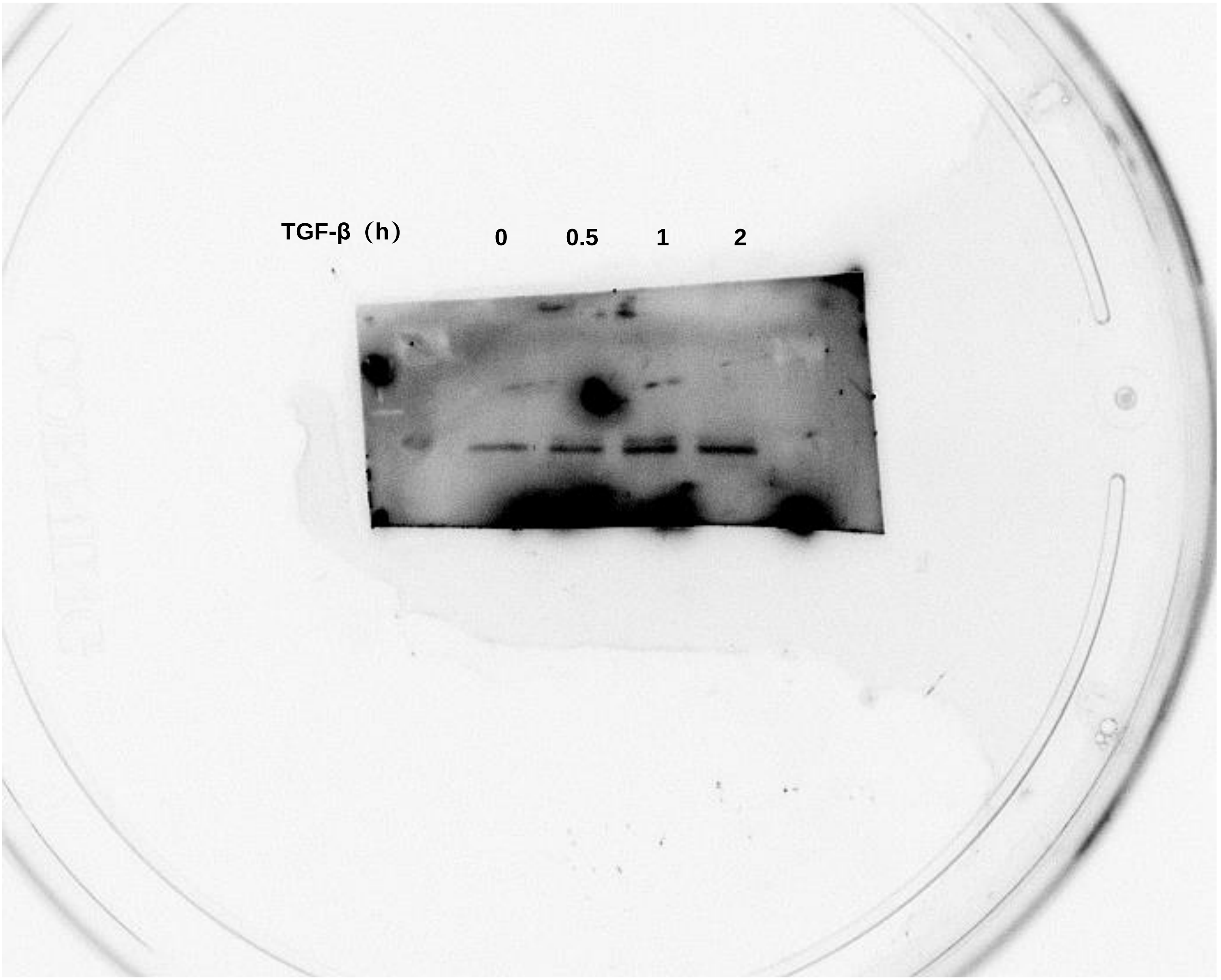

P-smad3(50-60kDa)

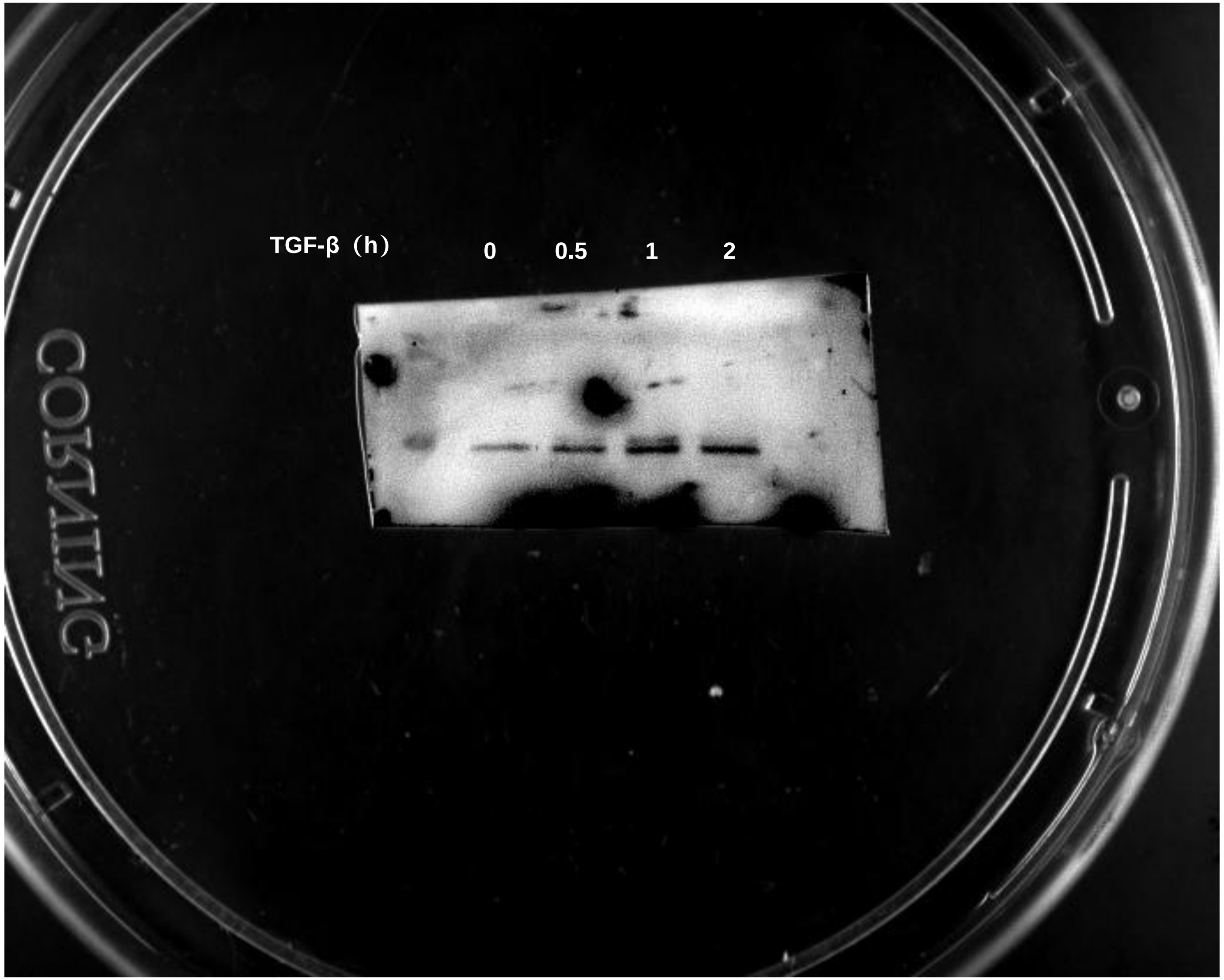

smad3(50-60kDa)

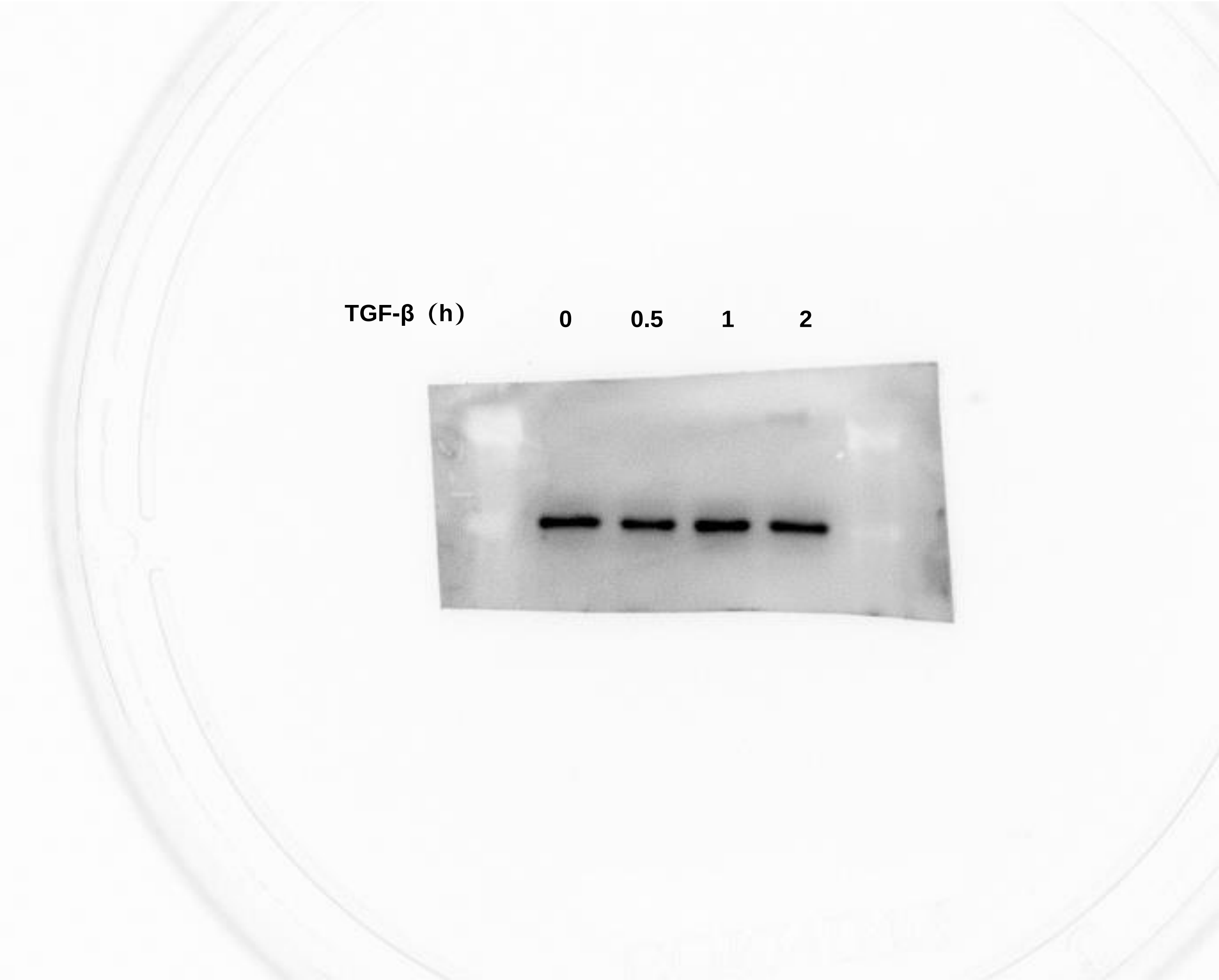

smad3(50-60kDa)

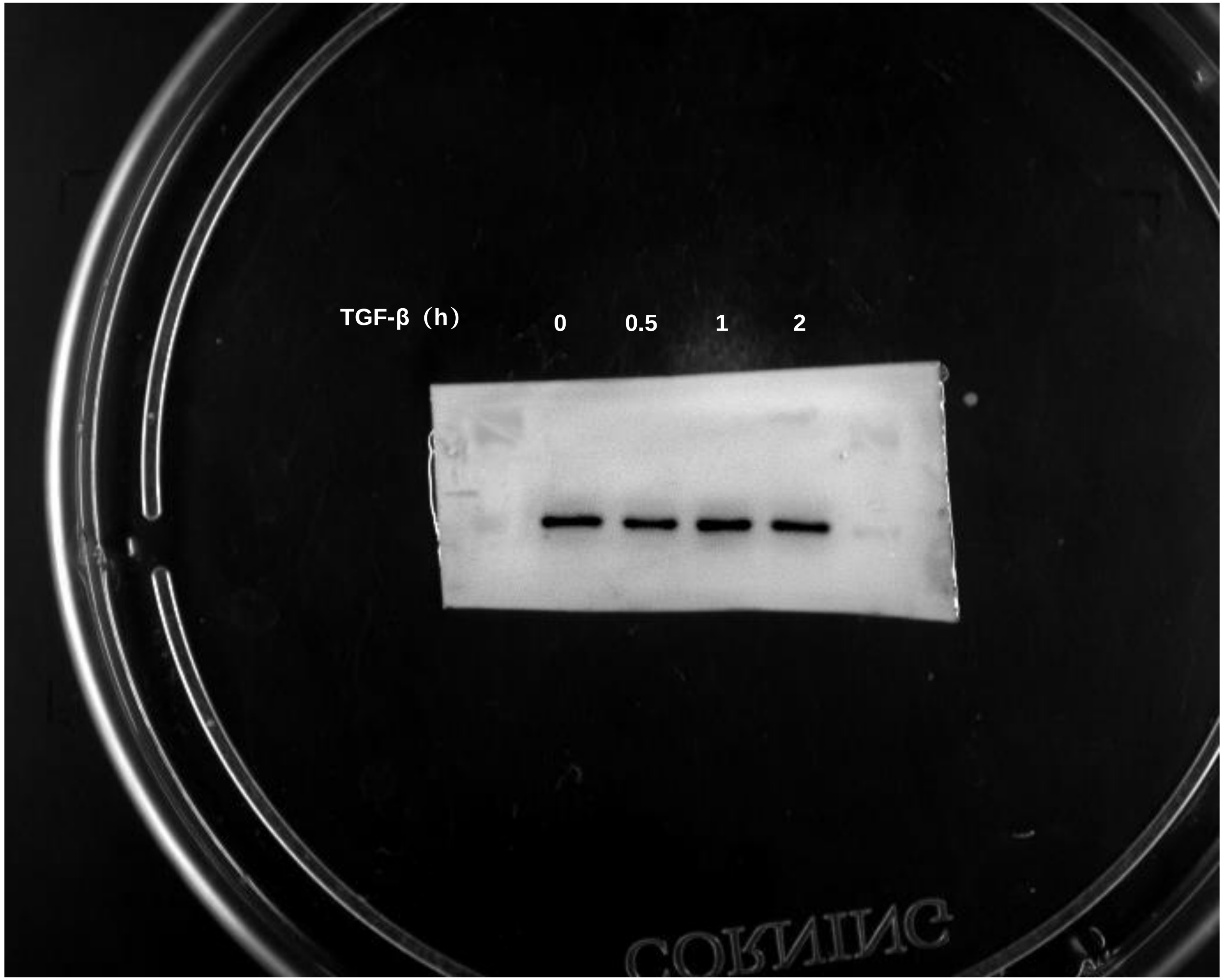

β-actin(45kDa)

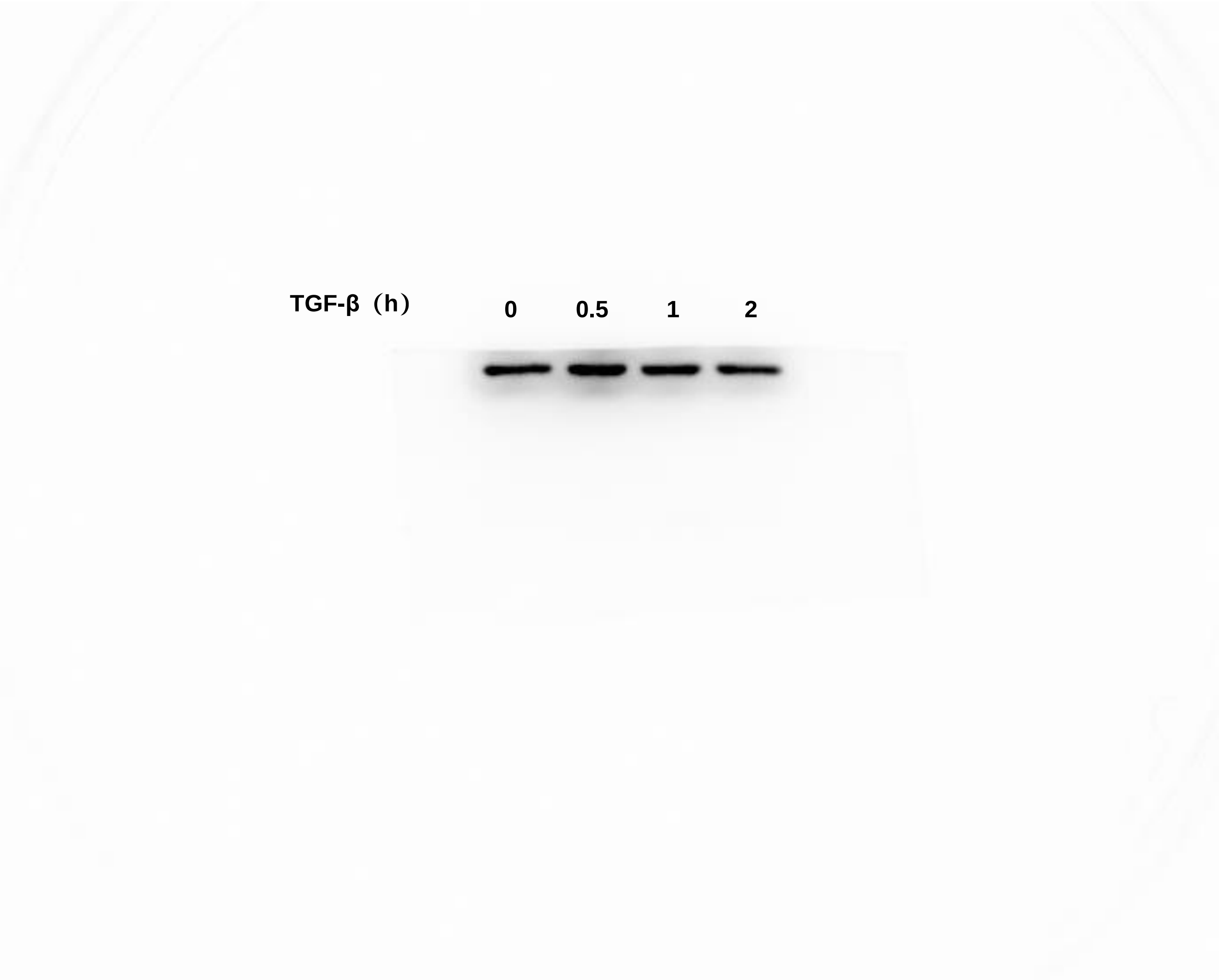

β-actin(45kDa)

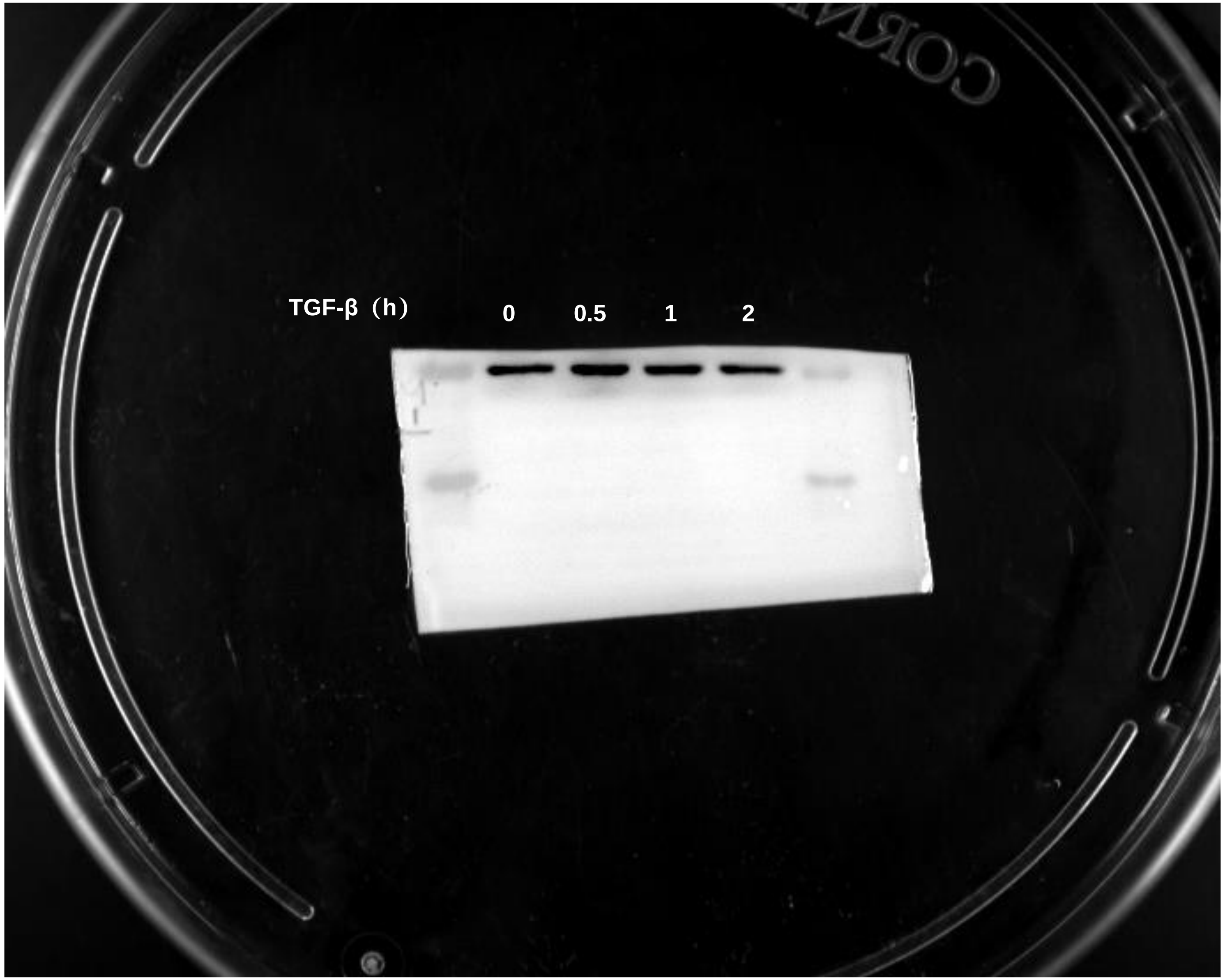

figure4-A-2

P-smad3(50-60kDa)

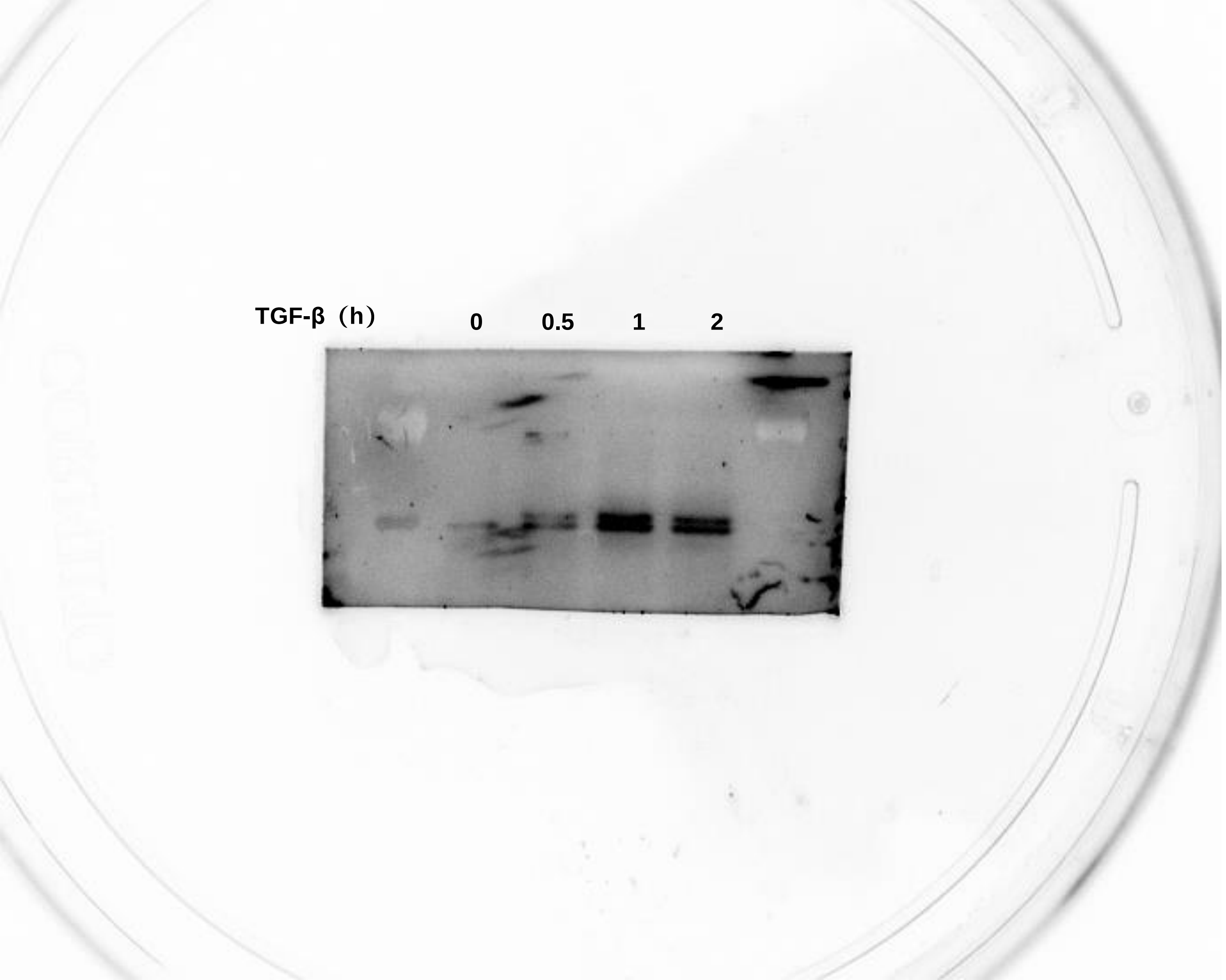

P-smad3(50-60kDa)

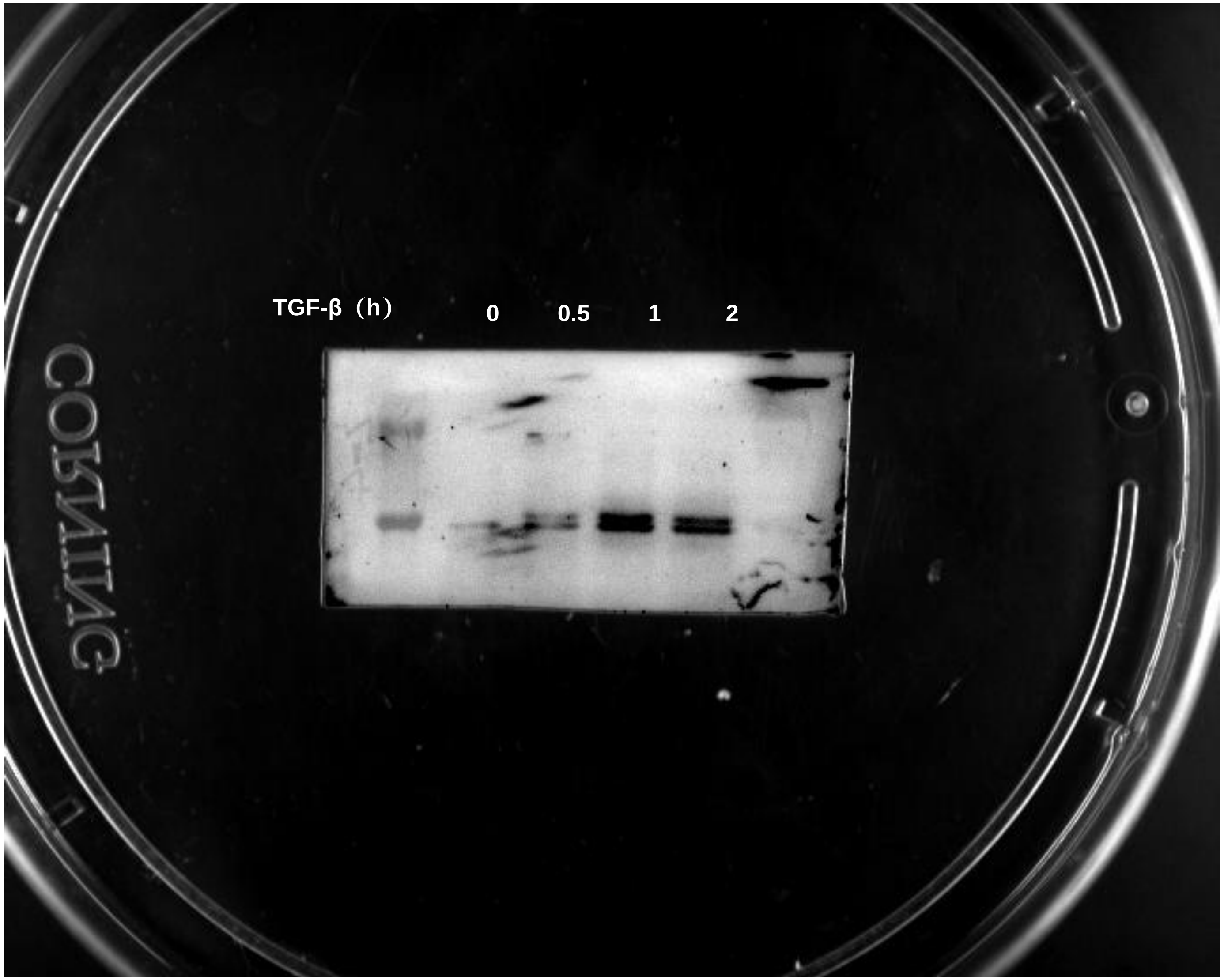

smad3(50-60kDa)

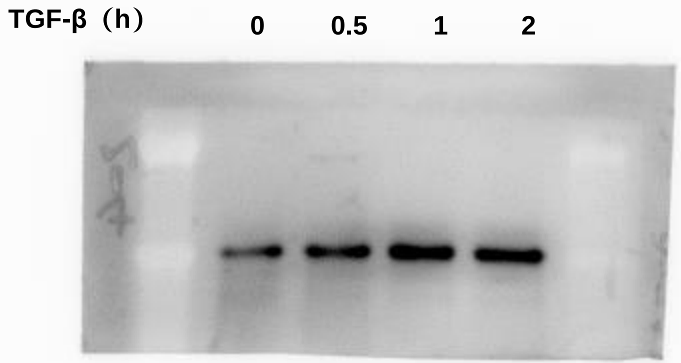

smad3(50-60kDa)

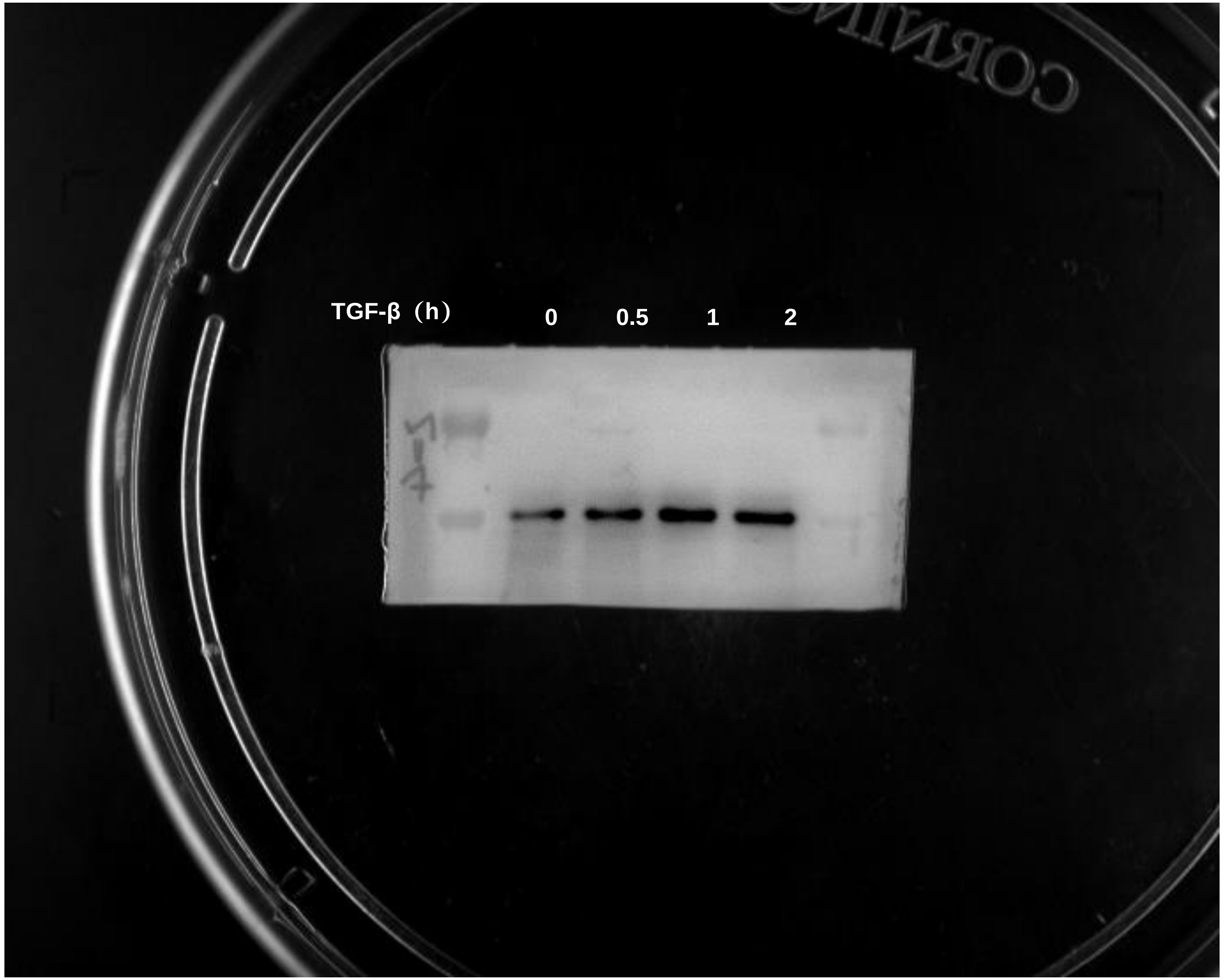

β-actin(45kDa)

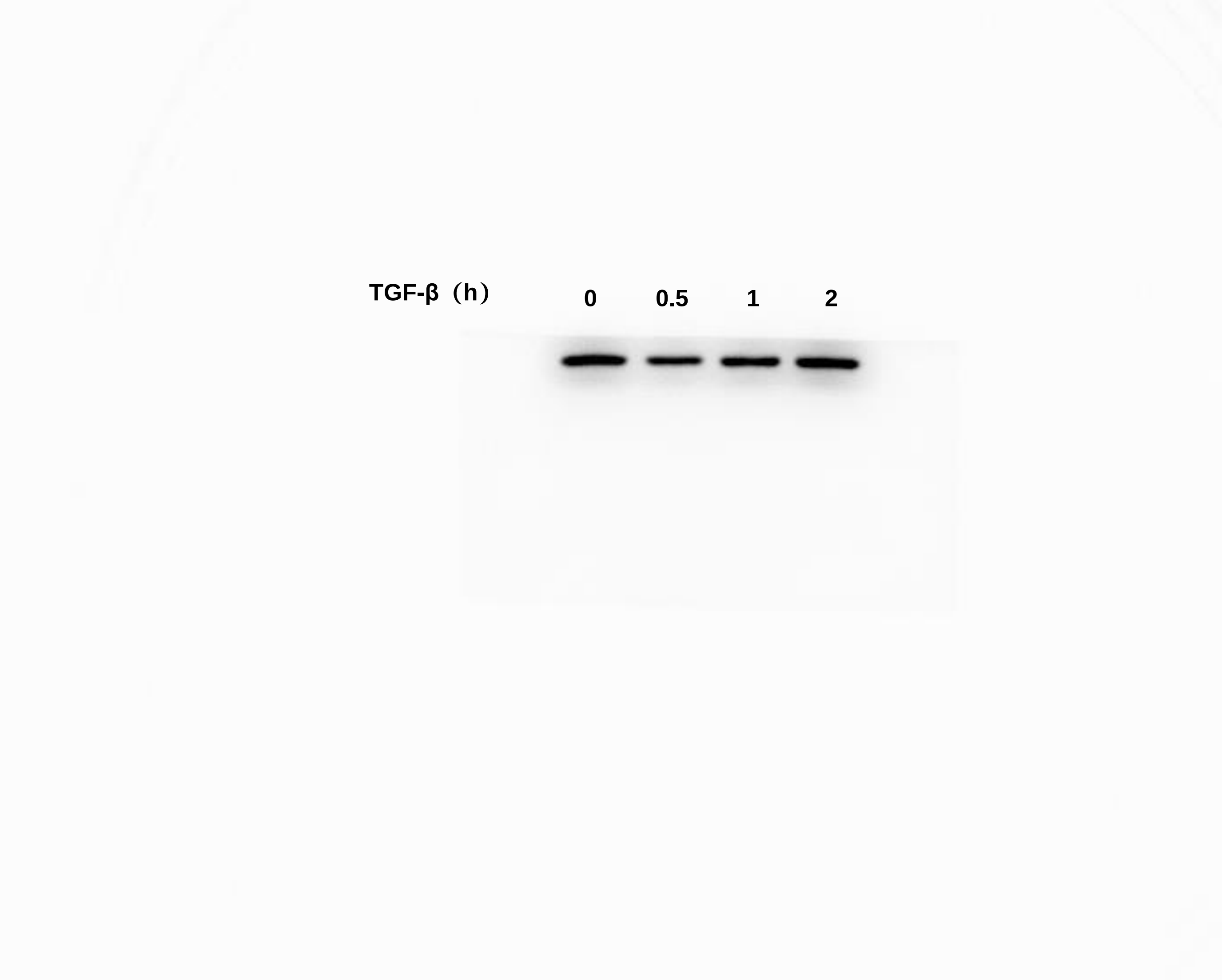

β-actin(45kDa)

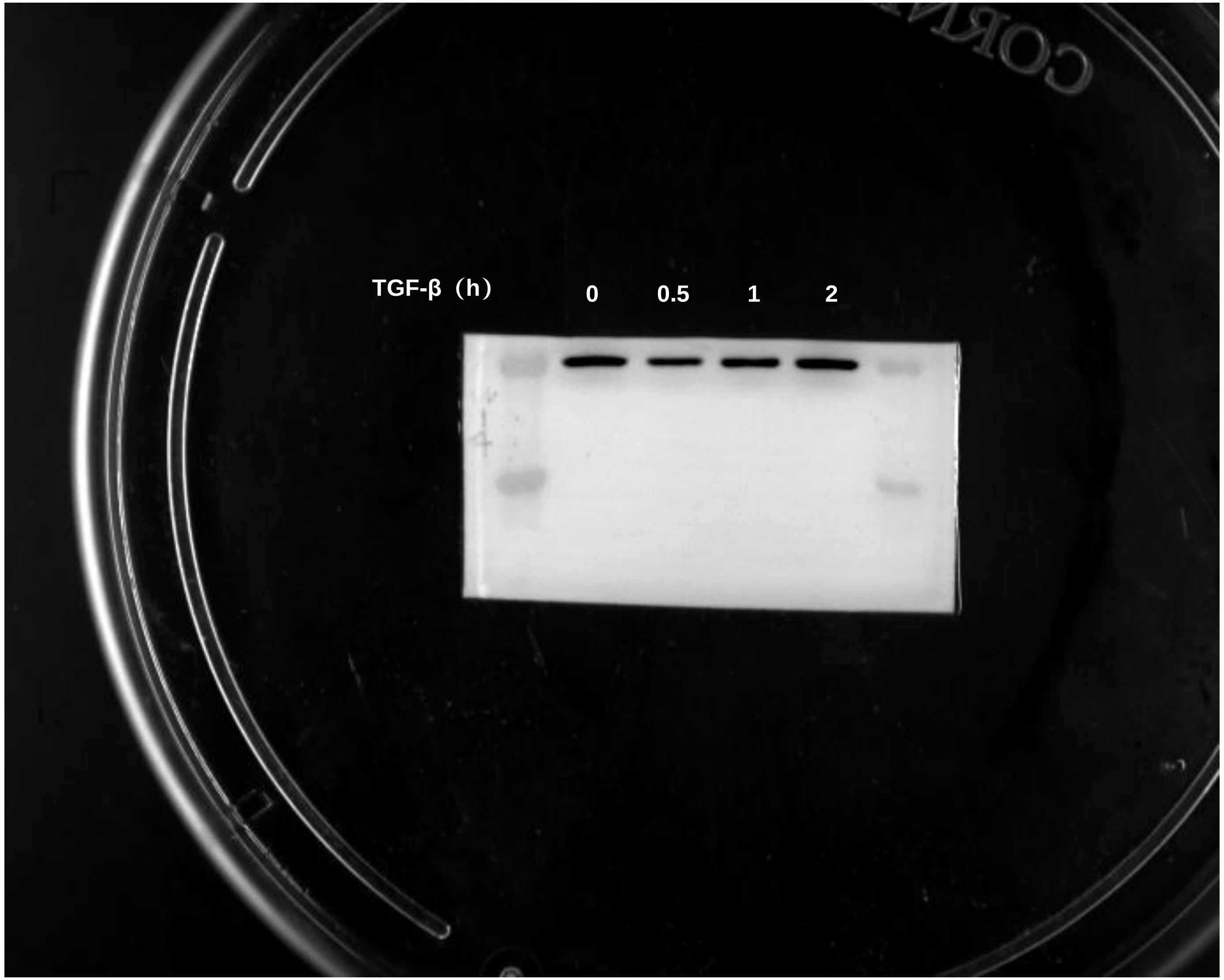

figure4-A-3

**P-smad3(50-60kDa)**

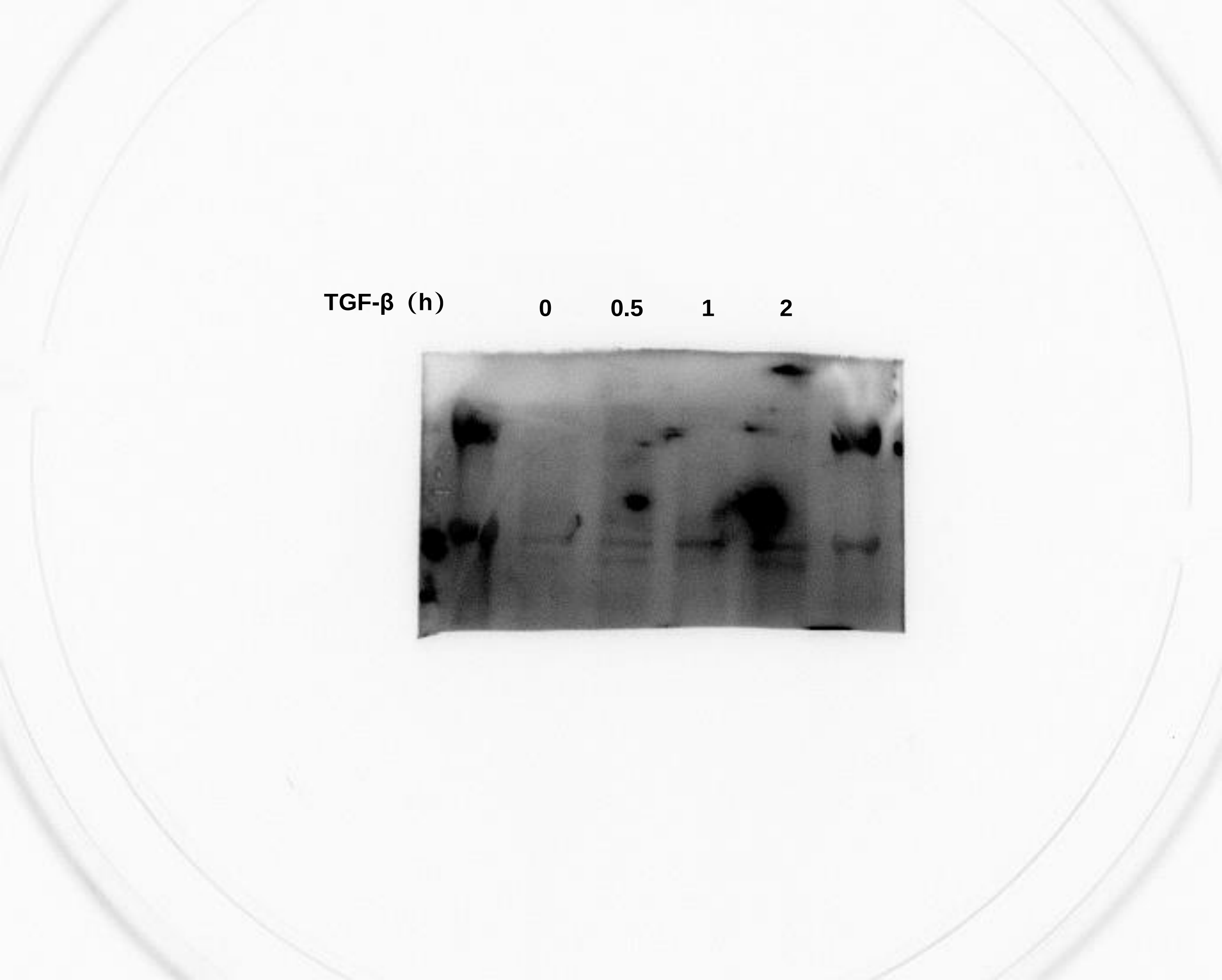

P-smad3(50-60kDa)

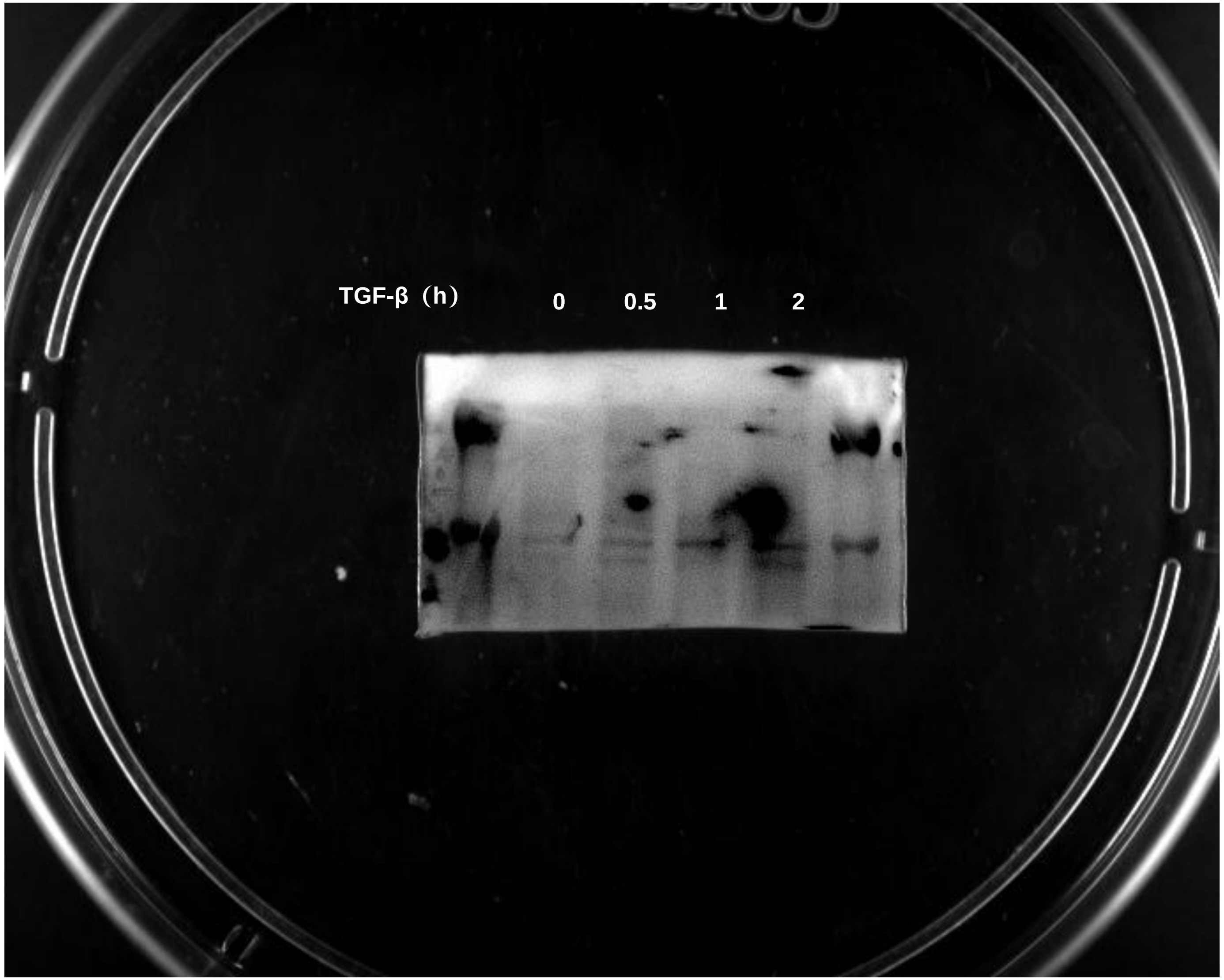

smad3(50-60kDa)

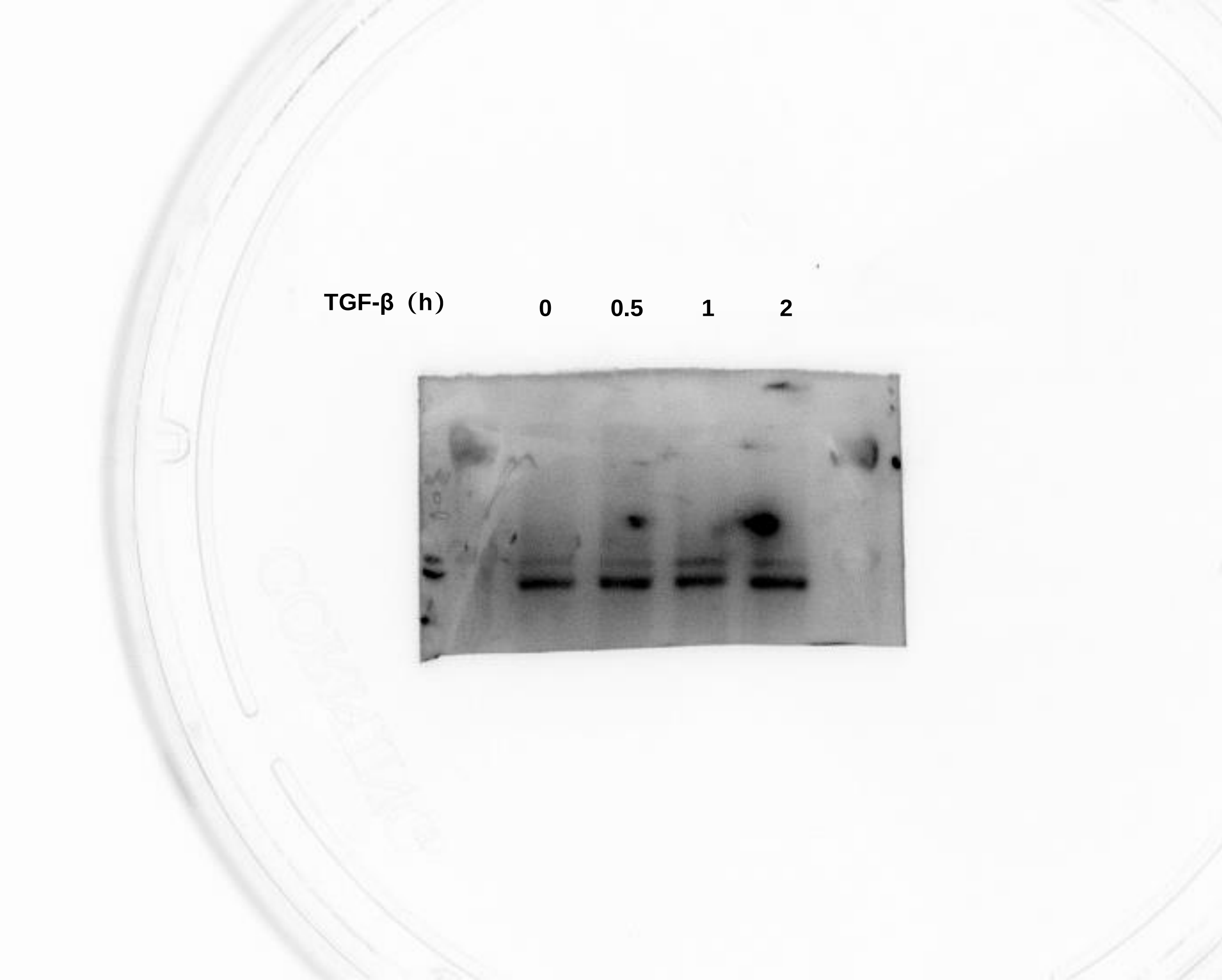

smad3(50-60kDa)

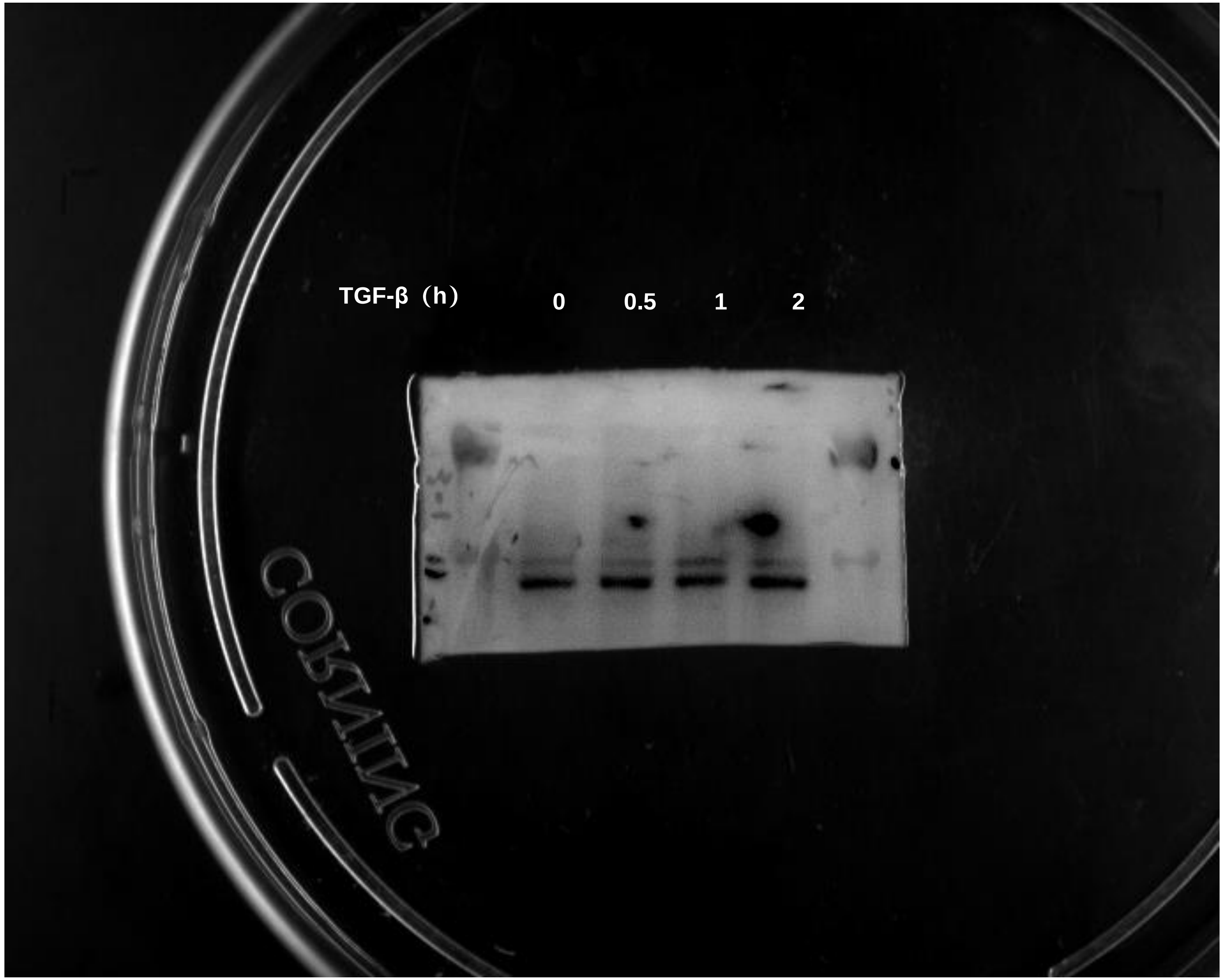

β-actin(45kDa)

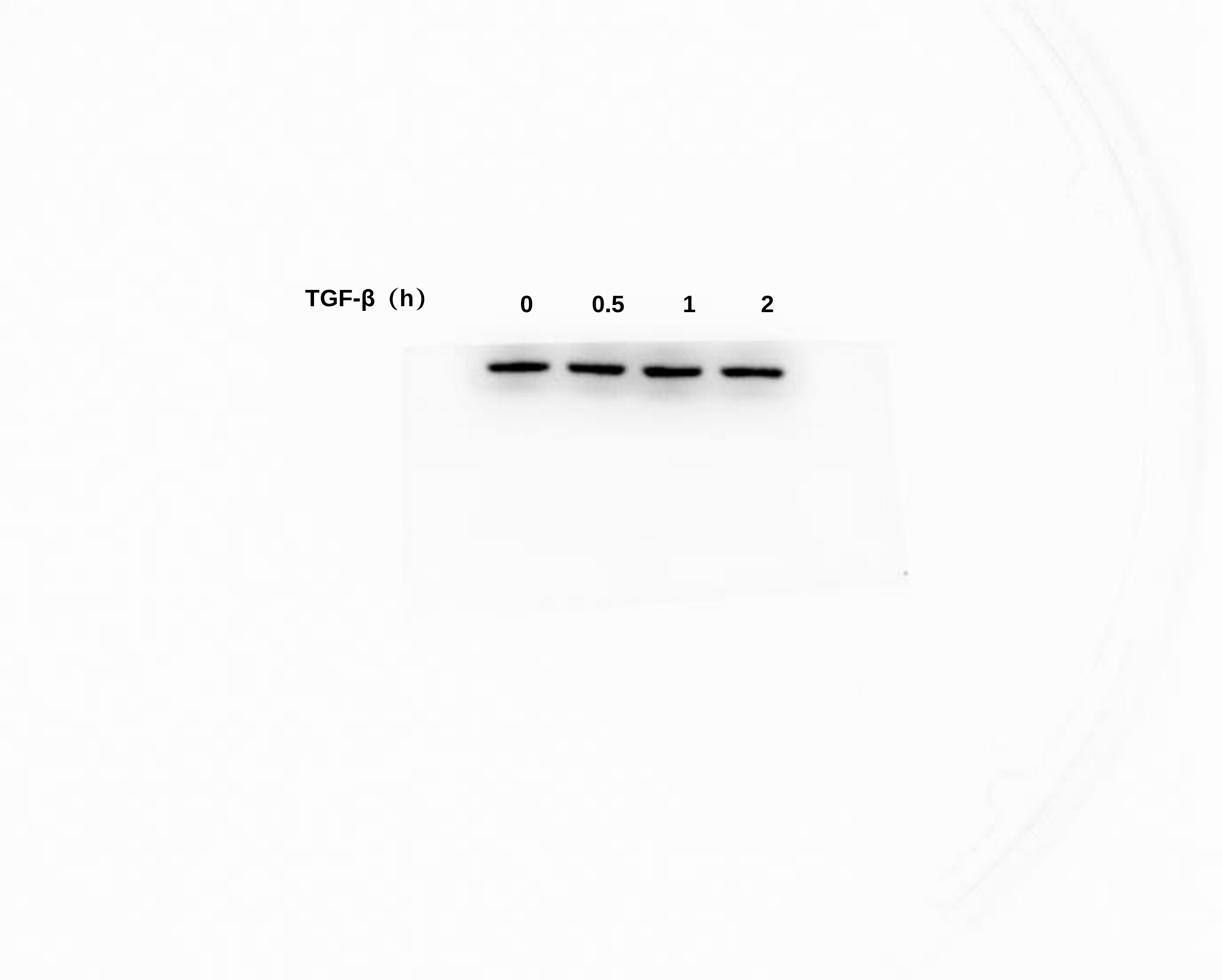

β-actin(45kDa)

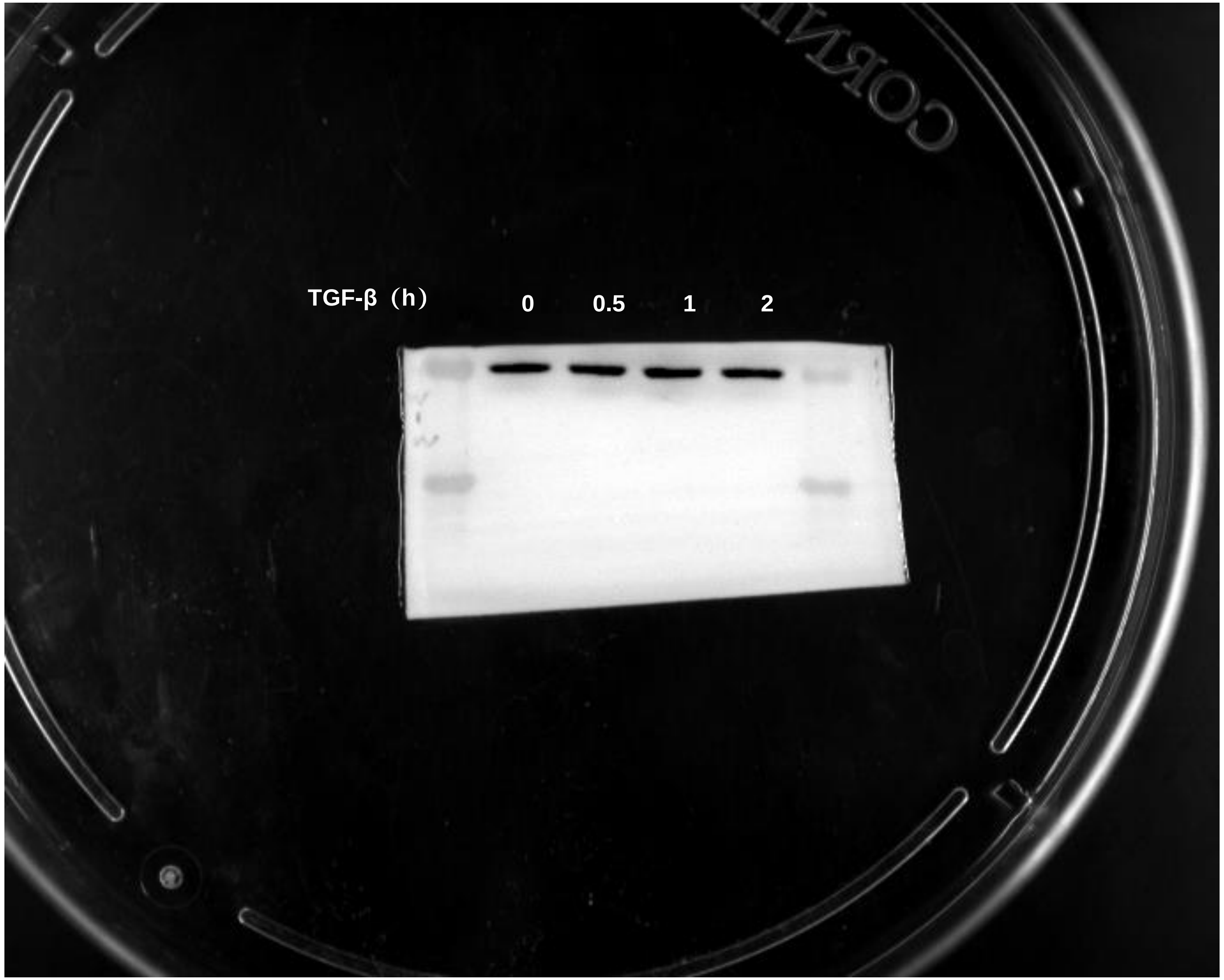

figure4-B-1

**P-smad3(50-60kDa)**

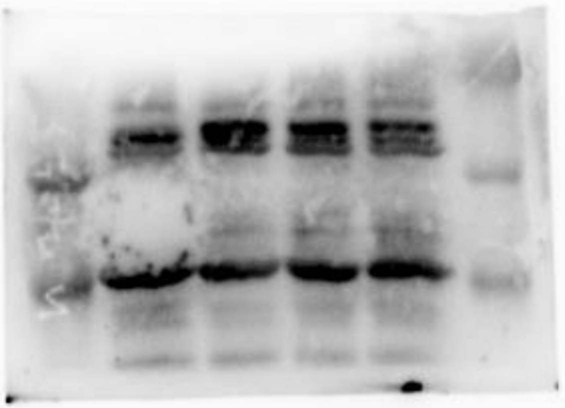

| | | | | |
|---|---|---|---|---|
| TGF-β | - | + | + | + |
| vetor | - | - | + | - |
| IFITM1 | - | - | - | + |

P-smad3(50-60kDa)

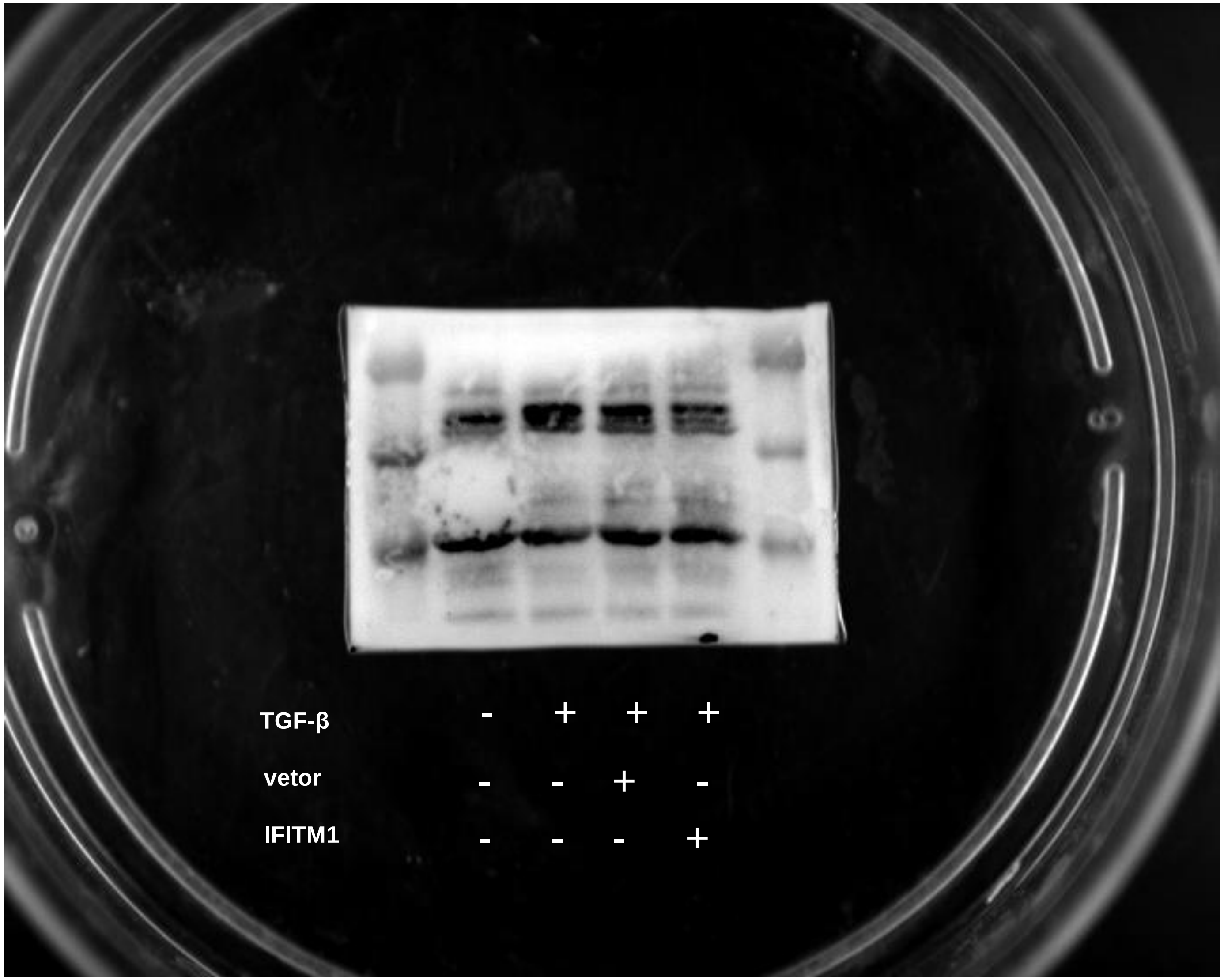

smad3(50-60kDa)

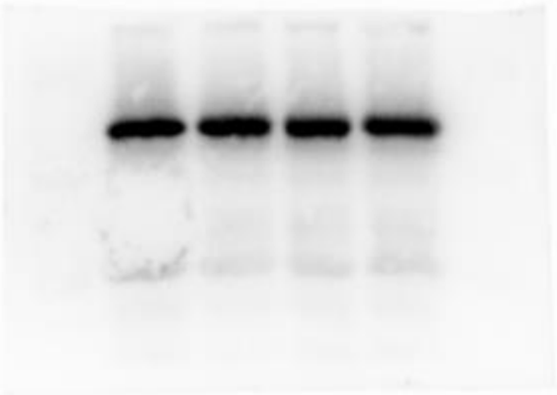

| | | | | |
|---|---|---|---|---|
| TGF-β | - | + | + | + |
| vetor | - | - | + | - |
| IFITM1 | - | - | - | + |

smad3(50-60kDa)

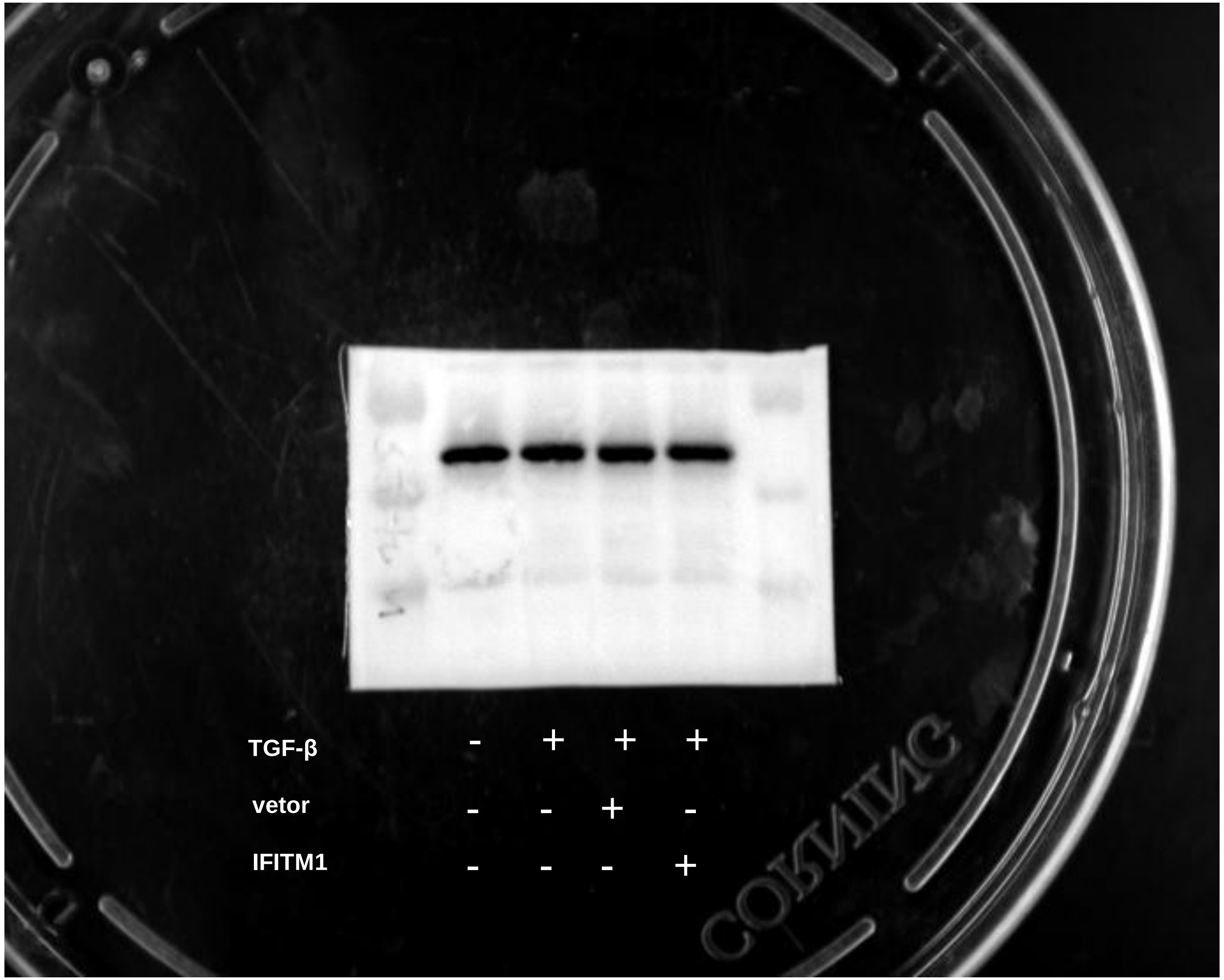

β-actin(45kDa)

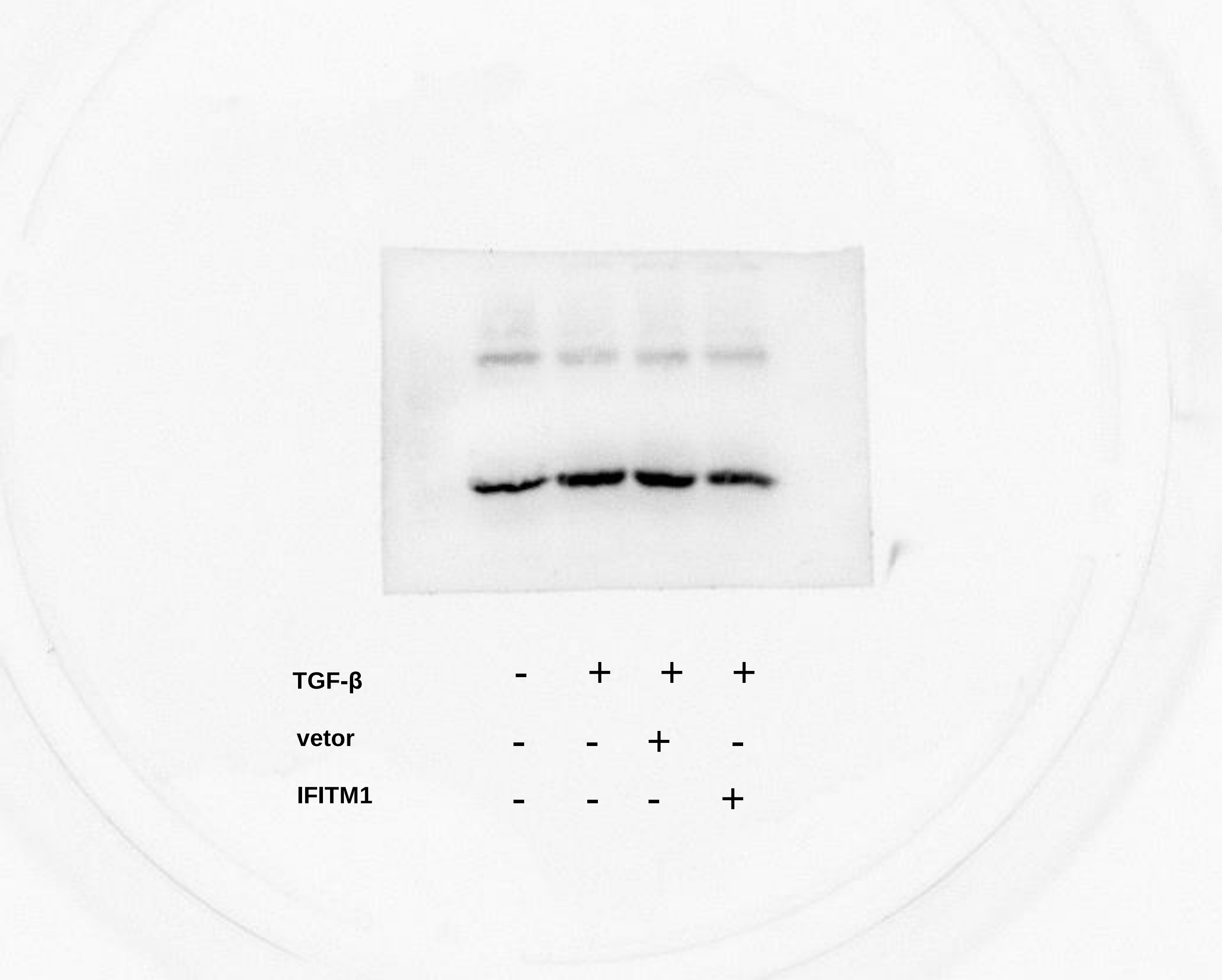

β-actin(45kDa)

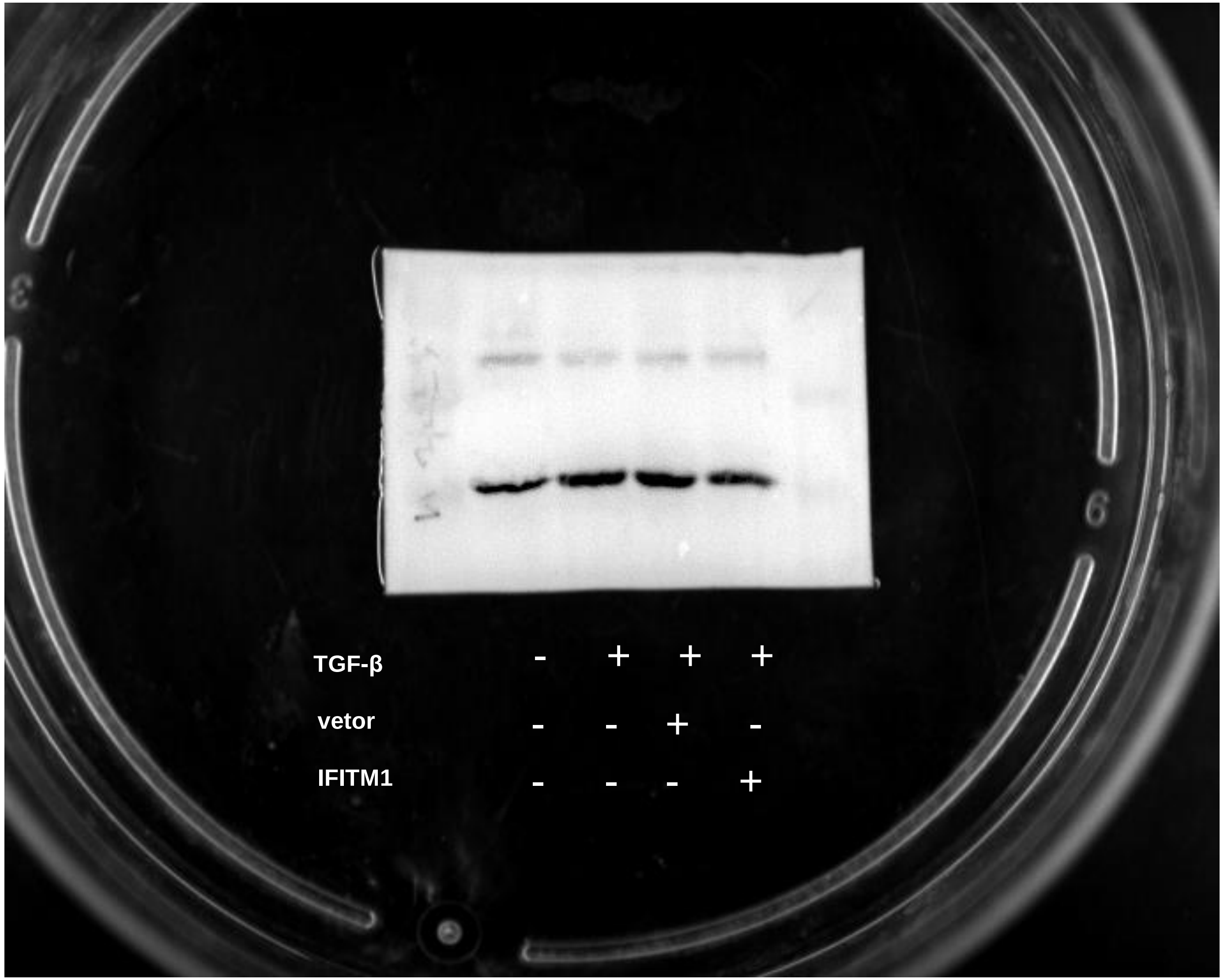

figure4-B-2

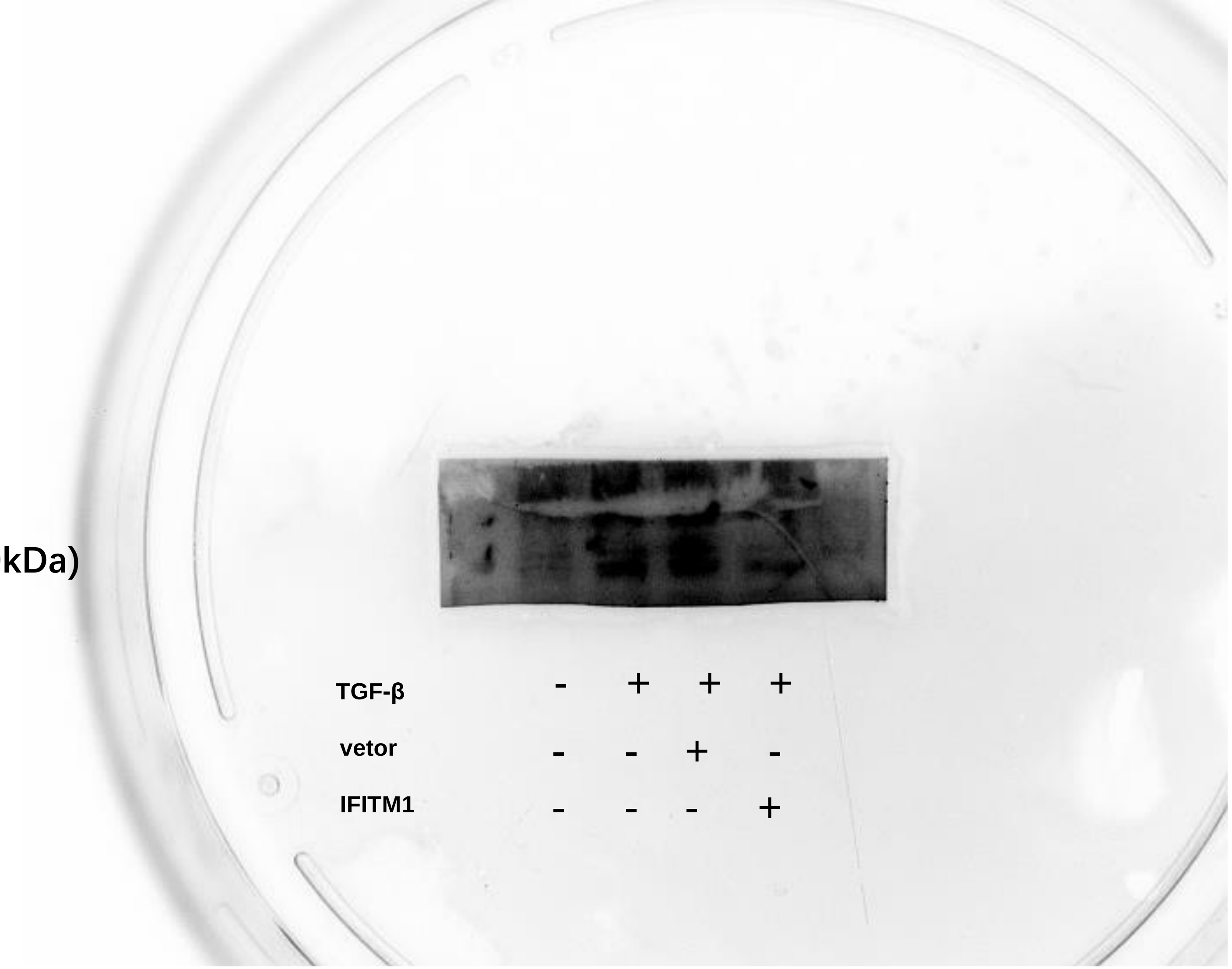

P-smad3(50-60kDa)

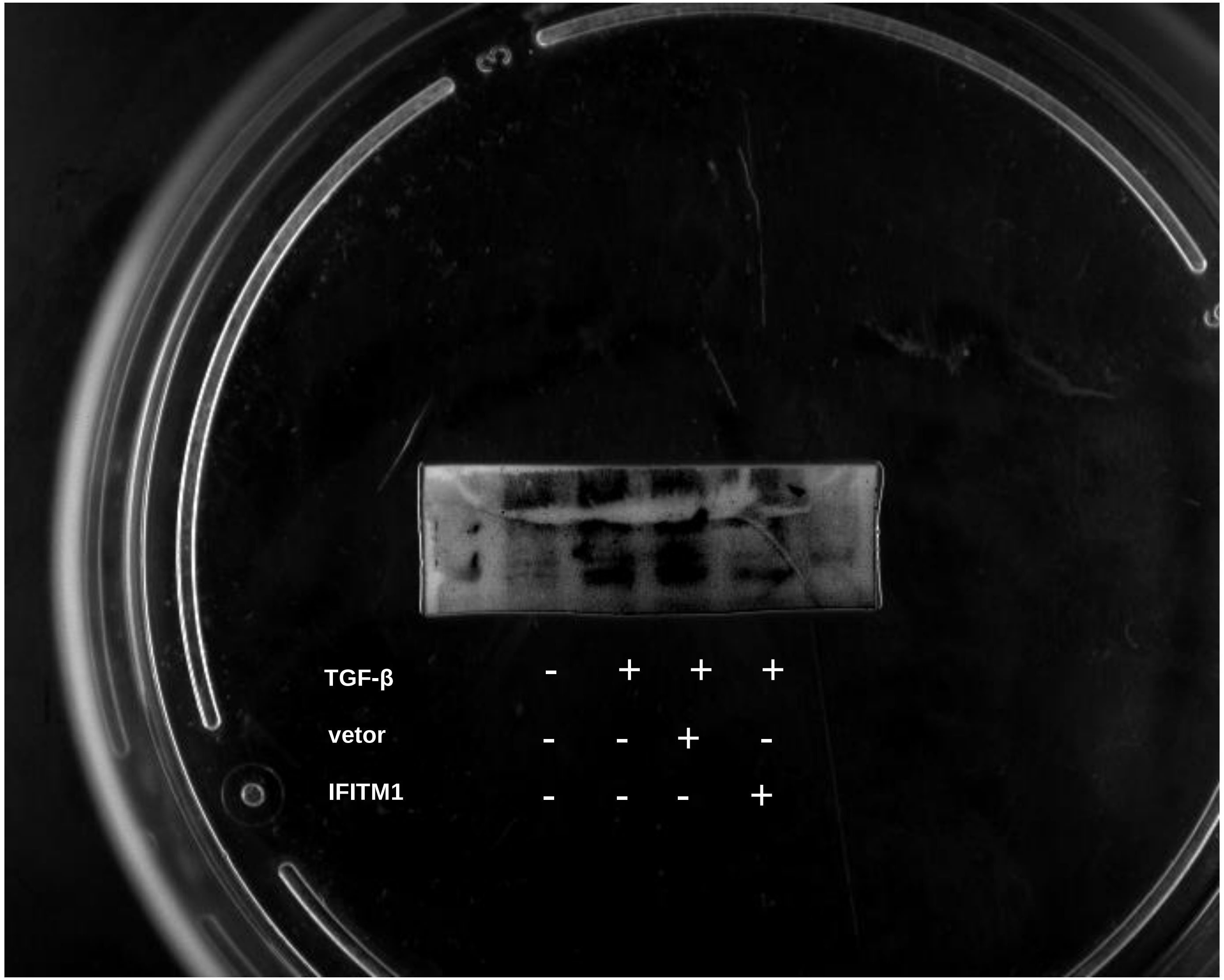

smad3(50-60kDa)

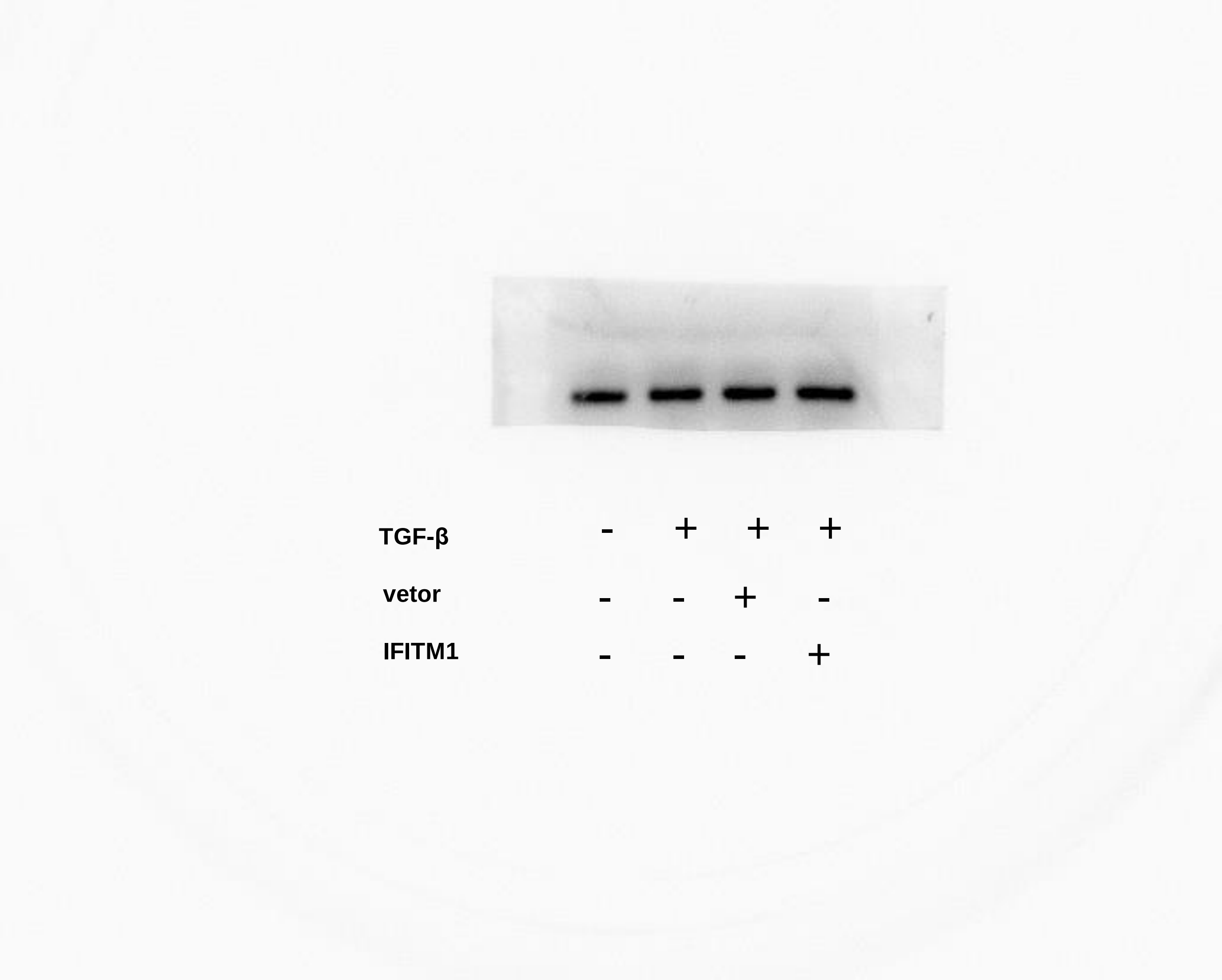

smad3(50-60kDa)

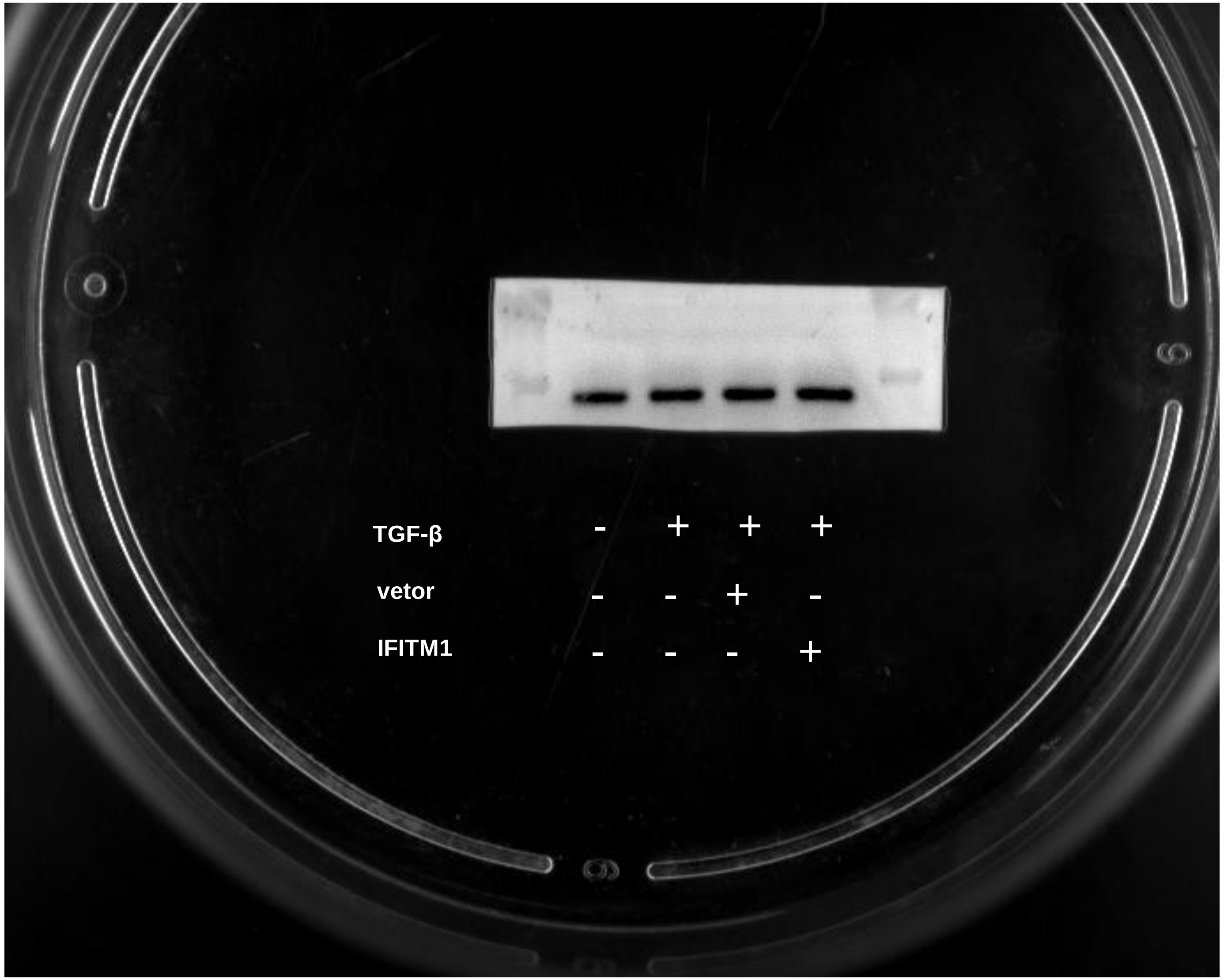

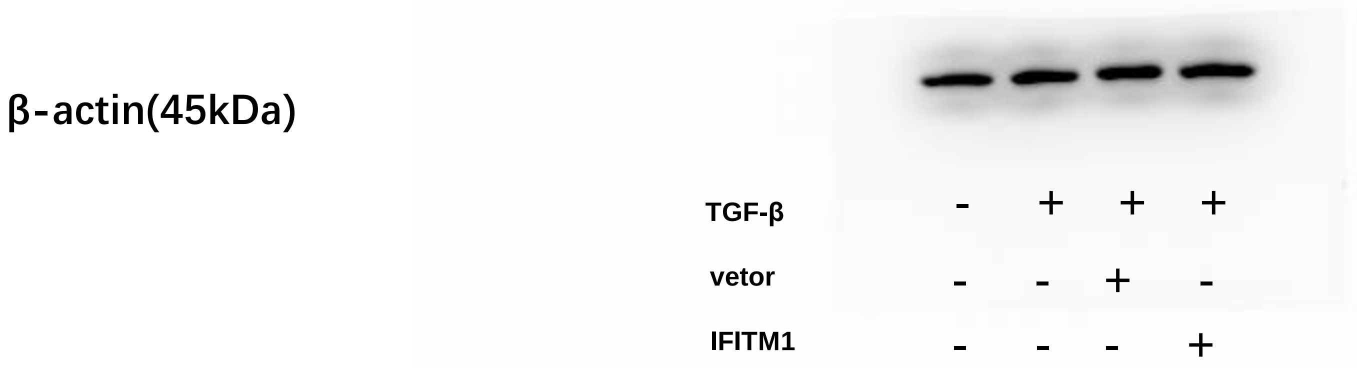
β-actin(45kDa)
TGF-β - + + +
vetor - - + -
IFITM1 - - - +

β-actin(45kDa)

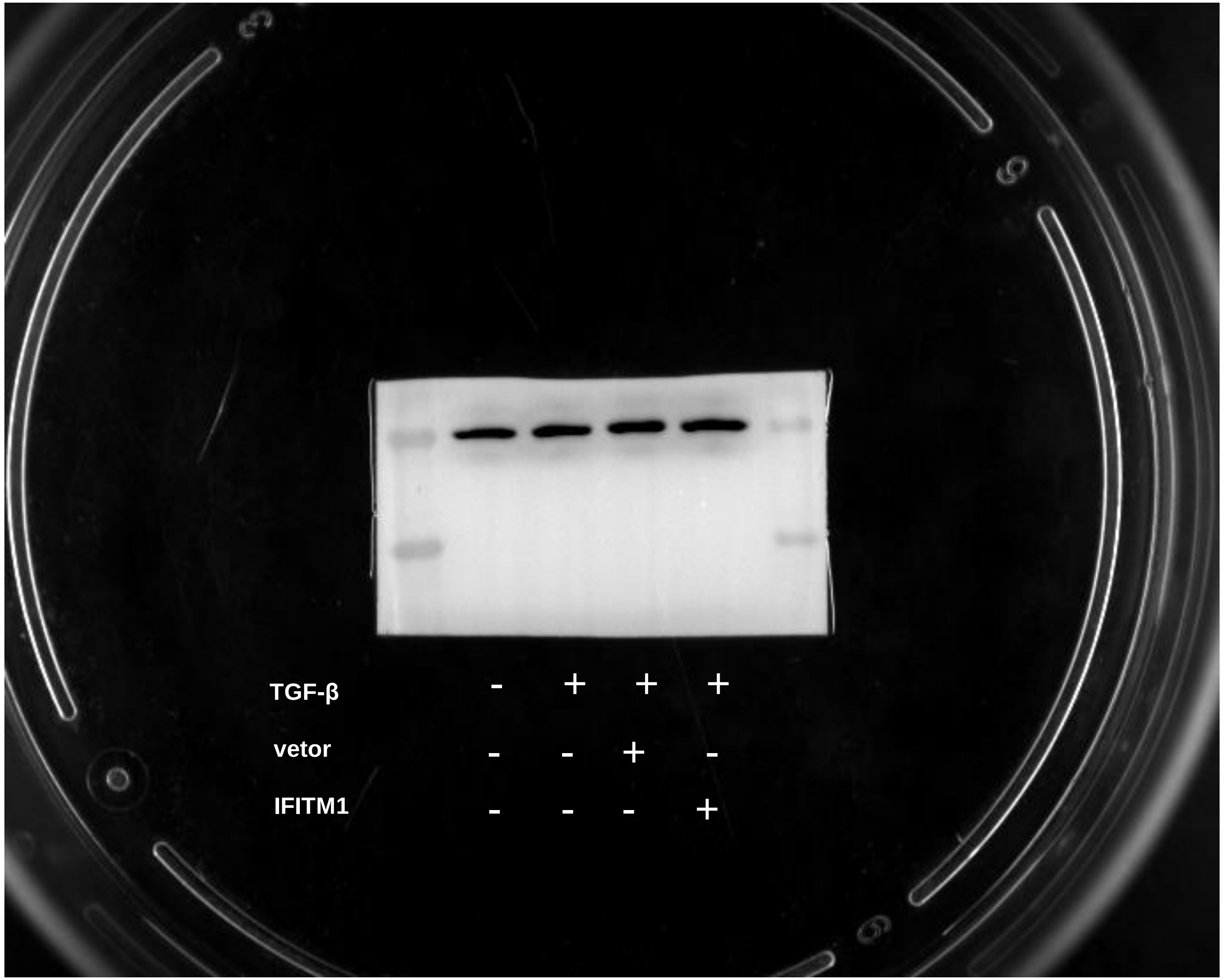

figure4-B-3

P-smad3(50-60kDa)

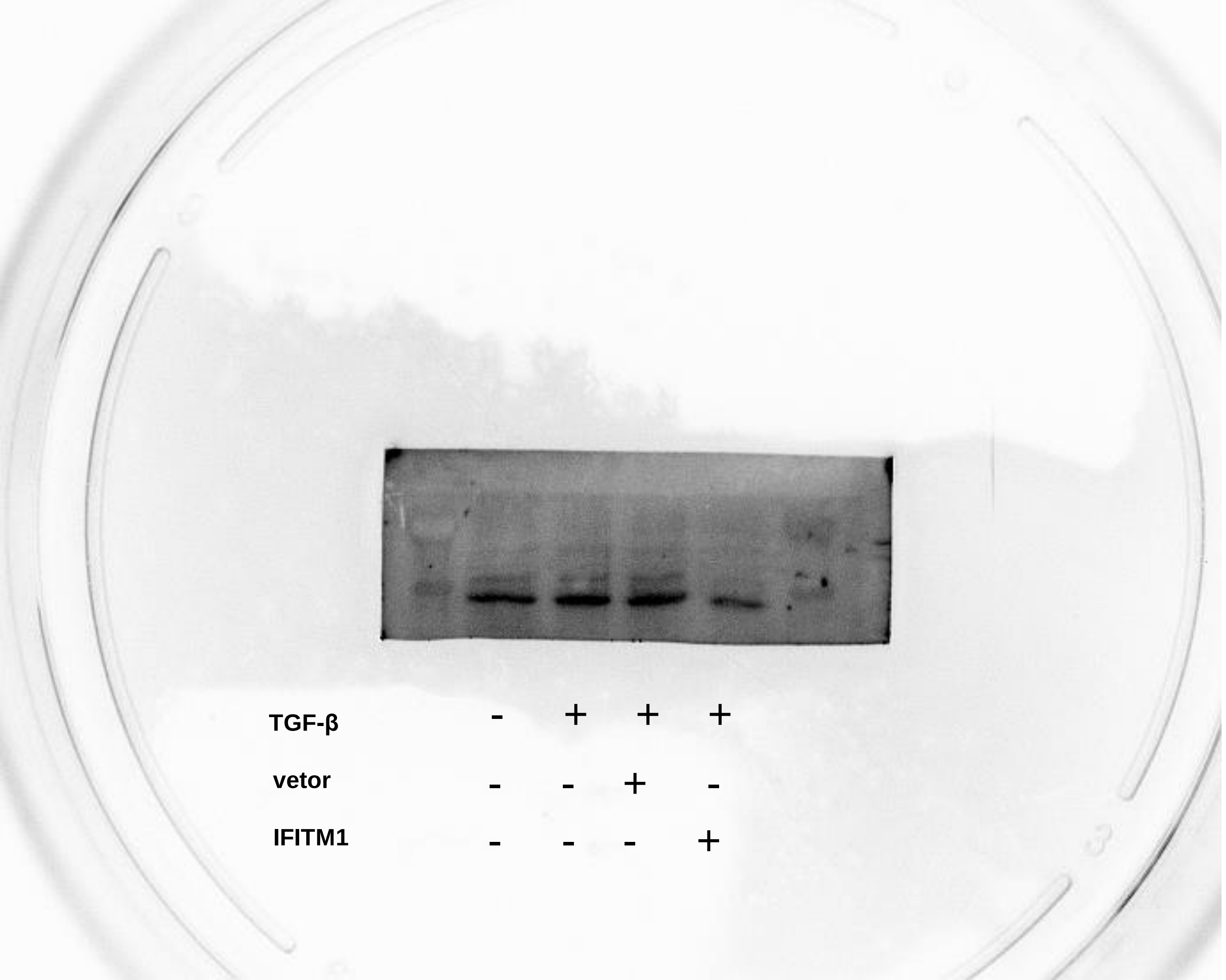

P-smad3(50-60kDa)

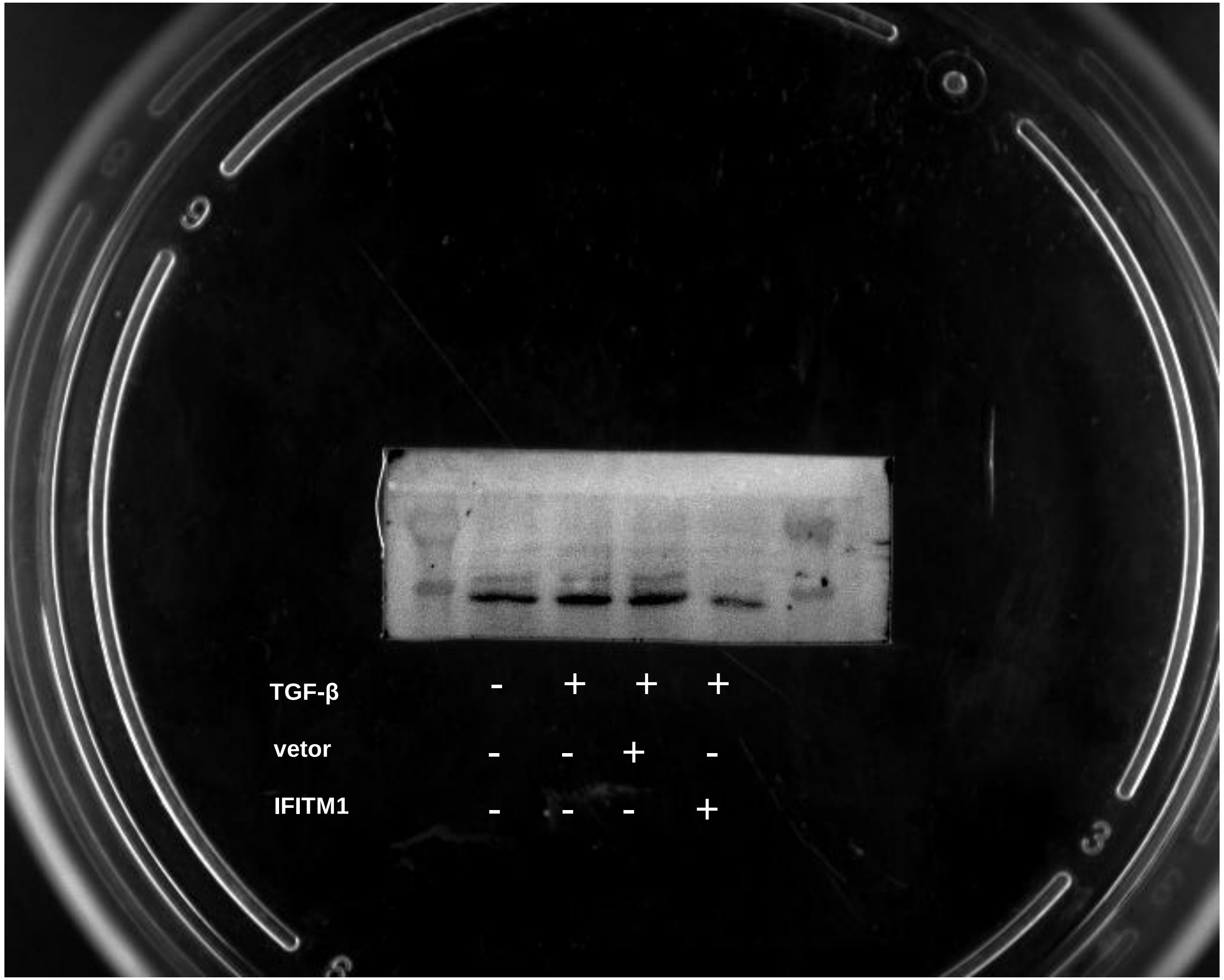

smad3(50-60kDa)

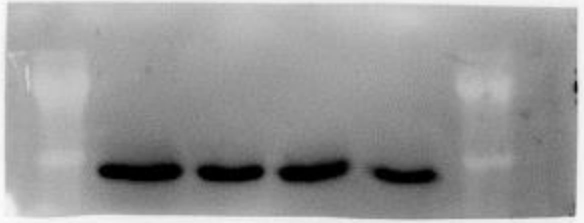

| | | | | |
|---|---|---|---|---|
| TGF-β | - | + | + | + |
| vetor | - | - | + | - |
| IFITM1 | - | - | - | + |

smad3(50-60kDa)

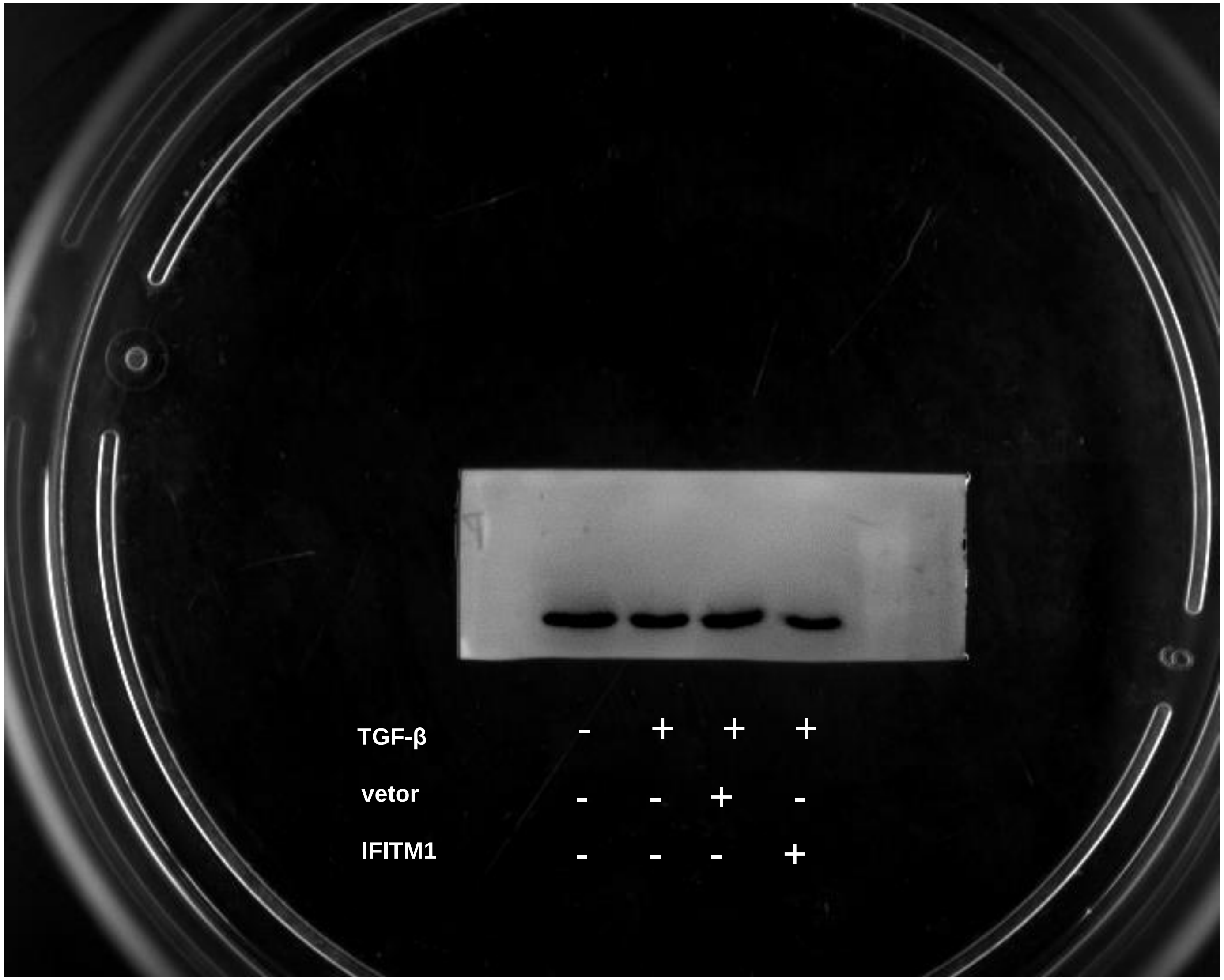

β-actin(45kDa)

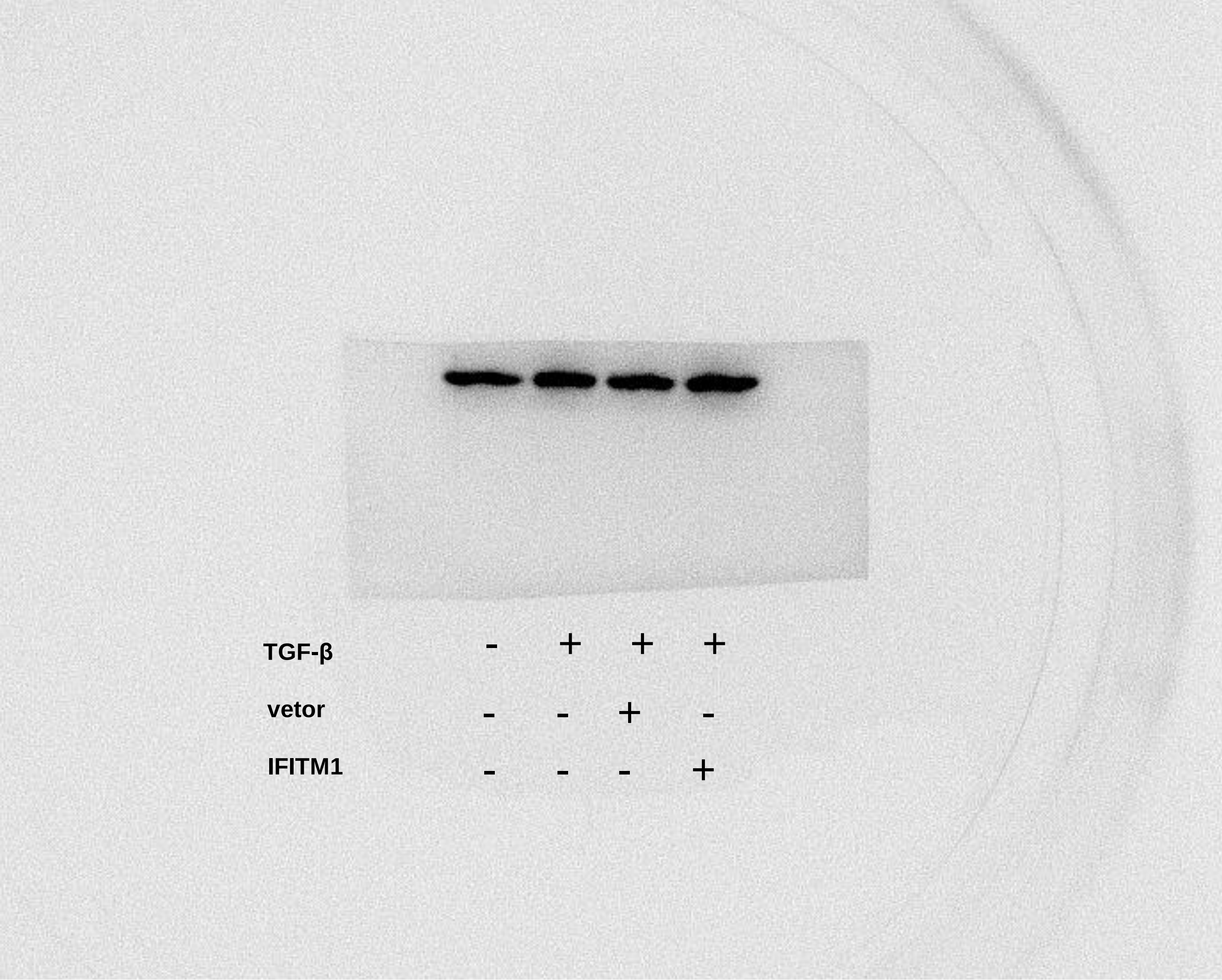

β-actin(45kDa)

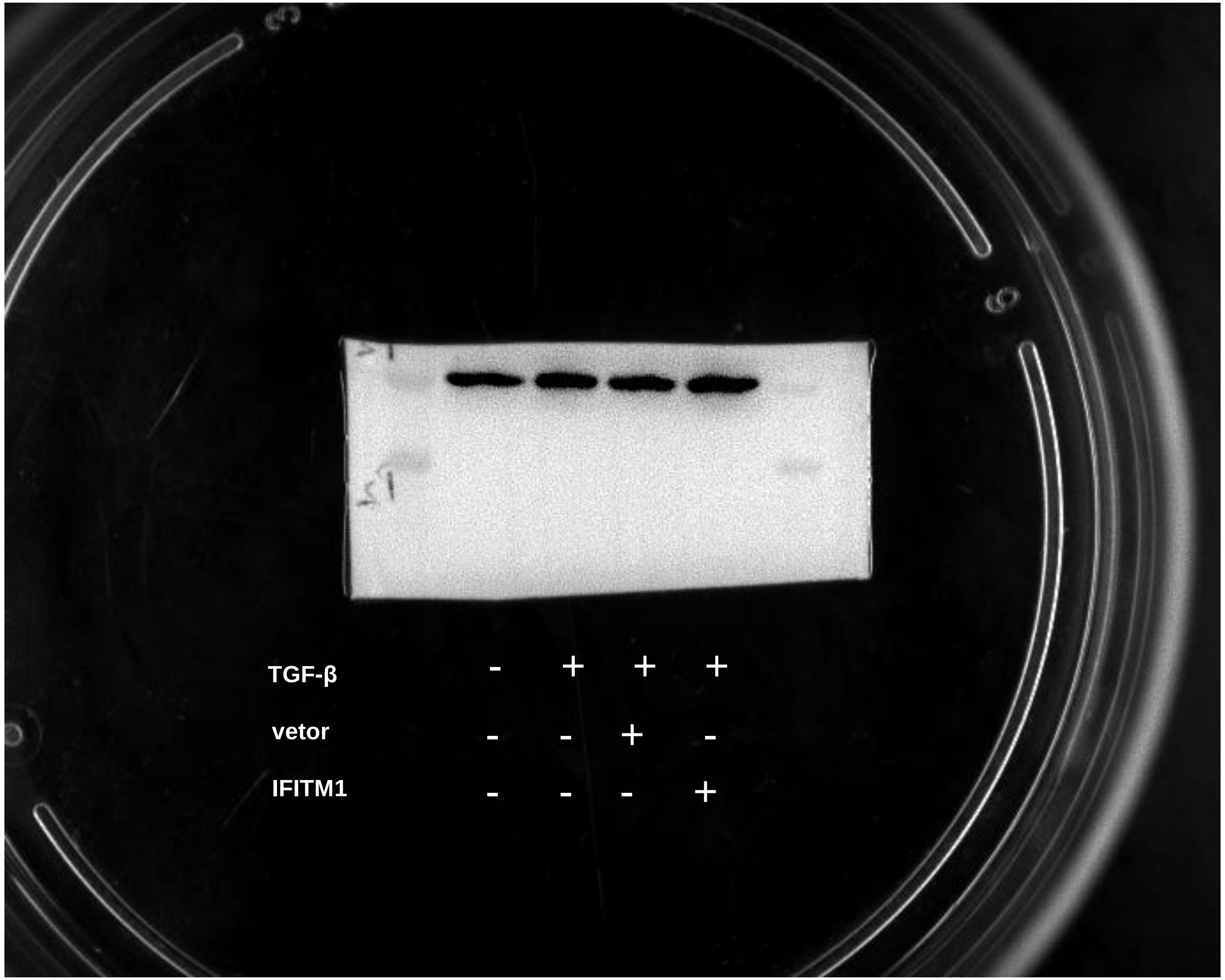

figure5

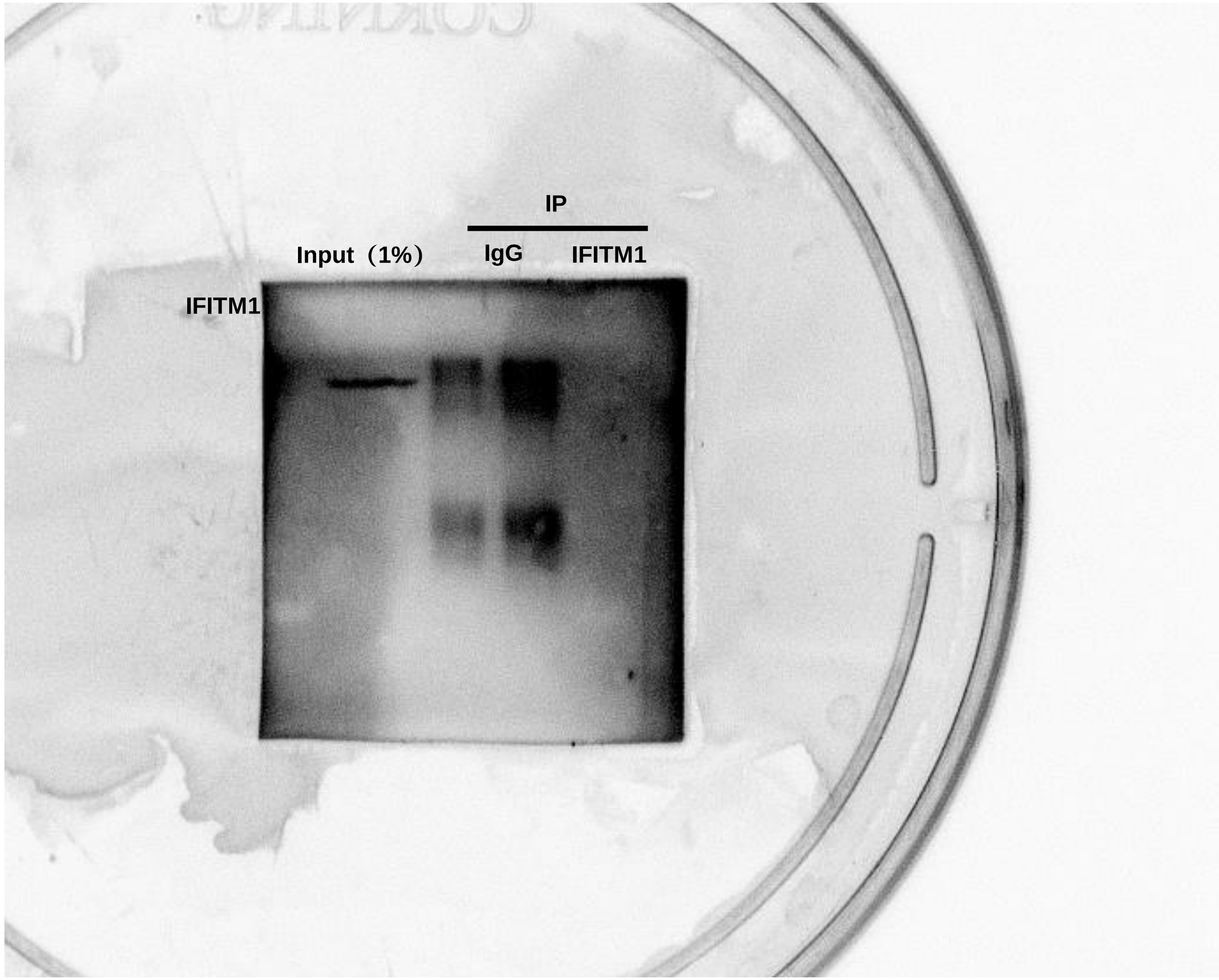

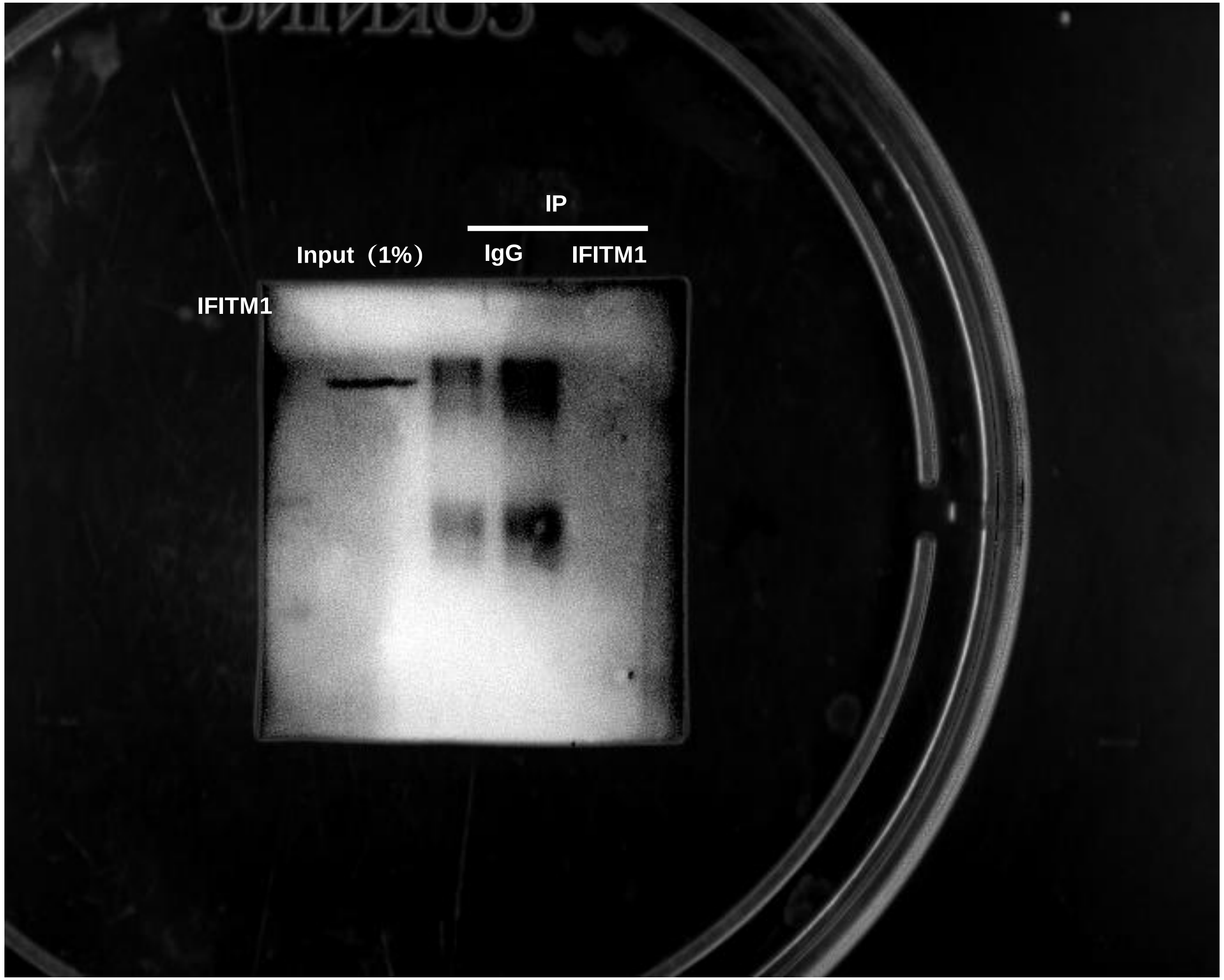
IP
Input（1%）
IgG
IFITM1
IFITM1

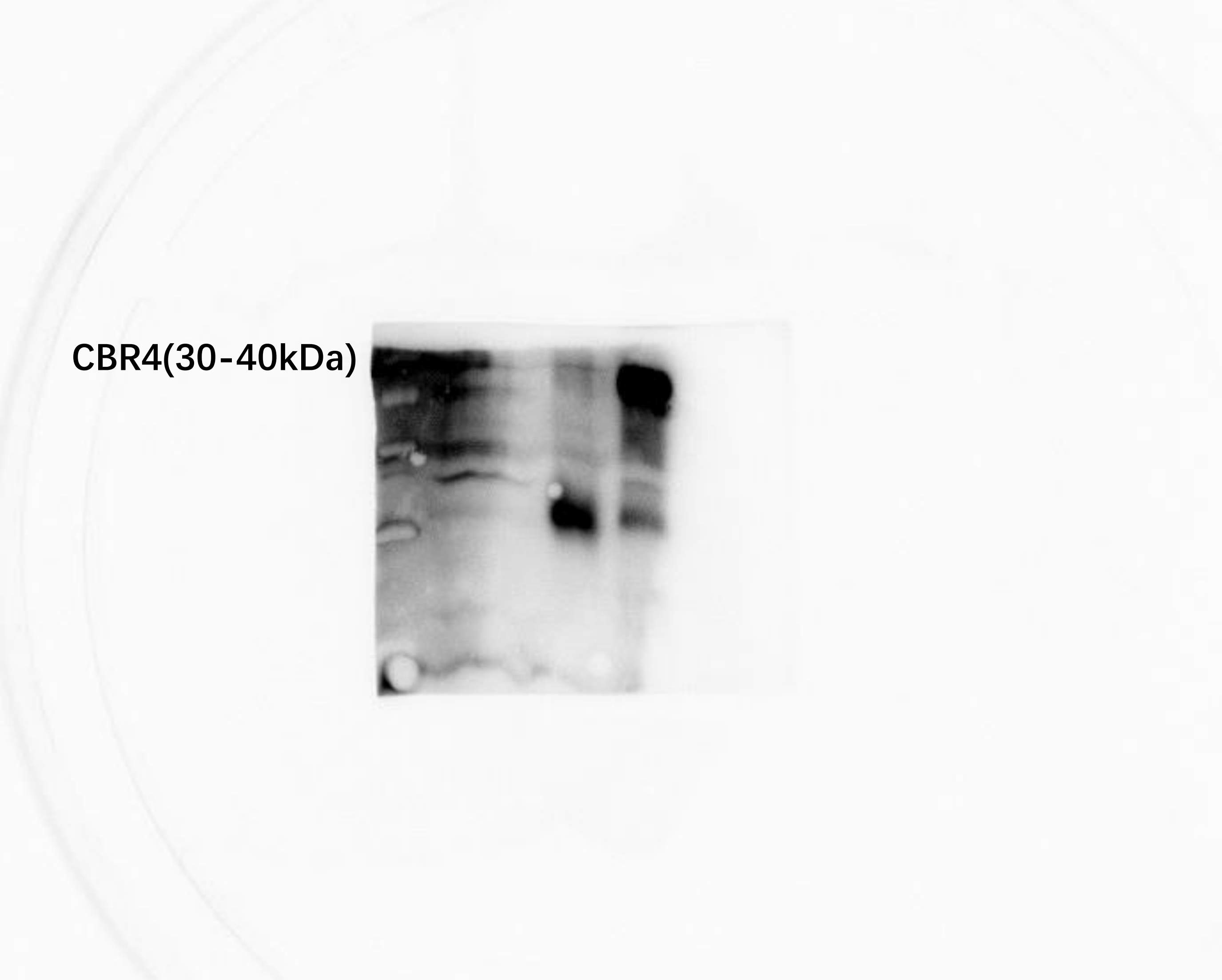
CBR4(30-40kDa)

CBR4(30-40kDa)

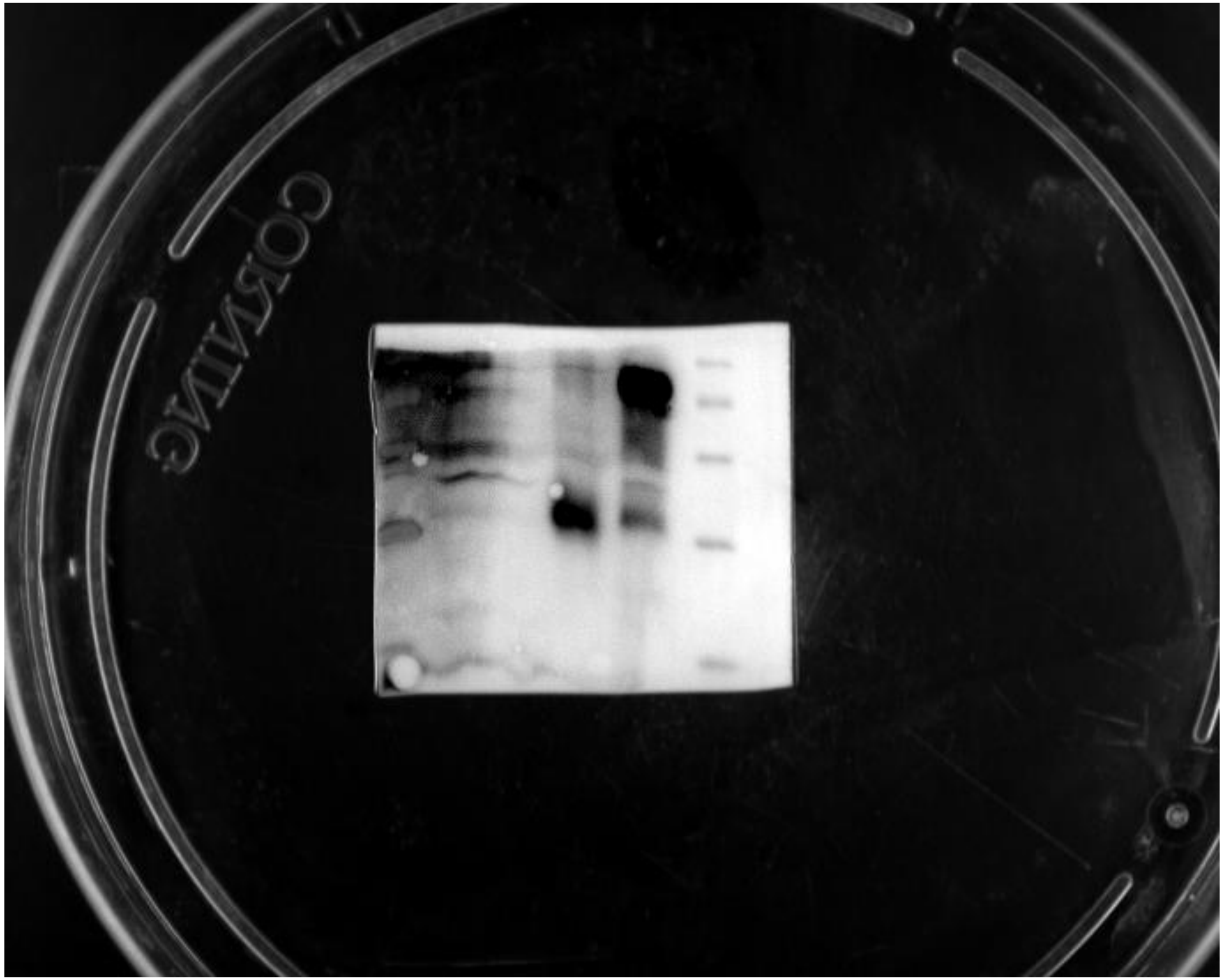

figure6-E

CBR4(30-40kDa)

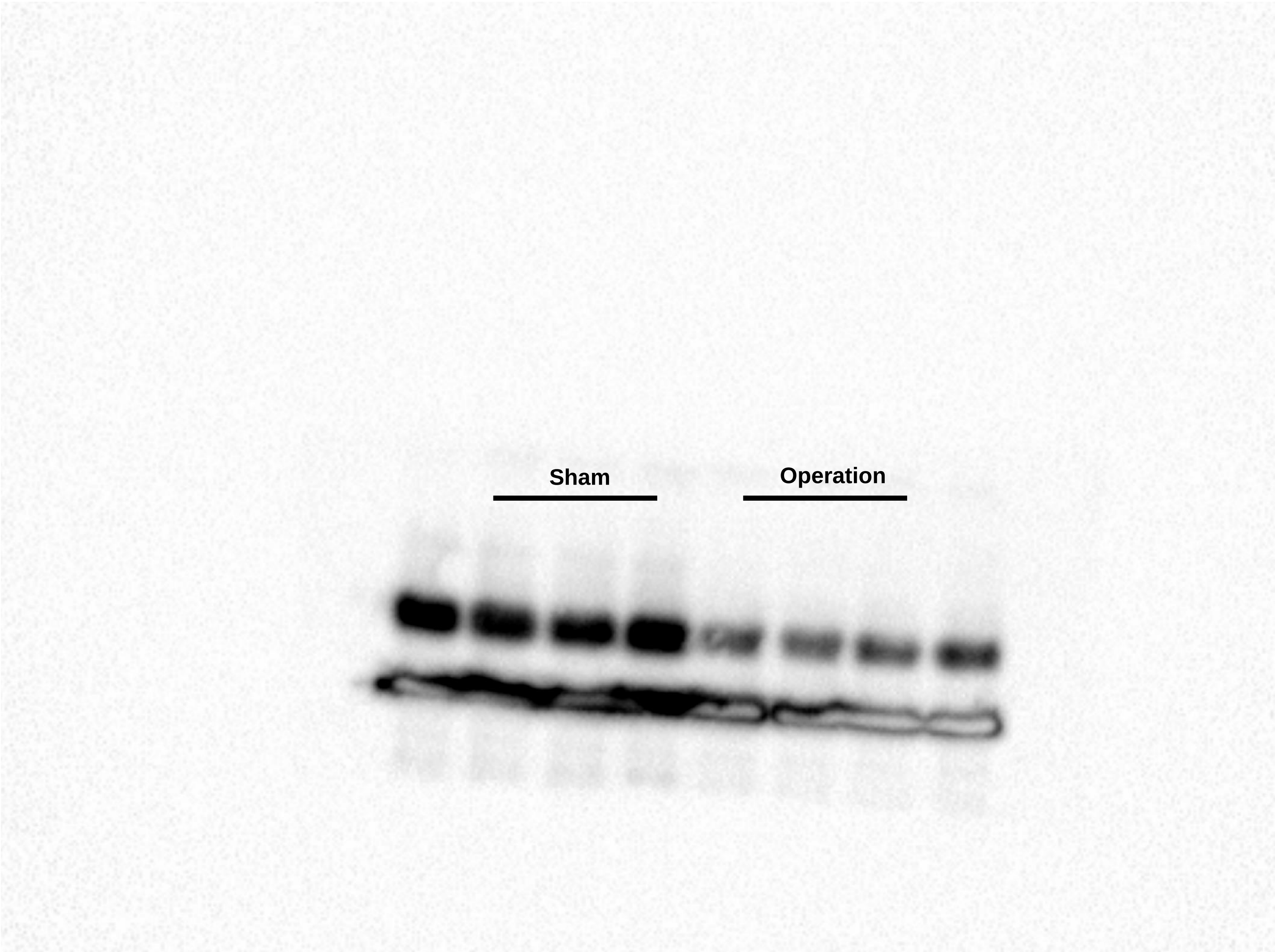

CBR4(30-40kDa)

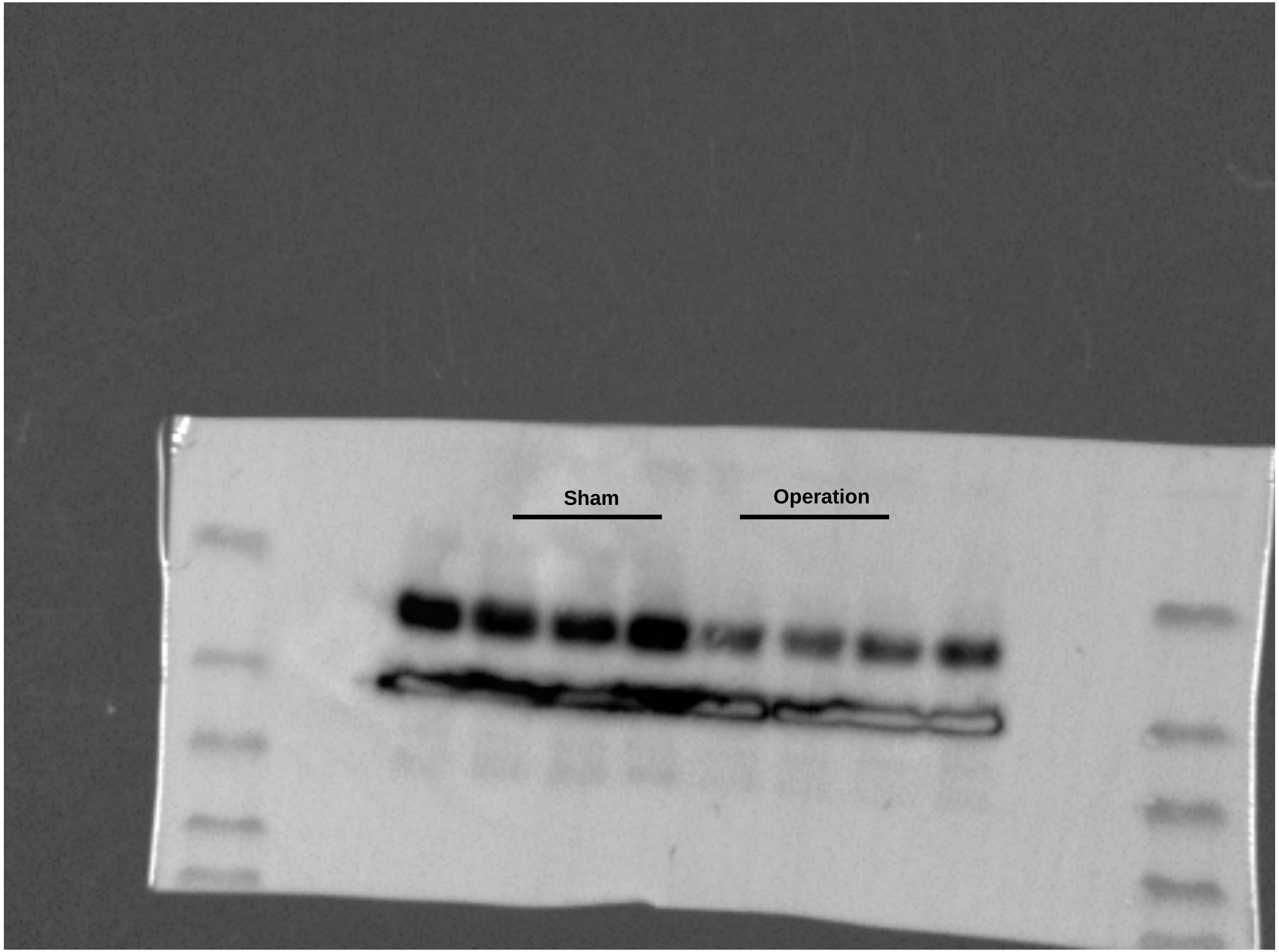

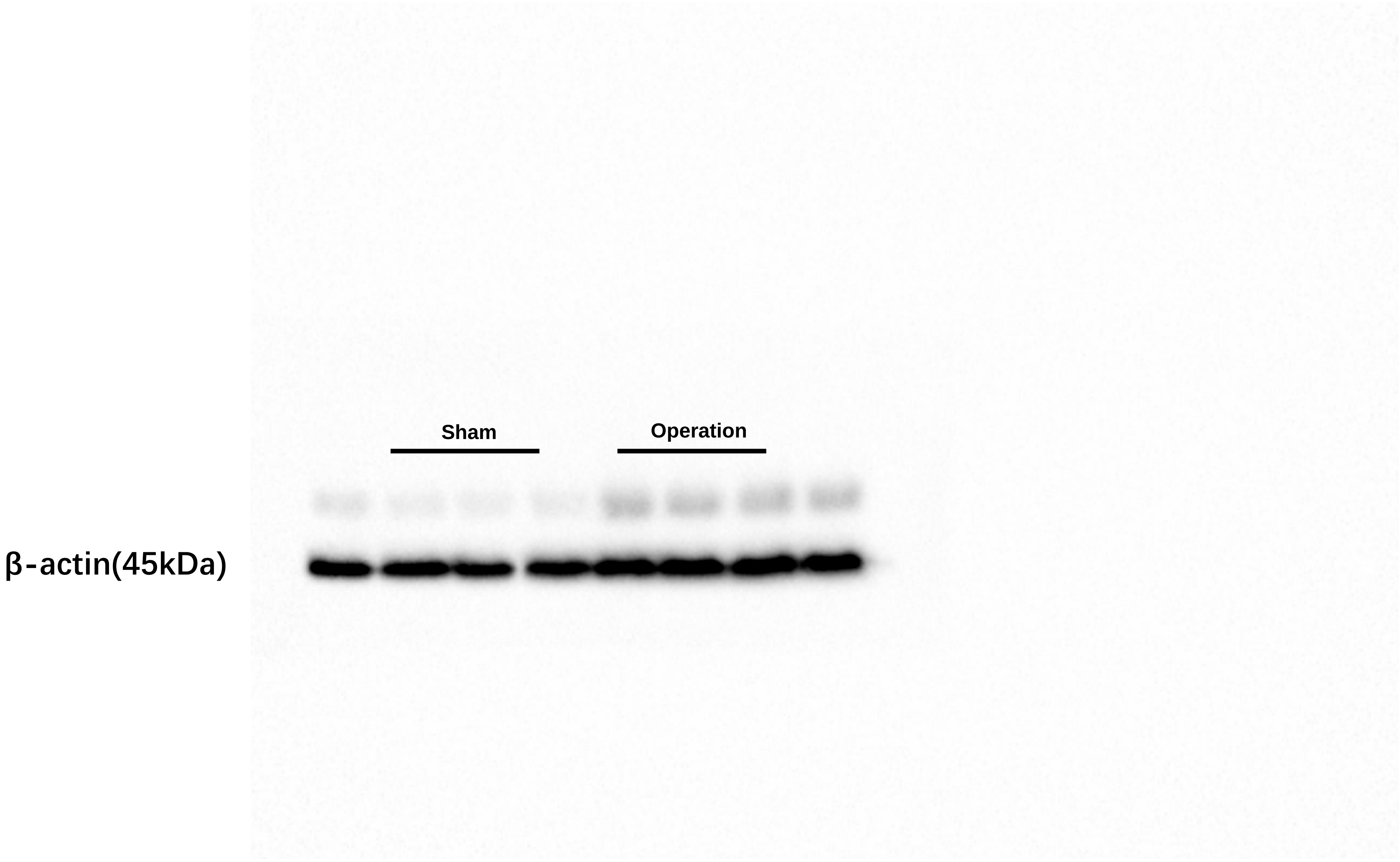

Sham
Operation
β-actin(45kDa)

CBR4(30-40kDa)

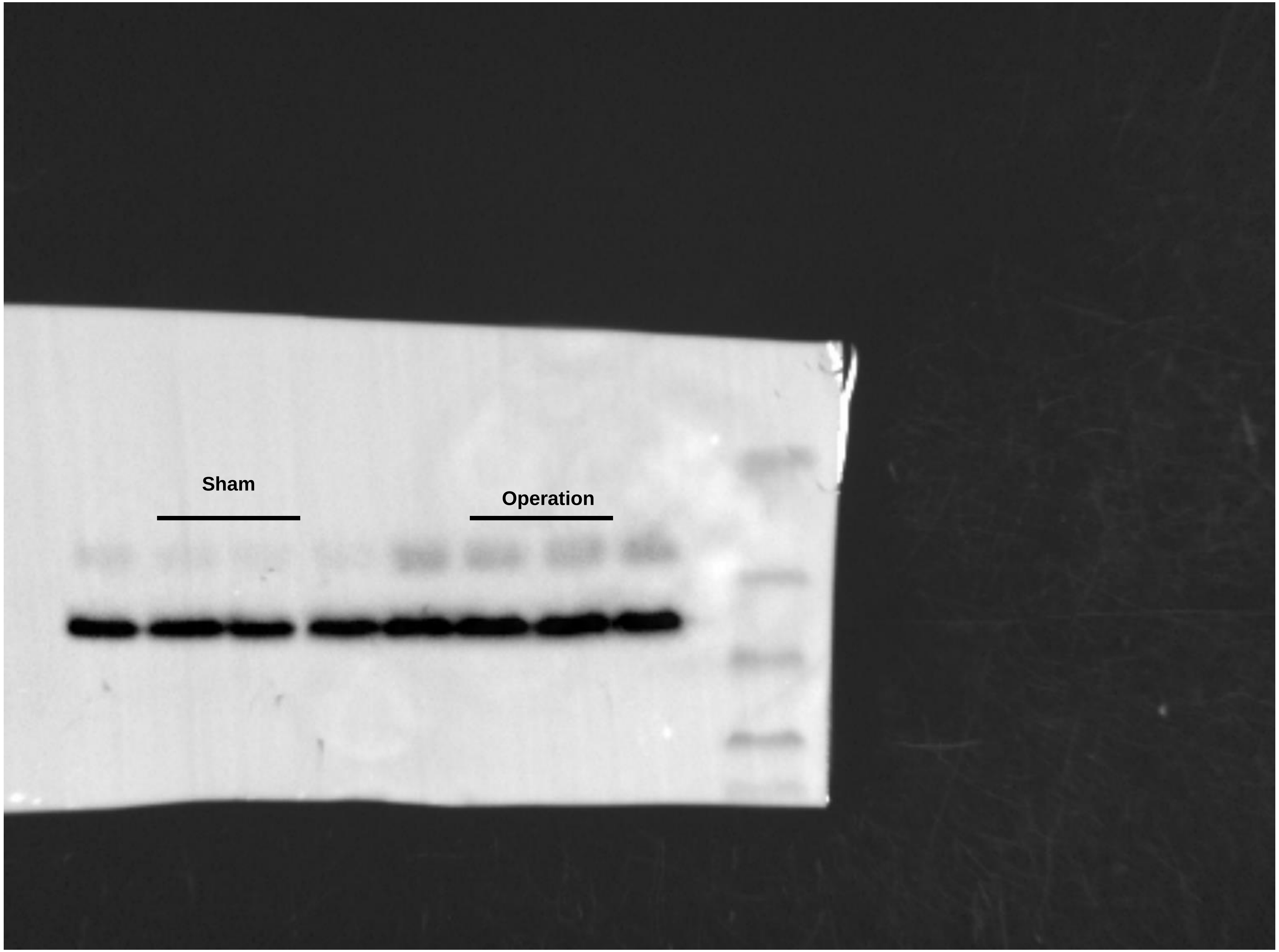

figure6-F

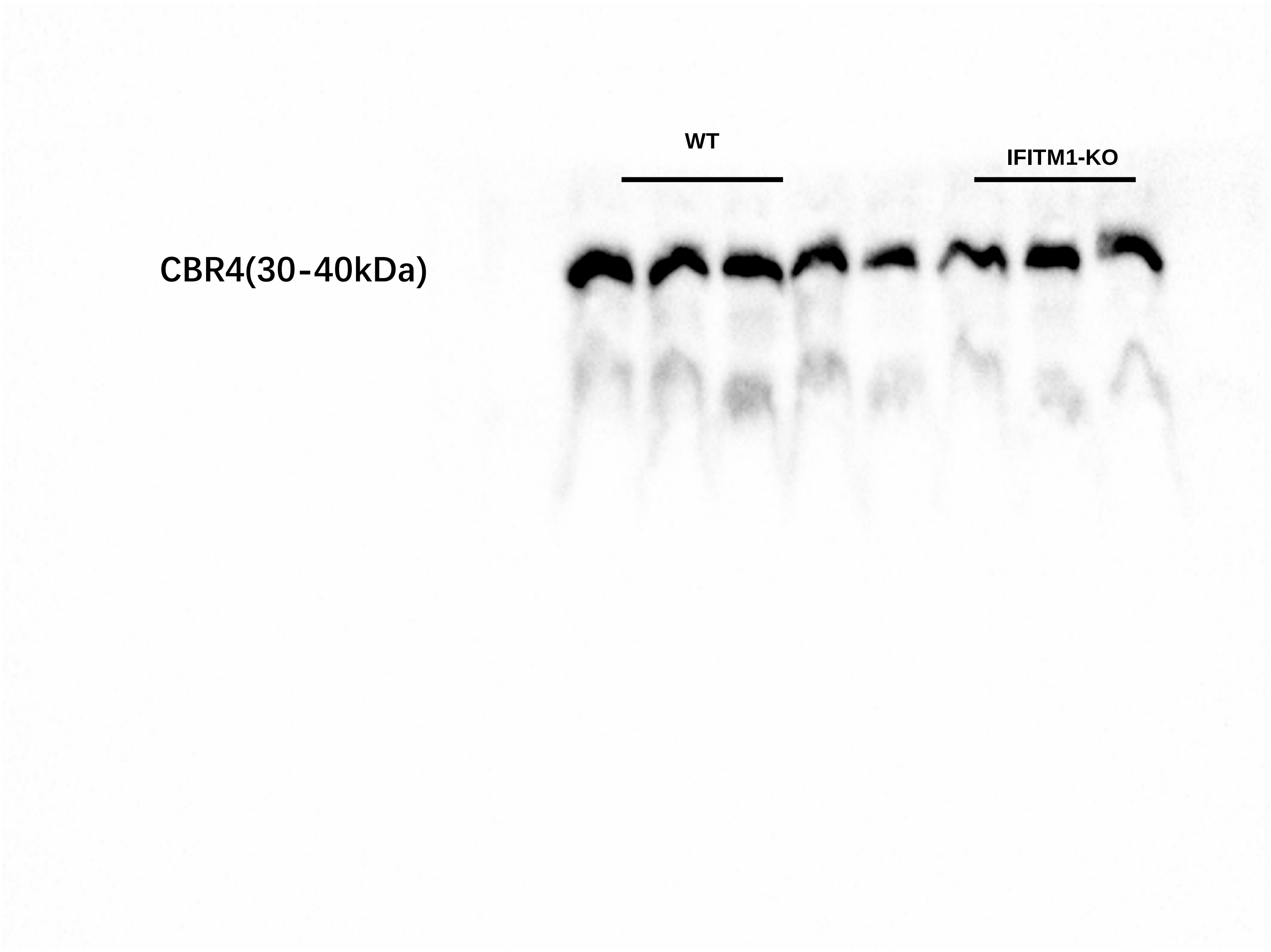

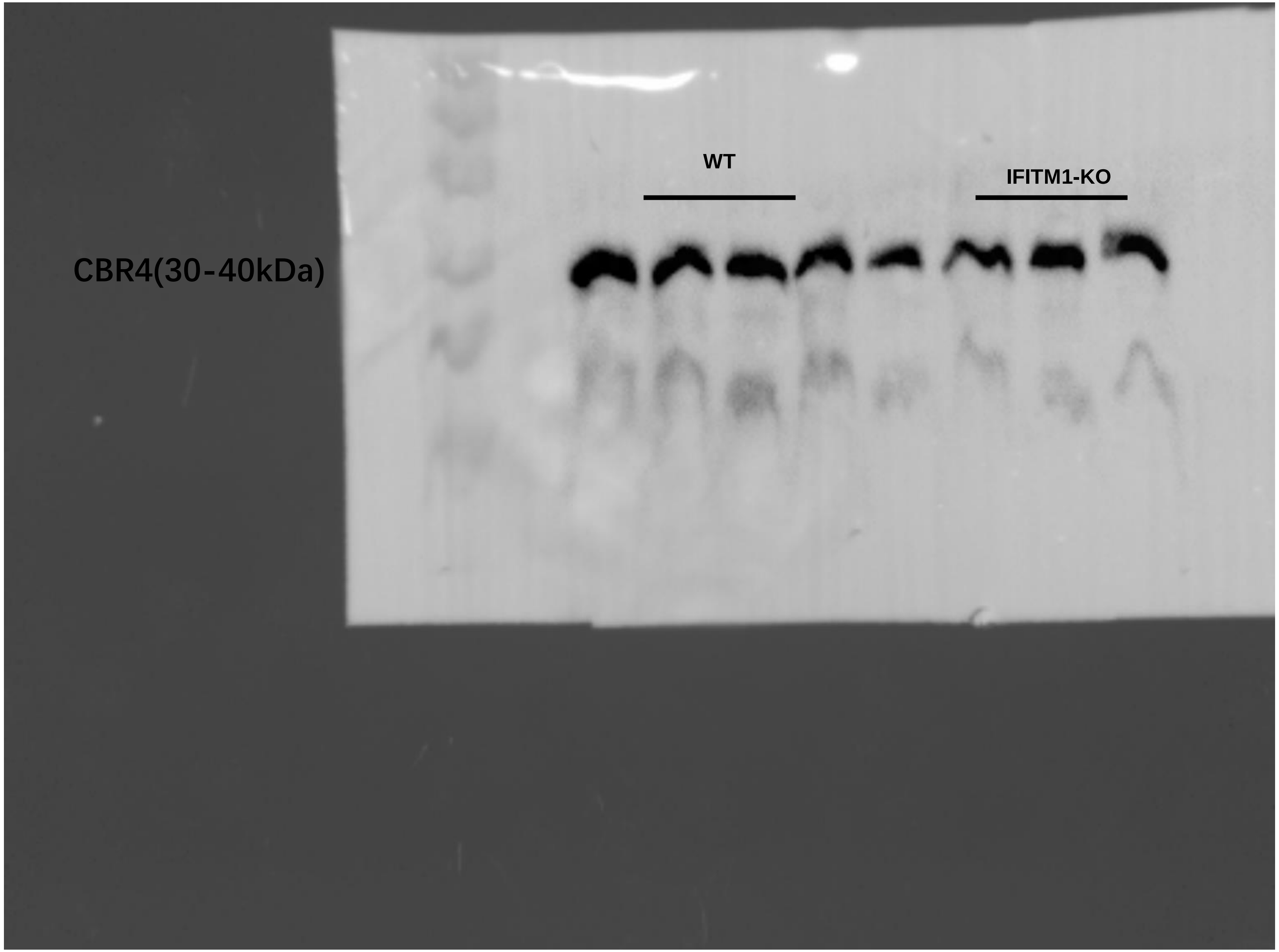
WT
IFITM1-KO
CBR4(30-40kDa)

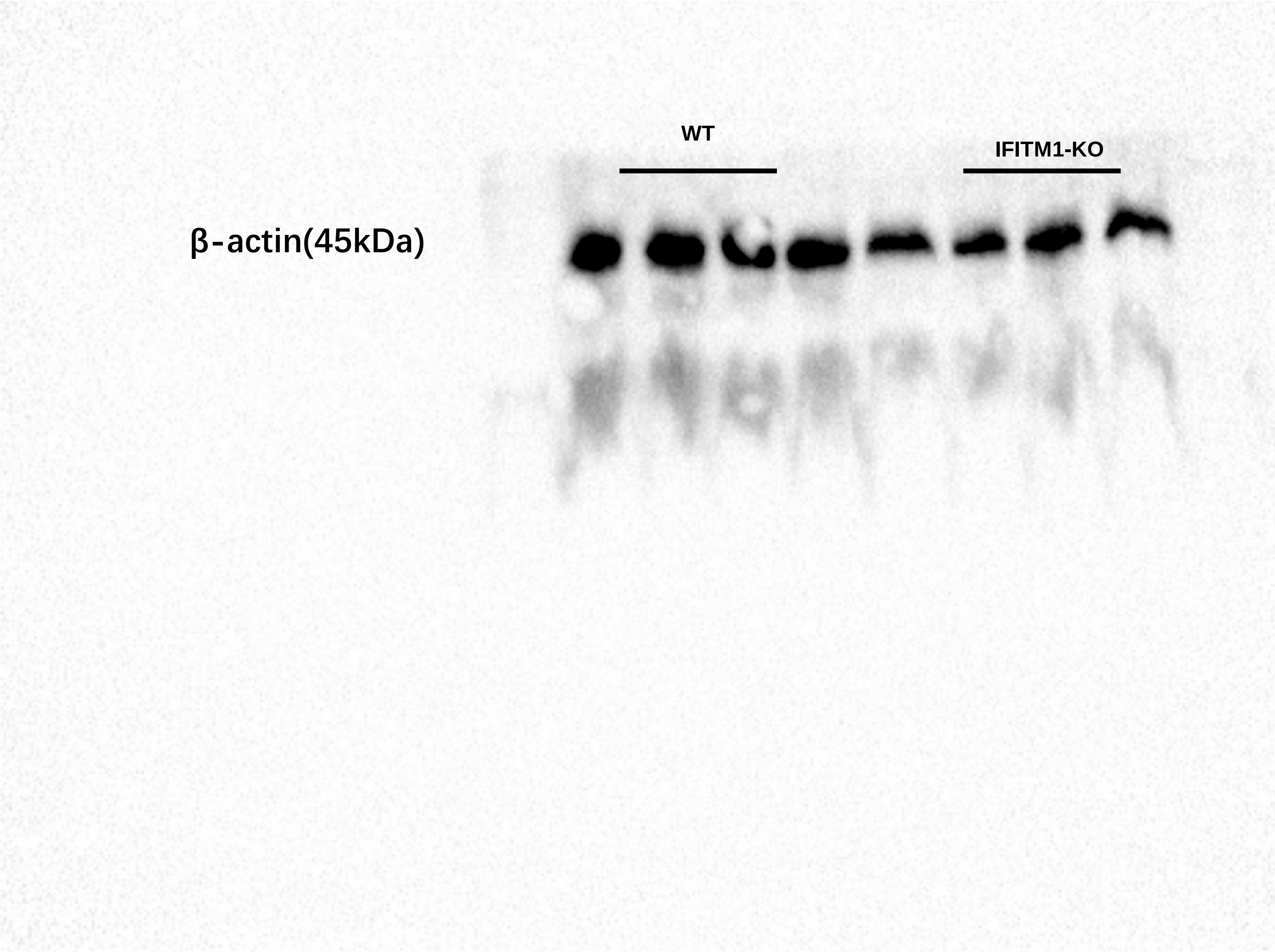
WT
IFITM1-KO
β-actin(45kDa)

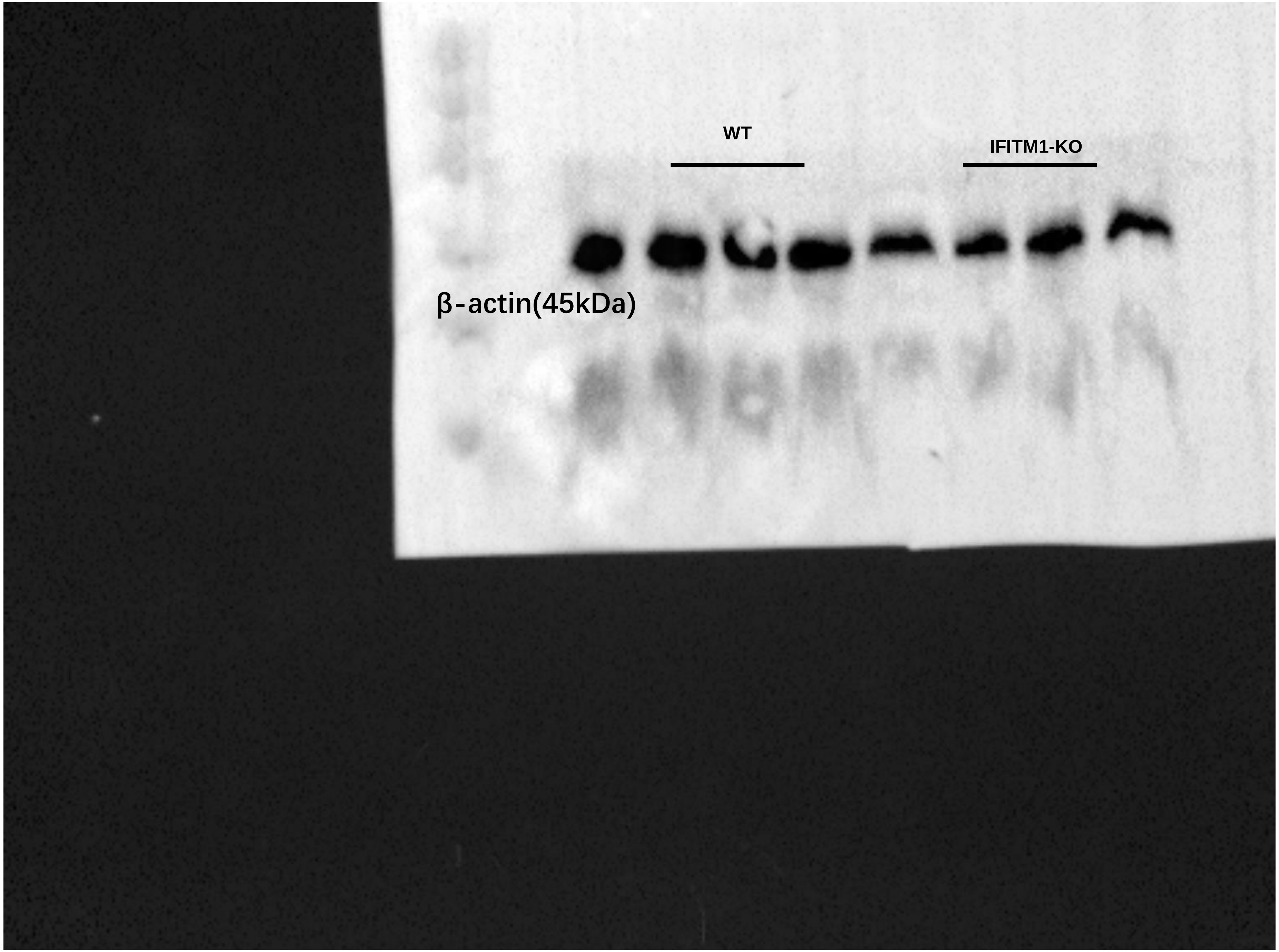
WT
IFITM1-KO
β-actin(45kDa)

figure7-A-1

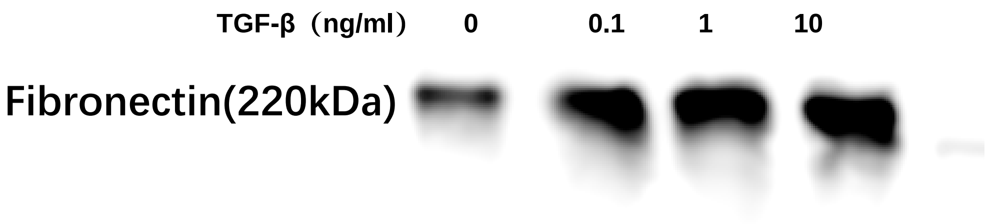

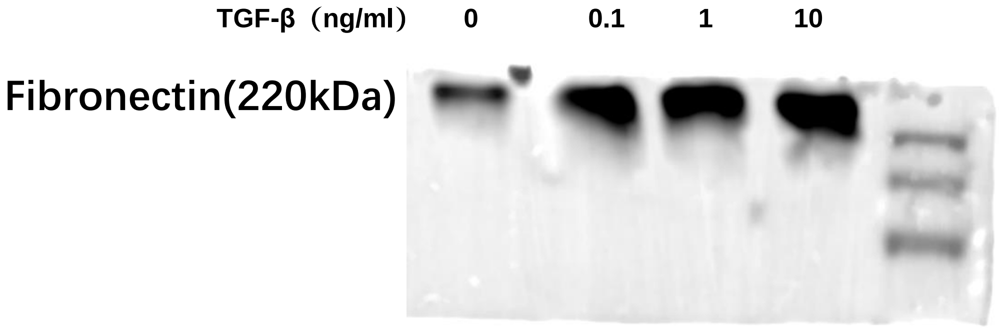
TGF-β（ng/ml） 0 0.1 1 10
Fibronectin(220kDa)

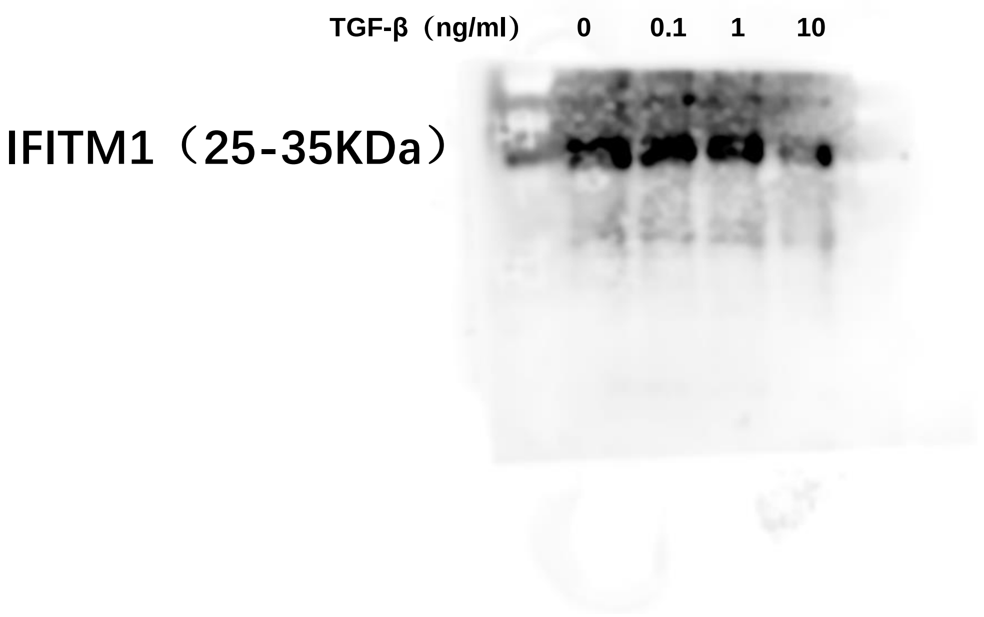
TGF-β（ng/ml） 0 0.1 1 10
IFITM1（25-35KDa）

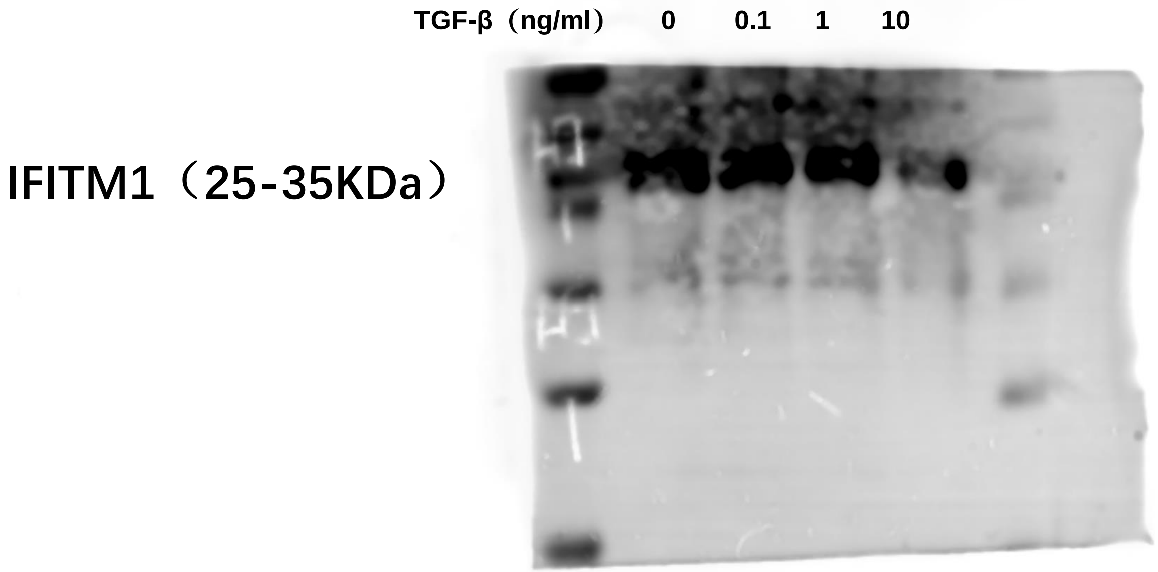
TGF-β（ng/ml） 0 0.1 1 10
IFITM1（25-35KDa）

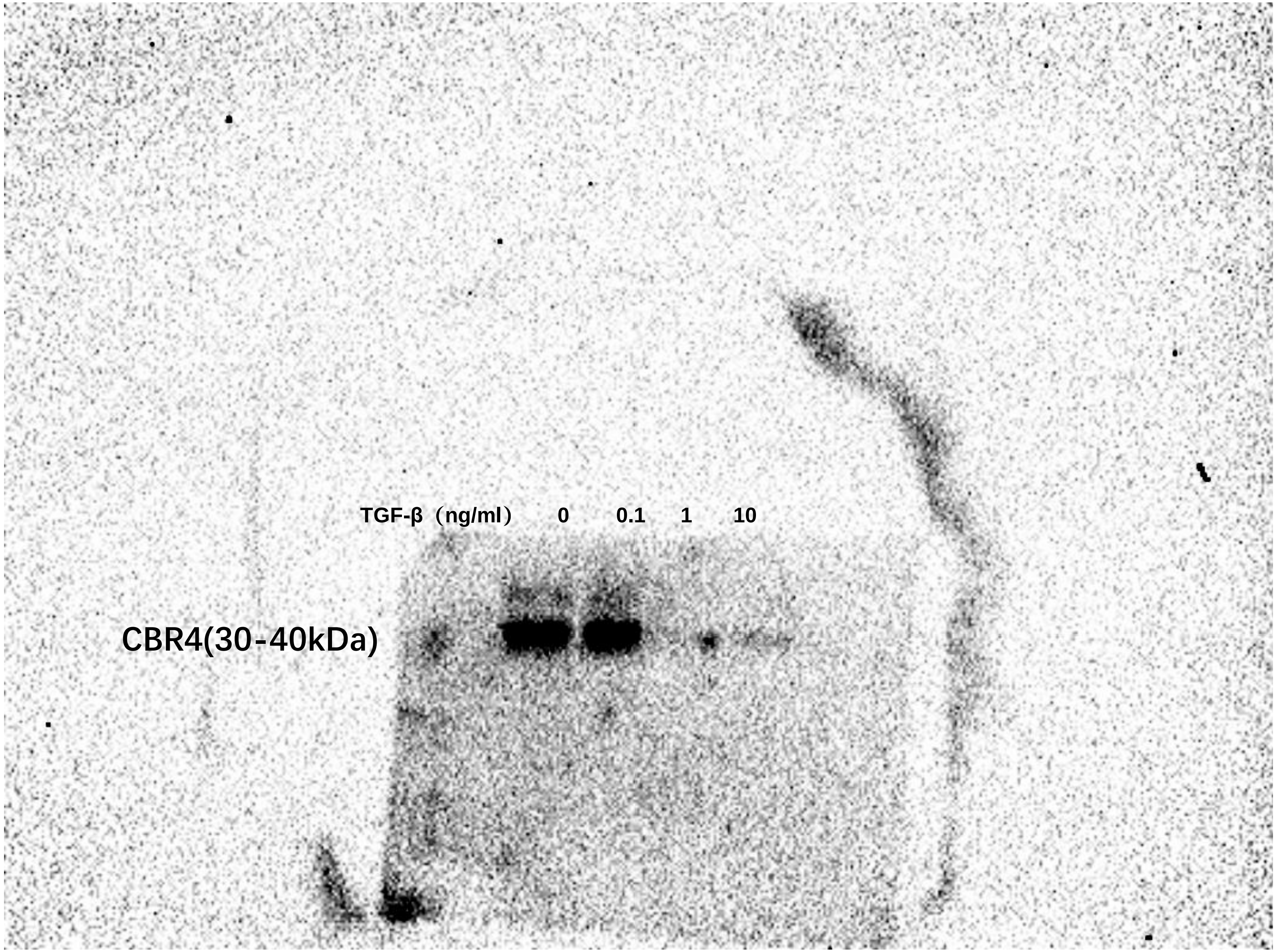
TGF-β（ng/ml） 0 0.1 1 10
CBR4(30-40kDa)

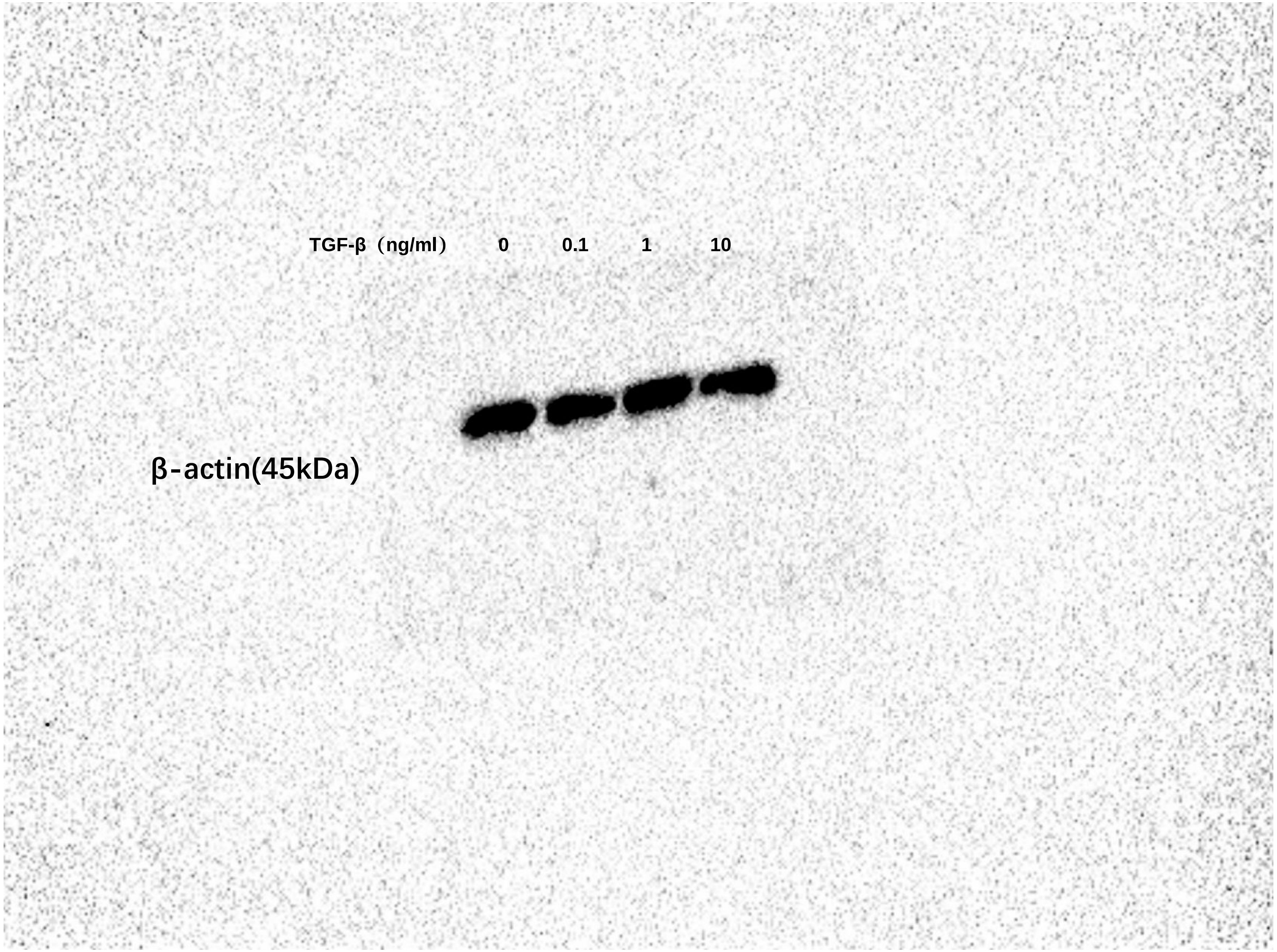
TGF-β（ng/ml） 0 0.1 1 10
β-actin(45kDa)

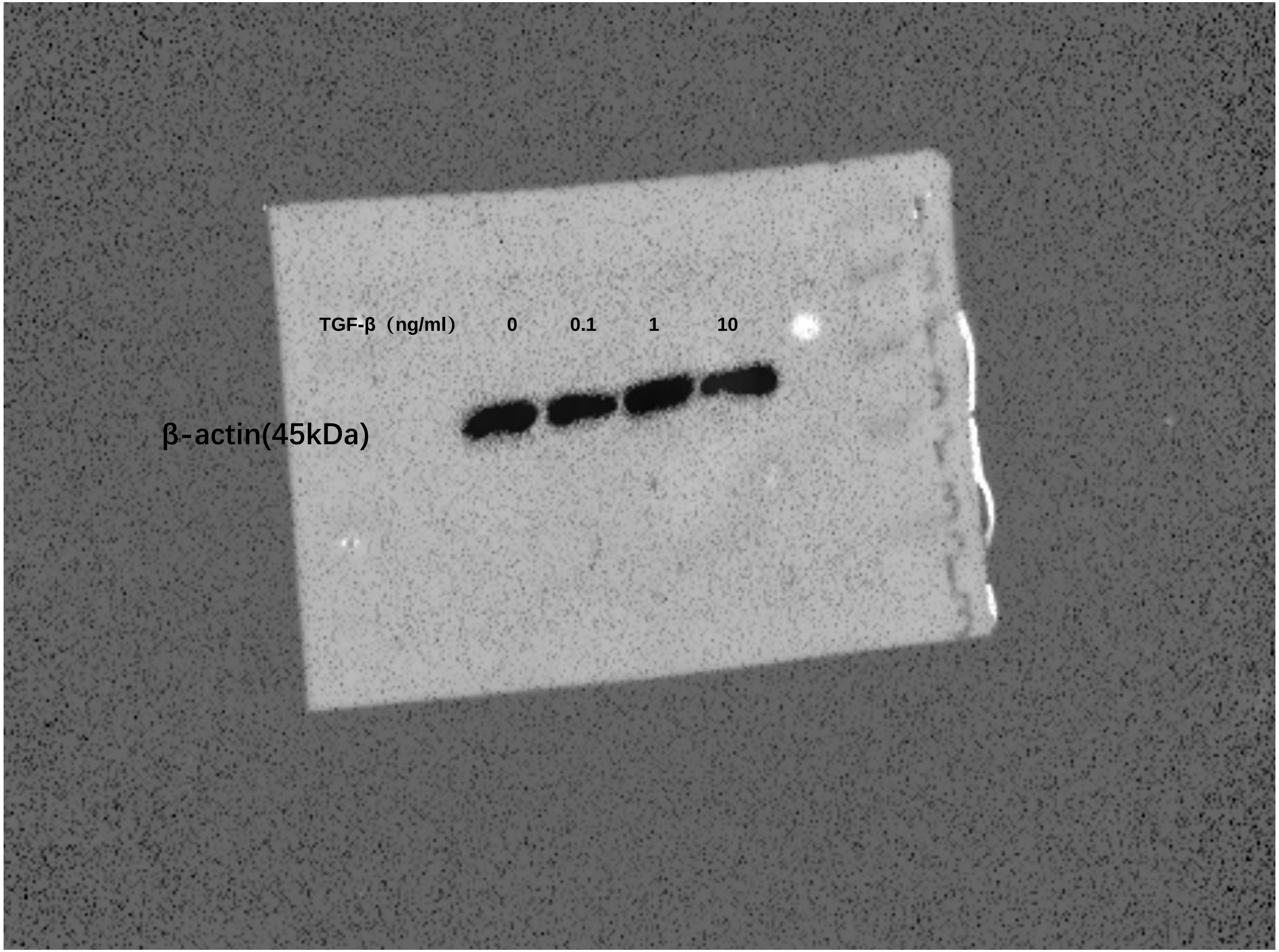
TGF-β（ng/ml）
0
0.1
1
10
β-actin(45kDa)

figure7-A-2

TGF-β（ng/ml） 0 0.1 1 10

Fibronectin(220kDa)

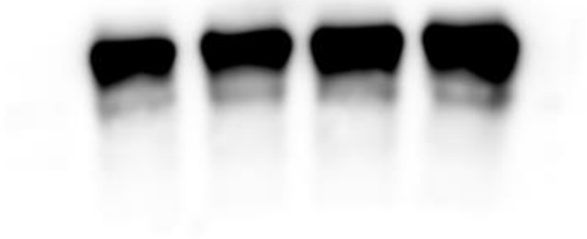

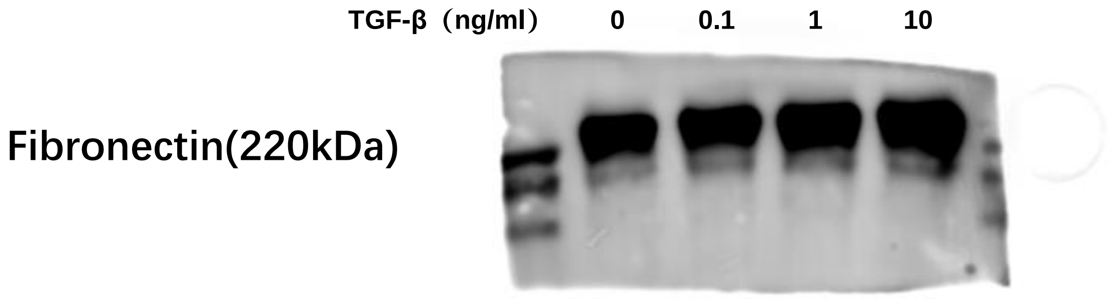
TGF-β（ng/ml）
0
0.1
1
10
Fibronectin(220kDa)

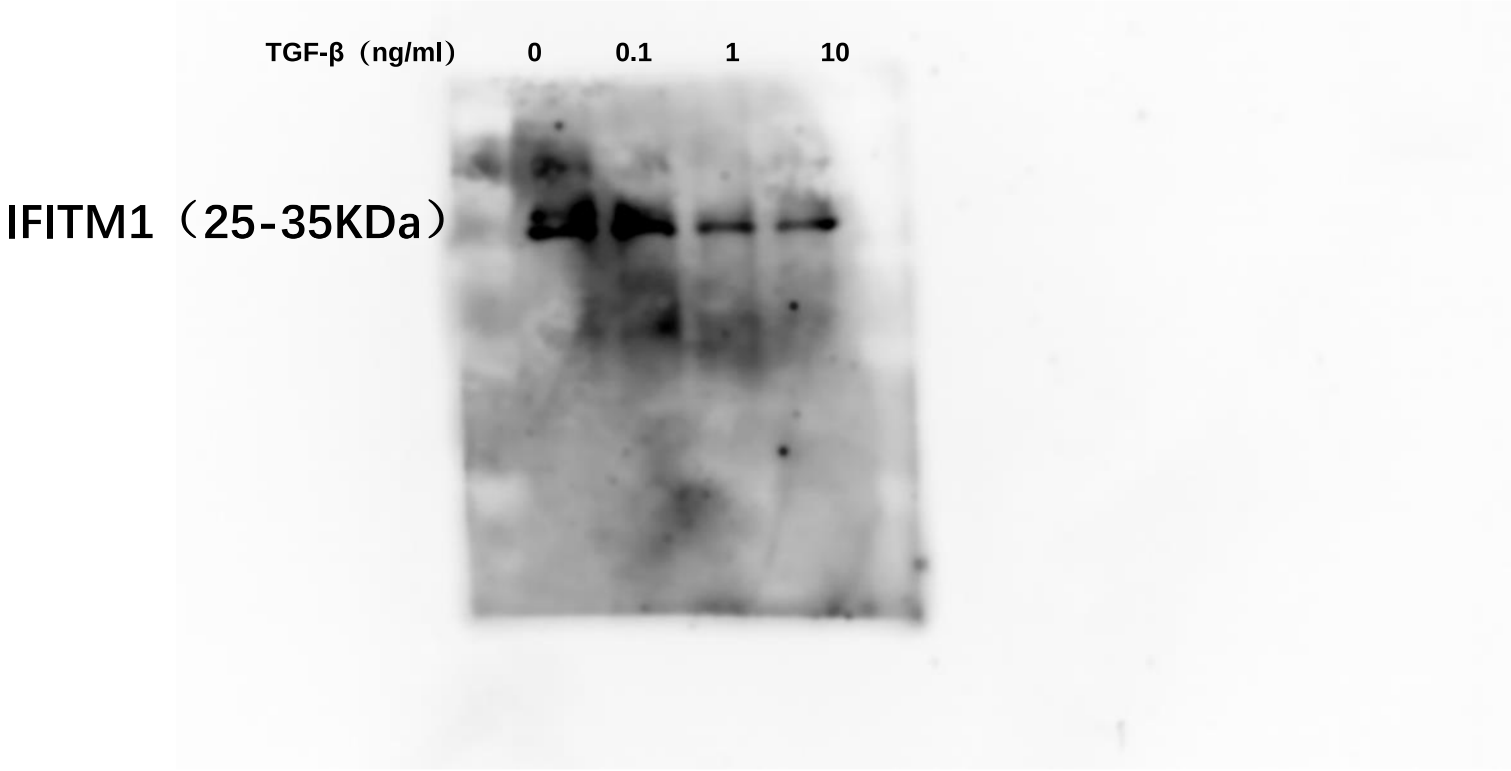
TGF-β（ng/ml）
0
0.1
1
10
IFITM1（25-35KDa）

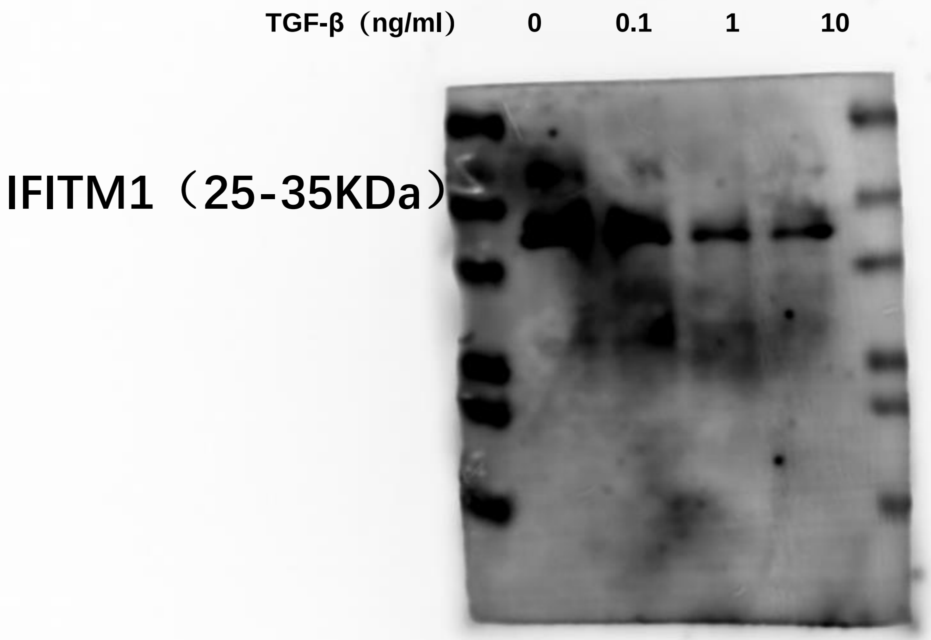
TGF-β（ng/ml） 0 0.1 1 10
IFITM1（25-35KDa）

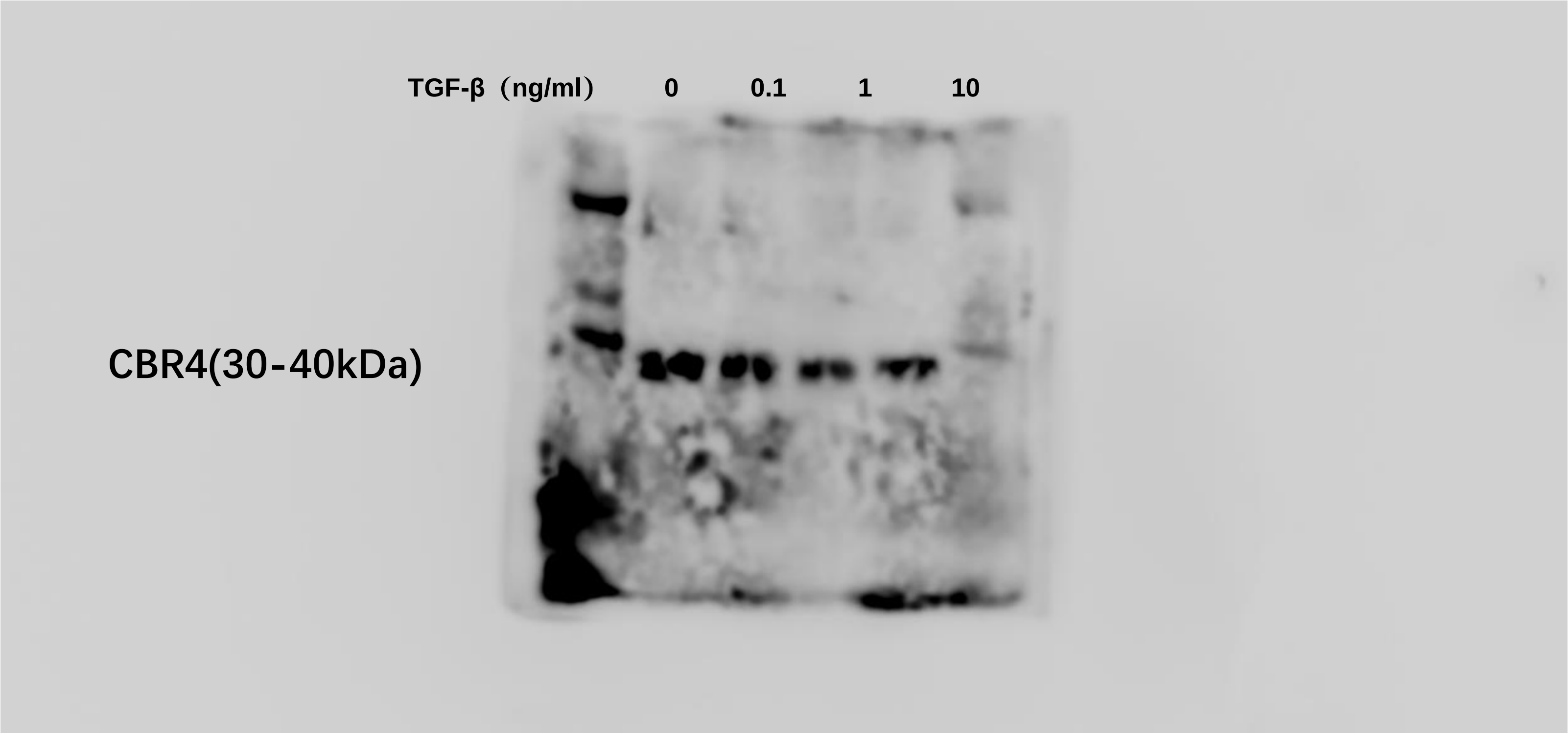
TGF-β（ng/ml）
0
0.1
1
10
CBR4(30-40kDa)

CBR4(30-40kDa)

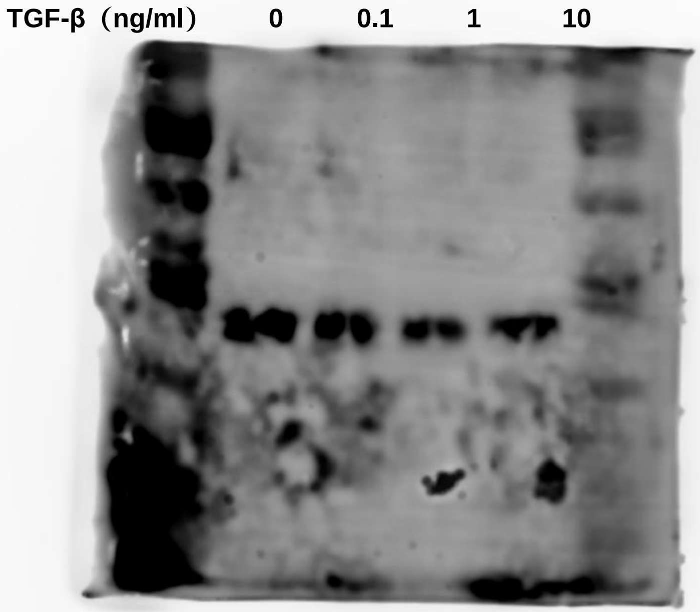

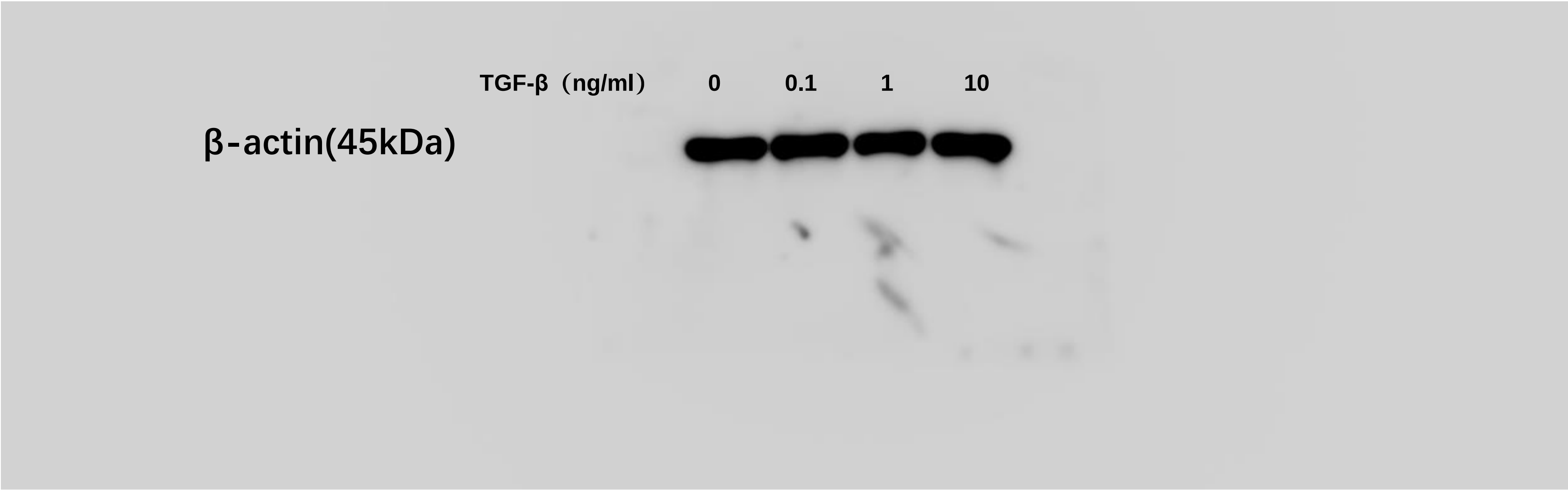
TGF-β（ng/ml）
0
0.1
1
10
β-actin(45kDa)

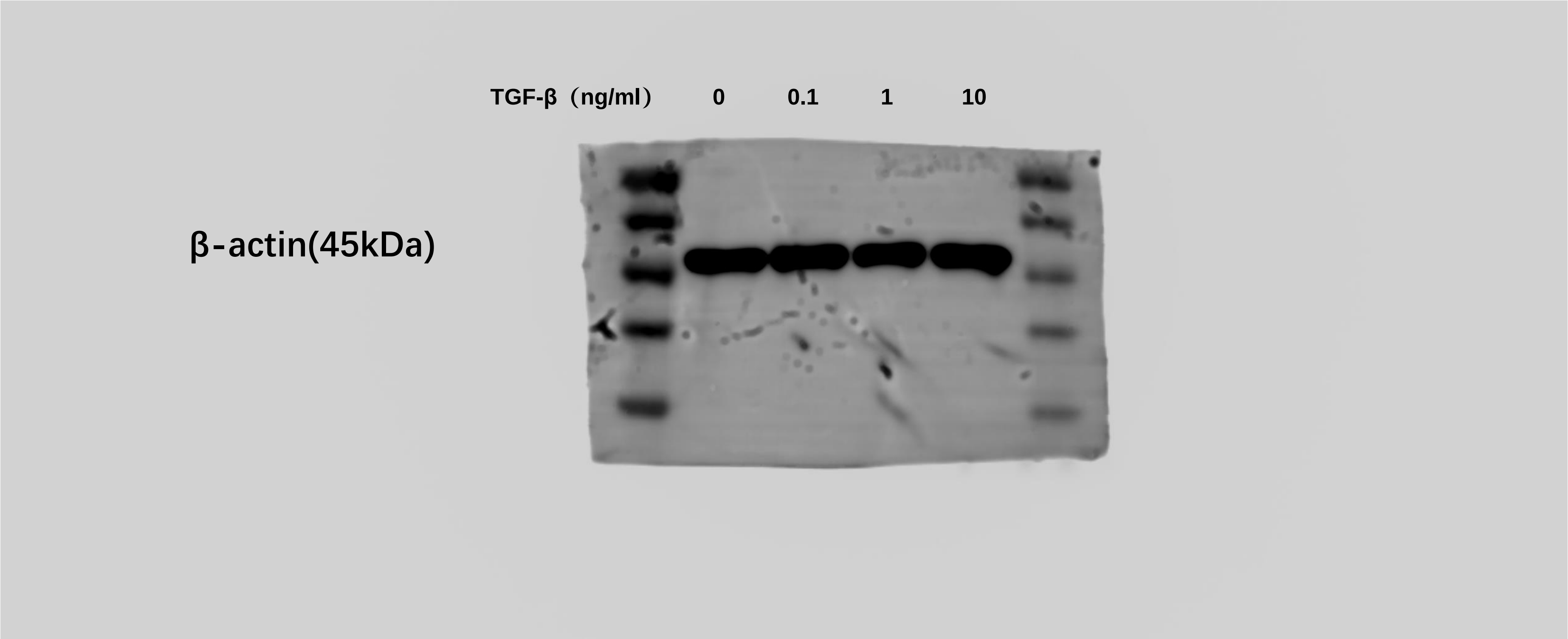
TGF-β（ng/ml） 0 0.1 1 10
β-actin(45kDa)

figure7-A-3

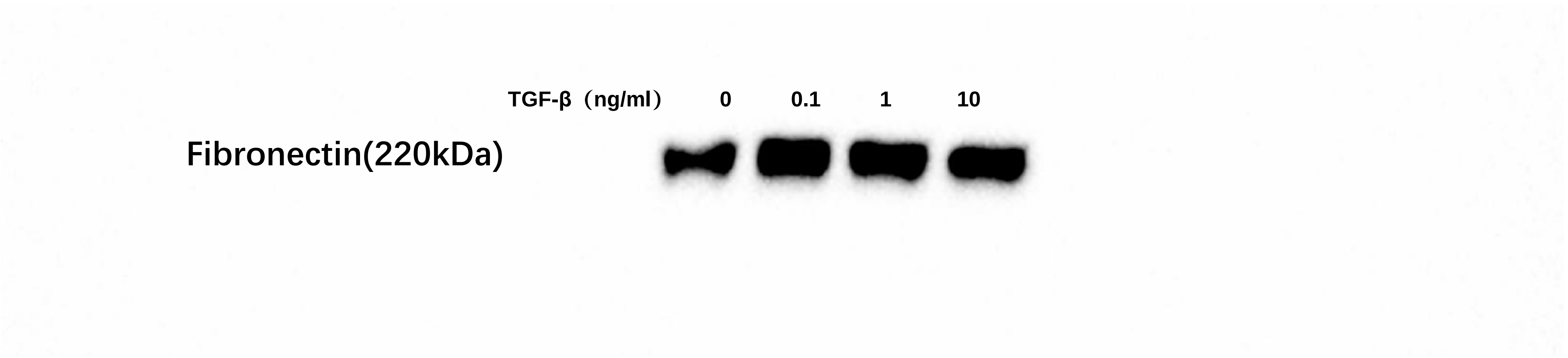

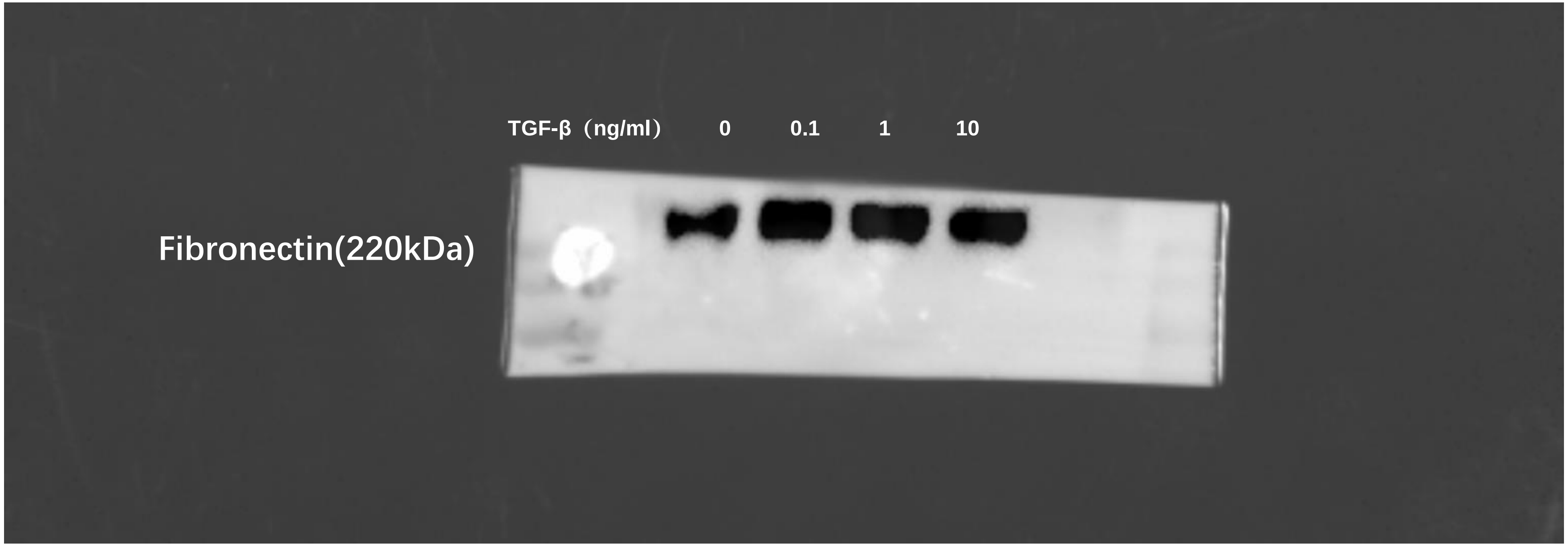
TGF-β（ng/ml）
0
0.1
1
10
Fibronectin(220kDa)

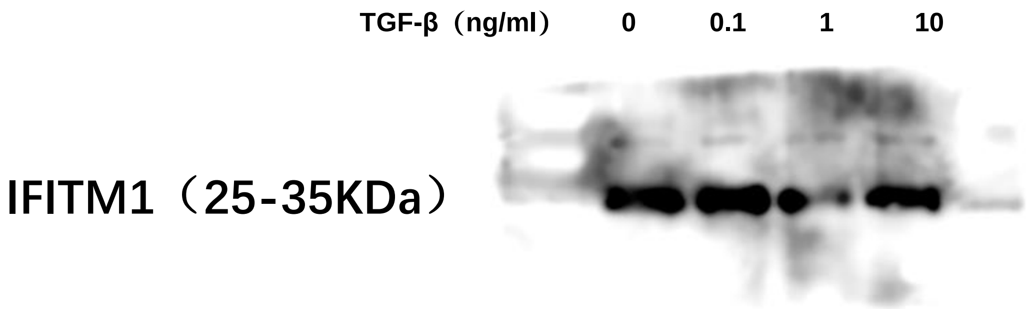
TGF-β（ng/ml） 0 0.1 1 10
IFITM1（25-35KDa）

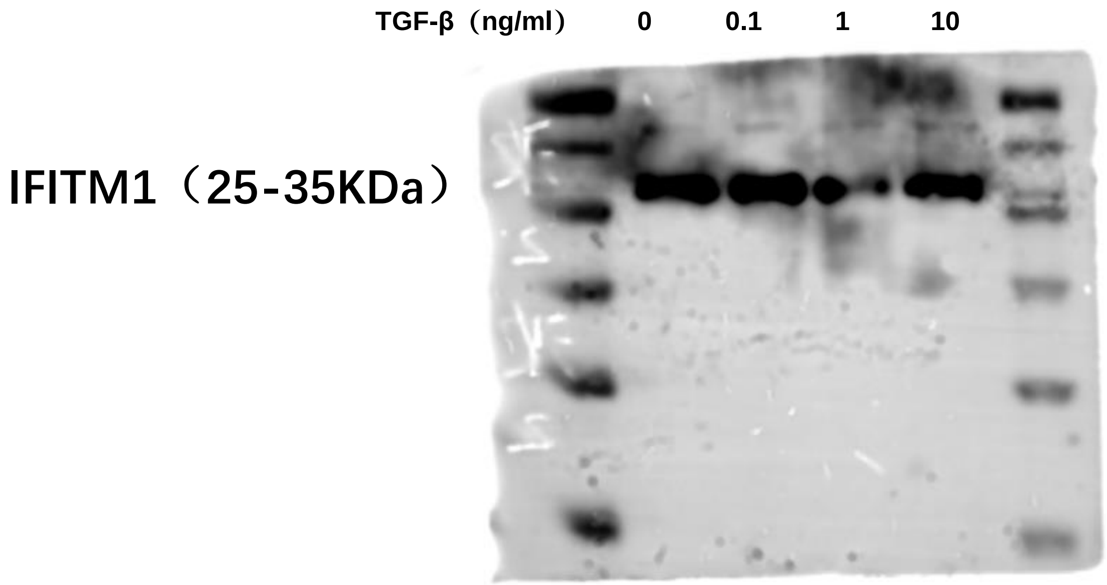

TGF-β（ng/ml）
0
0.1
1
10
IFITM1（25-35KDa）

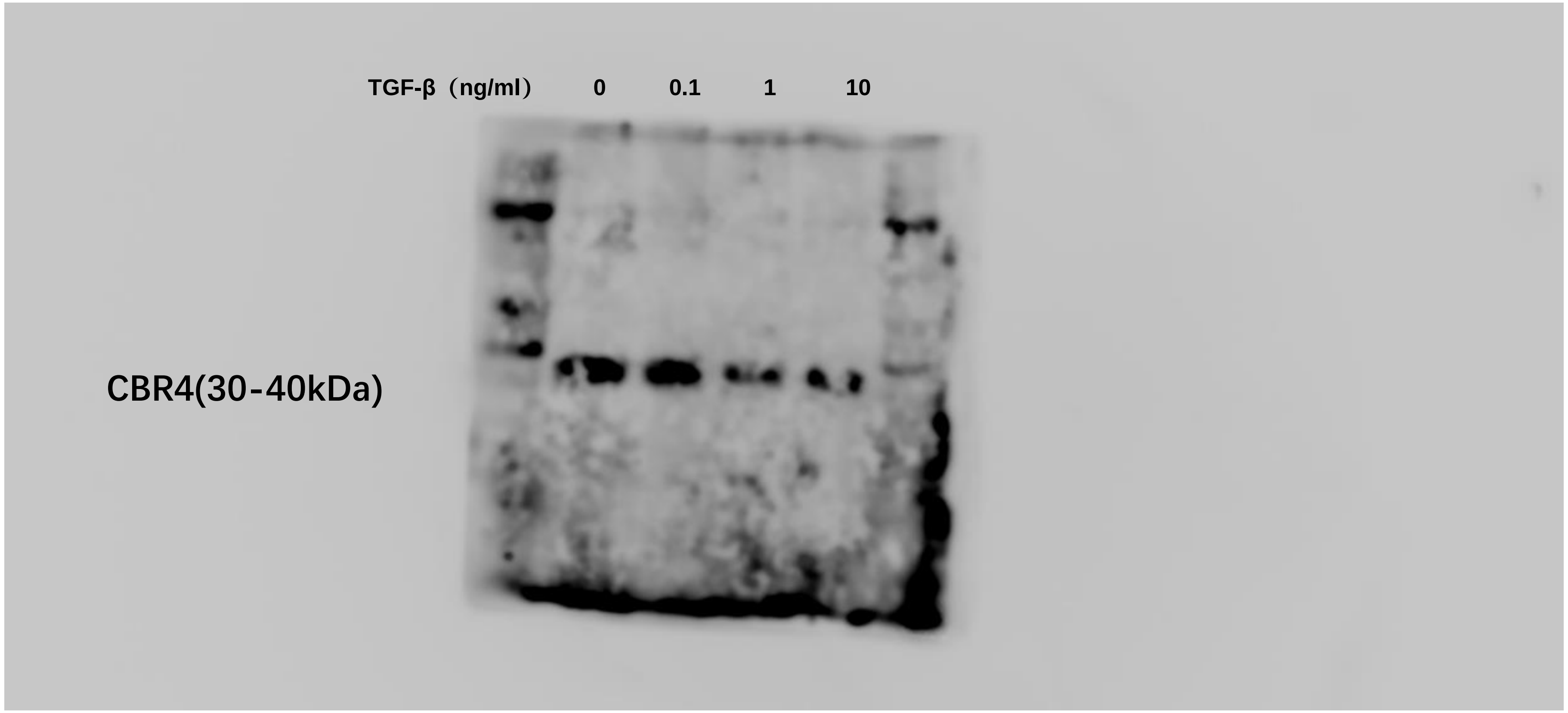
TGF-β（ng/ml）
0
0.1
1
10
CBR4(30-40kDa)

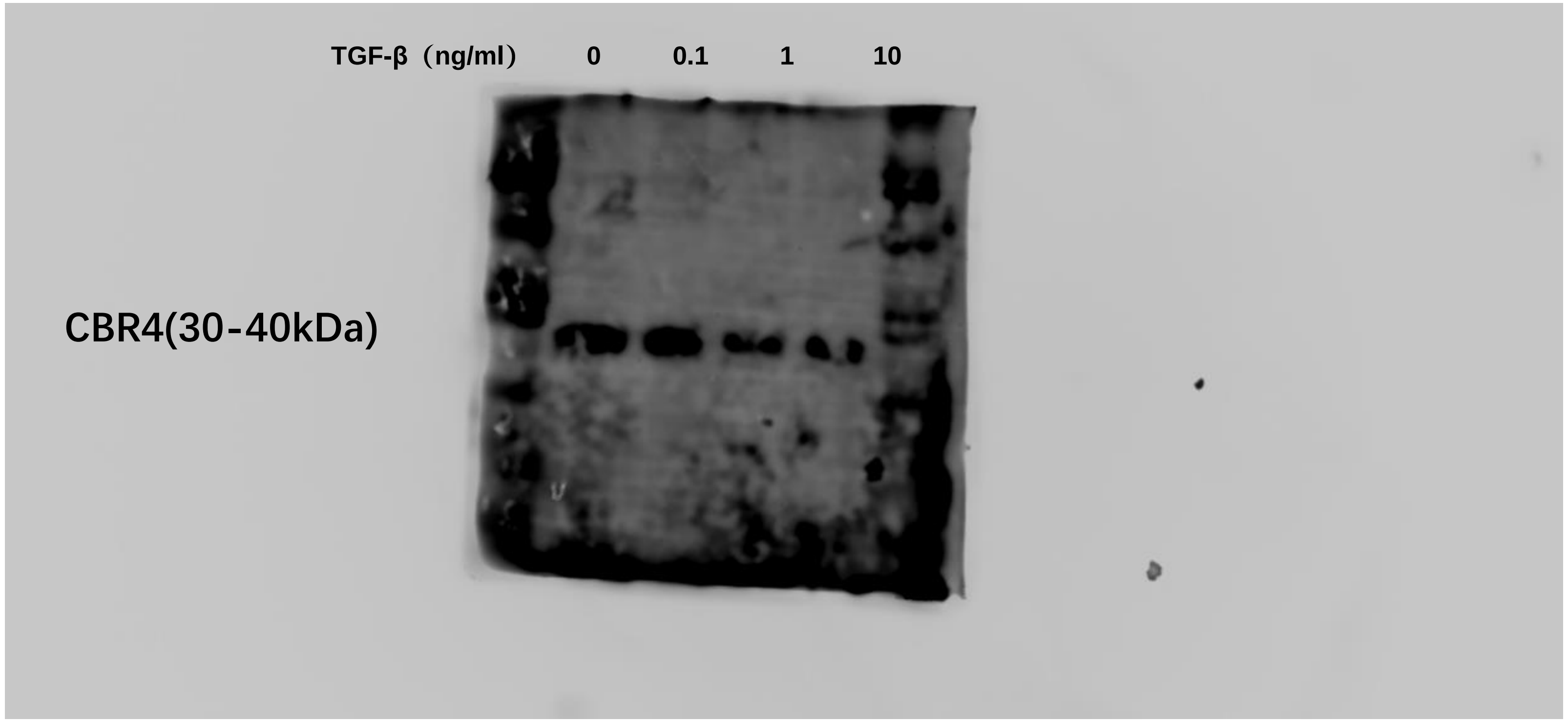
TGF-β（ng/ml）
0
0.1
1
10
CBR4(30-40kDa)

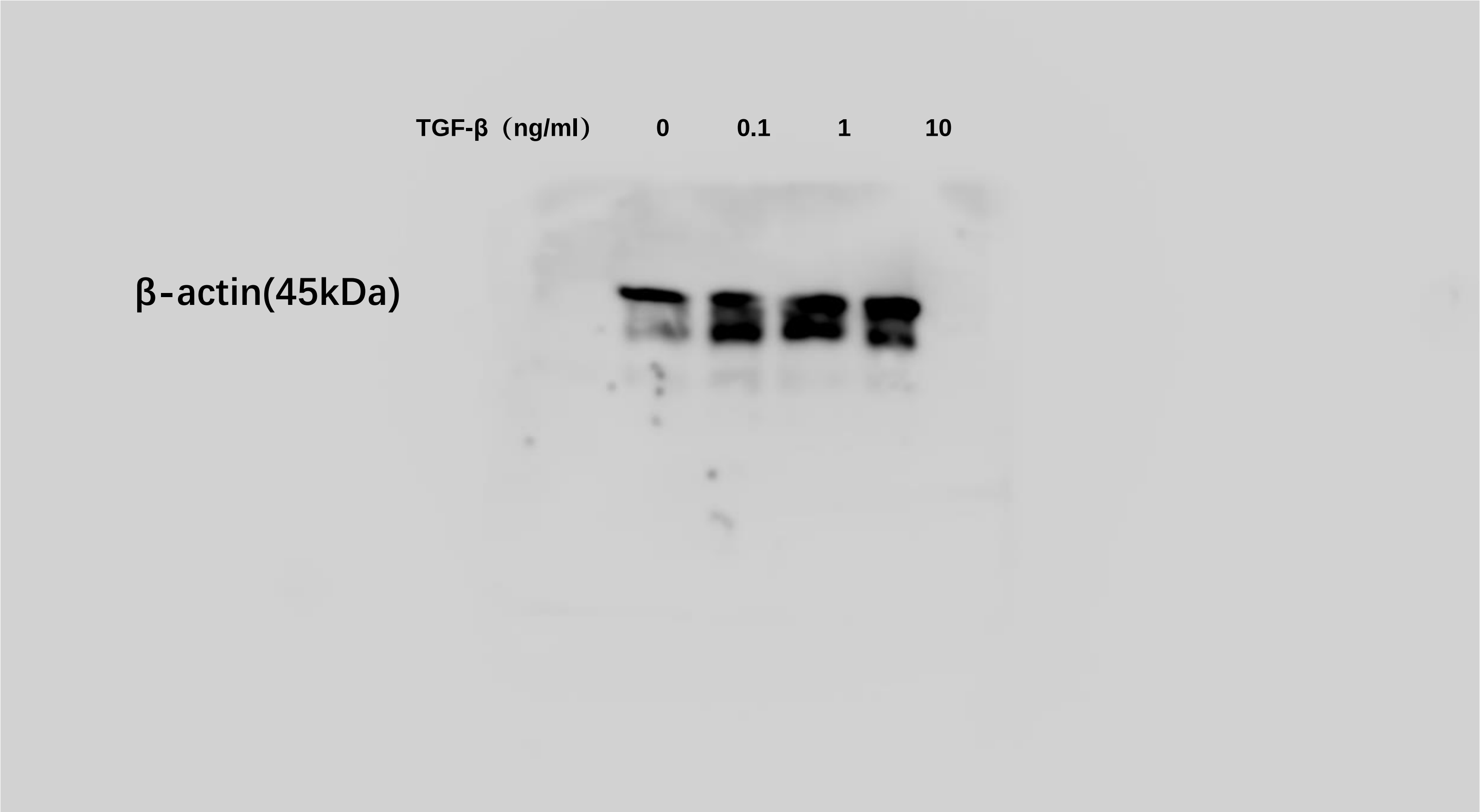
TGF-β（ng/ml）
0
0.1
1
10
β-actin(45kDa)

figure7-B-1

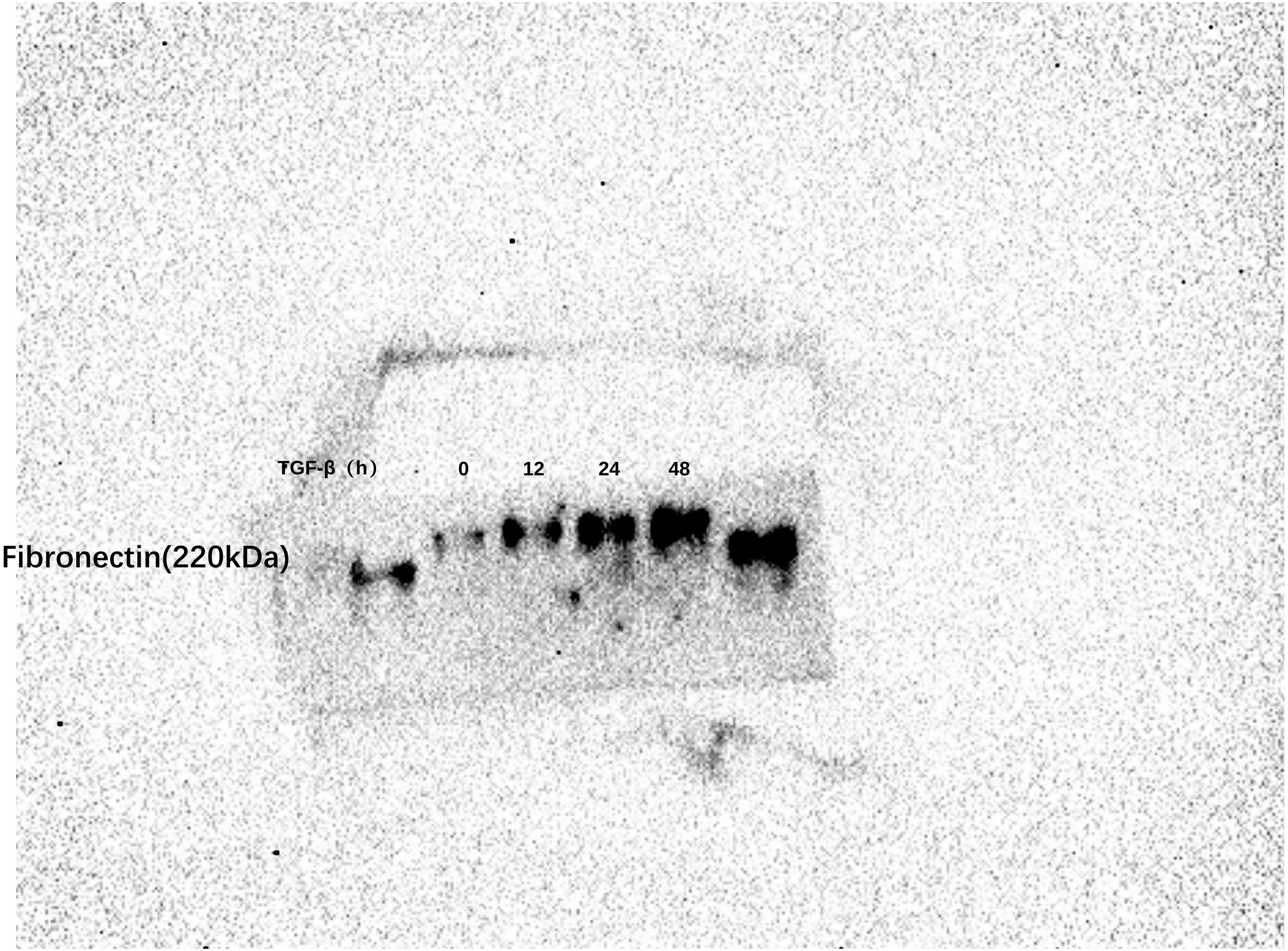

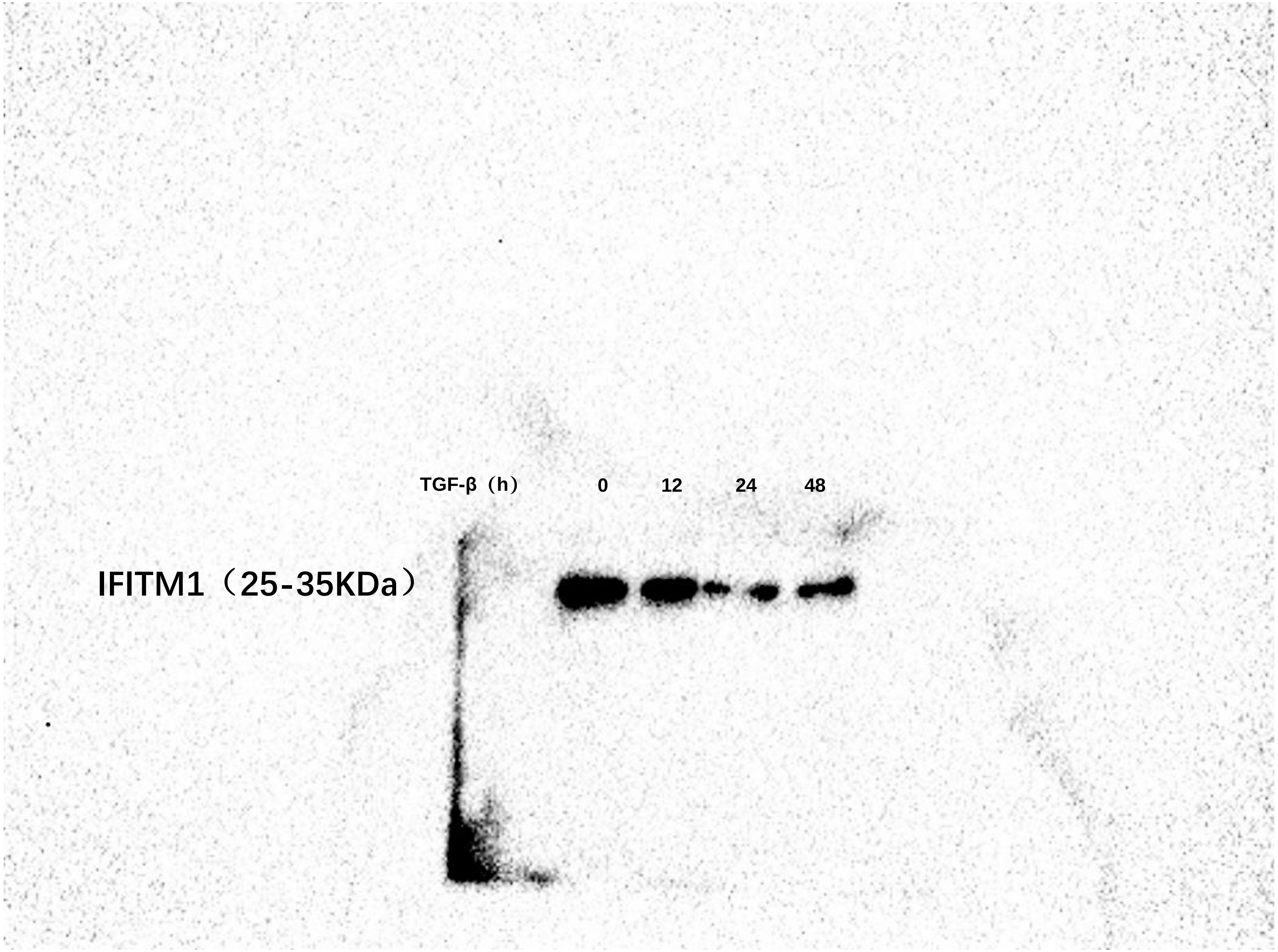
TGF-β（h）
0
12
24
48
IFITM1（25-35KDa）

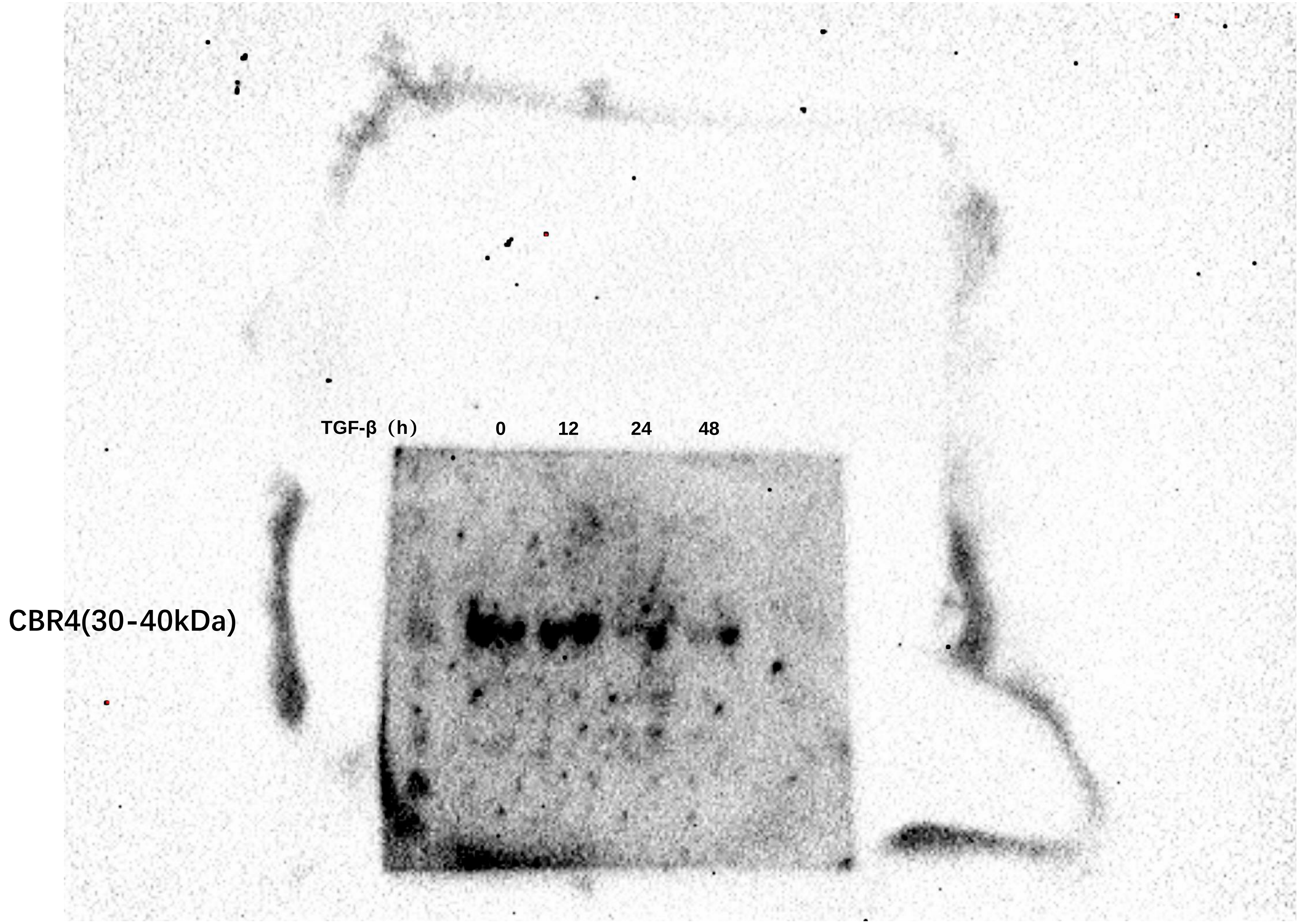
TGF-β（h）
0
12
24
48
CBR4(30-40kDa)

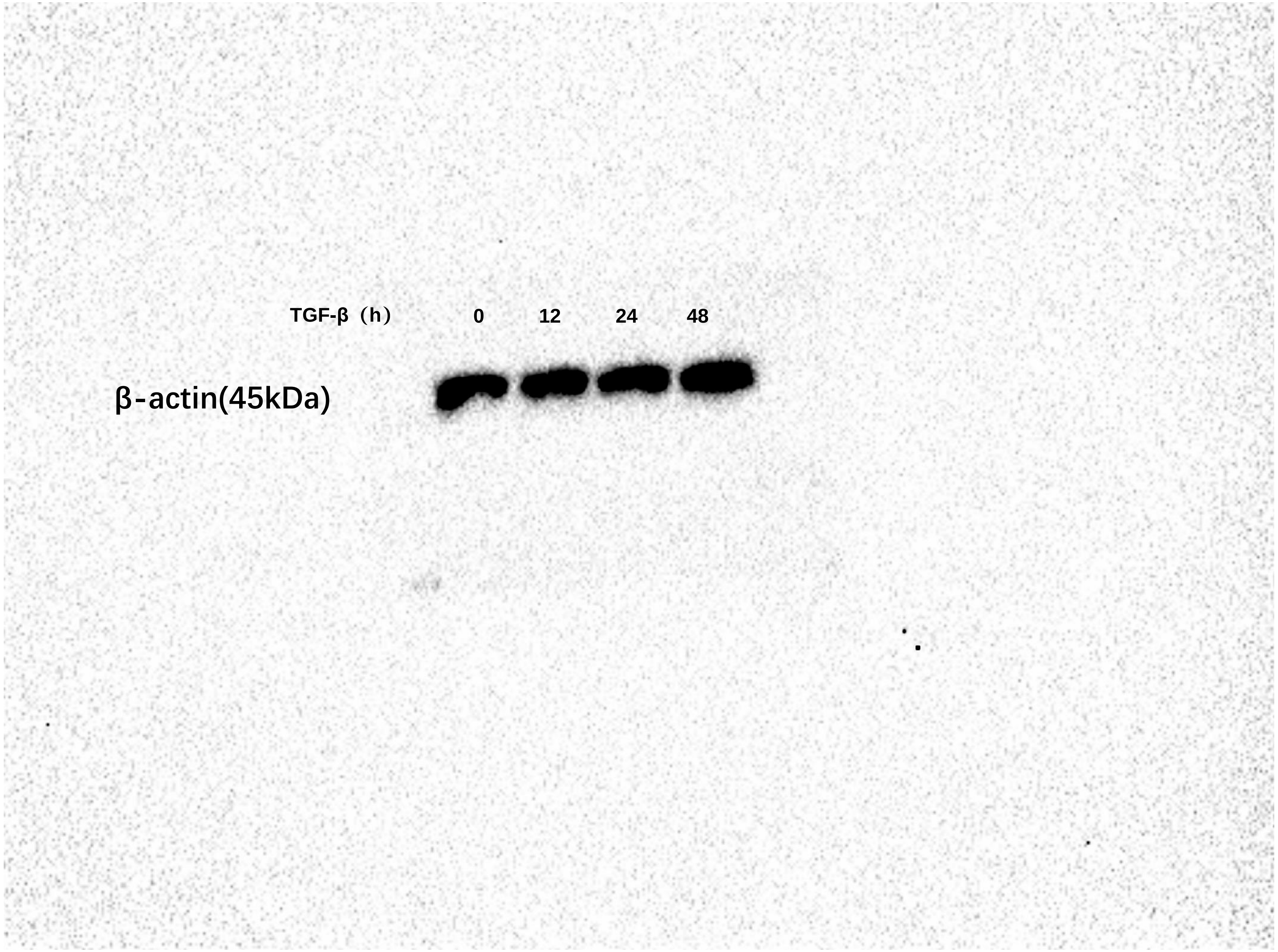
TGF-β（h）
0
12
24
48
β-actin(45kDa)

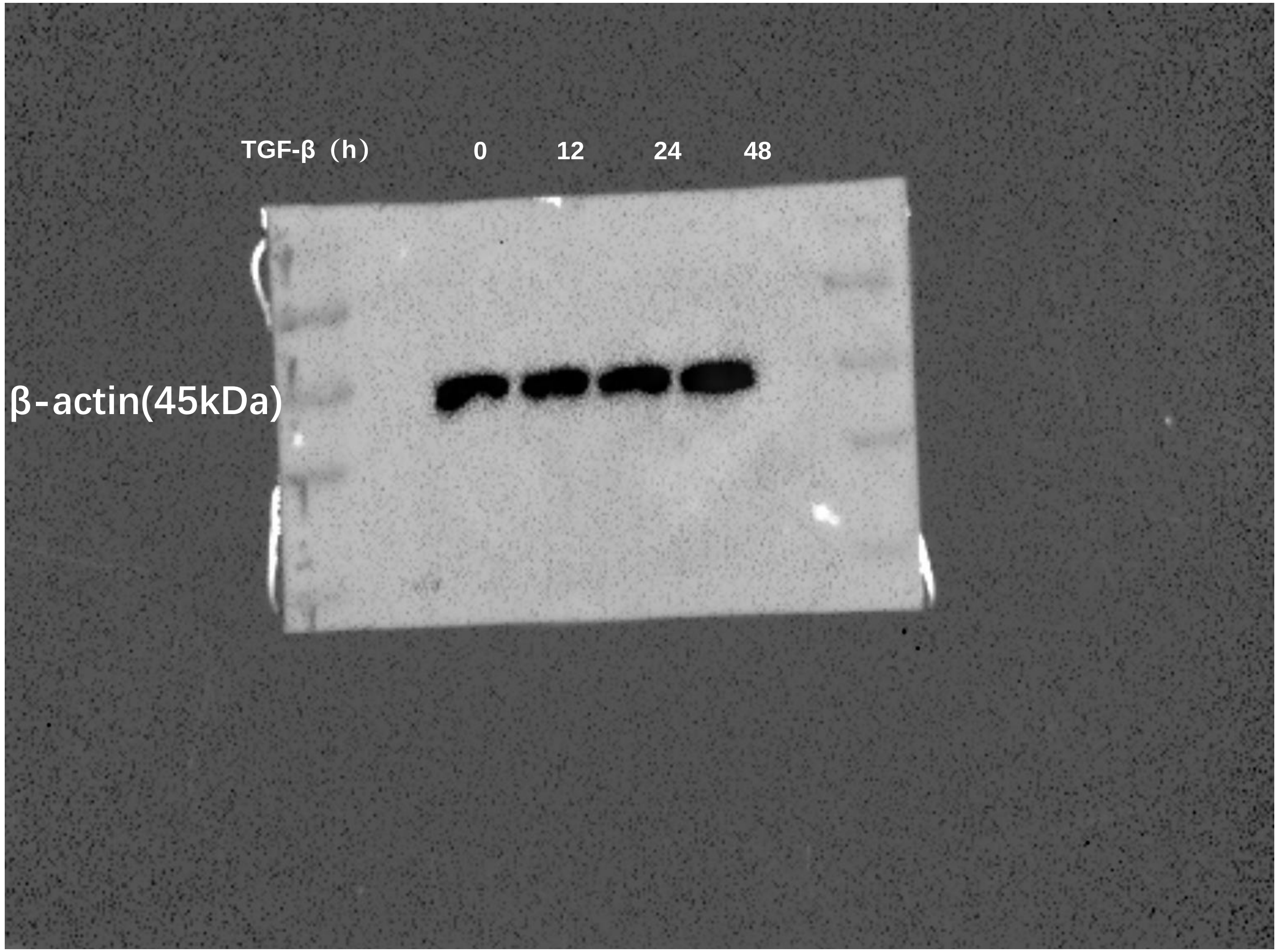
TGF-β（h）
0
12
24
48
β-actin(45kDa)

figure7-B-2

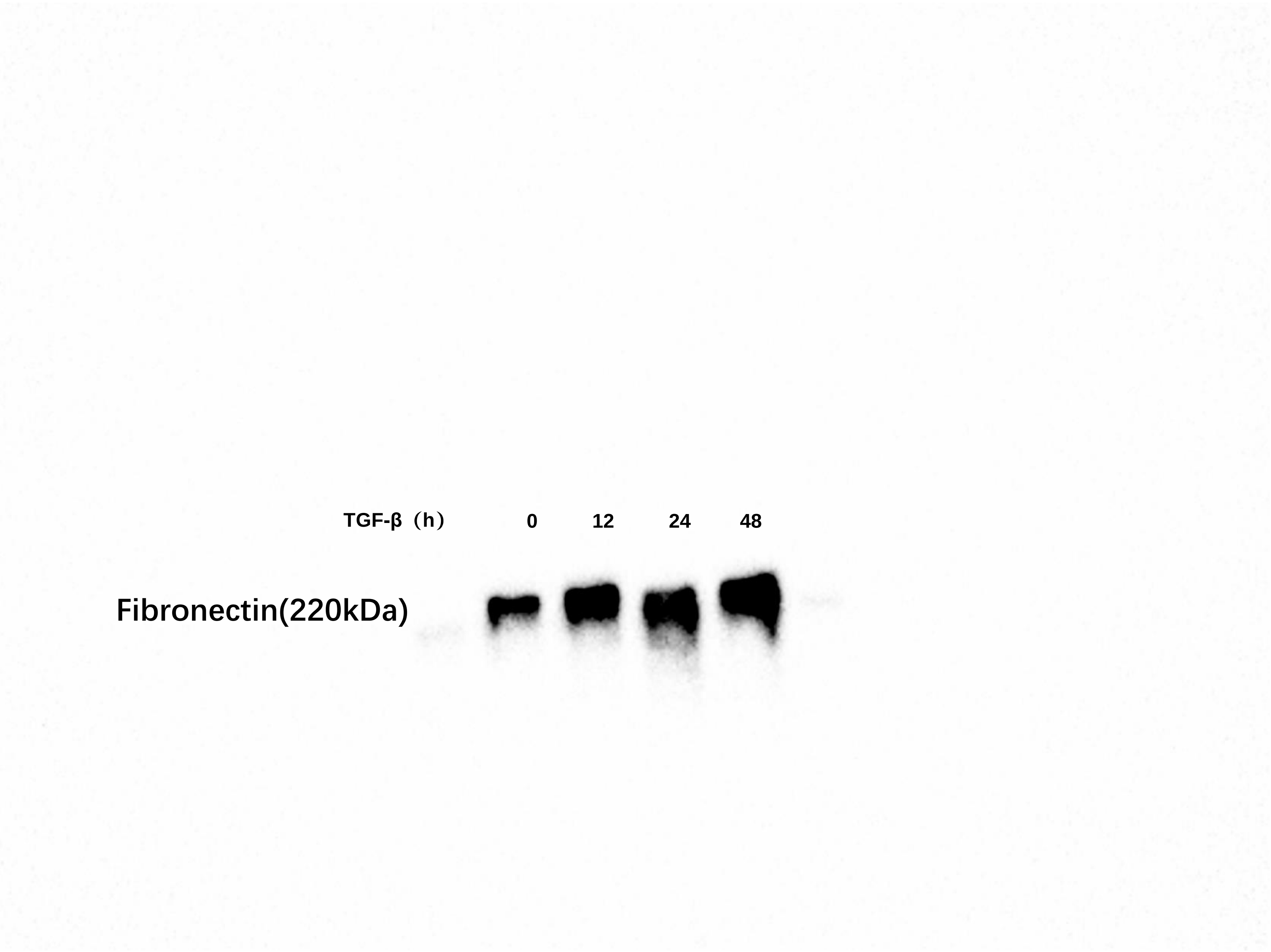

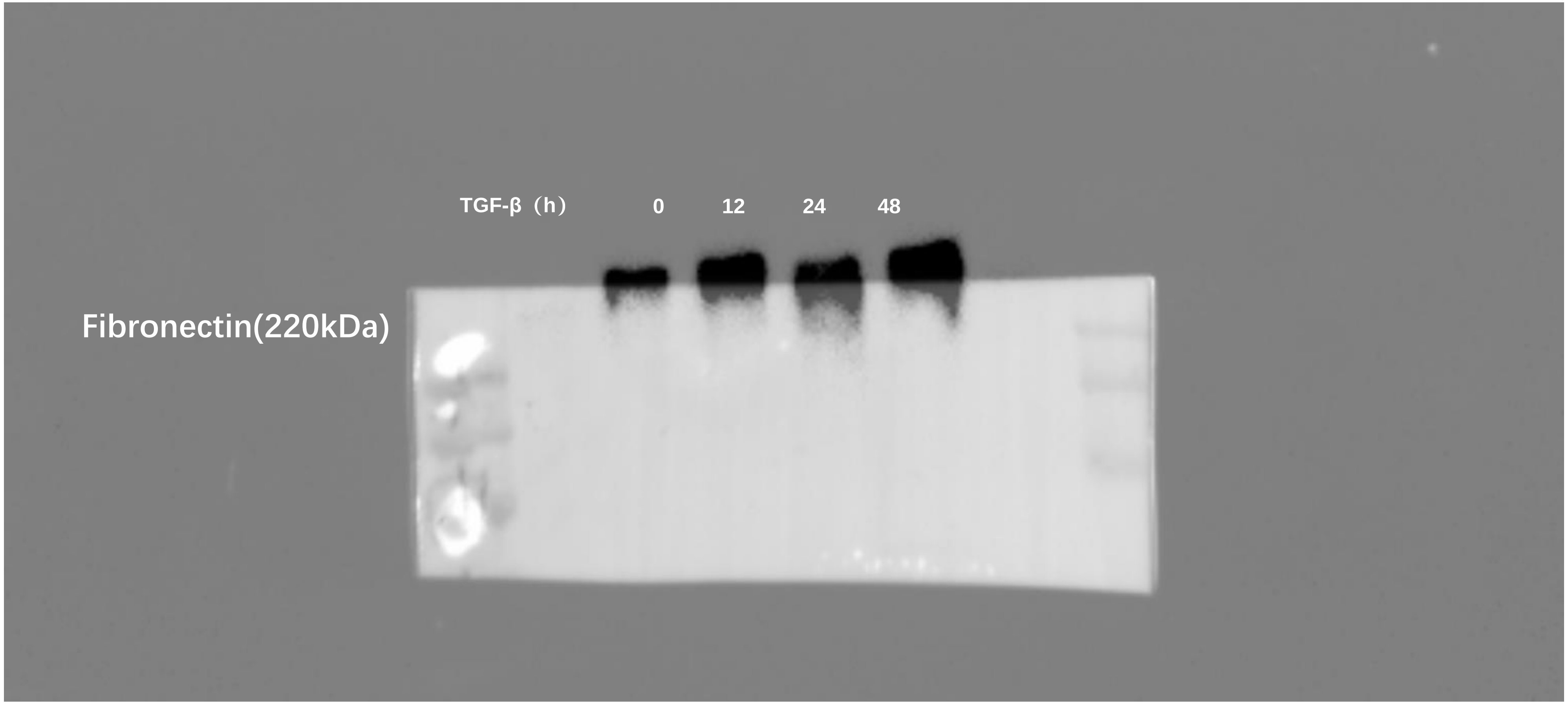
TGF-β（h）
0
12
24
48
Fibronectin(220kDa)

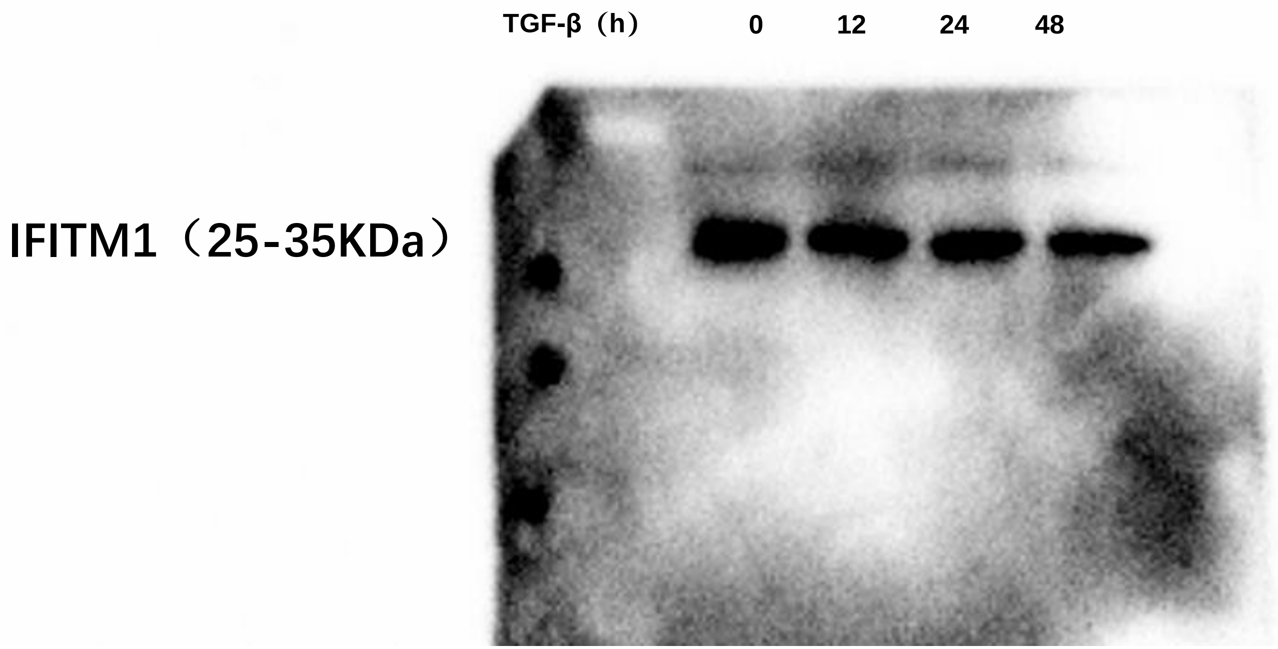
TGF-β（h）
0
12
24
48
IFITM1（25-35KDa）

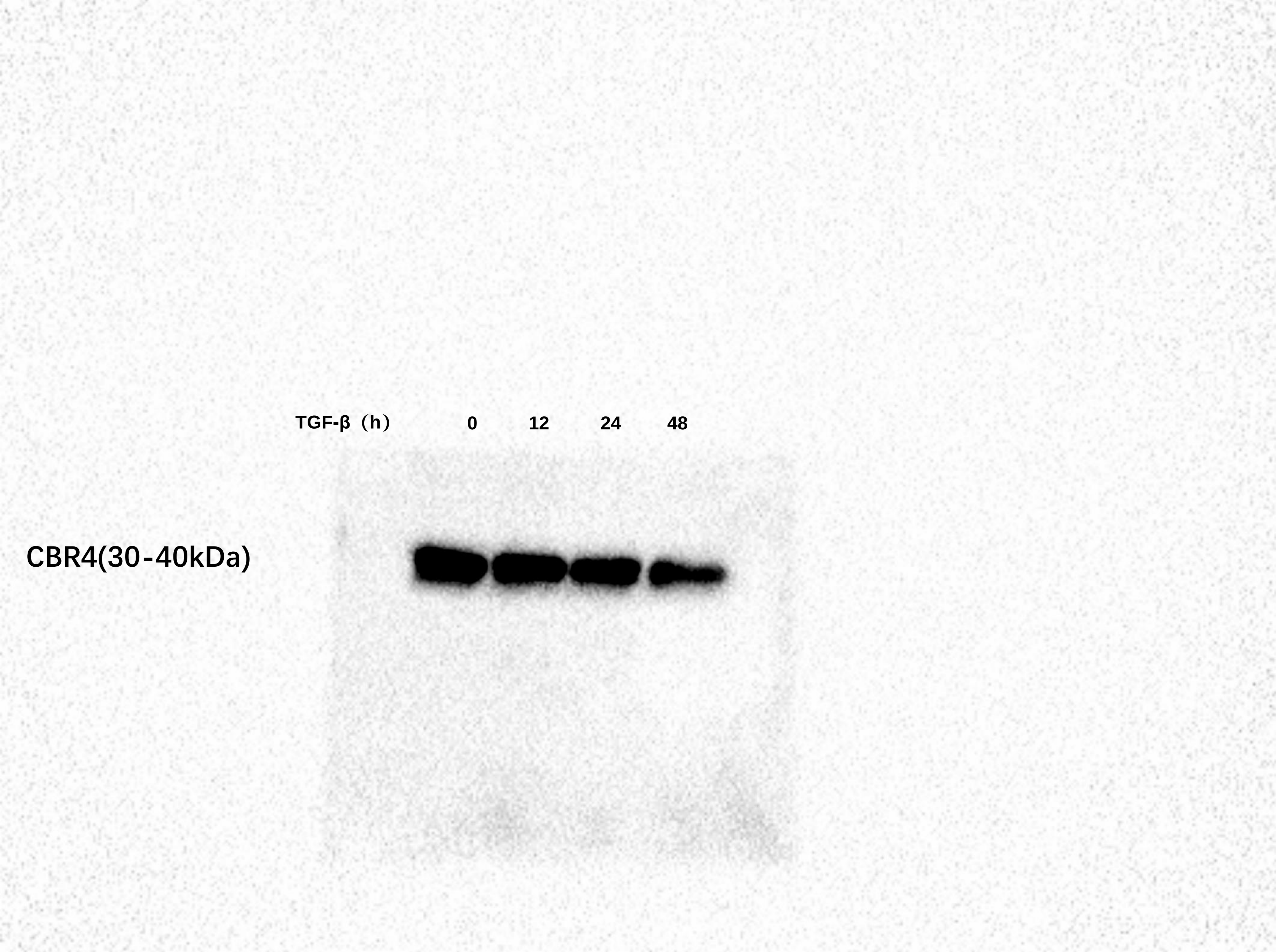
TGF-β（h）
0
12
24
48
CBR4(30-40kDa)

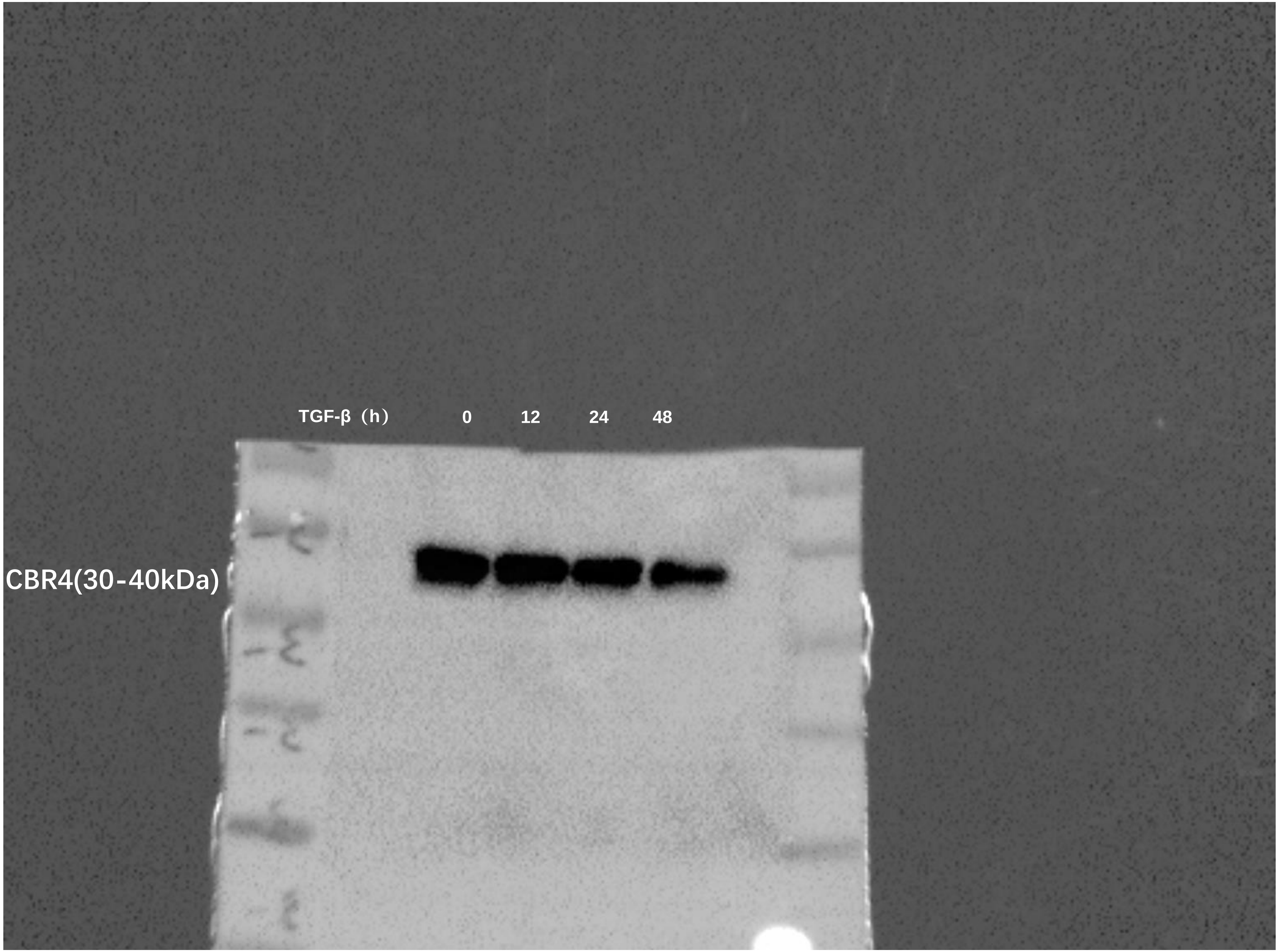
TGF-β（h）
0
12
24
48
CBR4(30-40kDa)

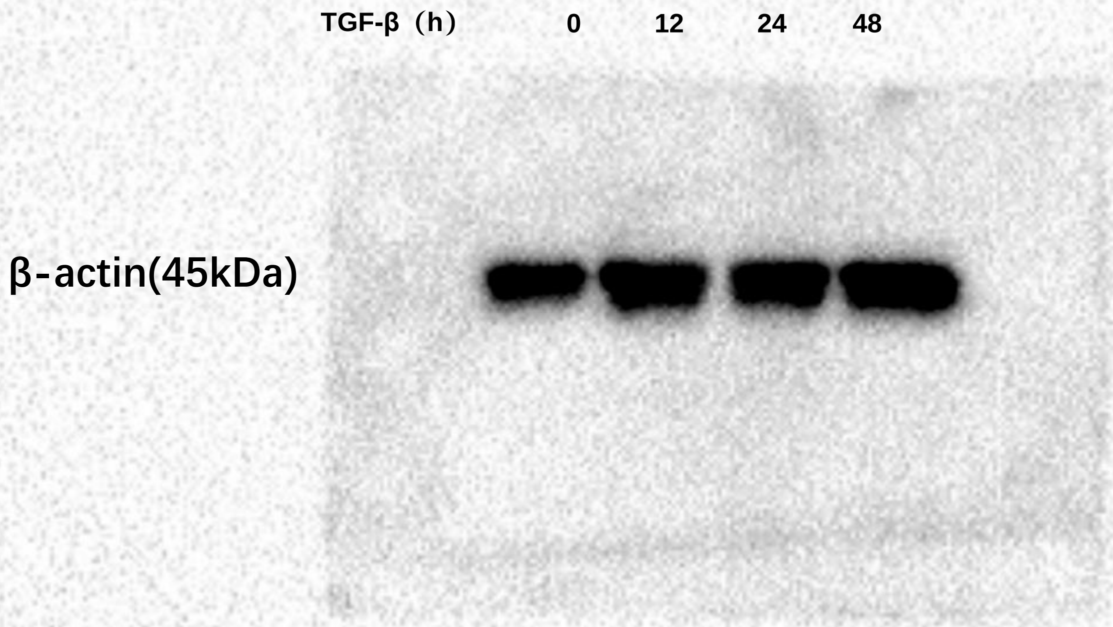
TGF-β（h） 0 12 24 48
β-actin(45kDa)

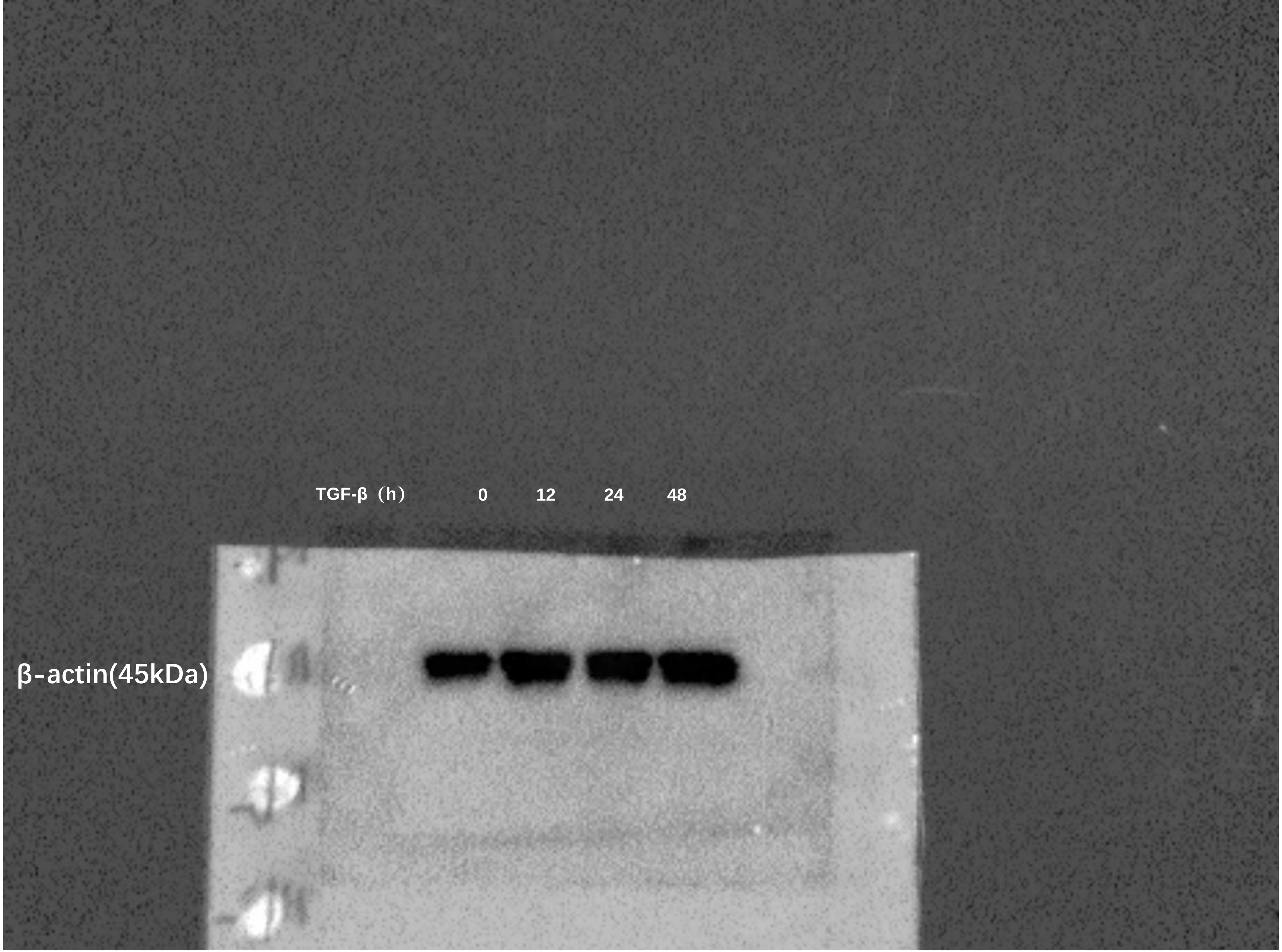
TGF-β（h）
0
12
24
48
β-actin(45kDa)

figure7-B-3

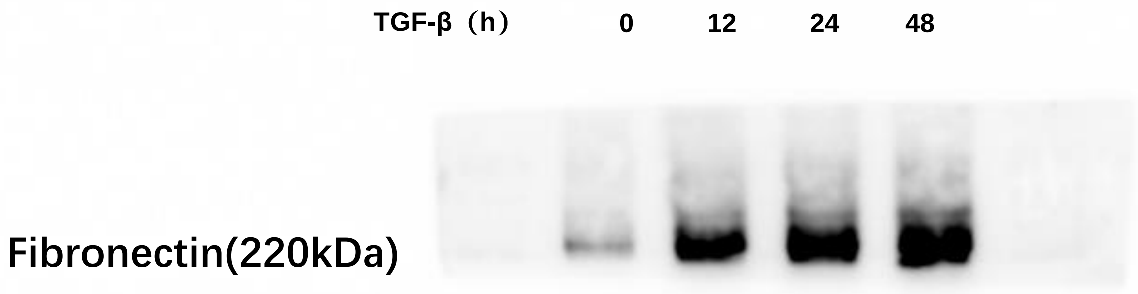

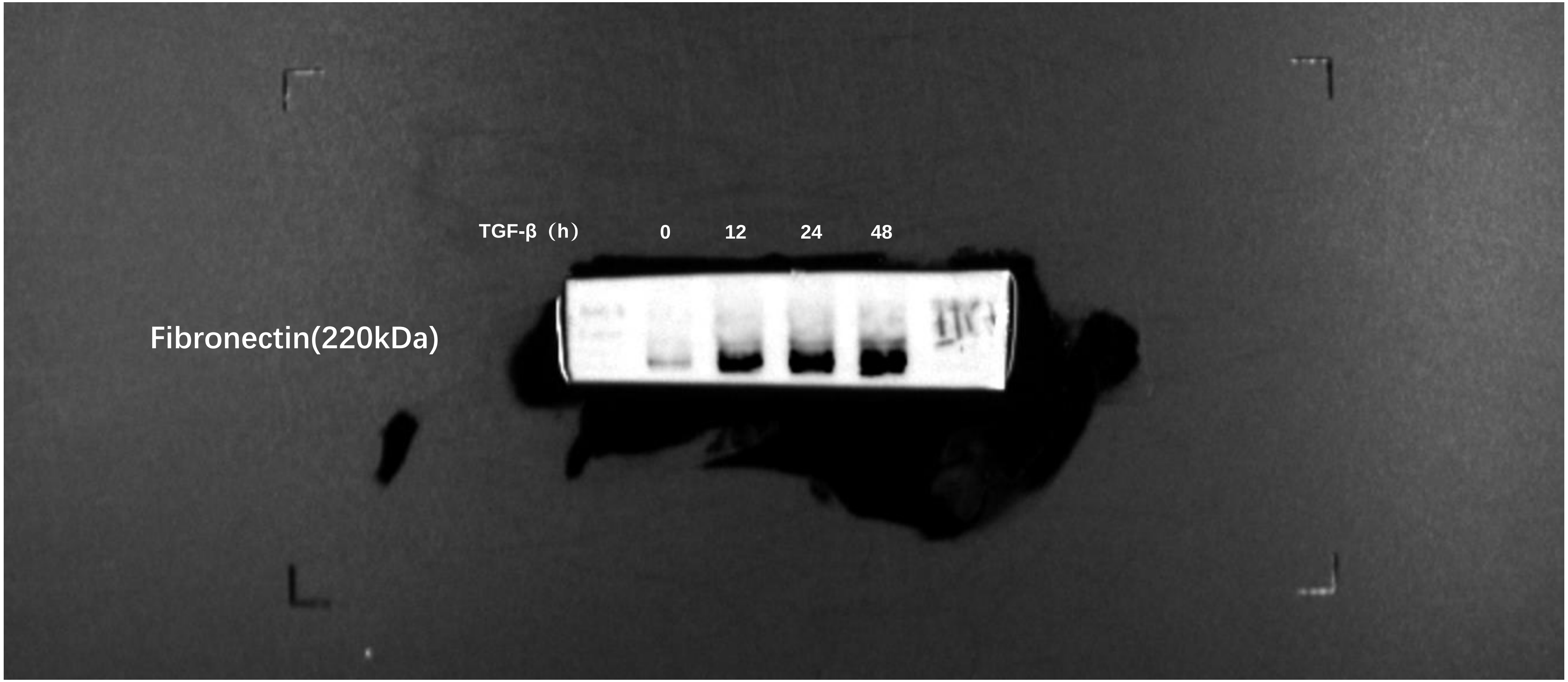
TGF-β（h）
0
12
24
48
Fibronectin(220kDa)

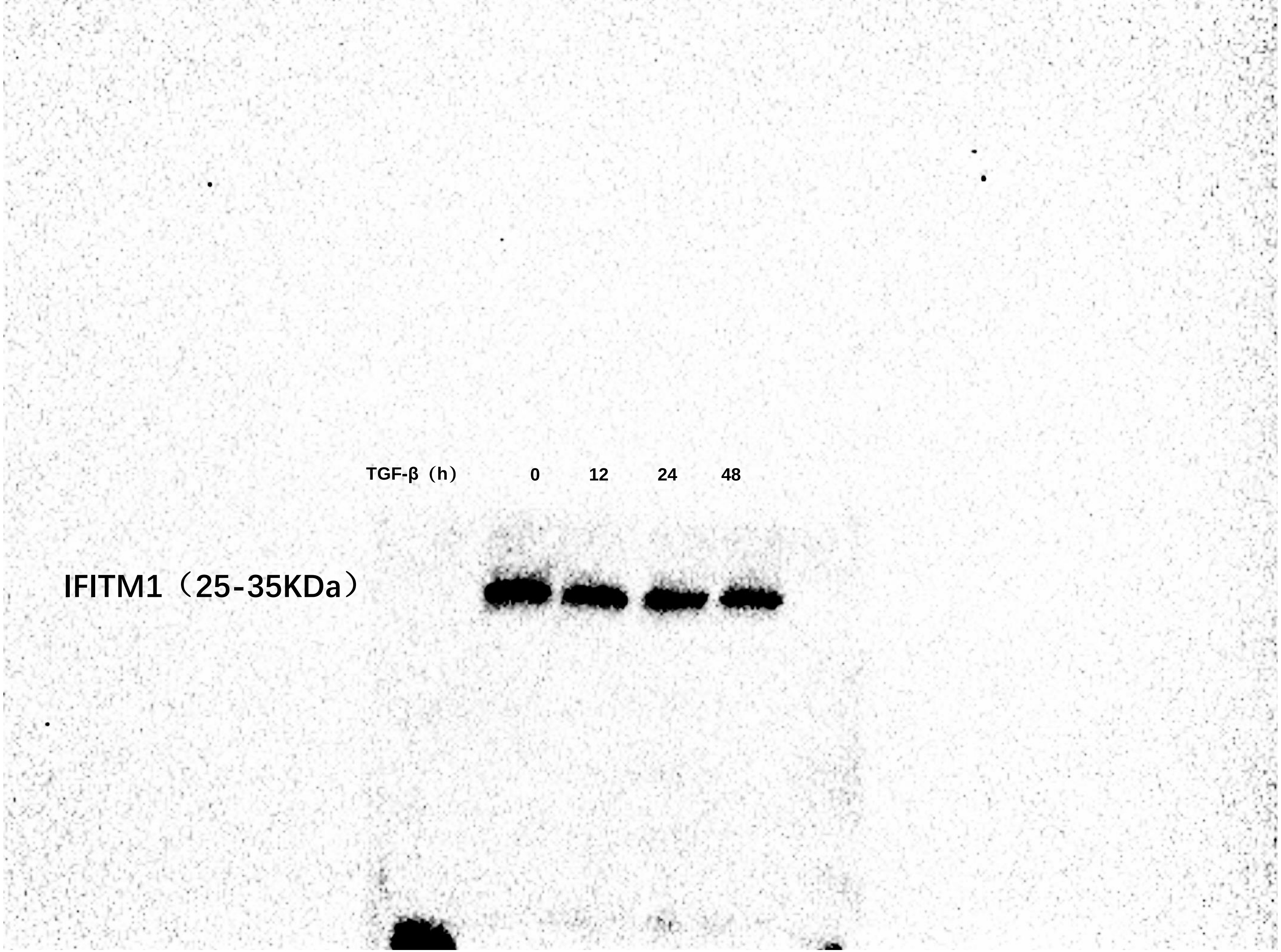
TGF-β（h）
0
12
24
48
IFITM1（25-35KDa）

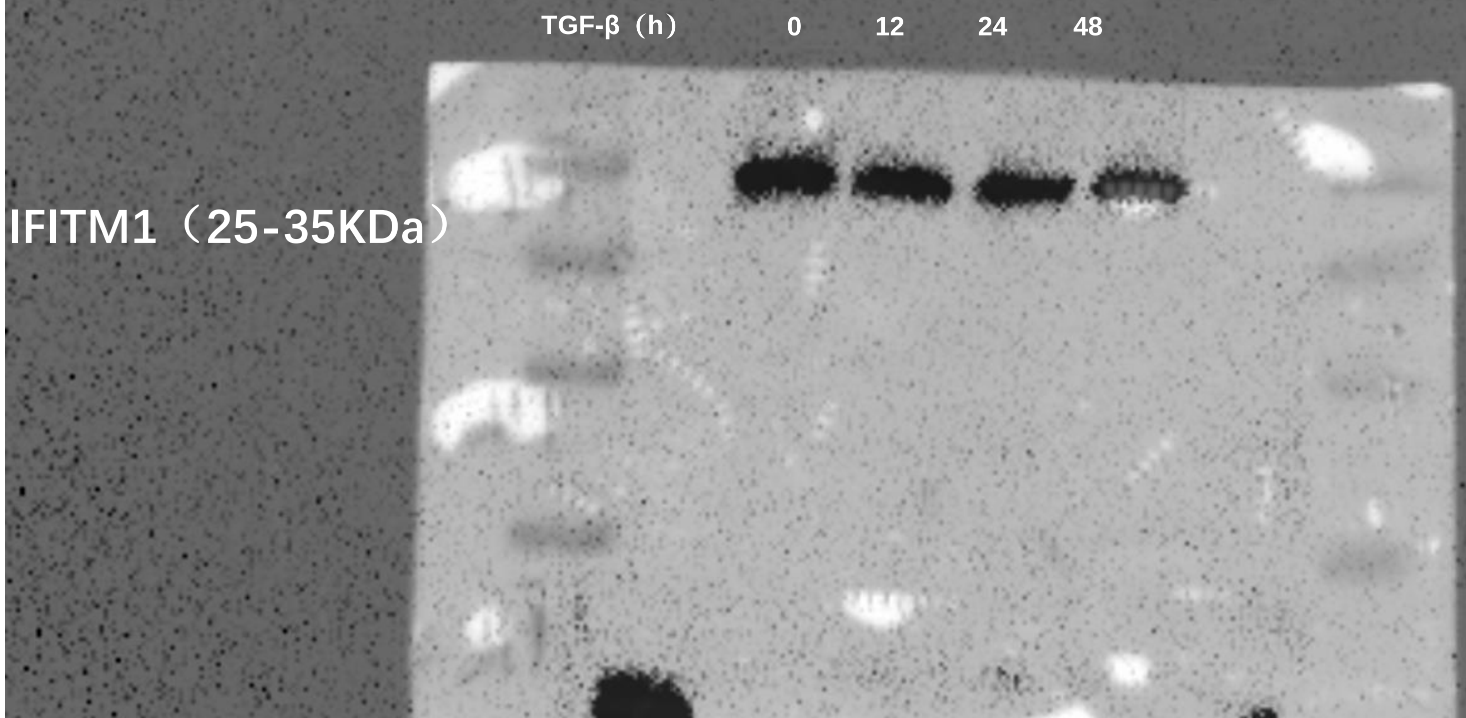
TGF-β（h）
0
12
24
48
IFITM1（25-35KDa）

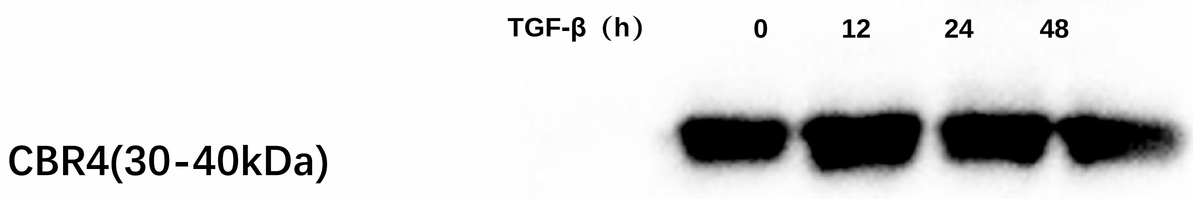
TGF-β（h）
0
12
24
48
CBR4(30-40kDa)

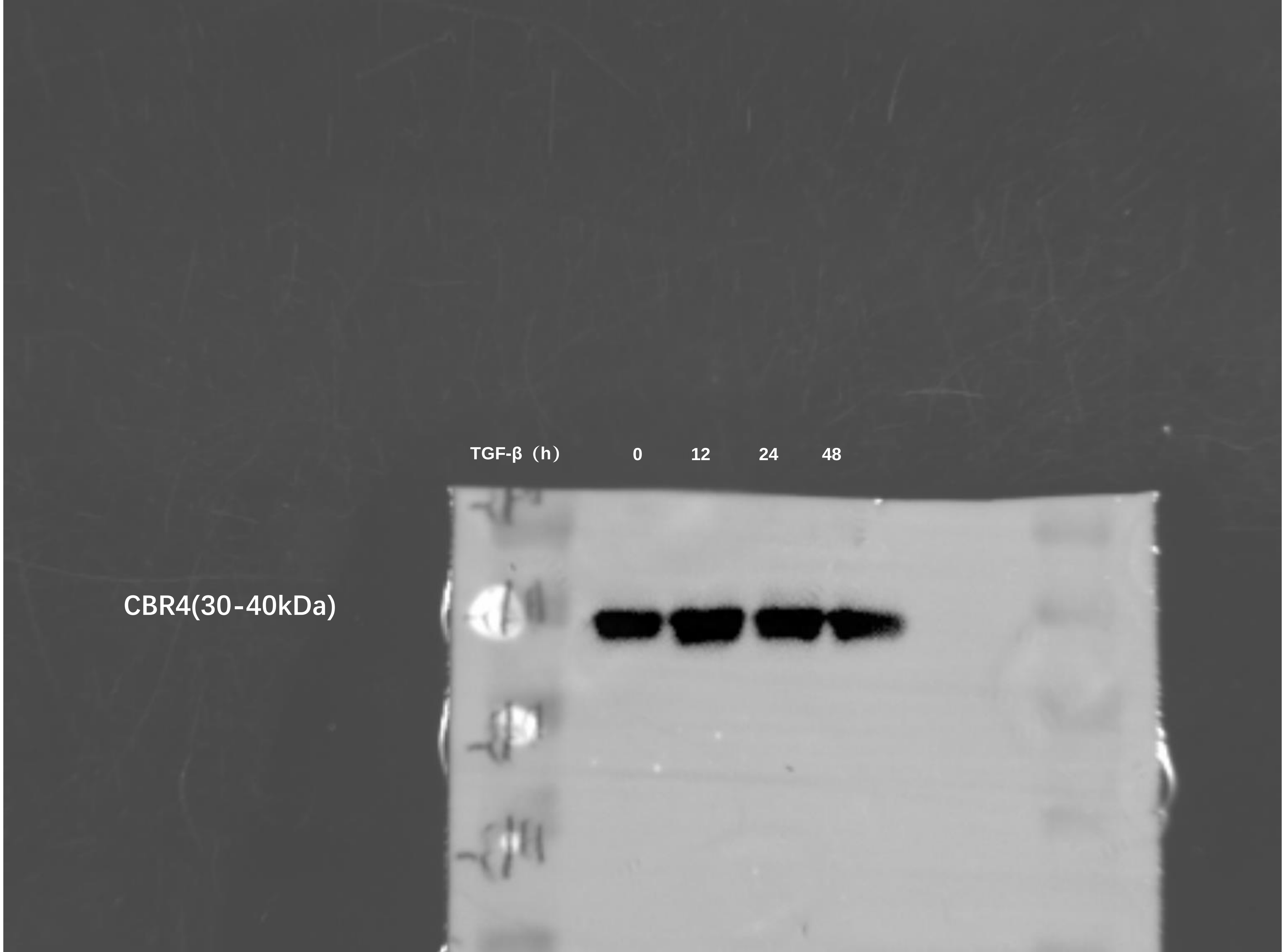
TGF-β（h）
0
12
24
48
CBR4(30-40kDa)

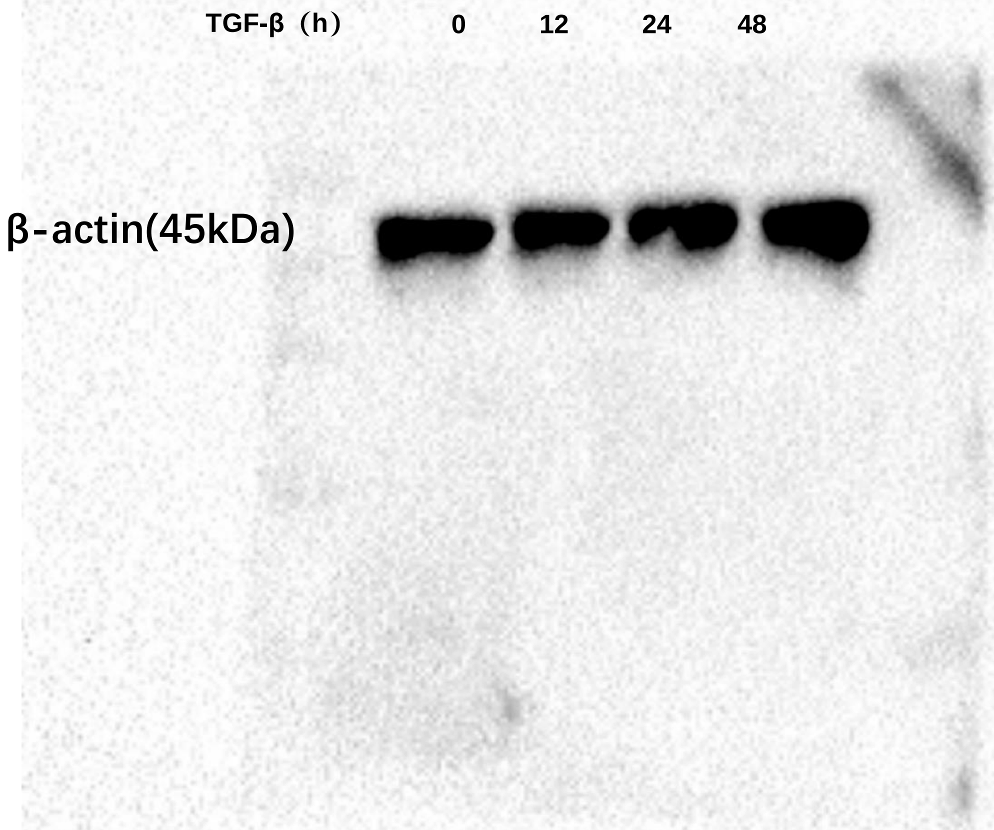
TGF-β（h）
0
12
24
48
β-actin(45kDa)

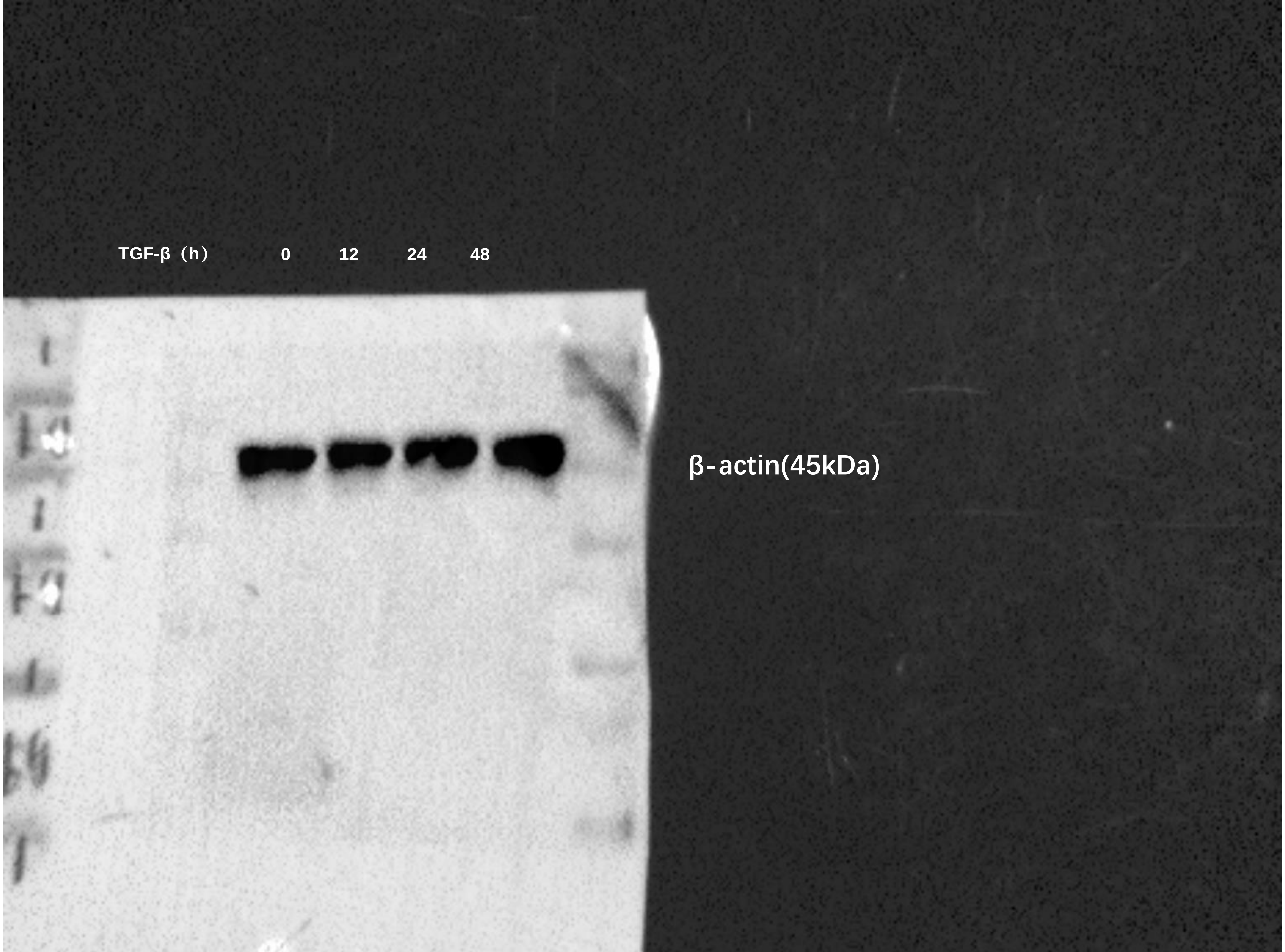
TGF-β（h）
0
12
24
48
β-actin(45kDa)

figure7-E-1

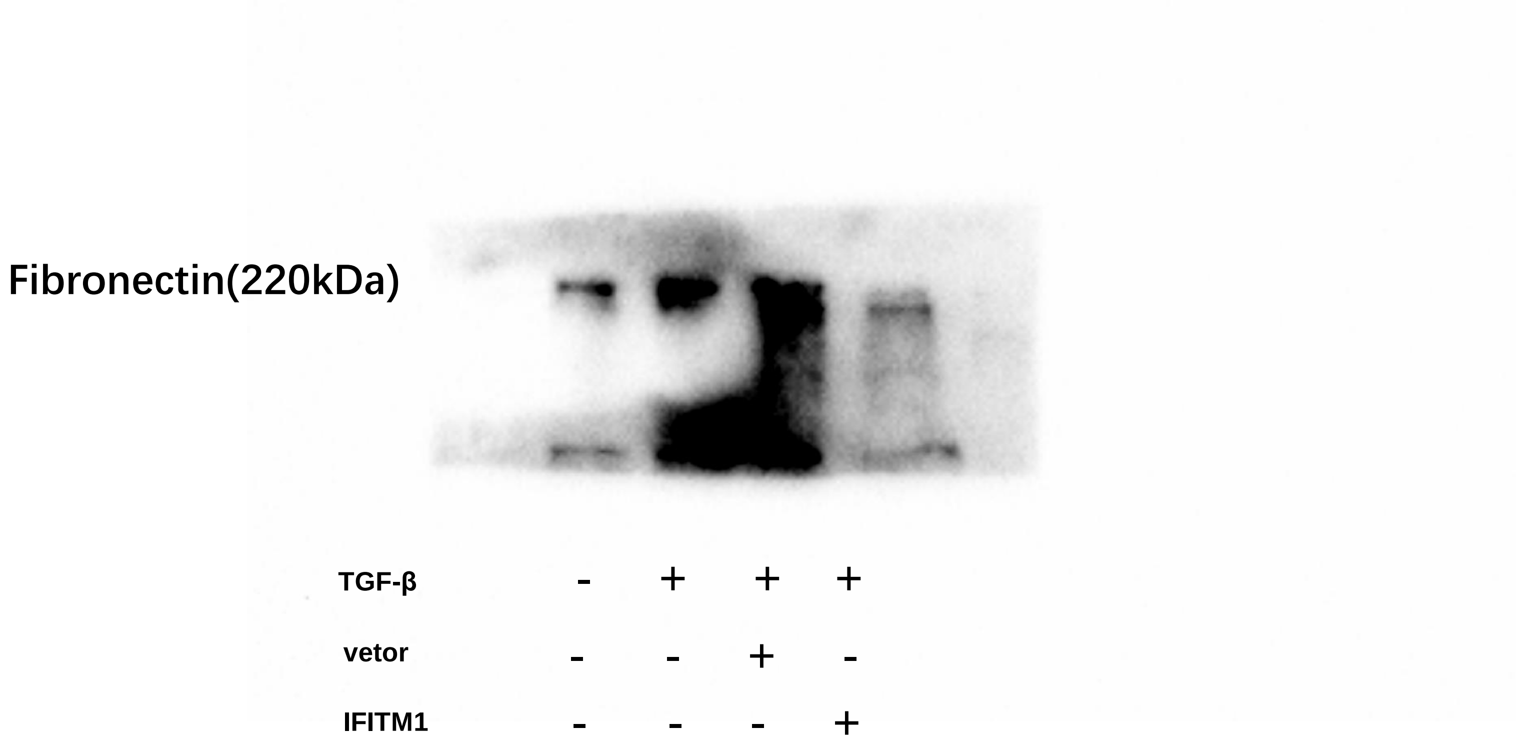

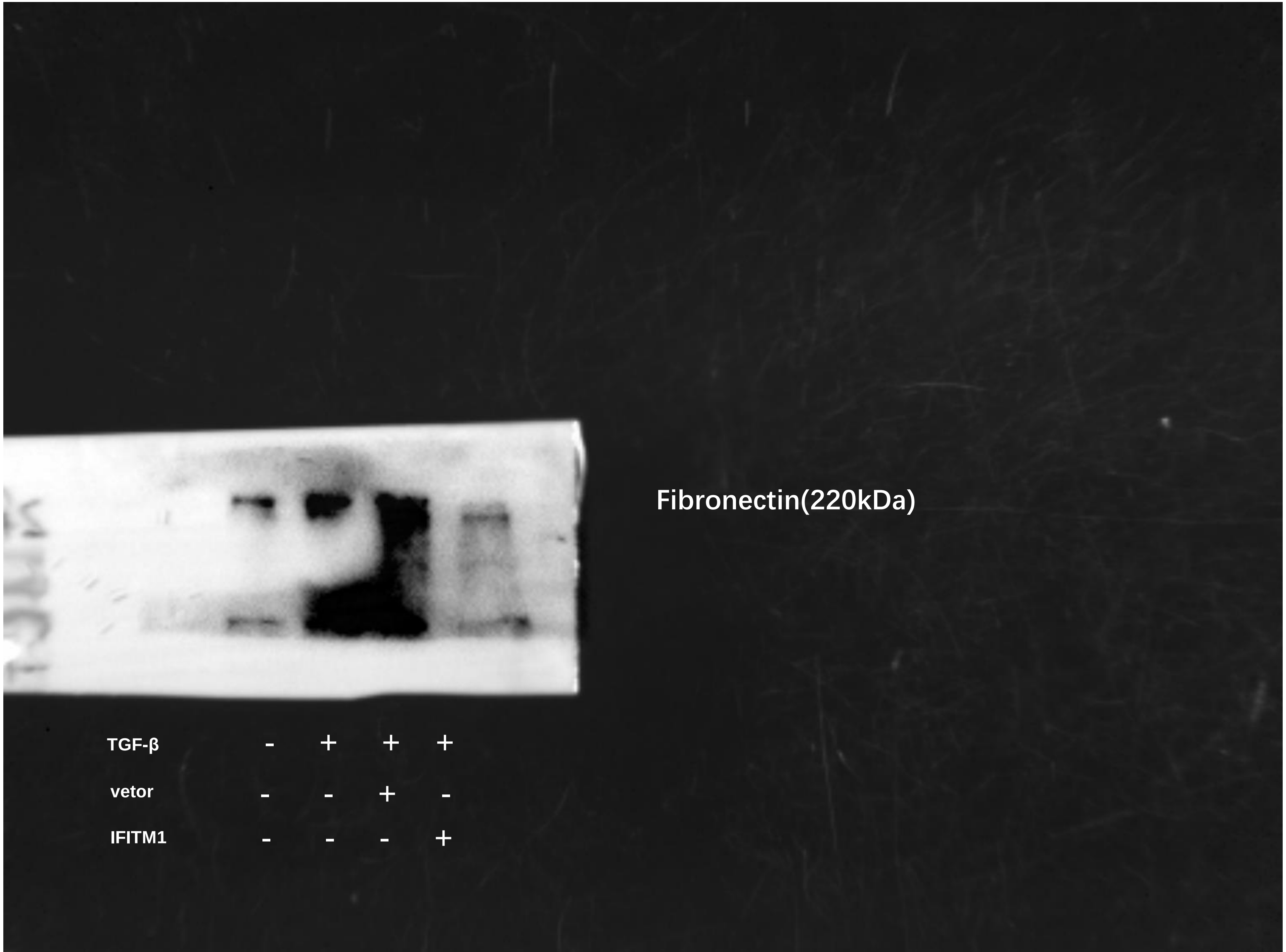
Fibronectin(220kDa)
TGF-β - + + +
vetor - - + -
IFITM1 - - - +

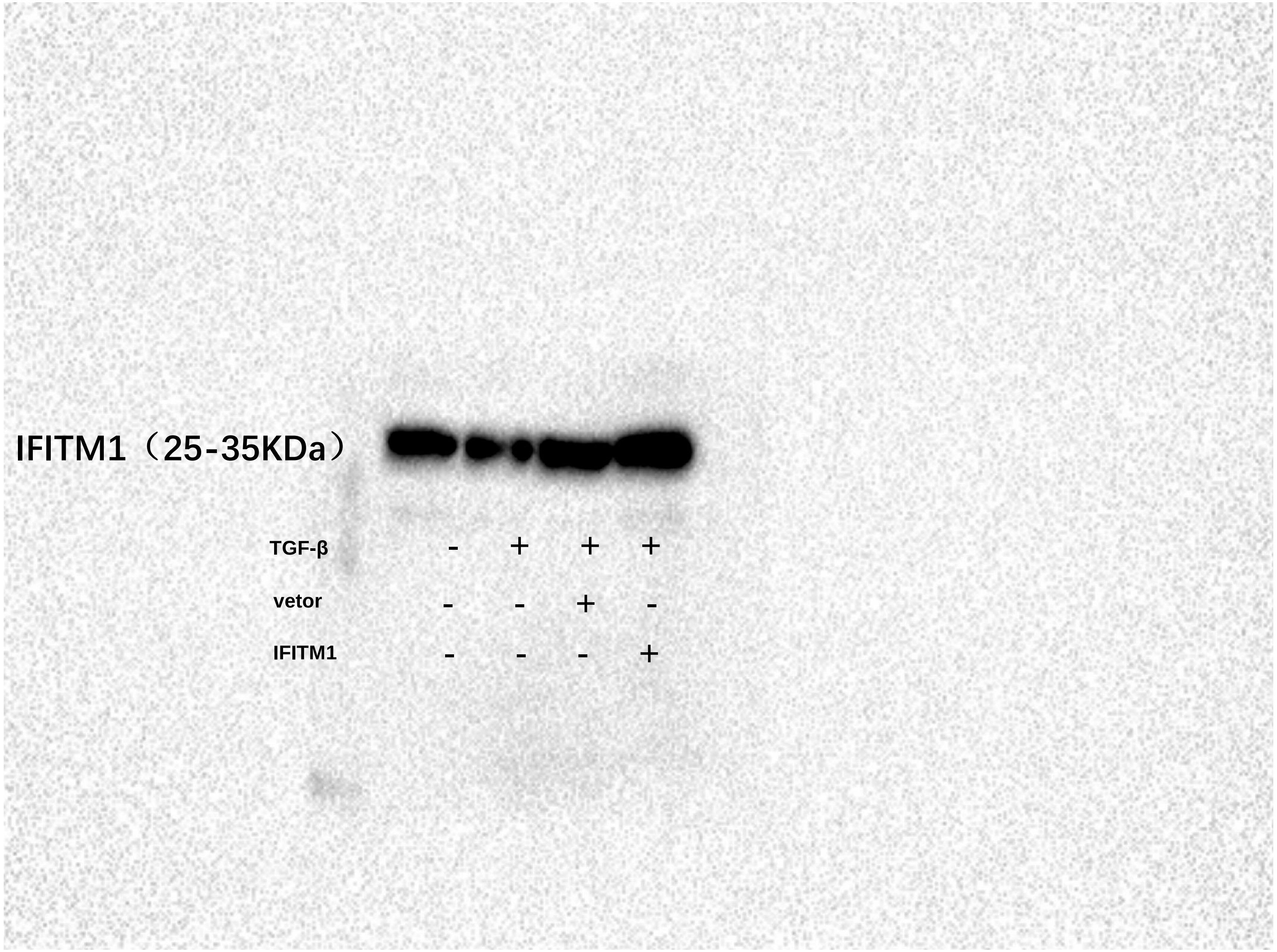
IFITM1（25-35KDa）
TGF-β - + + +
vetor - - + -
IFITM1 - - - +

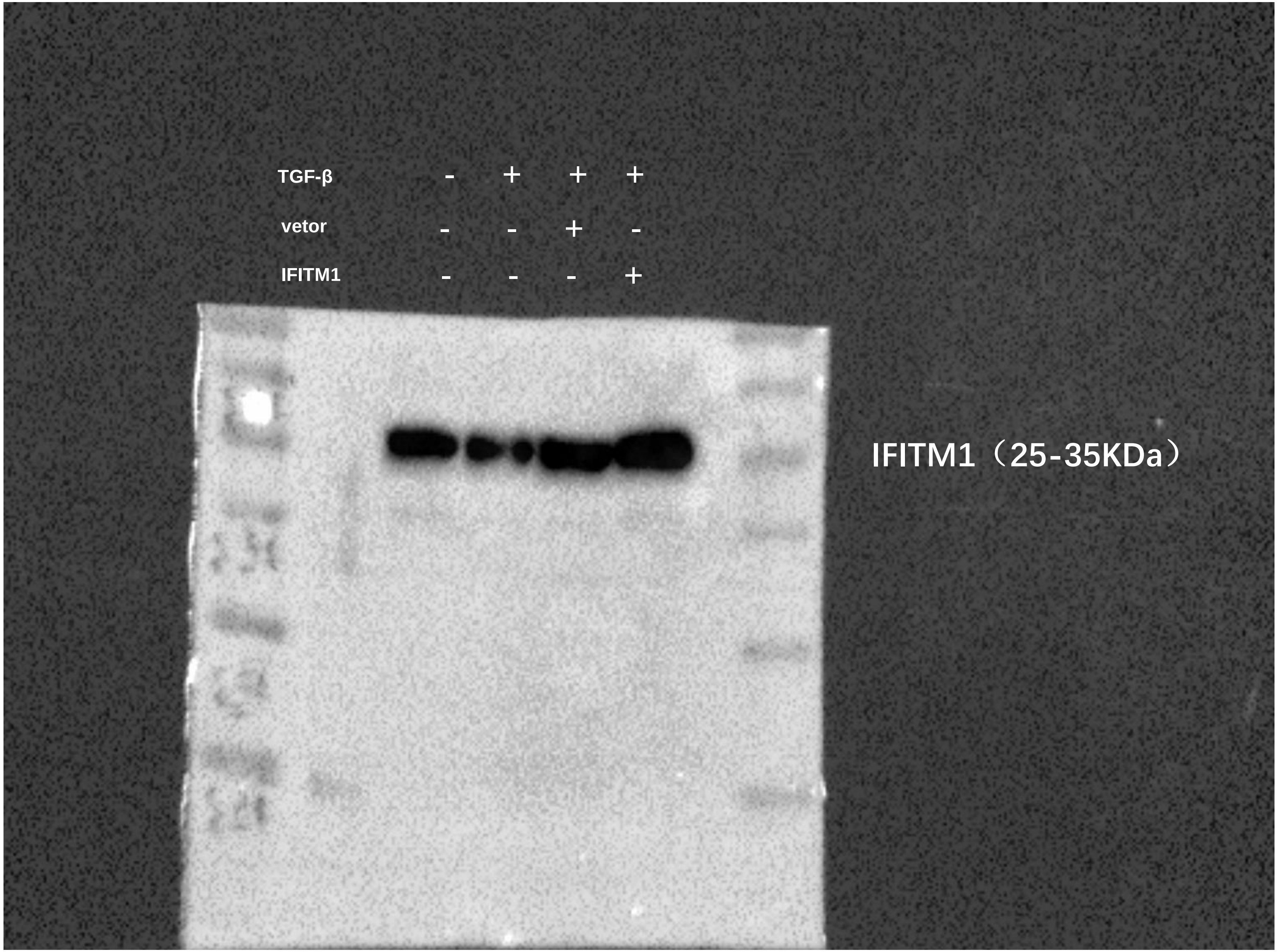
TGF-β - + + +
vetor - - + -
IFITM1 - - - +
IFITM1（25-35KDa）

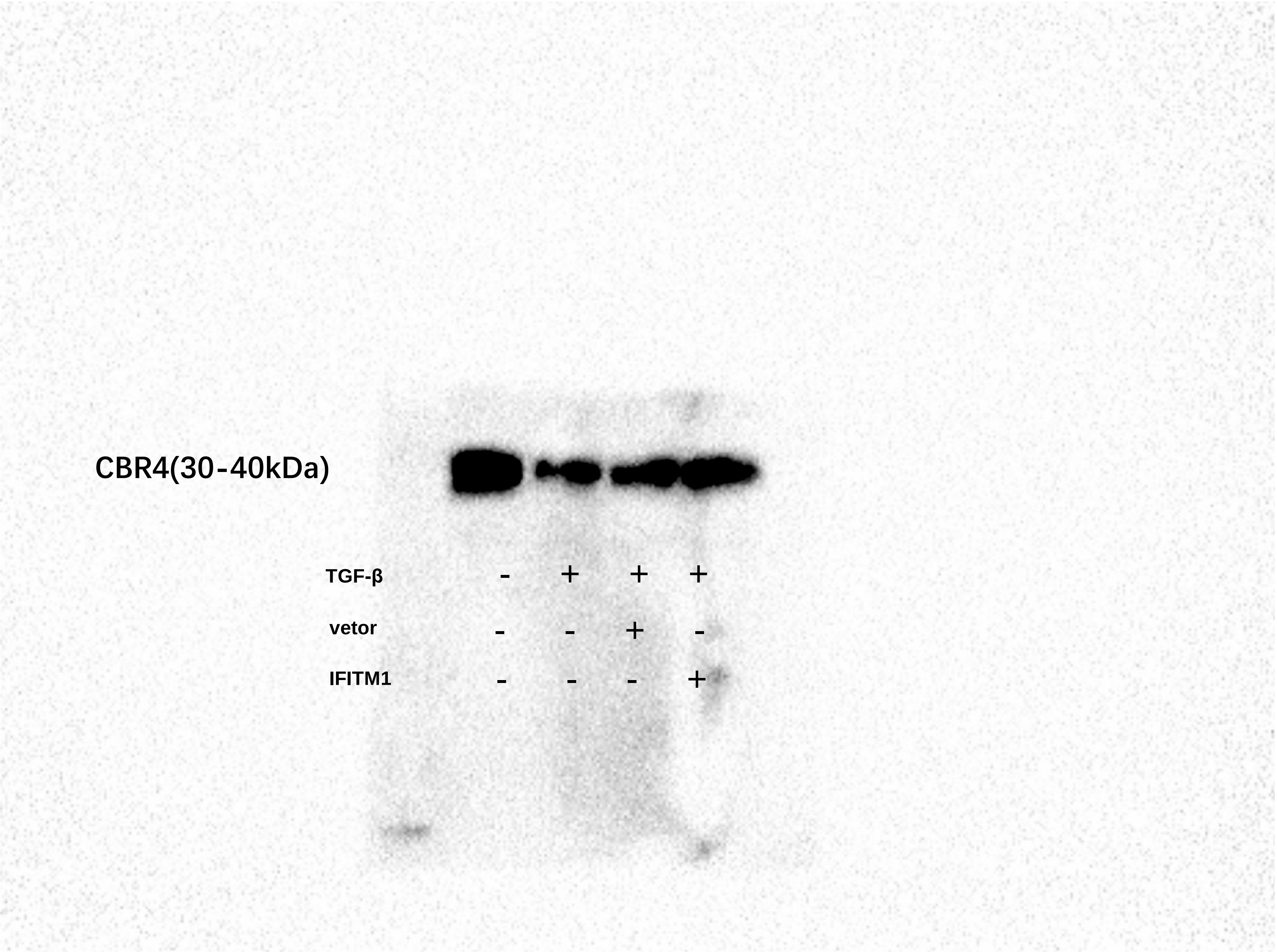
CBR4(30-40kDa)
TGF-β - + + +
vetor - - + -
IFITM1 - - - +

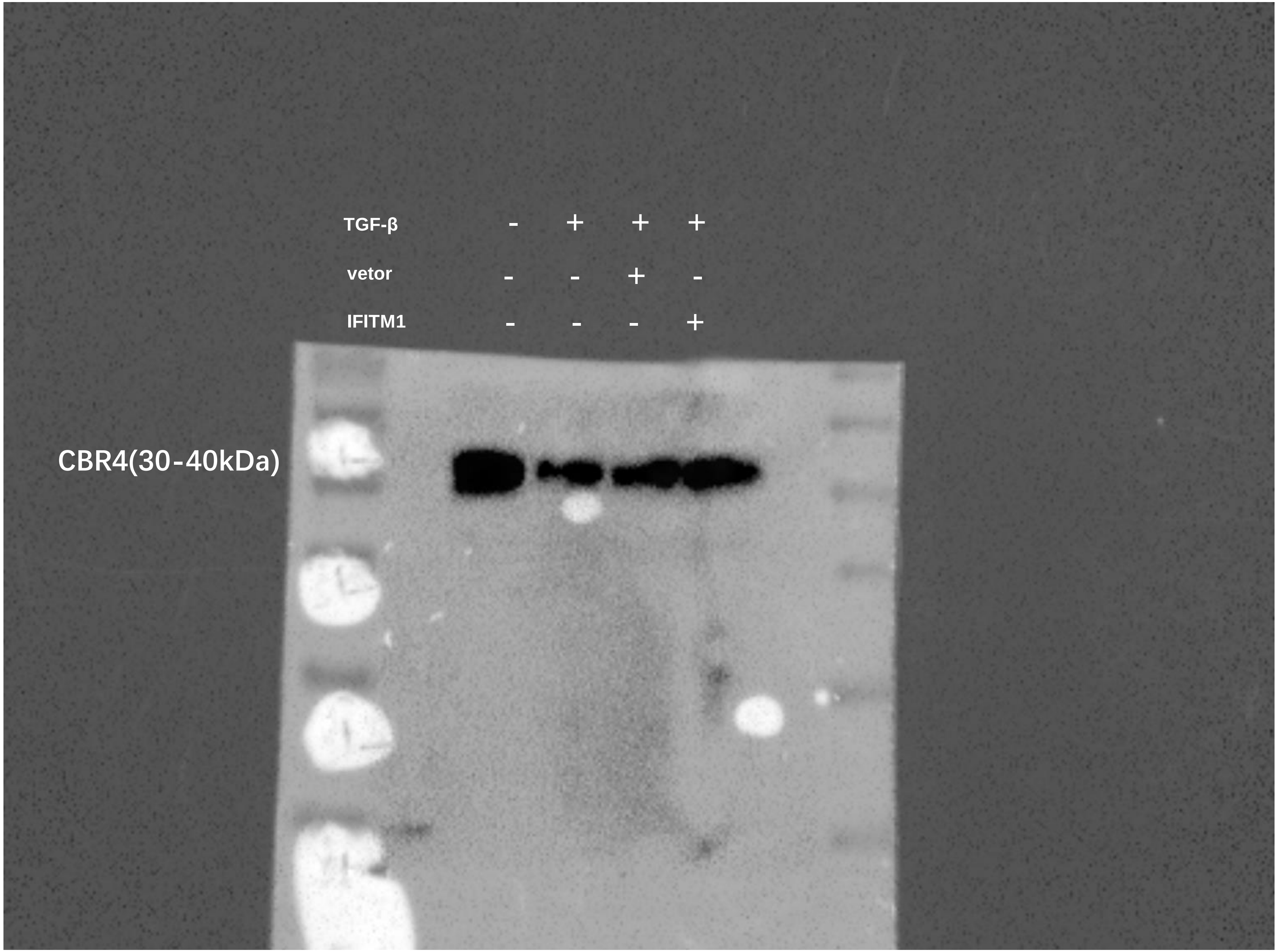
TGF-β - + + +
vetor - - + -
IFITM1 - - - +
CBR4(30-40kDa)

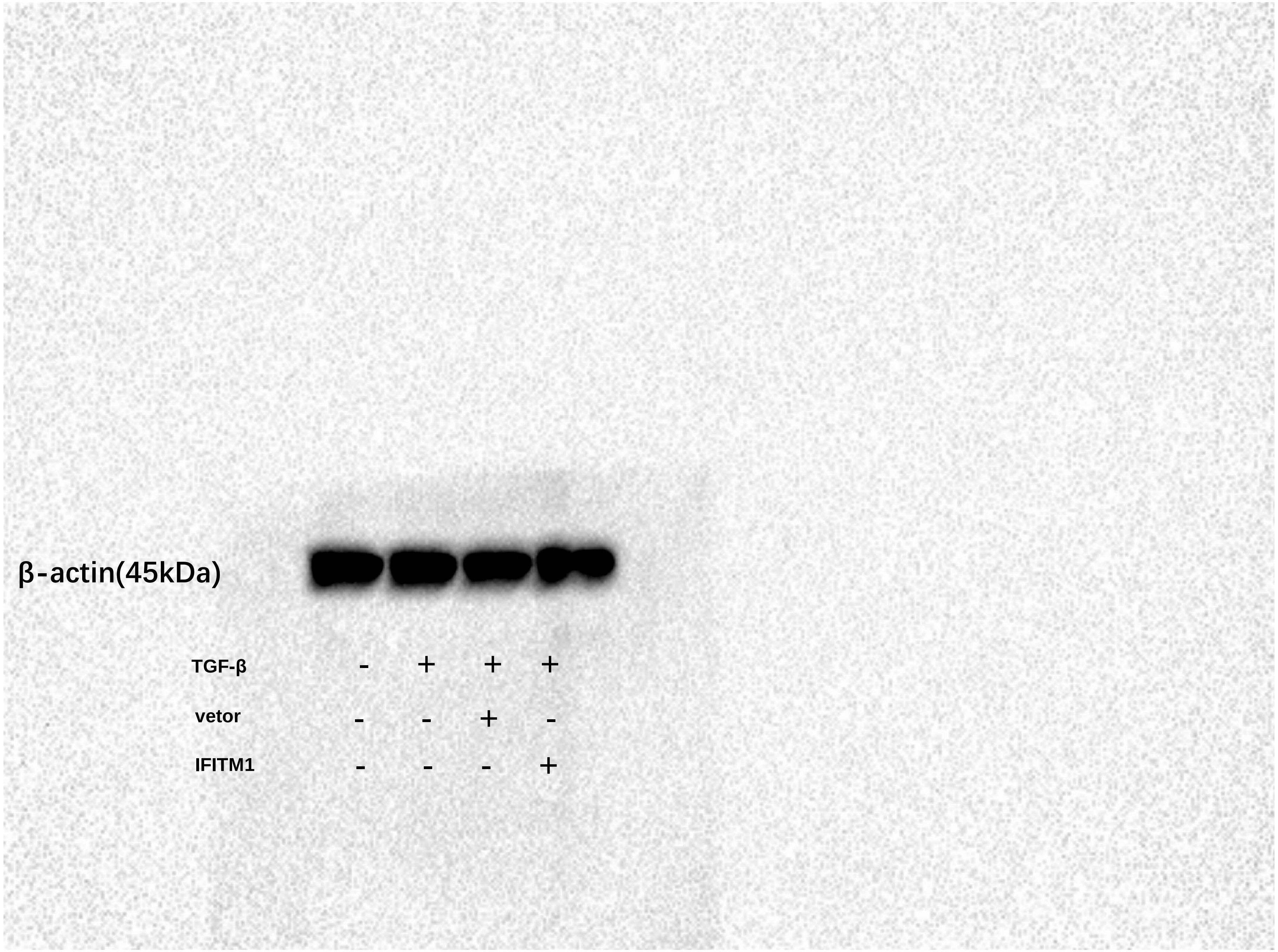
β-actin(45kDa)
TGF-β - + + +
vetor - - + -
IFITM1 - - - +

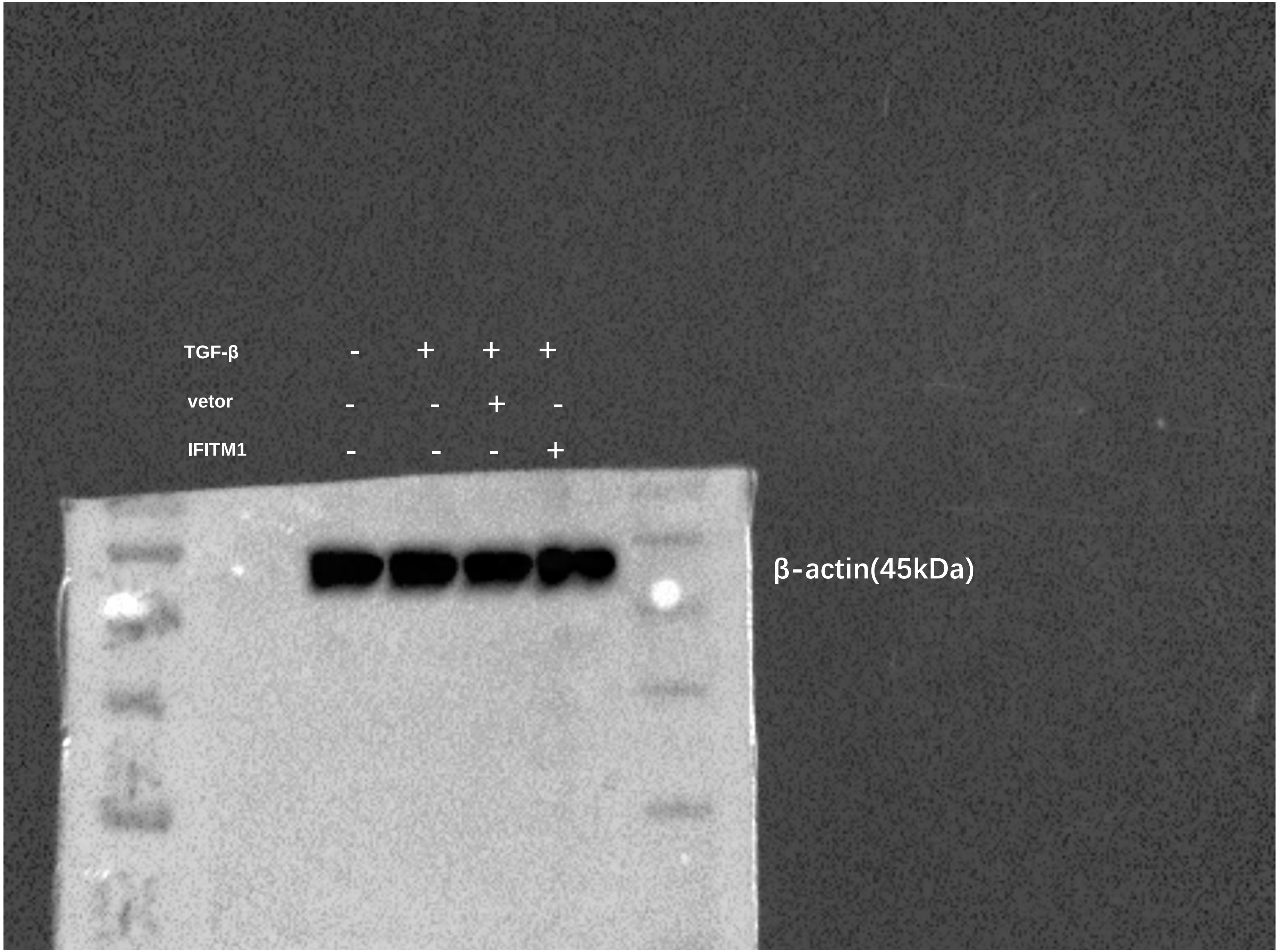

TGF-β - + + +
vetor - - + -
IFITM1 - - - +
β-actin(45kDa)

figure7-E-2

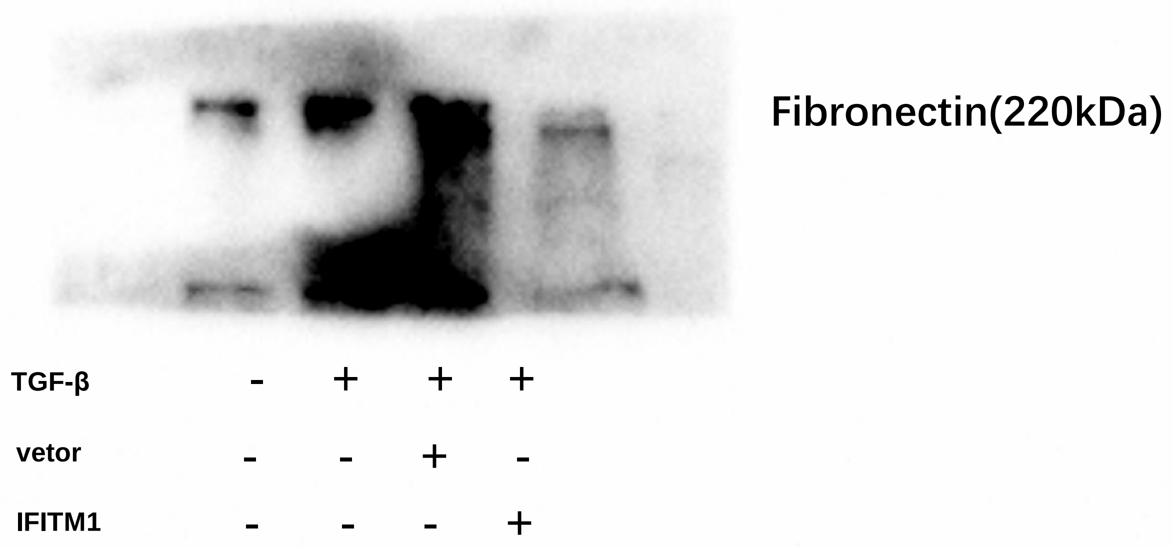

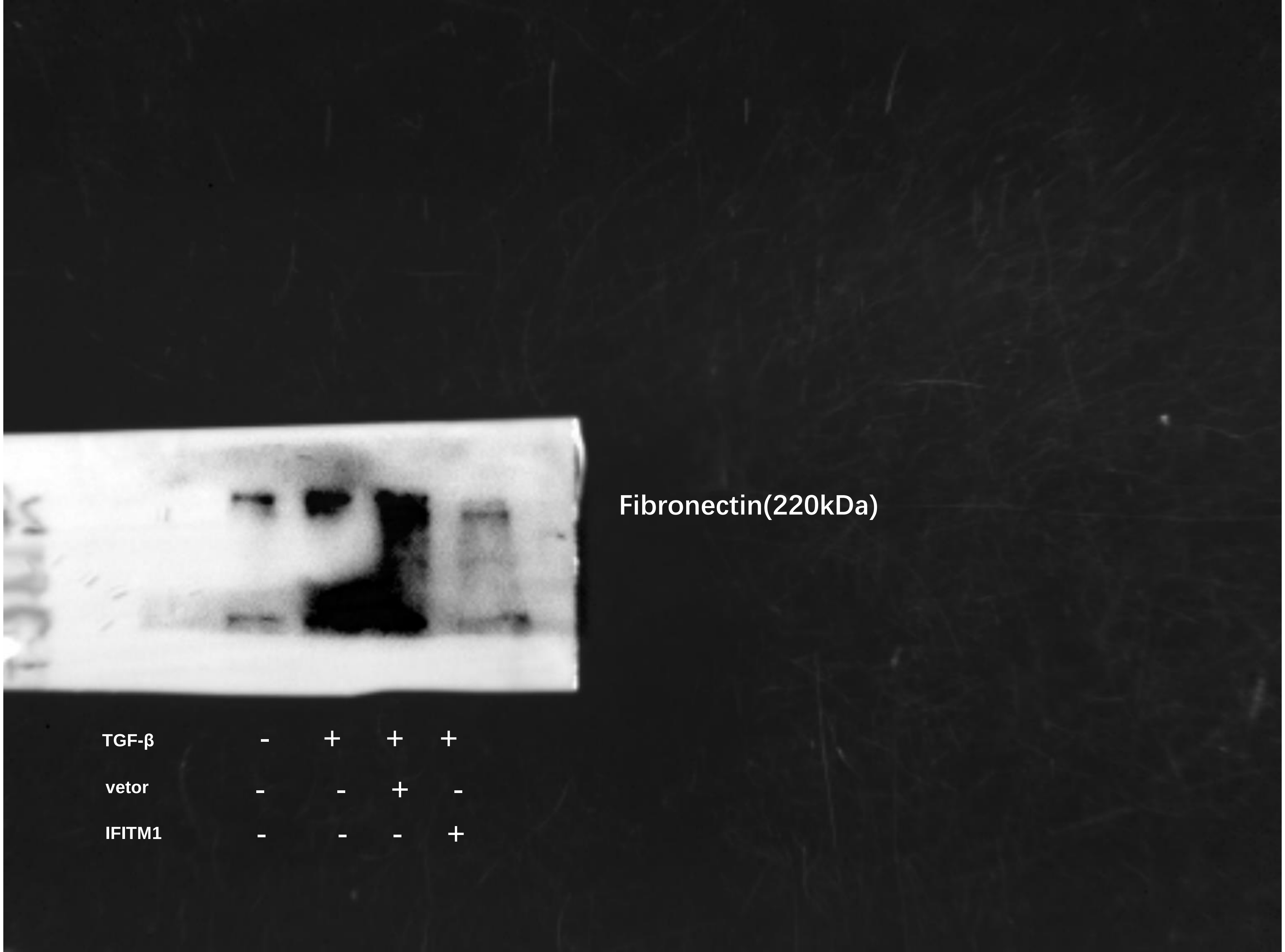
Fibronectin(220kDa)
TGF-β - + + +
vetor - - + -
IFITM1 - - - +

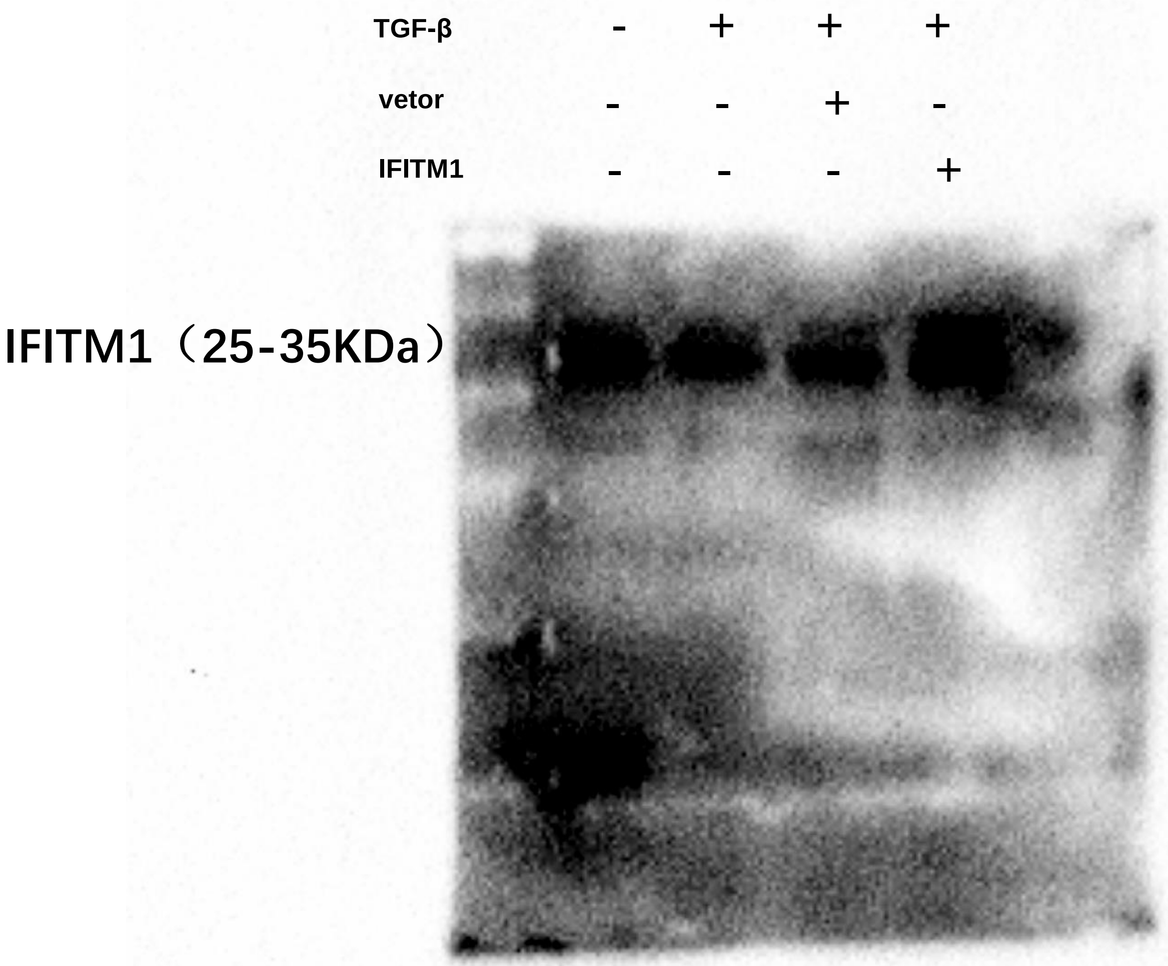
TGF-β - + + +
vetor - - + -
IFITM1 - - - +
IFITM1（25-35KDa）

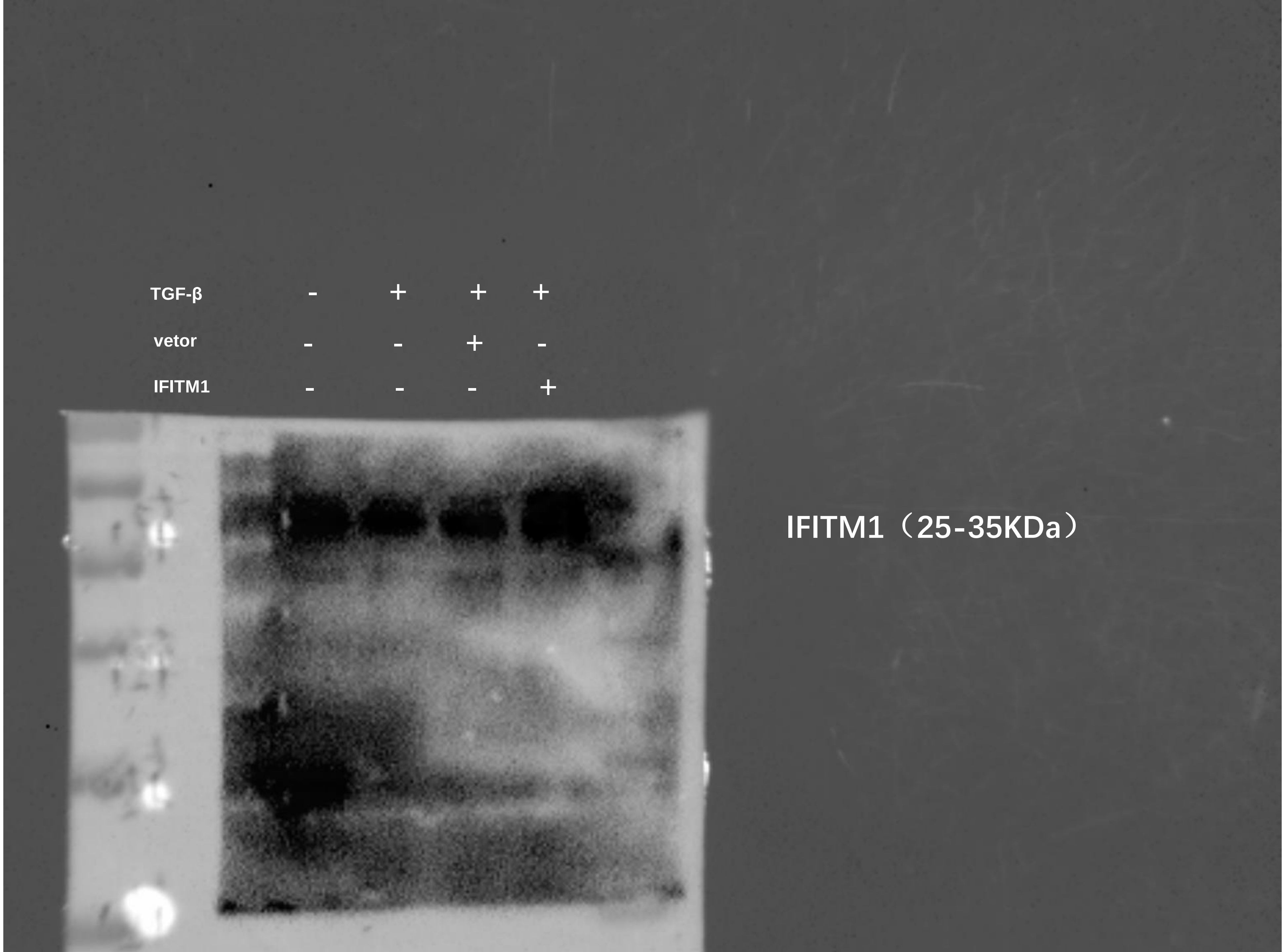
TGF-β - + + +
vetor - - + -
IFITM1 - - - +
IFITM1（25-35KDa）

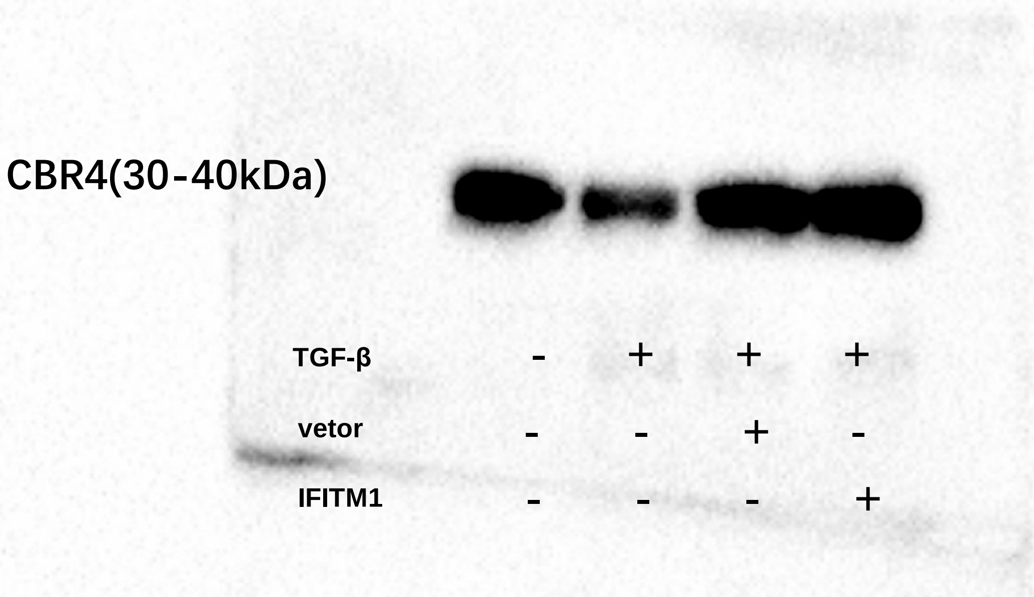
CBR4(30-40kDa)
TGF-β - + + +
vetor - - + -
IFITM1 - - - +

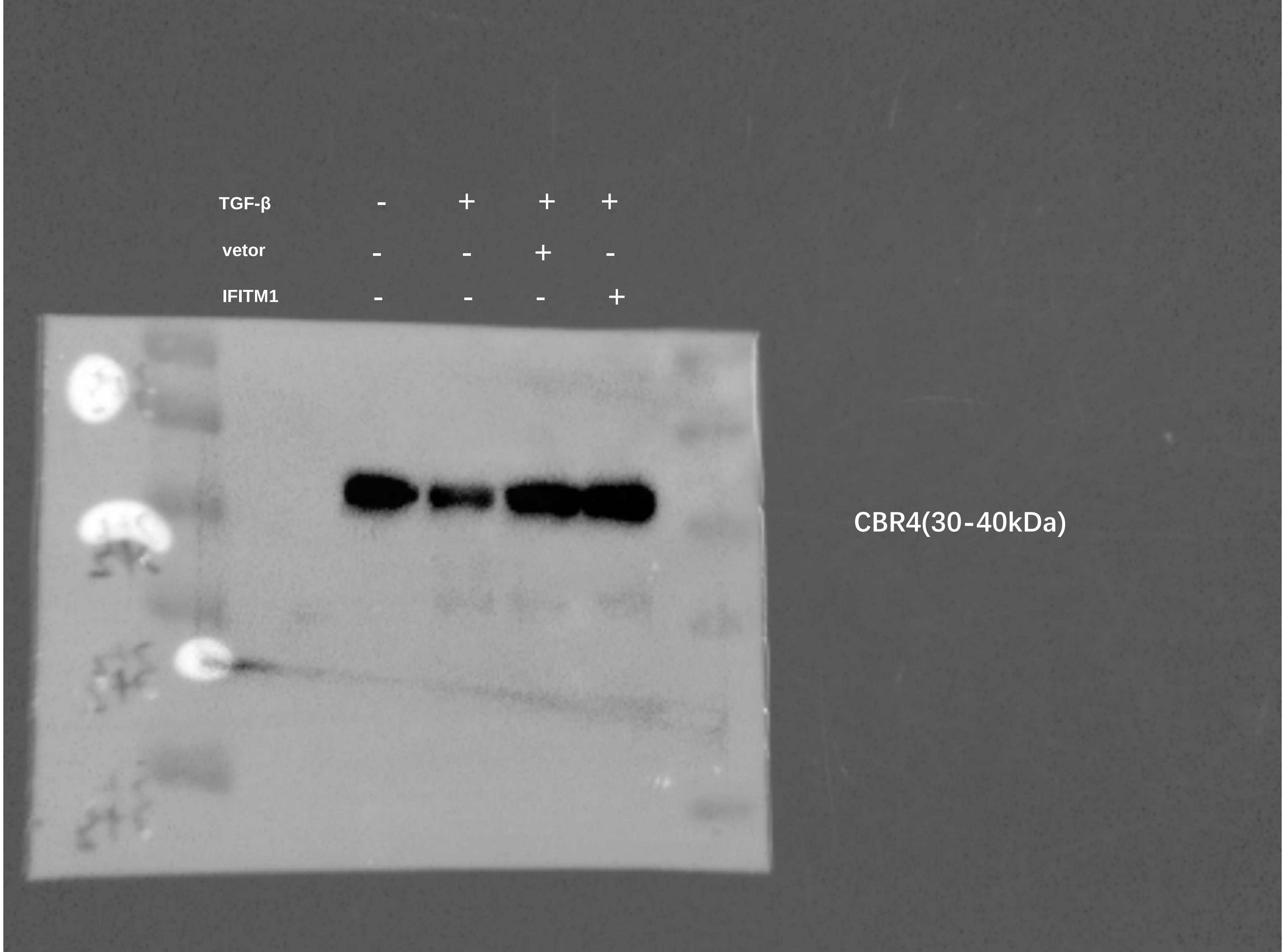
TGF-β - + + +
vetor - - + -
IFITM1 - - - +
CBR4(30-40kDa)

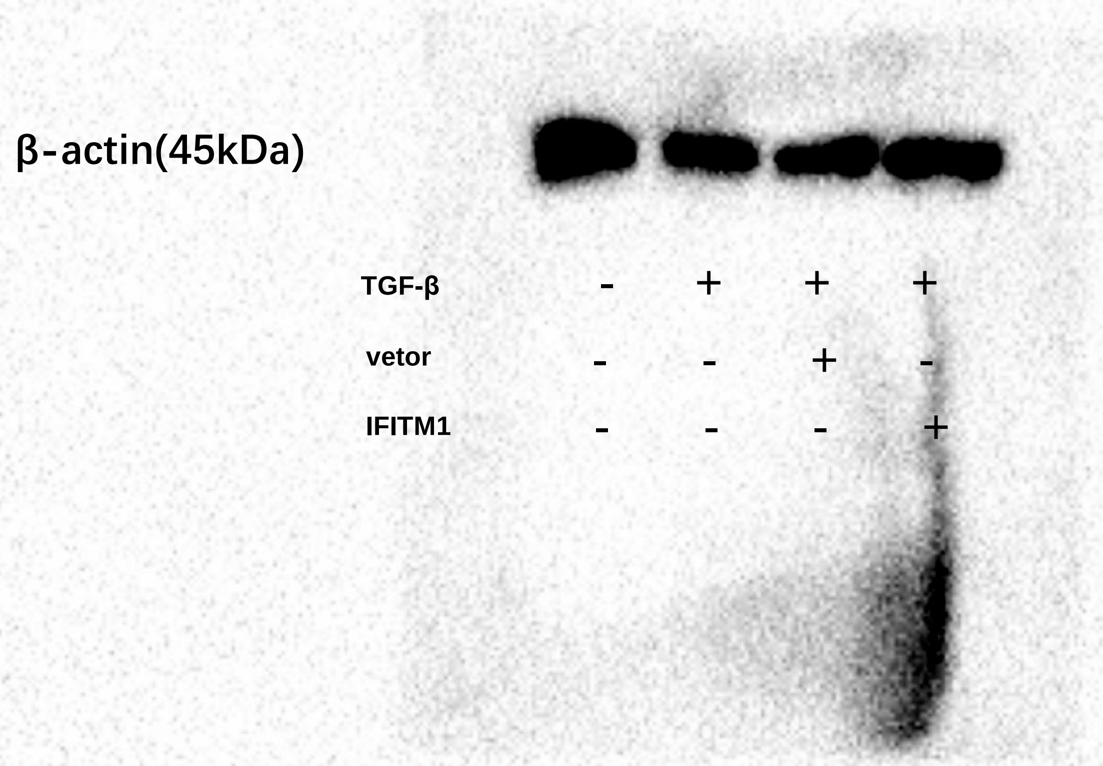
β-actin(45kDa)
TGF-β - + + +
vetor - - + -
IFITM1 - - - +

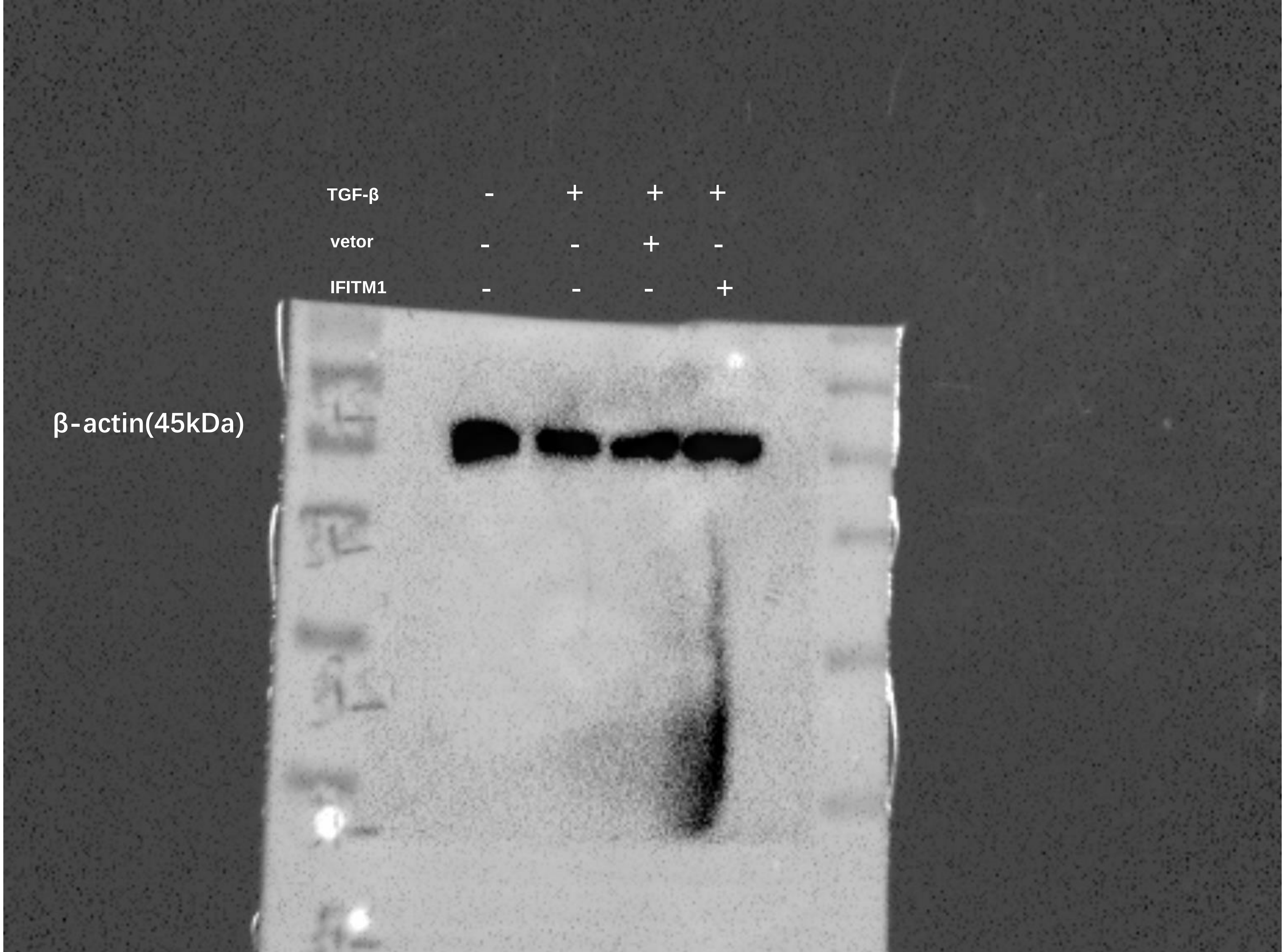
TGF-β - + + +
vetor - - + -
IFITM1 - - - +
β-actin(45kDa)

figure7-E-3

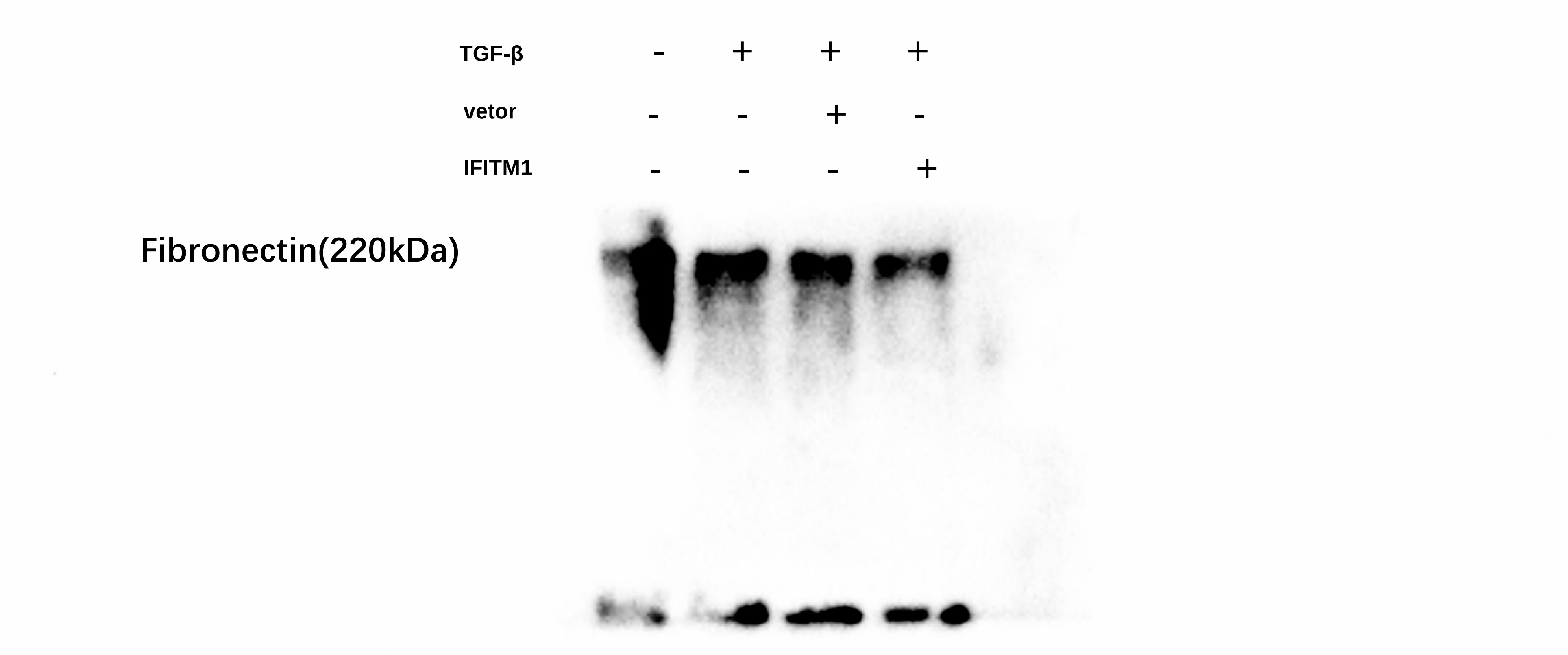

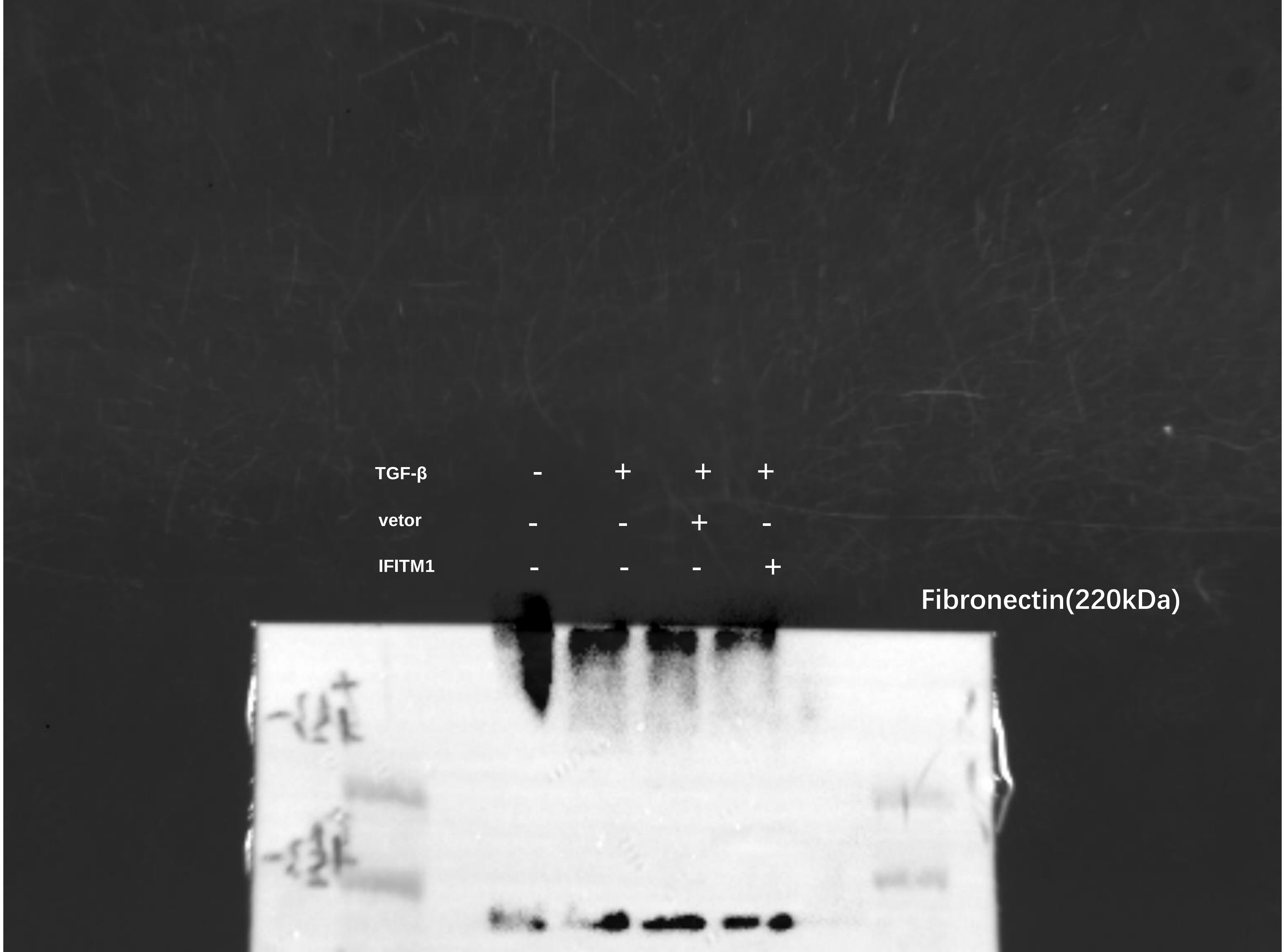
TGF-β - + + +
vetor - - + -
IFITM1 - - - +
Fibronectin(220kDa)

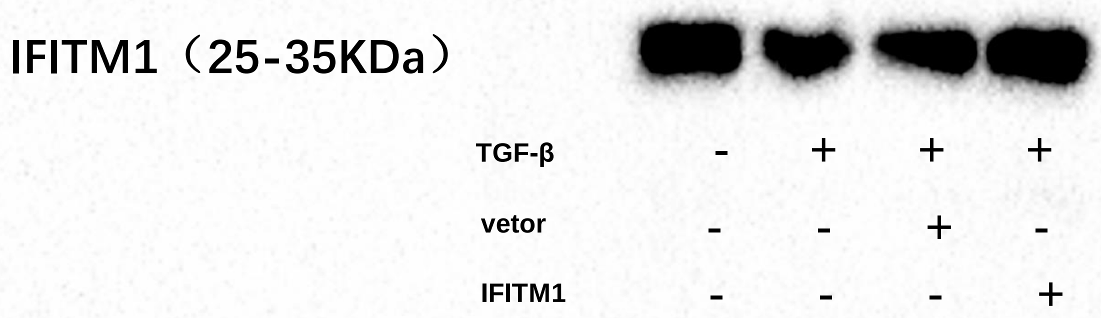
IFITM1（25-35KDa）
TGF-β - + + +
vetor - - + -
IFITM1 - - - +

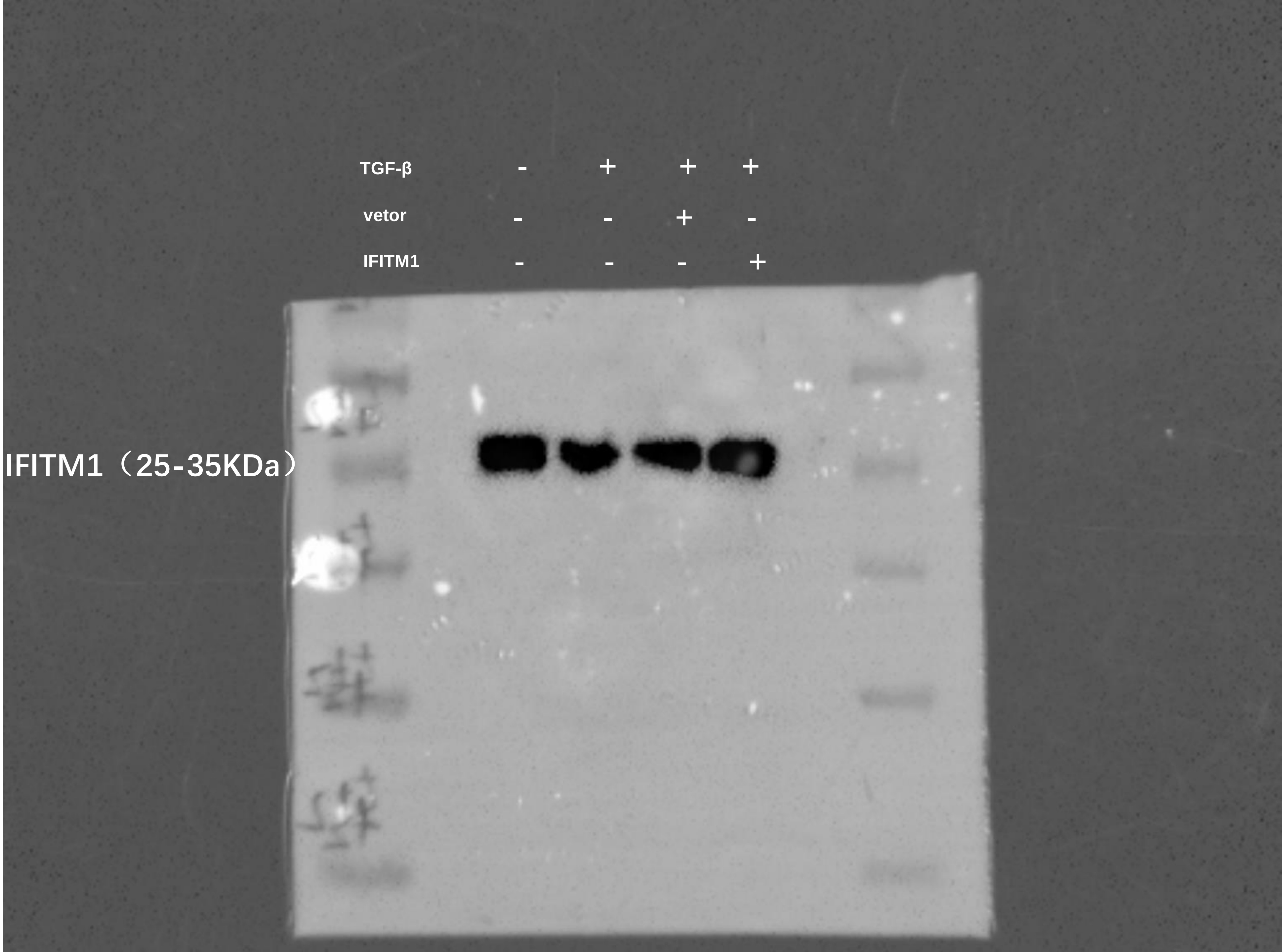
TGF-β - + + +
vetor - - + -
IFITM1 - - - +
IFITM1（25-35KDa）

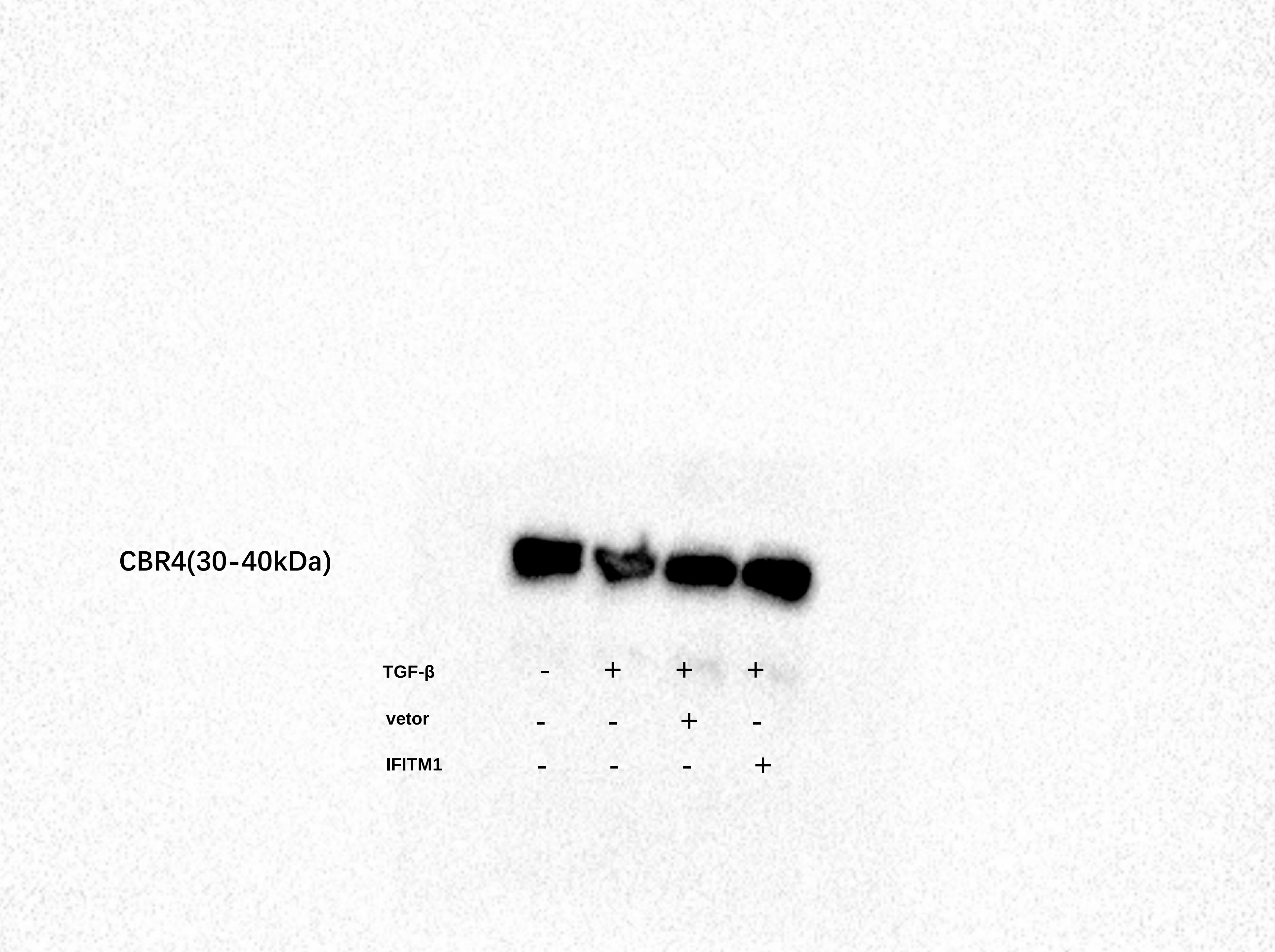
CBR4(30-40kDa)
TGF-β - + + +
vetor - - + -
IFITM1 - - - +

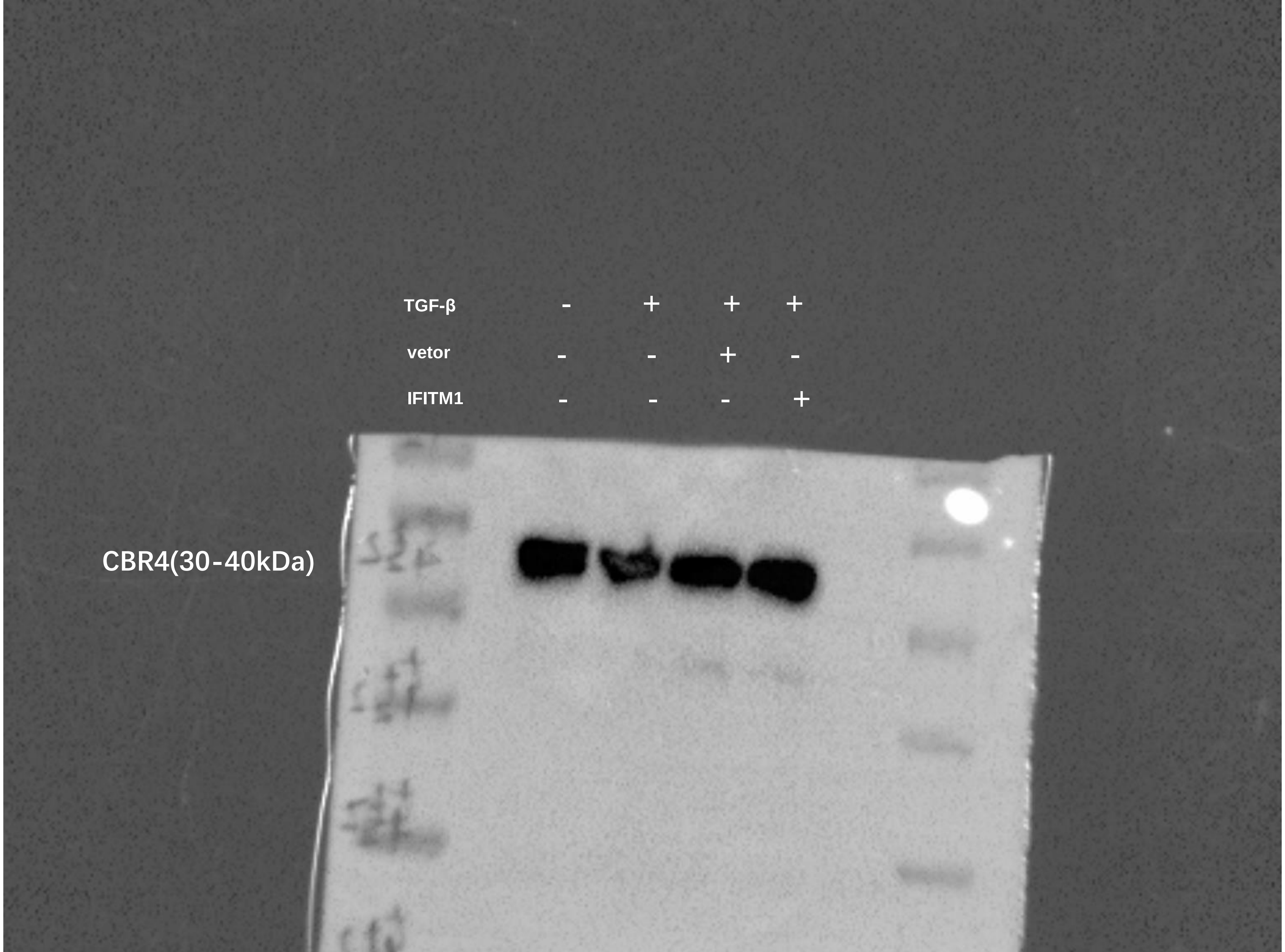
TGF-β - + + +
vetor - - + -
IFITM1 - - - +
CBR4(30-40kDa)

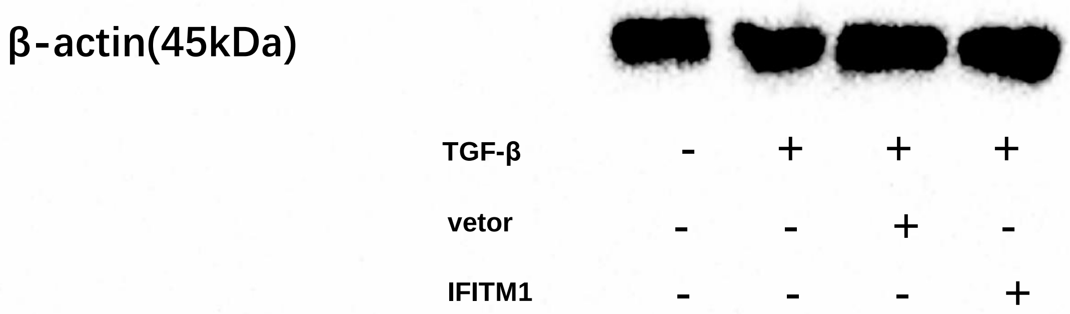
β-actin(45kDa)
TGF-β - + + +
vetor - - + -
IFITM1 - - - +

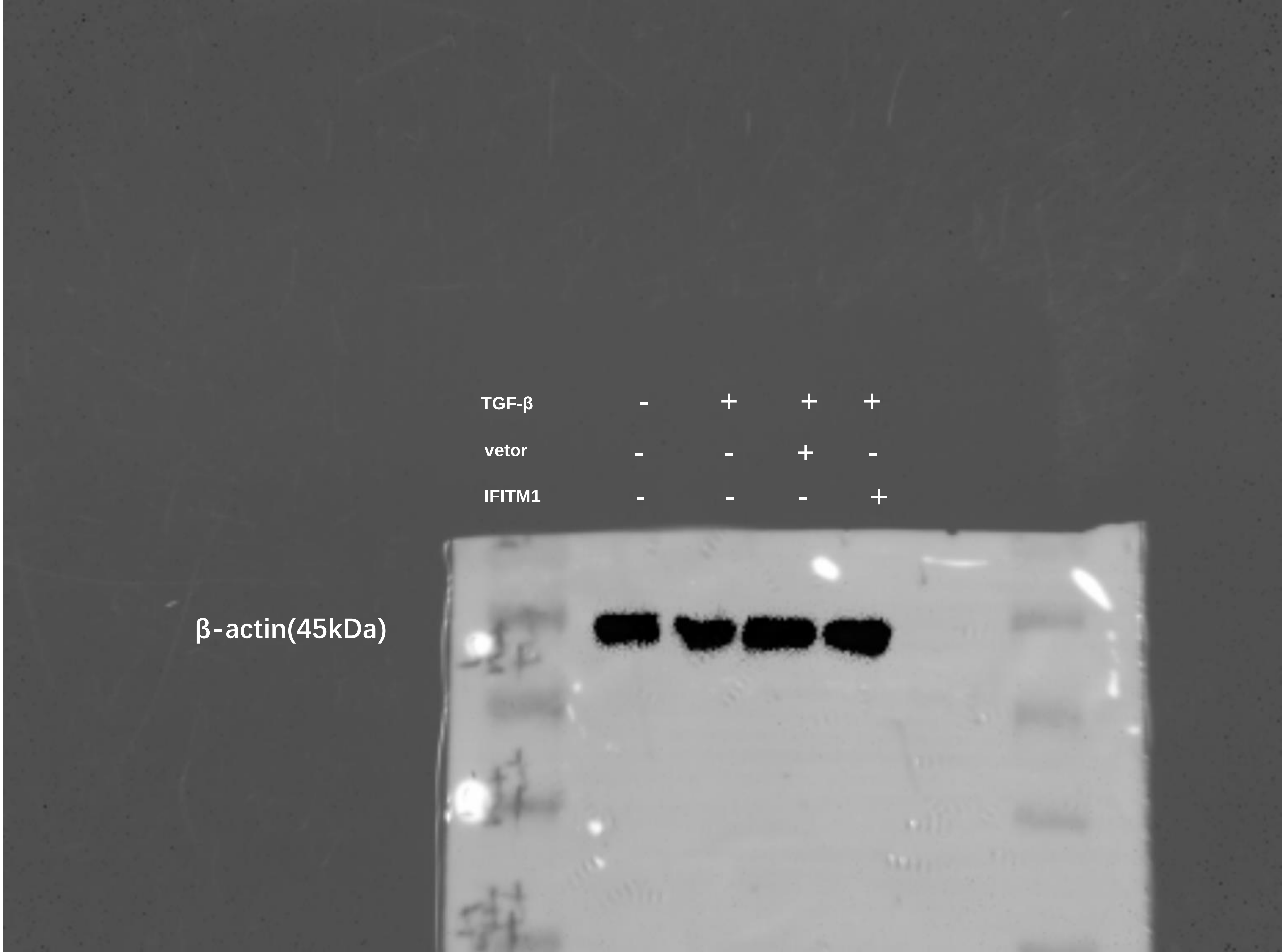
TGF-β - + + +
vetor - - + -
IFITM1 - - - +
β-actin(45kDa)

figure7-F-1

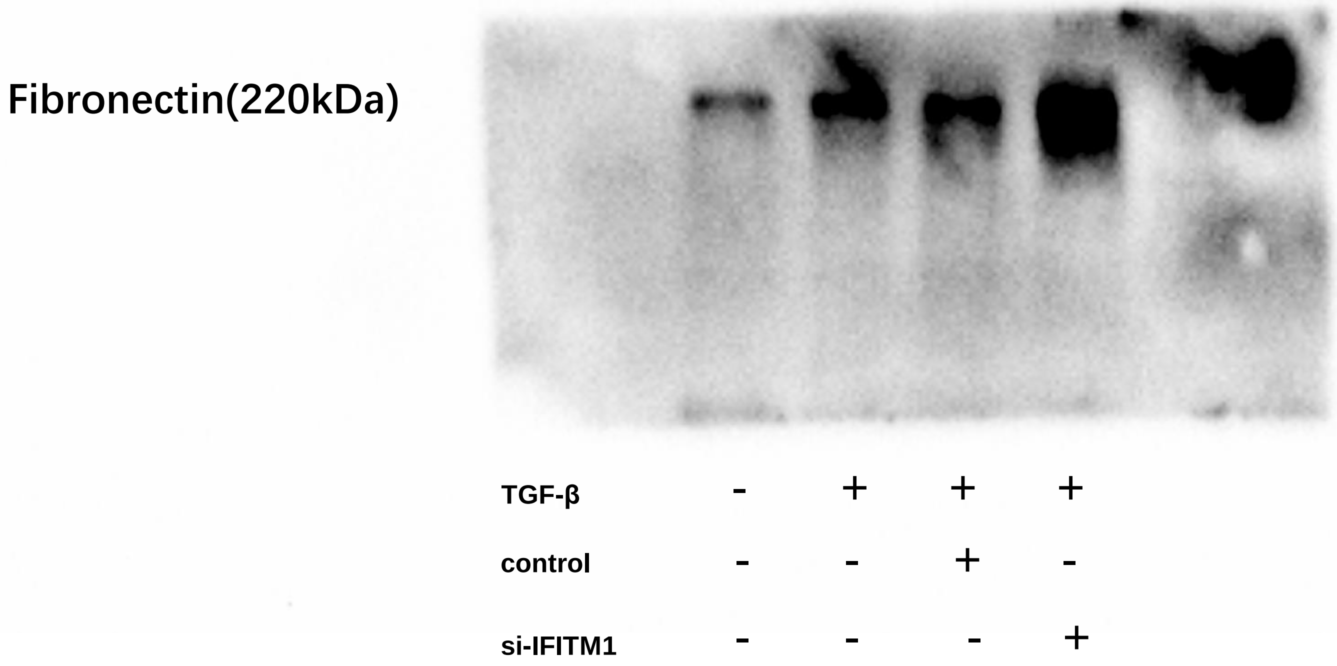

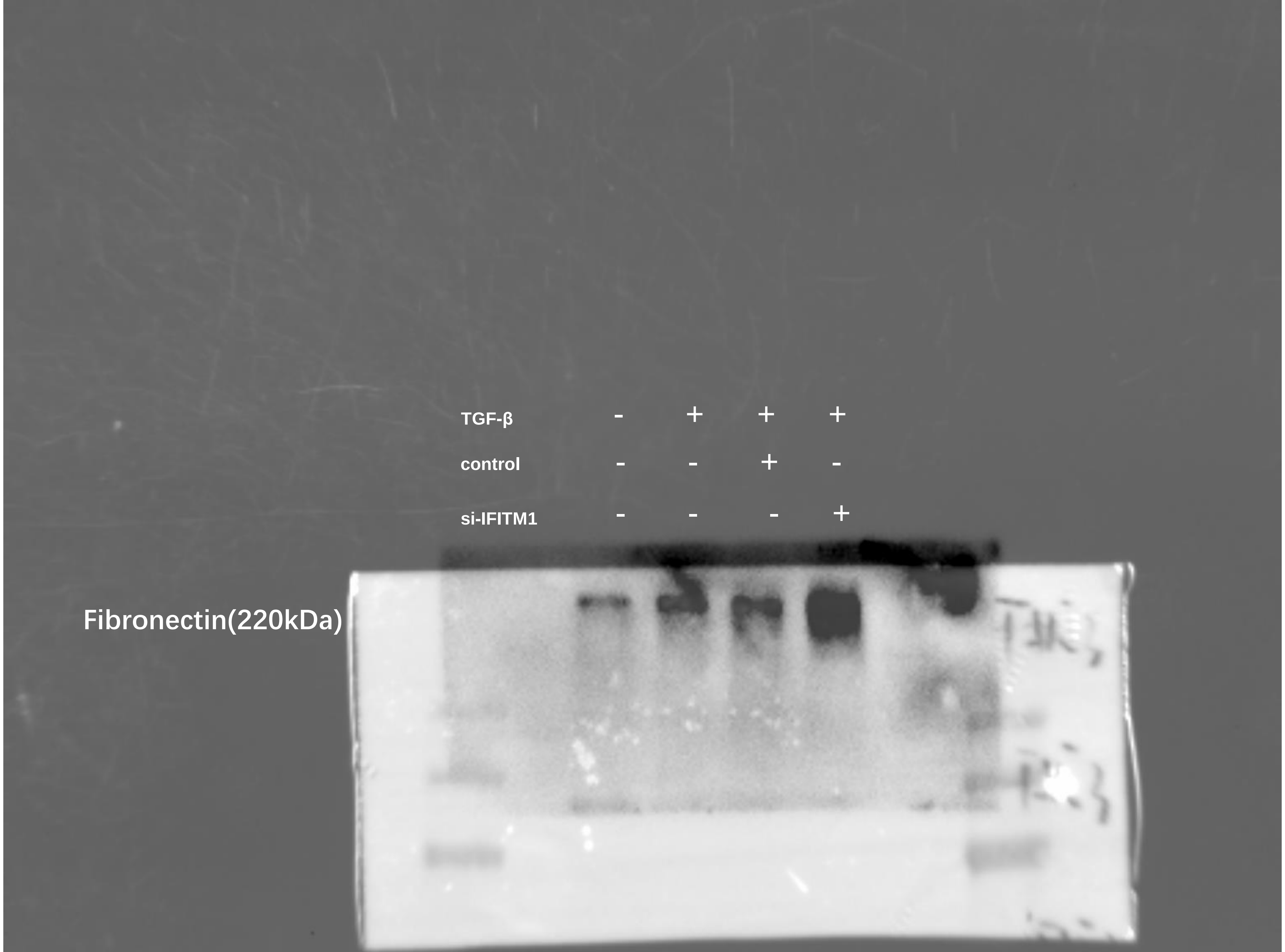
TGF-β - + + +
control - - + -
si-IFITM1 - - - +
Fibronectin(220kDa)

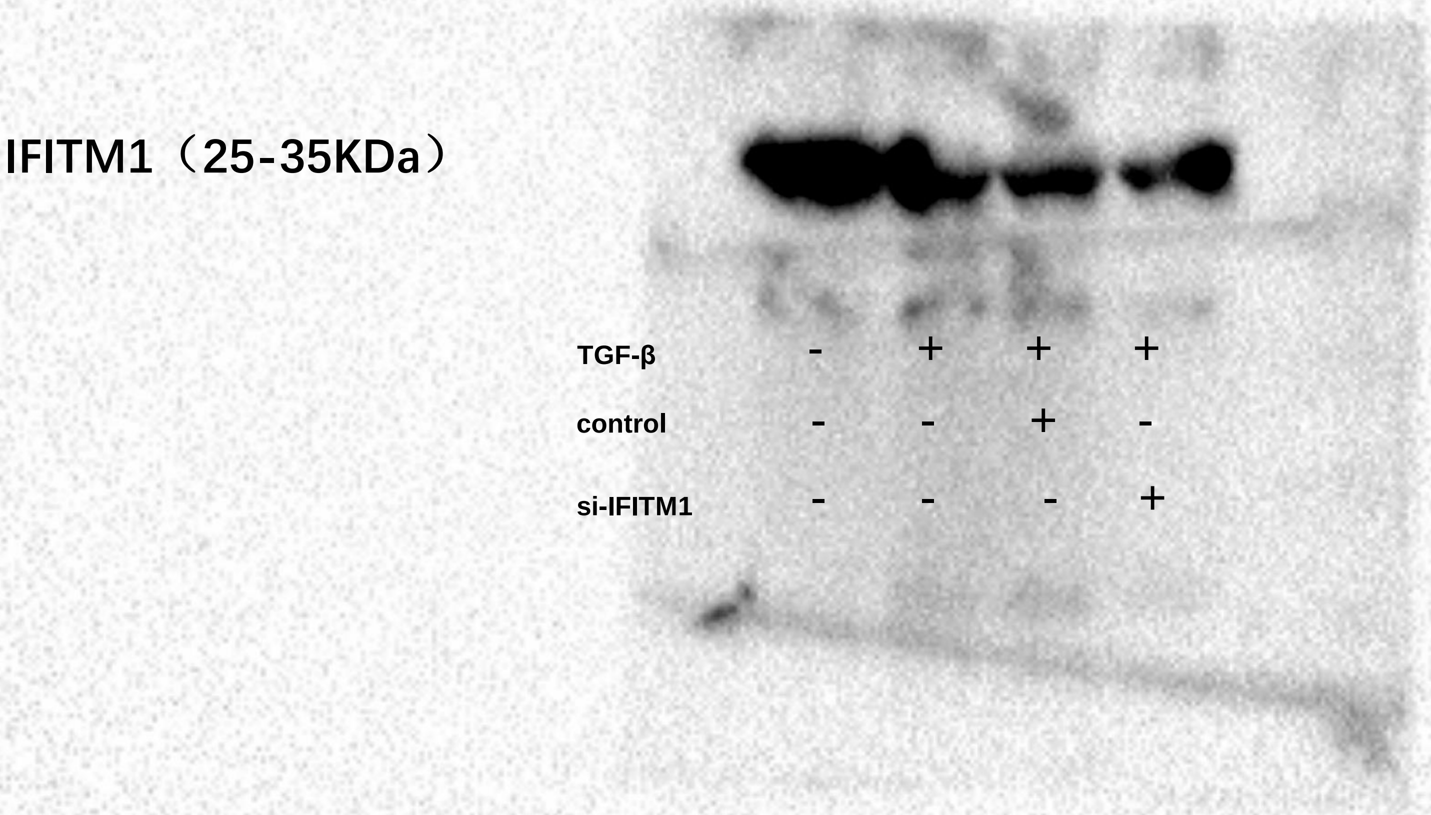
IFITM1（25-35KDa）
TGF-β - + + +
control - - + -
si-IFITM1 - - - +

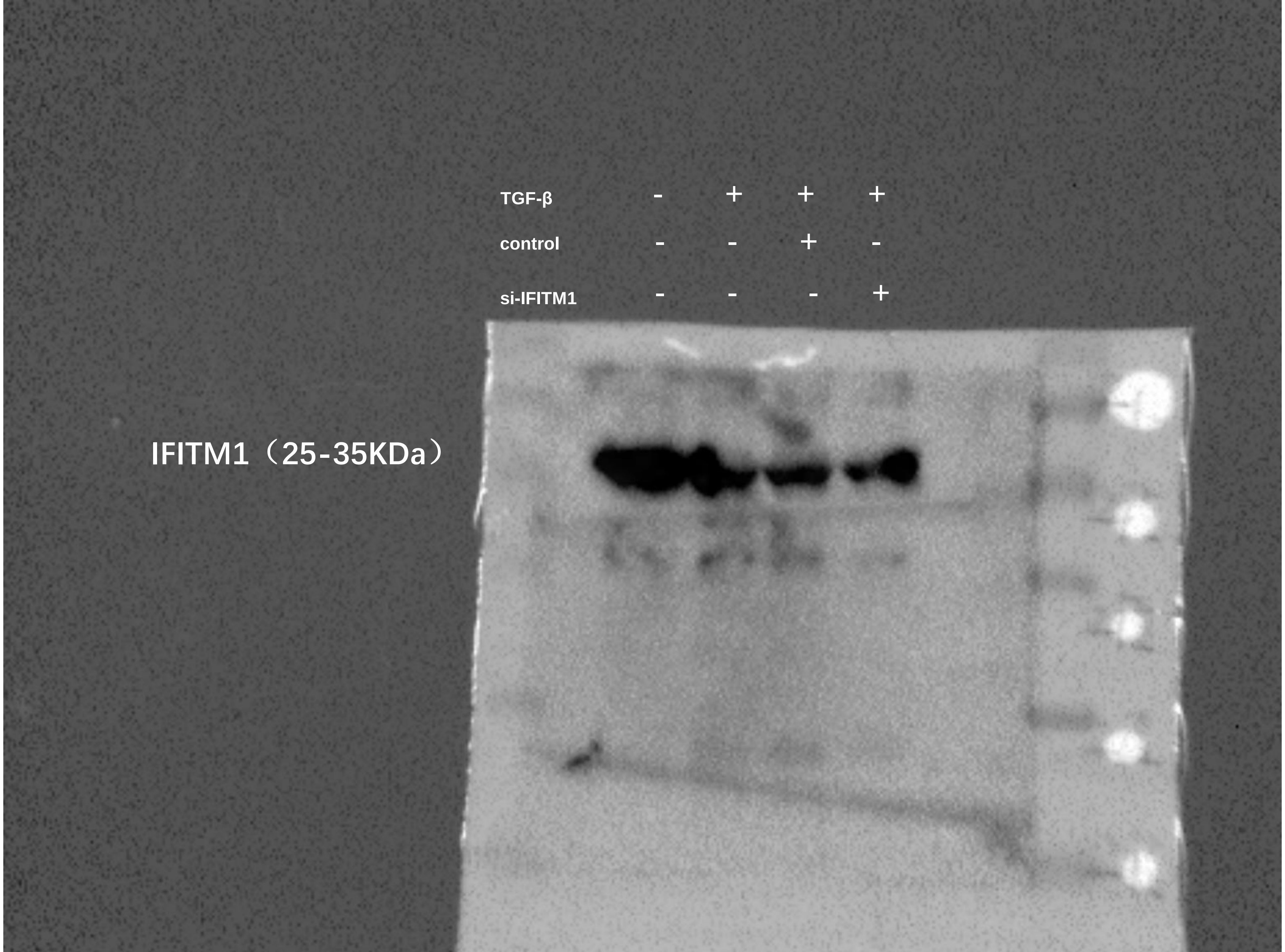
TGF-β - + + +
control - - + -
si-IFITM1 - - - +
IFITM1（25-35KDa）

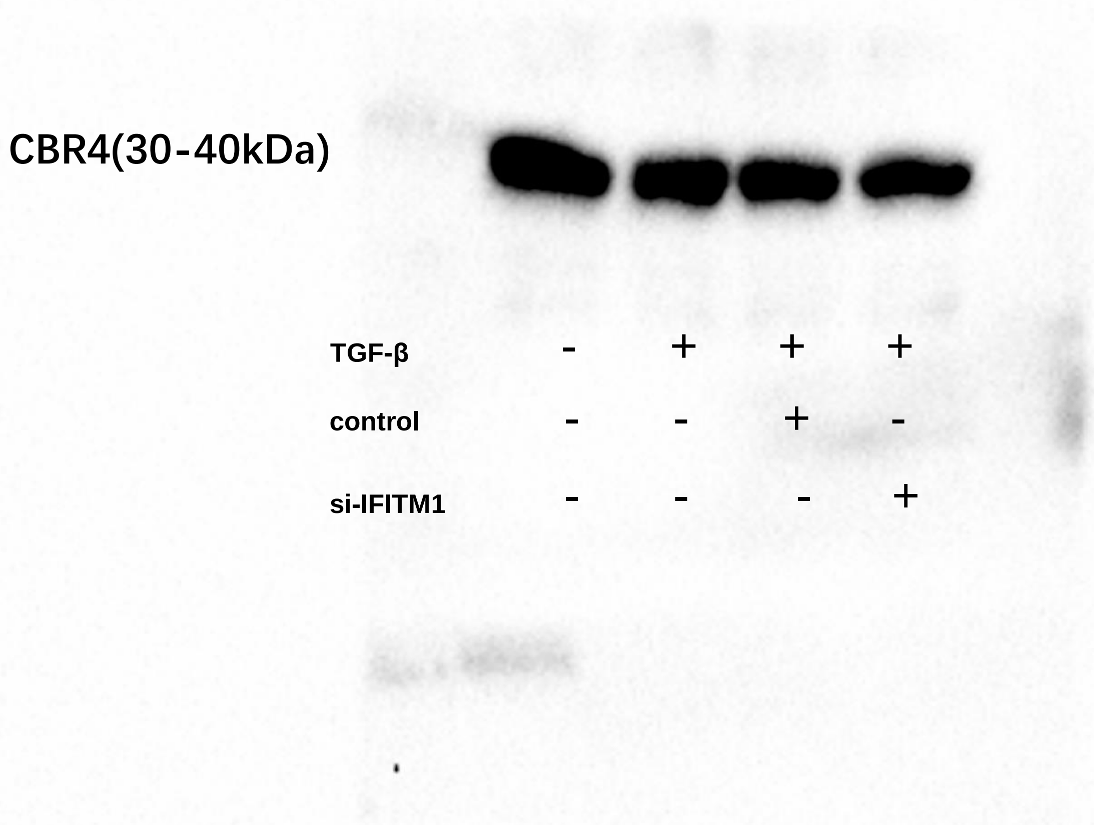
CBR4(30-40kDa)
TGF-β - + + +
control - - + -
si-IFITM1 - - - +

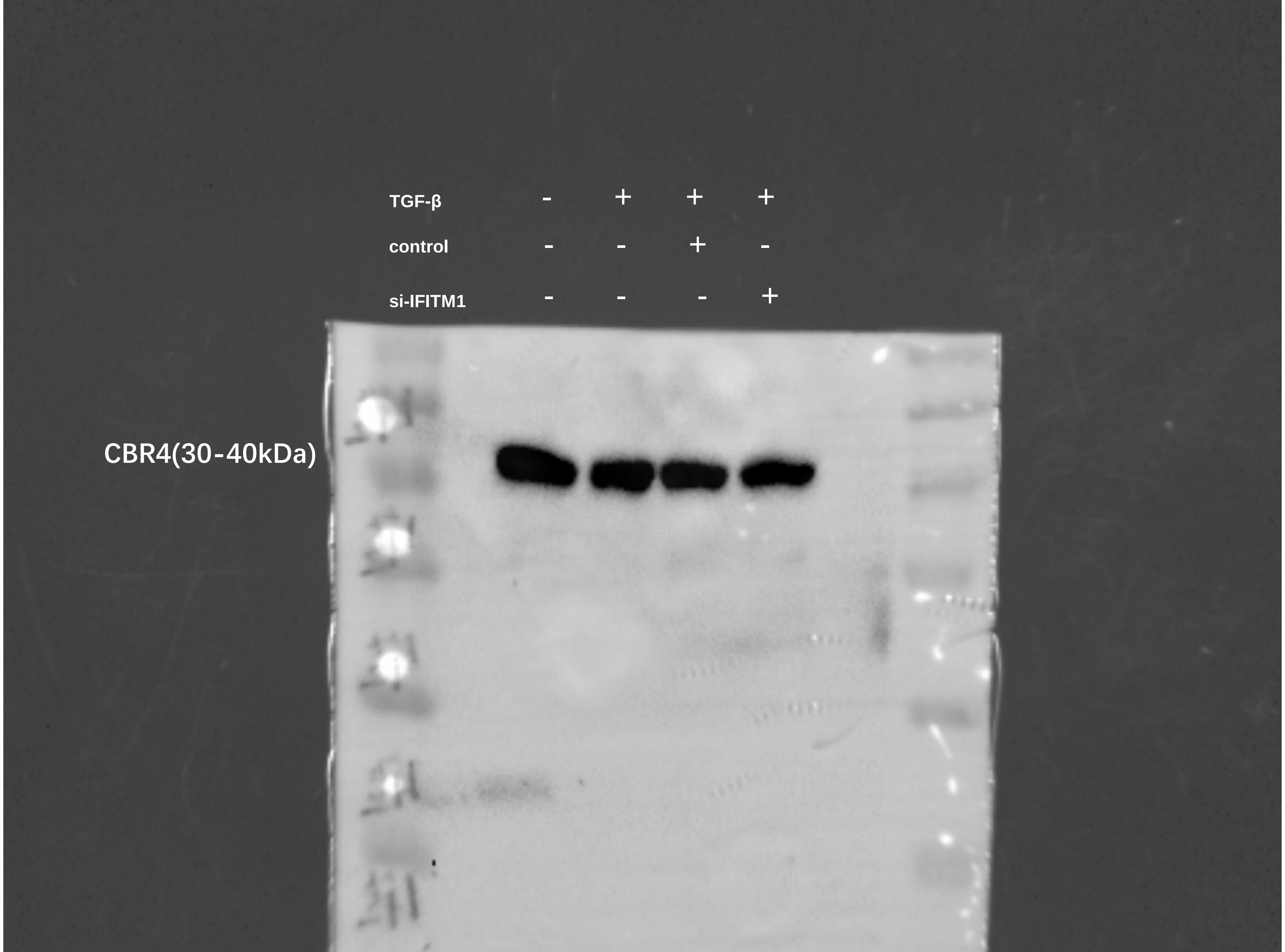
TGF-β - + + +
control - - + -
si-IFITM1 - - - +
CBR4(30-40kDa)

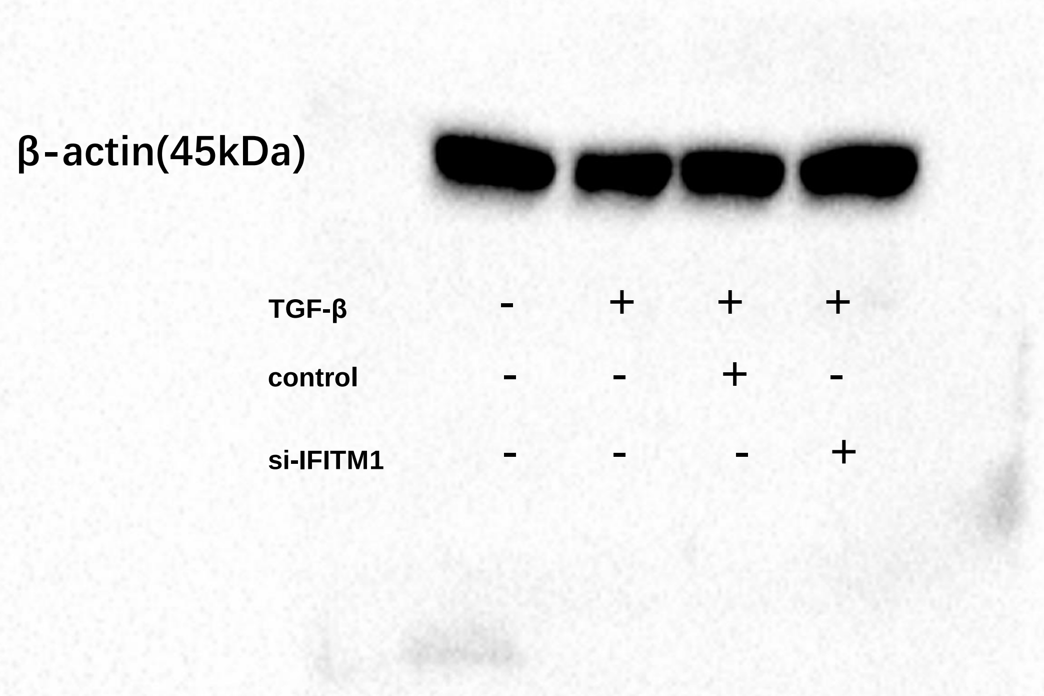
β-actin(45kDa)
TGF-β - + + +
control - - + -
si-IFITM1 - - - +

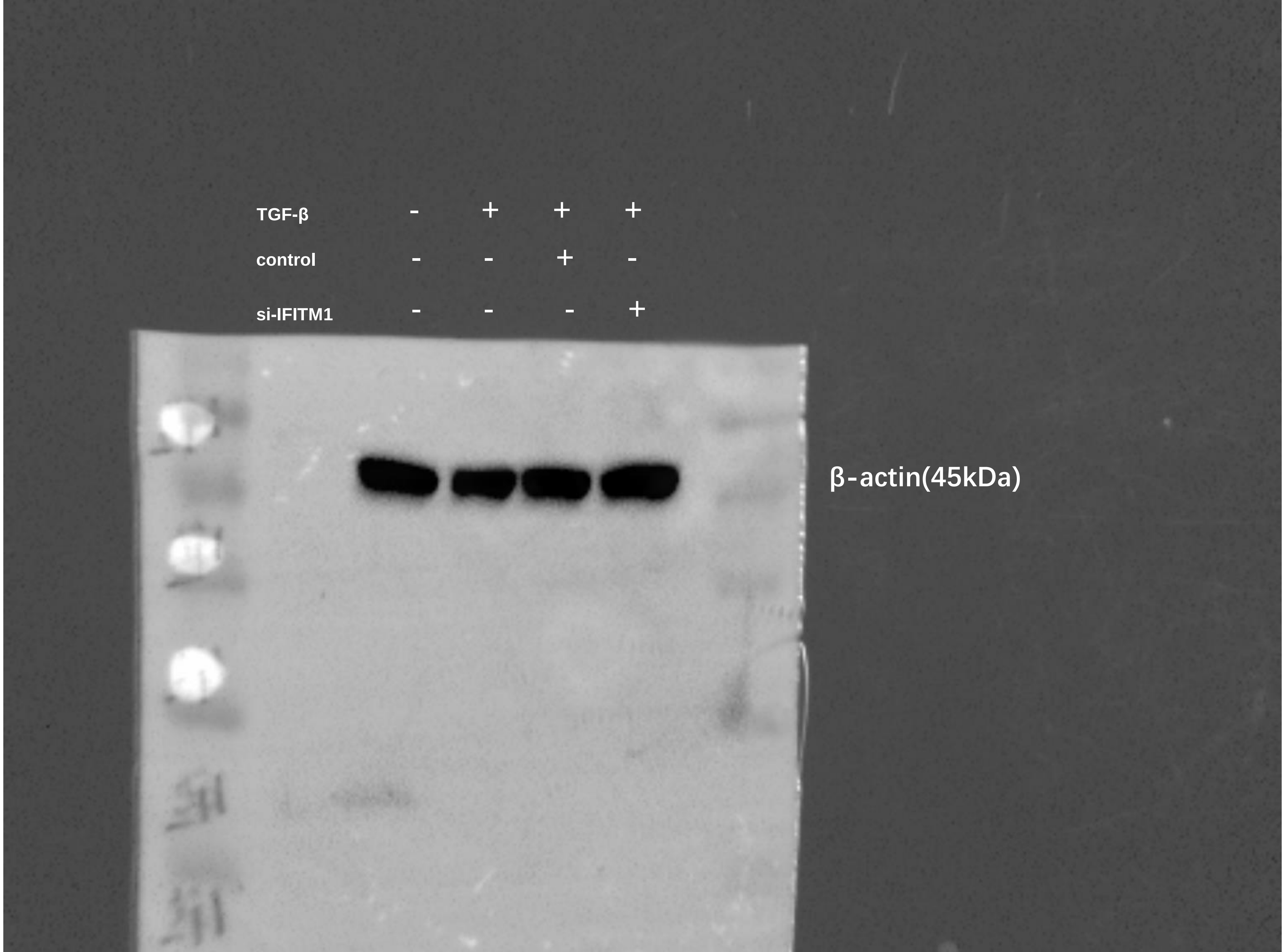
TGF-β - + + +
control - - + -
si-IFITM1 - - - +
β-actin(45kDa)

figure7-F-2

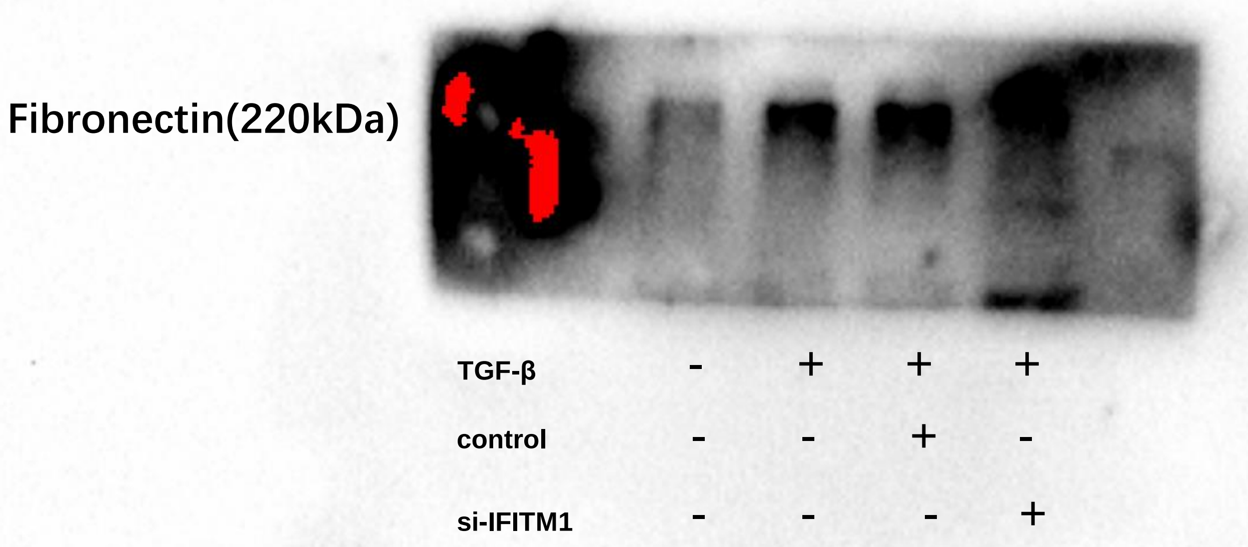

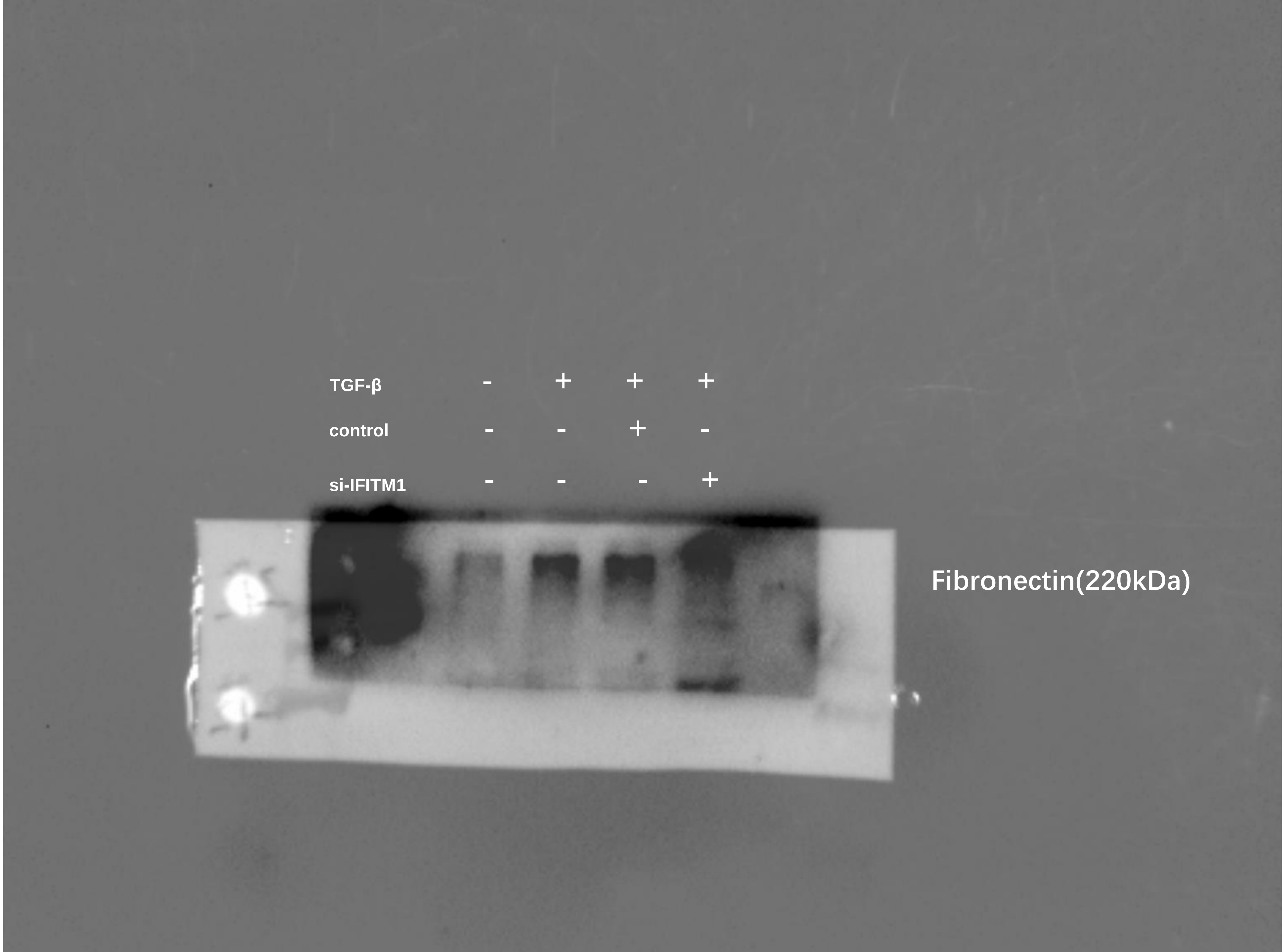
TGF-β - + + +
control - - + -
si-IFITM1 - - - +
Fibronectin(220kDa)

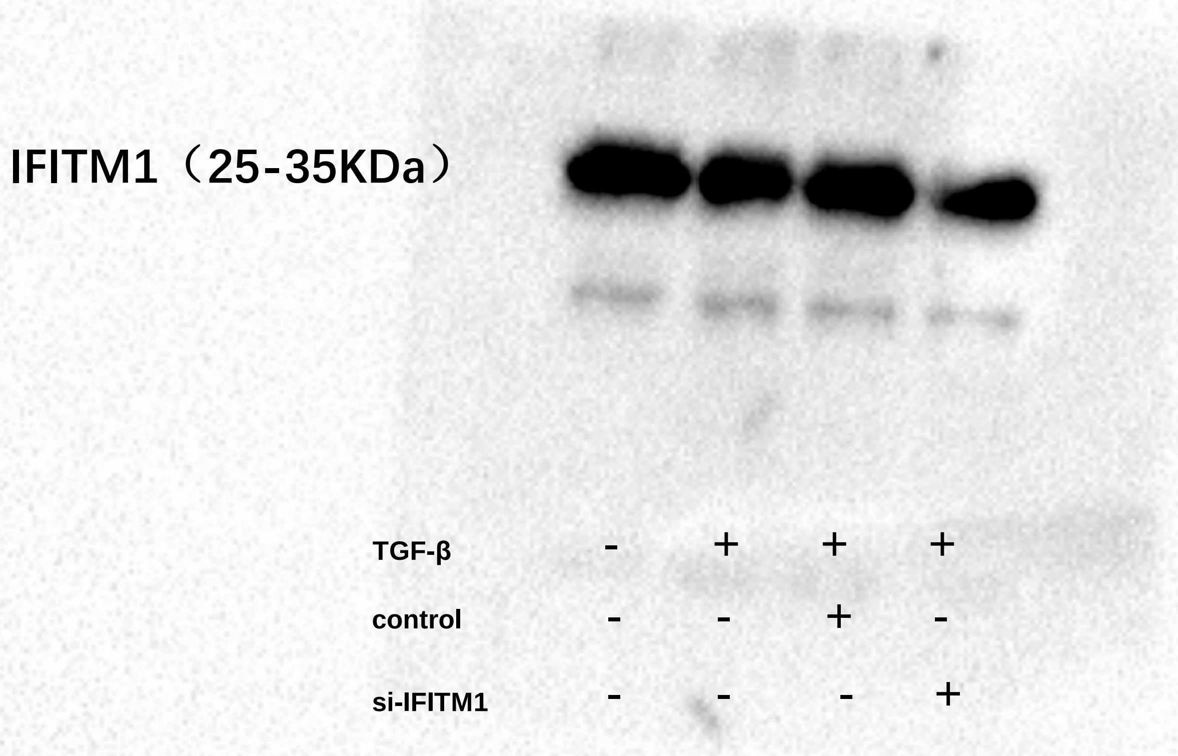
IFITM1（25-35KDa）
TGF-β - + + +
control - - + -
si-IFITM1 - - - +

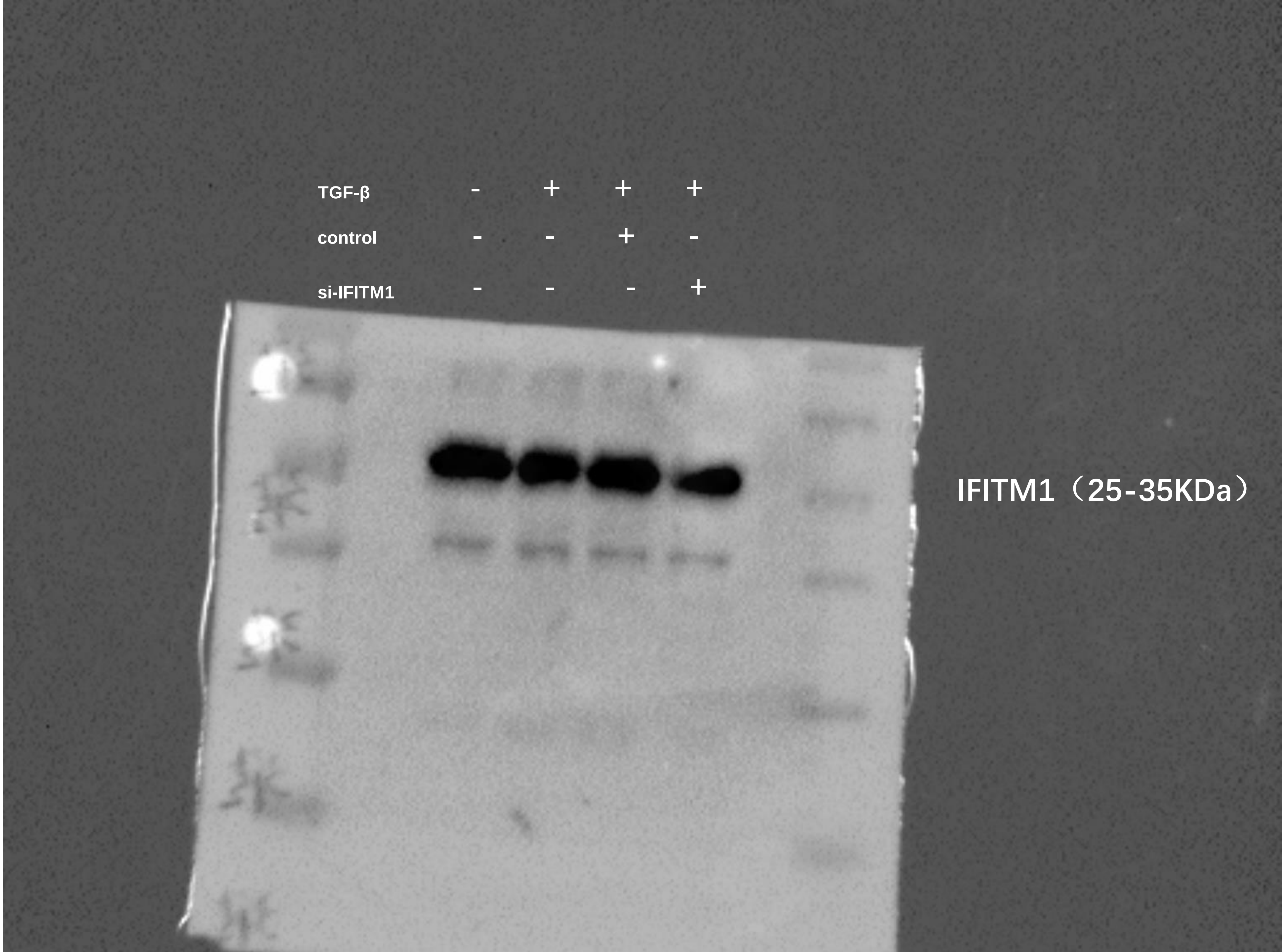
TGF-β - + + +
control - - + -
si-IFITM1 - - - +
IFITM1（25-35KDa）

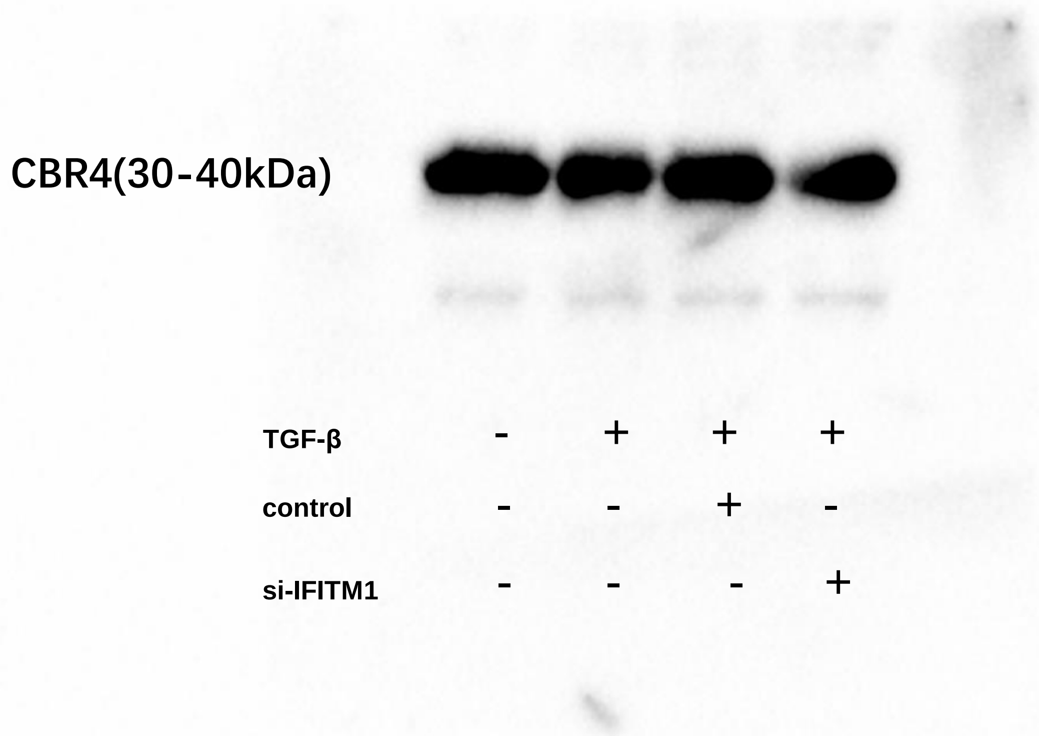
CBR4(30-40kDa)
TGF-β - + + +
control - - + -
si-IFITM1 - - - +

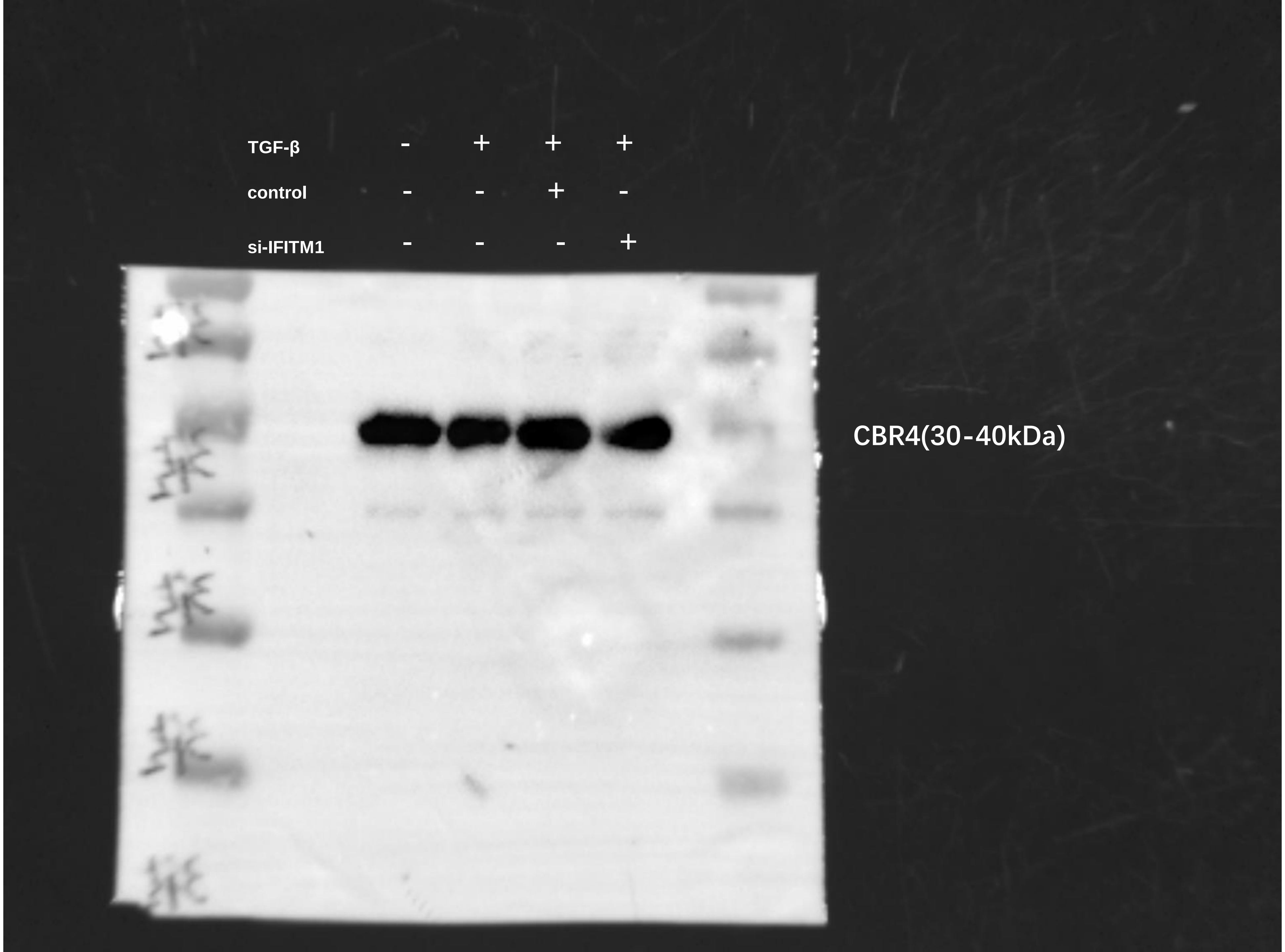
TGF-β - + + +
control - - + -
si-IFITM1 - - - +
CBR4(30-40kDa)

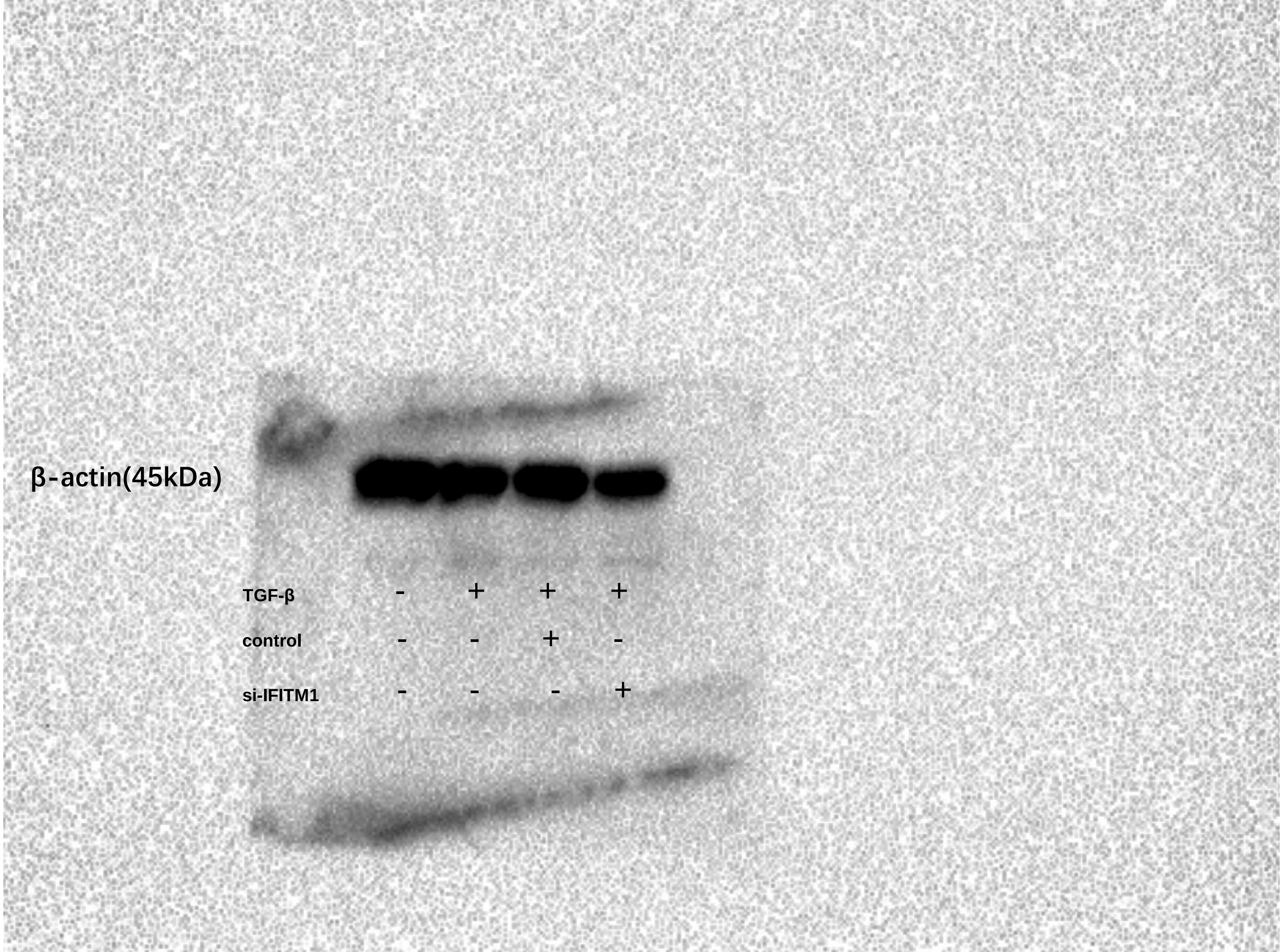
β-actin(45kDa)
TGF-β - + + +
control - - + -
si-IFITM1 - - - +

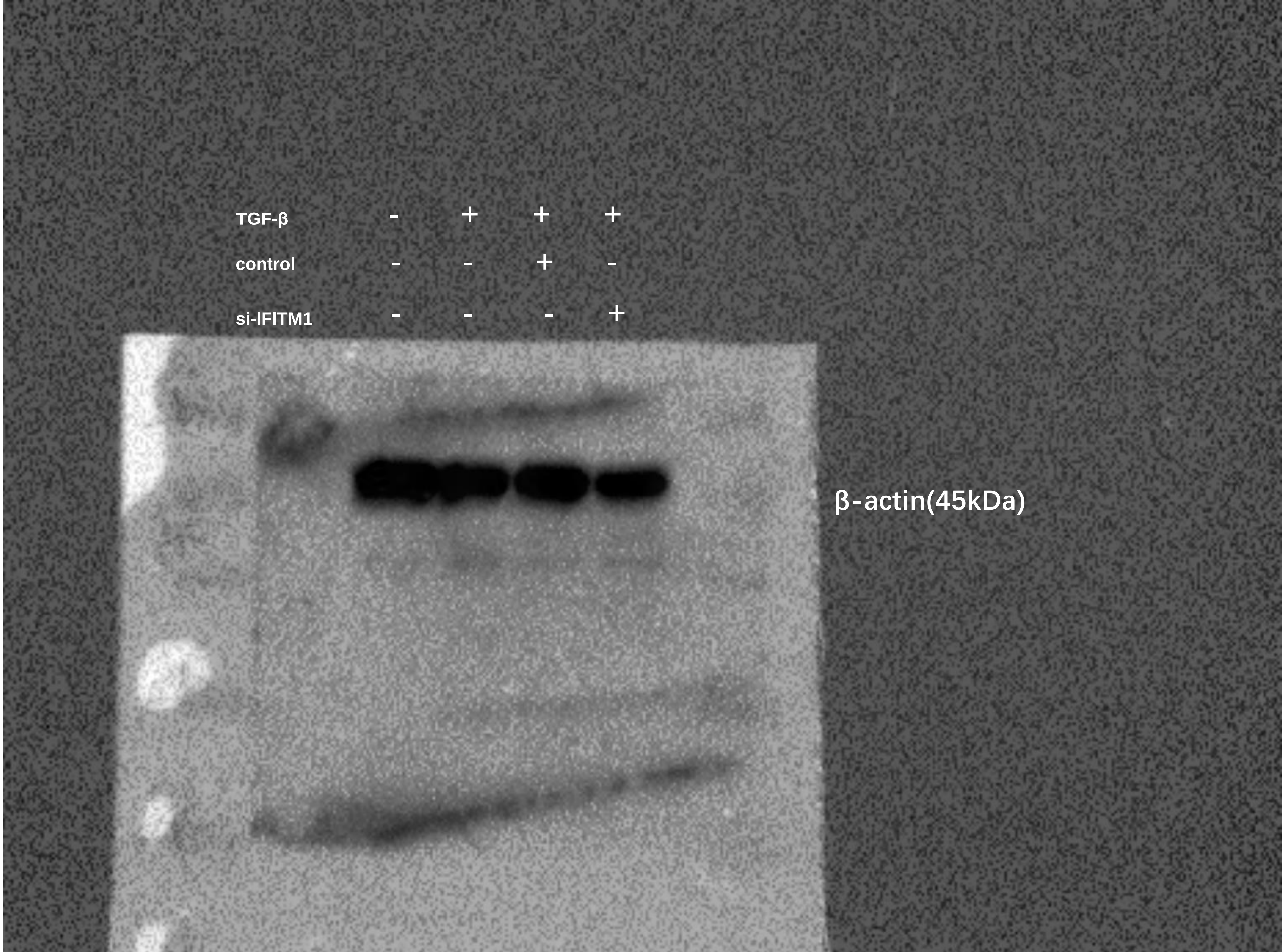
TGF-β - + + +
control - - + -
si-IFITM1 - - - +
β-actin(45kDa)

figure7-F-3

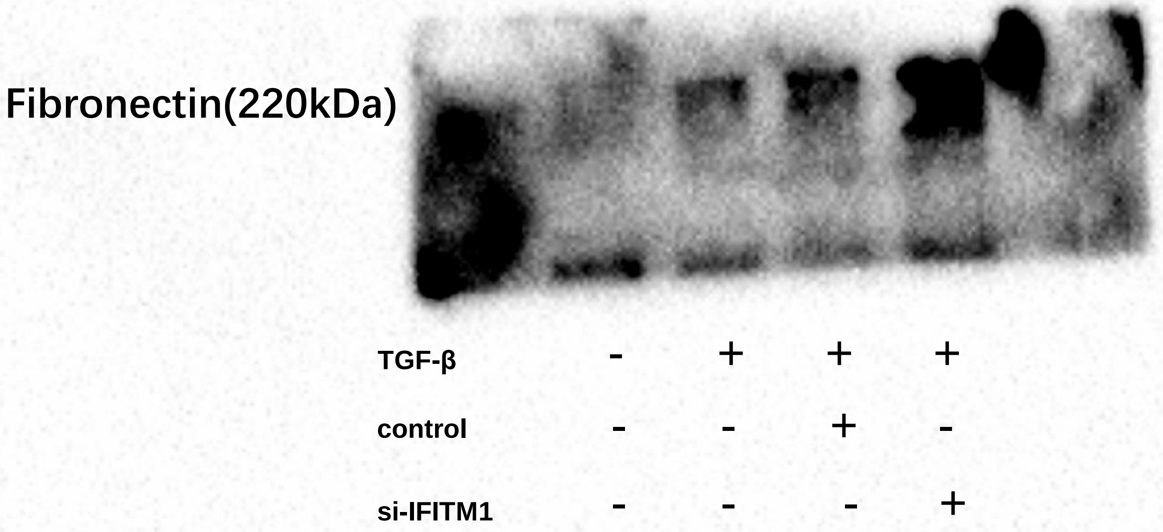

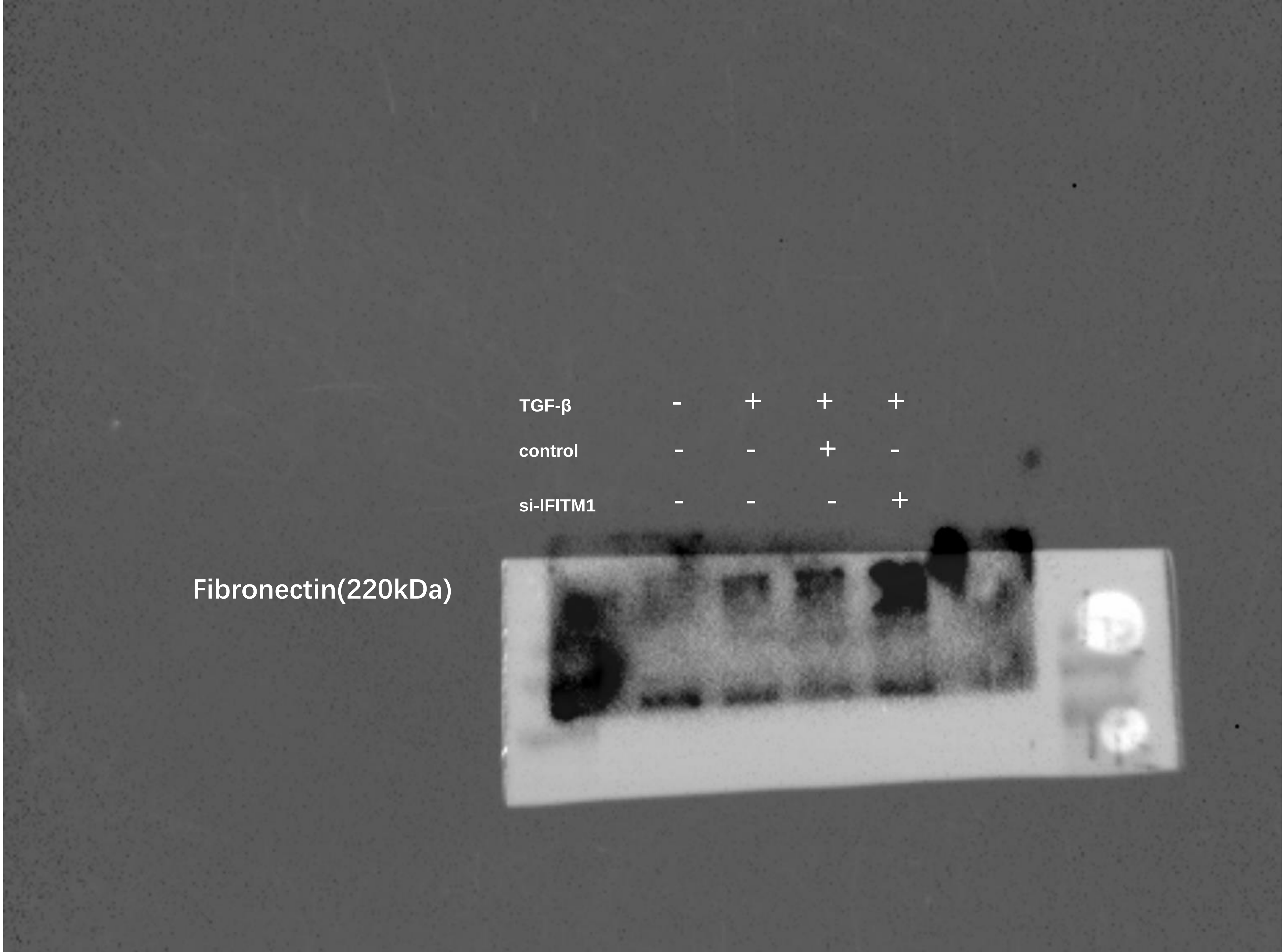
TGF-β - + + +
control - - + -
si-IFITM1 - - - +
Fibronectin(220kDa)

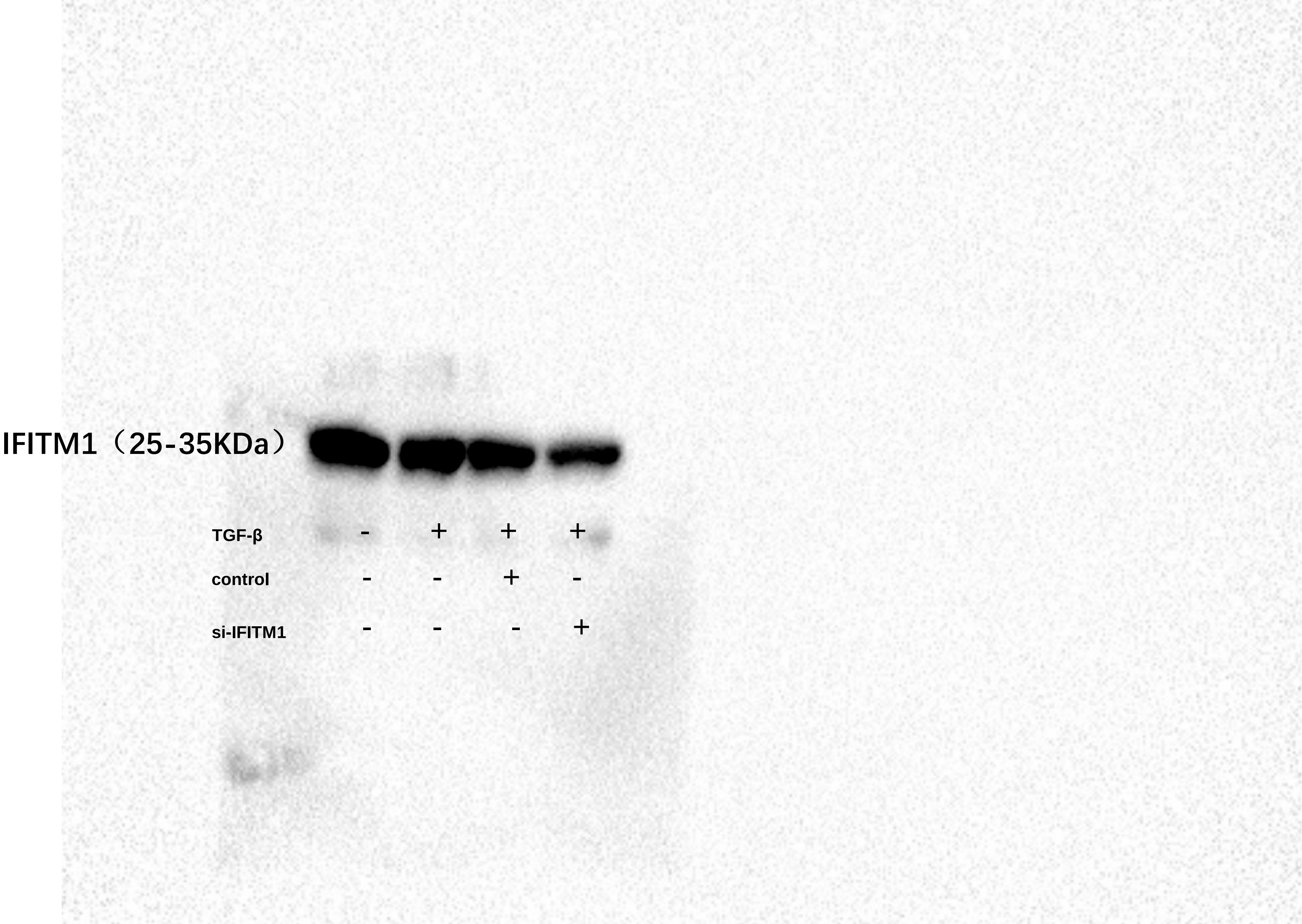
IFITM1（25-35KDa）
TGF-β - + + +
control - - + -
si-IFITM1 - - - +

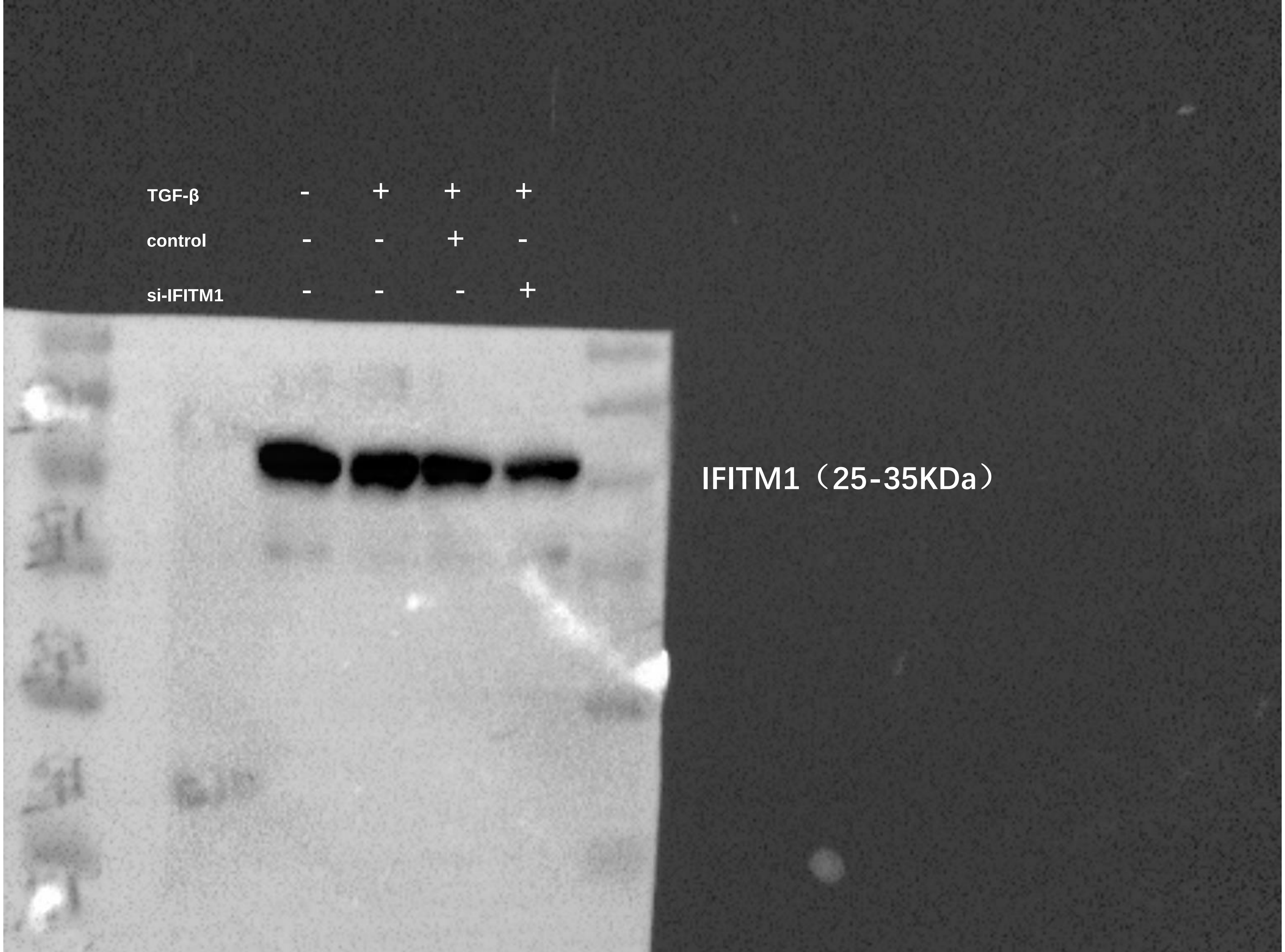
TGF-β - + + +
control - - + -
si-IFITM1 - - - +
IFITM1（25-35KDa）

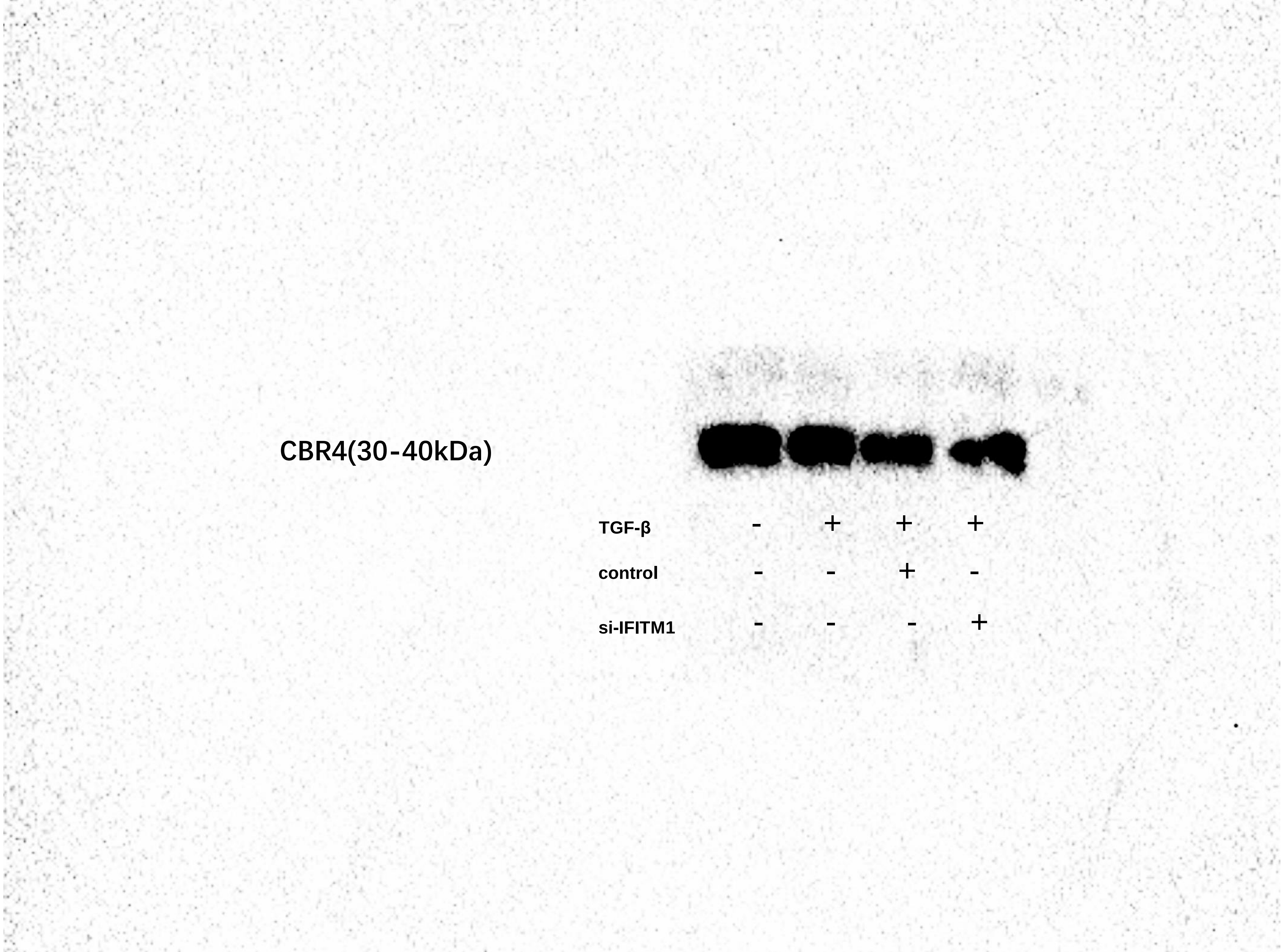
CBR4(30-40kDa)
TGF-β - + + +
control - - + -
si-IFITM1 - - - +

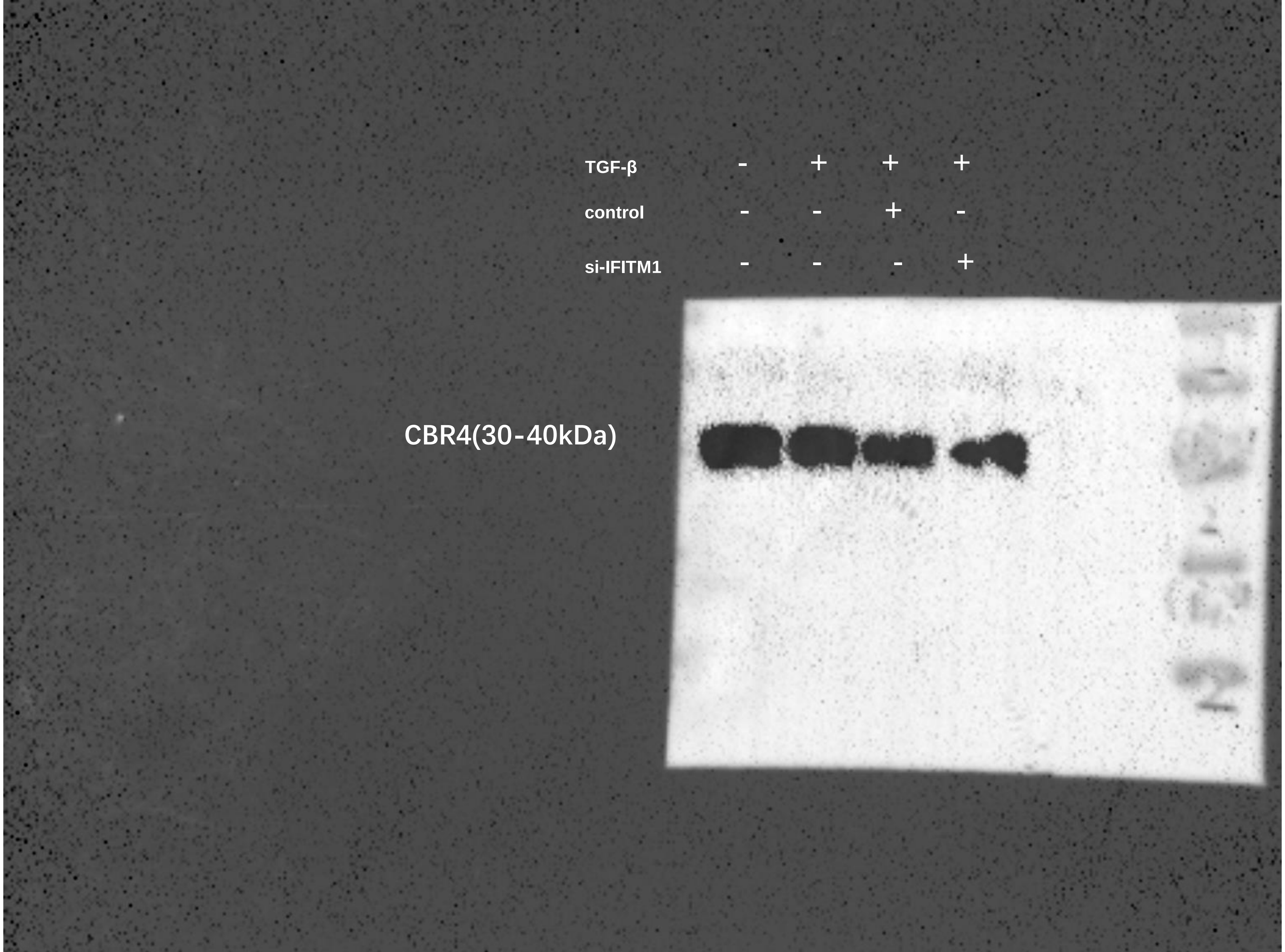
TGF-β - + + +
control - - + -
si-IFITM1 - - - +
CBR4(30-40kDa)

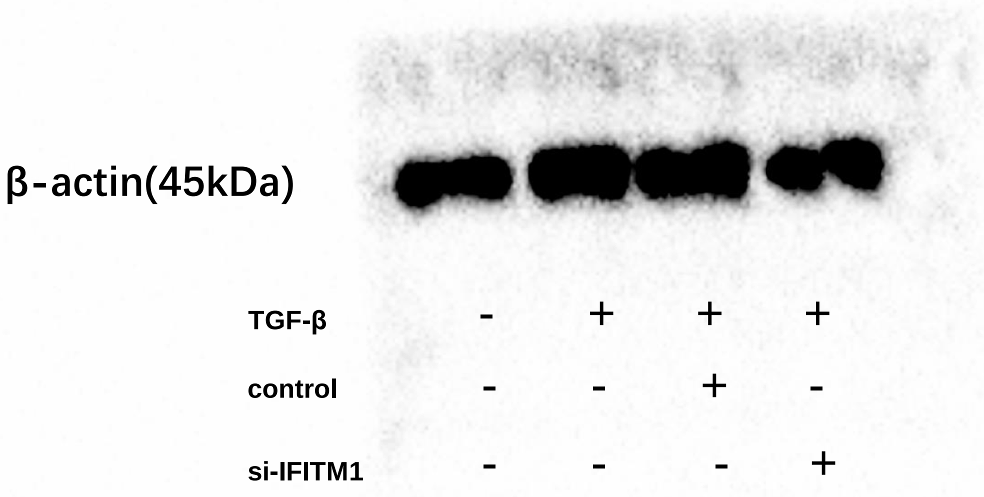
β-actin(45kDa)
TGF-β - + + +
control - - + -
si-IFITM1 - - - +

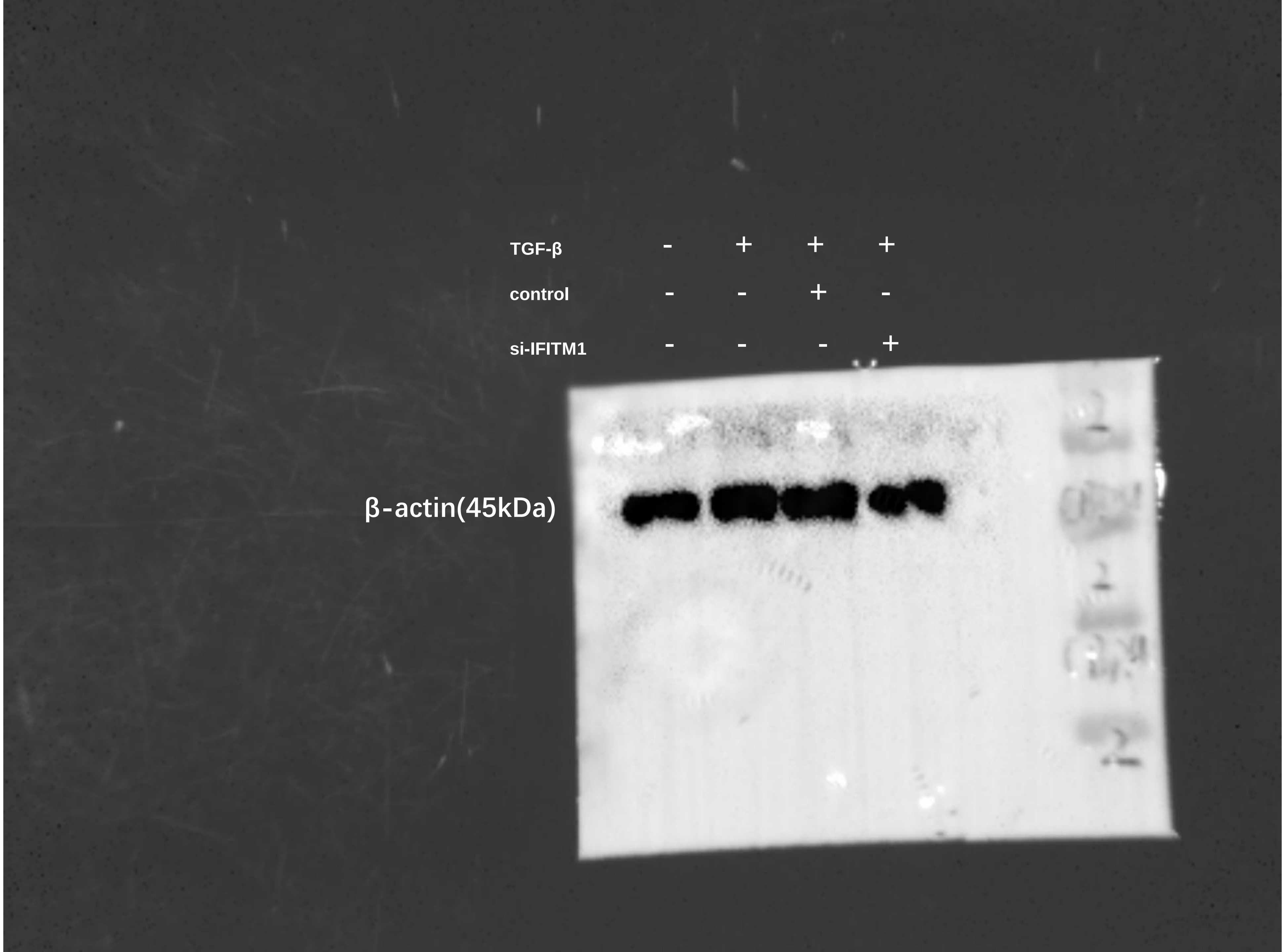
TGF-β - + + +
control - - + -
si-IFITM1 - - - +
β-actin(45kDa)
